# Single-site glycine-specific labeling of proteins

**DOI:** 10.1038/s41467-019-10503-7

**Published:** 2019-06-10

**Authors:** Landa Purushottam, Srinivasa Rao Adusumalli, Usha Singh, V. B. Unnikrishnan, Dattatraya Gautam Rawale, Mansi Gujrati, Ram Kumar Mishra, Vishal Rai

**Affiliations:** 1 Department of Chemistry, Indian Institute of Science Education and Research (IISER) Bhopal, Bhopal, 462066 India; 2 Department of Biological Sciences, Indian Institute of Science Education and Research (IISER) Bhopal, Bhopal, 462066 India

**Keywords:** Chemical modification, Chemical tools, Chemical modification, Chemical tools

## Abstract

Labeling of native proteins invites interest from diverse segments of science. However, there remains the significant unmet challenge in precise labeling at a single site of a protein. Here, we report the site-specific labeling of natural or easy-to-engineer N-terminus Gly in proteins with remarkable efficiency and selectivity. The method generates a latent nucleophile from N-terminus imine that reacts with an aldehyde to deliver an aminoalcohol under physiological conditions. It differentiates N-Gly as a unique target amongst other proteinogenic amino acids. The method allows single-site labeling of proteins in isolated form and extends to lysed cells. It administers an orthogonal aldehyde group primed for late-stage tagging with an affinity tag, ^19^F NMR probe, and a fluorophore. A user-friendly protocol delivers analytically pure tagged proteins. The mild reaction conditions do not alter the structure and function of the protein. The cellular uptake of fluorophore-tagged insulin and its ability to activate the insulin-receptor mediated signaling remains unperturbed.

## Abstract (web summary)

Single-site labelling of proteins is desirable, e.g., for analytical purposes. Here, the authors developed a method in which they use an aldol-type reaction to modify proteins at N-terminal glycine residues in an efficient and selective manner, which is also applicable to cell lysates.

## Introduction

**Fig. 1:**
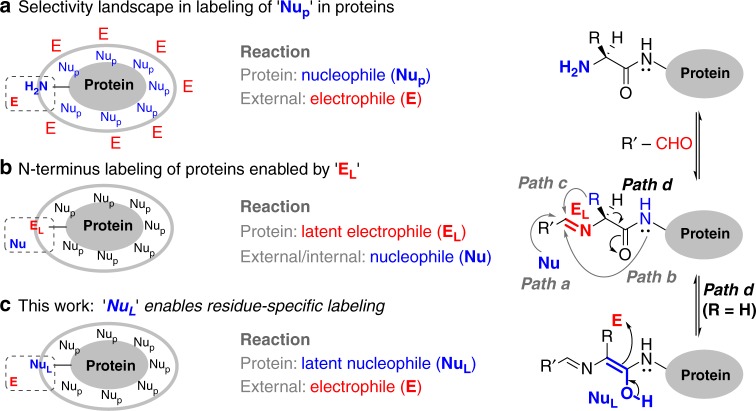
Residue-specificity in labeling of proteins. **a** Nucleophilic addition of protein residues to an electrophile. **b** A latent electrophile, imine, renders the N-terminus labeling of proteins (path a–path c). **c** Latent nucleophile enables single-site N-terminus Gly labeling

Single-site labeling of proteins facilitates investigation of several biological processes through attachment of biophysical probes, imaging probes, and toxins. Such labeling emerges through pre-engineered protein equipped with unnatural amino acids^1^, an amino acid sequence recognized by enzymes^2^, and single cysteine^3,4^ or a cysteine placed in a π-clamp^5^. The understanding of organic chemistry with proteins has nurtured the growth of bioconjugation. These biomolecules can be perceived as a macromolecular substrate with multiple nucleophilic functional groups (Nu_P_). The obvious route for bioconjugation would involve its reaction with an electrophile (Fig. 1a). For single-site labeling in such cases, a functional group has to compete with all the other nucleophilic residues (chemoselectivity) and its additional copies (site-selectivity). The chemoselective labeling of low-frequency residues (Cys^6,7^, Tyr^8^, and Trp^9^) can address the latter to some extent. For other cases, the single-site labeling of side chain functionalities could be driven through ligand–protein interaction^10^, linchpin directed modification^11^ or targeting the ε-amine of most reactive Lys residue (N^ε^-NH_2_)^12–16^. Alongside, the N-terminus α-amine (N^α^-NH_2_) has established its place as a notable reactivity hotspot. In a remarkable example, the biomimetic transformation of N-terminus residue delivers a carbonyl group primed for subsequent orthogonal reactions^17^.

The kinetic labeling of N^α^-NH_2_ can be achieved through its chemoselective modification with an electrophile as determined by a few groups including us (Fig. 1a)^18–30^. The alternate route involves a latent electrophile (E_L_) generated in the form of imine from N^α^-NH_2_ and aldehyde (Fig. 1b). It gives us the opportunity to address the two challenges in two separate steps. If we can generate E_L_ chemoselectively, the nucleophile is only required to address the site-selectivity. This approach delivers reductive alkylation in a chemoselective transformation (path a, Fig. 1b)^31^. However, the site-selectivity gets compromised very early in the reaction in the presence of competing imines. On the contrary, the nucleophilic attack of backbone amide to imine generated by N^α^-NH_2_ and 2-pyridinecarboxaldehyde can render isolable imidazolidinone with high selectivity (path b, Fig. 1b)^32^. Unfortunately, none of these methodologies can distinguish one N-terminus residue from the other. In this perspective, N-terminal Cys containing proteins can render unique reactivity to form thiazolidine with an aldehyde or 2-cyanobenzothiazole (path c, Fig. 1b)^33–35^. However, it is a challenge to identify unique reactivity for other N-terminus residues. In particular, selective targeting of N-terminus Gly poses a prominent complexity as there is no side-chain residue to assist.

Here, we demonstrate that an aldehyde with appropriately designed hydrogen bond acceptor can result in the exclusive labeling of N-terminus Gly residue. The remarkable selectivity of C–C bond formation extends from an amino acid to structurally diverse proteins. Besides, the protocol operates conveniently in a mixture of proteins or cell lysate. The residue-specific installation of an orthogonal aldehyde group renders analytically pure tagged proteins in high yields through the late-stage single-site introduction of the desired probe.

## Results

We hypothesized that a multi-step transformation could provide a roadmap to address the challenge of chemoselectivity and residue selectivity. The initial generation of the E_L_ with capability to generate another reactive center (latent nucleophile, Nu_L_, path d) could provide the platform. While the first step can address chemoselectivity, the latter can give us an opportunity to explore the residue specificity. Also, the Nu_L_ generated with a protein will be required to display orthogonal reactivity in the presence of proteinogenic nucleophiles (Fig. 1c). At first, the challenge was to design an imine that can generate the Nu_L_ under physiological conditions. We hypothesized that H-bond acceptors in the imine group (Fig. 1b) could assist proton shuttling and stabilize the Nu_L_. We anticipated that the intramolecular H-bond acceptors might overcome the competing intermolecular interactions with protein and solvent. Subsequently, we can explore the reactivity of Nu_L_ towards an external electrophile (Fig. 1c). Also, we anticipated that the unsubstituted amino acid (Gly) could potentially render a reactivity profile different from all the other substituted amino acids.

### Aminoalcohol formation with Gly under ambient conditions

**Fig. 2:**
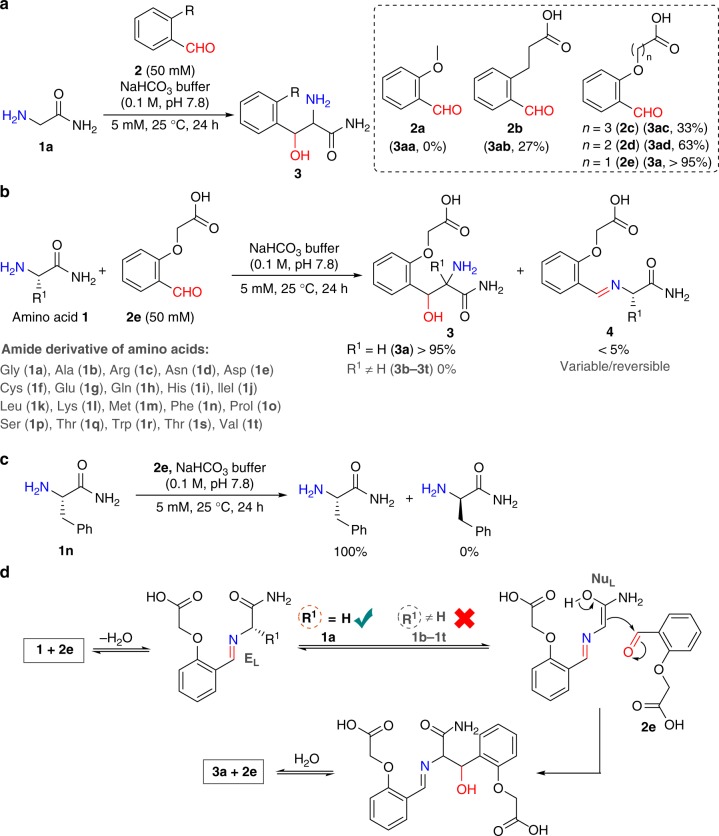
Stable aminoalcohol formation with glycine. **a** Aldehyde with H-bond acceptors forms a stable aminoalcohol with **1a** (also see Supplementary Fig. 72). **b** Selective formation of aminoalcohol with **1a**. **c** Stereo-stability of substituted amino acid (**1n**) under the reaction conditions. **d** Plausible mechanism for glycine specific formation of aminoalcohol

We selected ortho-substituted benzaldehyde to garner the advantage of geometrical constraint imposed by the aromatic ring. We placed one H-bond acceptor ortho to the aldehyde in the form of a methoxy group (**2a**, Fig. 2a). The reaction of Gly amide **1a** with reagent **2a** fails to result in any adduct (Fig. 2a). Next, we designed the reagent **2b** that proffers a carbonyl group as H-bond acceptor. It was exciting to note the formation of isolable product (**3ab**, 27% conversion, LC-MS). Encouraged by this result, we investigated the effect of two oxygen centers as H-bond acceptors in the reagent **2c**. We found it to be inefficient in improving the conversion to the product (**3ac**, 33%, LC-MS). The redesigned reagent **2d** led to 63% conversion of the product that further enhanced (**3a**, > 95%, LC-MS) with reagent **2e**. A non-polar derivative of product (**3a**) was purified and characterized as aminoalcohol after thorough mass and NMR analysis (Supplementary Figs. 13–27).

### Unsubstituted amino acid versus substituted amino acids

In another exciting result, we noted that the imine formed by Ala **1b** remained inert towards any subsequent transformation contrary to Gly **1a** (Fig. 2b and Supplementary Table 2). With this data in hand, we examined whether all the other proteinogenic amino acids (**1c**–**1t**) behave similarly to Ala **1b**. No trace of product **3b**–**3t** except imine formation **4b**–**4t** was observed with the amino acids **1b**–**1t** (Fig. 2b and Supplementary Table 2). In a control experiment, the vortexing of Phe (**1n**) under the reaction conditions did not result in its racemization (Fig. 2c and Supplementary Fig. 48). These results reconfirm the presence of a high barrier to the formation of the Nu_L_ in substituted amino acids (**1b**–**1t**, Fig. 2d). We believe that the unfavorable orientation of the H-bond acceptor in these cases inhibit the process. Also, the imine from the Lys side chain (N^ɛ^-NH_2_) remains unreactive for any subsequent transformation (see Supplementary Fig. 73).

### N-terminus Gly labeling of large peptides and proteins

**Fig. 3:**
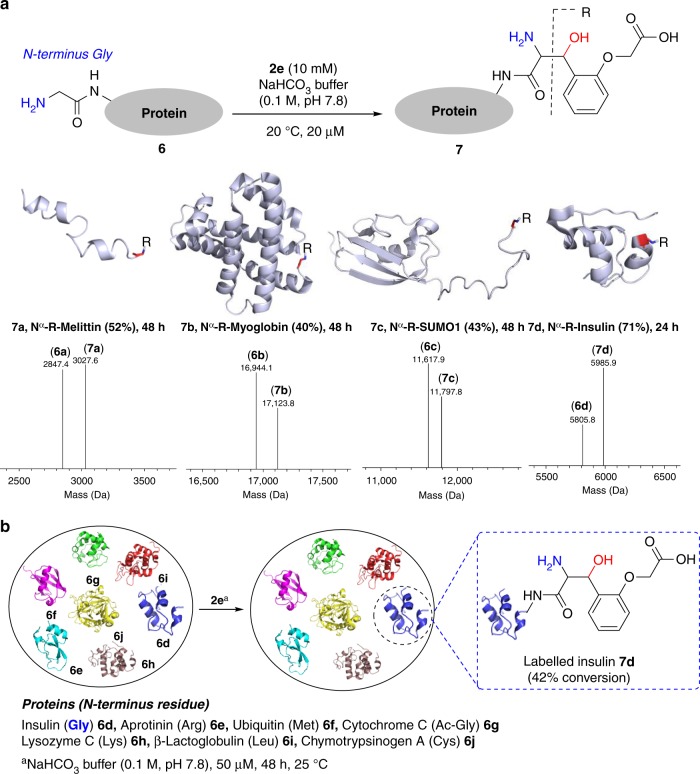
N-terminus Gly labeling of proteins. **a** A user-friendly protocol delivers single-site labeling of proteins. Deconvoluted ESI-MS confirms the %conversion and rules out the possibility of side reactions (also see Supplementary Figs. 70 and 71). **R** indicates the fragment from reagent **2e** attached to the protein. **b** Extension of methodology for labeling single protein (insulin **6d**) in a mixture of proteins (**6d**–**6j**)

To explore the translation of aminoalcohol chemistry, we selected melittin **6a** [26 residues, one N^α^-NH_2_ (Gly), three Lys N^ε^-NH_2_]. The reaction of reagent **2e** with **6a** led to mono-labeled melittin **7a** (52% conversion, Fig. 3a); in a control experiment, RNase A (N-terminus Lys) resulted in no detectable product in 72 h. We confirmed the site of labeling (N-terminus Gly) by MS (labeled G1-Q26, *m/z* 3027.51 [M+H]^+^) followed by MS-MS (Supplementary Fig. 49). Next, we selected a diagnostically relevant essential hemoprotein myoglobin **6b** [153 residues, one N^α^-NH_2_ (Gly), nineteen Lys N^ε^-NH_2_] and vortexed it with aldehyde **2e**. The mono-labeled myoglobin (**7b**, 40% conversion) is formed exclusively in this case (Fig. 3a). Peptide mapping of the enzymatic digest (labeled G1-K16, *m/z* 1996.37 [M+H]^+^) and MS-MS confirmed the site of labeling at N-terminus Gly (Supplementary Fig. 50). It was imperative to evaluate the chemistry with recombinant proteins. The engineering of Gly at the N-terminus of proteins is convenient^36^. In bacteria, the N-terminal Met is excised from proteins exposing the penultimate Gly with high efficiency^37^. Also, the recombinant proteins expressed with a recognition sequence for prescission protease renders the desired N-terminus Gly post proteolytic digestion^38^. We adopted the latter route and generated small Ubiquitin-like modifier (SUMO1) protein **6c** with a Gly residue at the N-terminus (see Supplementary information for details). The labeling of **6c** with **2e** went smoothly to result in N-terminus mono-labeled SUMO1 (**7c**, 43% conversion, Fig. 3a). Peptide mapping (labeled G1-L3, *m/z* 466.37 [M+H]^+^) and MS-MS confirmed the site of labeling at N-terminus Gly (Supplementary Fig. 51). Next, we selected insulin **6d** that contains two N-terminus [51 residues, two N^α^-NH_2_, chain A (Gly), chain B (Phe), one Lys N^ε^-NH_2_]. The imine formed by N^α^-NH_2_ (Phe) of chain B is established to exhibit the preferential reactivity^21–23^. However, the vortexing of insulin **6d** with the reagent **2e** results in the mono-labeled insulin **7d** in 71% conversion (Fig. 3a). The disulfide reduction of **7d**, peptide mapping, and MS-MS led to the identification of N^α^-NH_2_ (Gly) modification in chain A (labeled G1-N21, *m/z* 2563.00 [M+H]^+^, Supplementary Fig. 52). Next, we selected a mixture of seven proteins (**6d–6j**, Fig. 3b). Gratifyingly, the labeling experiment with reagent **2e** resulted in exclusive labeling of N-terminus Gly bearing insulin **6d** to render mono-labeled product **7d** (42% conversion, Fig. 3b and Supplementary Fig. 53). The methodology exhibits remarkable selectivity as none of the other residues in any protein interfere.

### Access to analytically pure tagged proteins

**Fig. 4:**
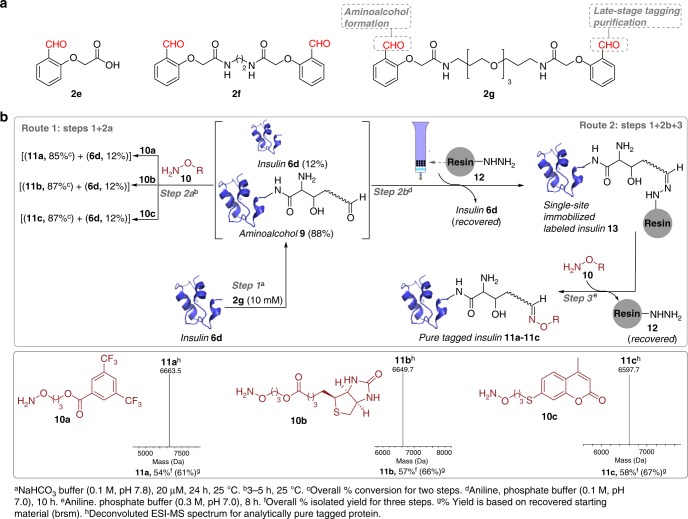
Late-stage tagging and isolation of analytically pure tagged proteins. **a** Design elements of a symmetric bis-aldehyde. **b** Insulin is labeled through aminoalcohol formation with reagent **2g**. The labeled insulin **9** can be treated with derivatives of O-hydroxylamine **10a**–**10c** for tagging them with ^19^F-NMR probe, biotin, and coumarin. Alternatively, it can be immobilized on hydrazide functionalized resin through single-site to deliver **13**. The unreacted insulin **6d** is recovered and recycled. Subsequent treatment of **13** with **10a** delivers analytically pure **11a** after removing excess **10a** through spin concentration. The hydrazide functionalized resin **12** is recovered and recycled multiple (5–8) times without loss of activity. Similar protocol renders pure **11b** and **11c**. For single-step late-stage installation of a probe, please see Supplementary Fig. 74

At this stage, understanding the impact of functional group derivatizations of **2e** on the reaction was essential for achieving the downstream tasks. We found that the amide linkage could be used while redesigning the reagent **2e** to a symmetric bis-aldehyde (**2f** and **2g**, Fig. 4a) with the handle for late-stage modification. Initially, the reaction of insulin with bis-aldehyde **2f** resulted in poor conversions. However, the incorporation of polyethylene glycol (PEG)_3_ linker in redesigned aldehyde **2g** regained the solubility of reagent and efficiency of transformation (88%, step 1, Fig. 4b; for the reaction of proteins **6a**–**6c** and *Pf*AOS1 **6k** with **2g**, see Supplementary Figs. 62–64). We did not observe the formation of insulin–insulin complex (Supplementary Table 3 and Fig. 65). Initially, we examined the efficiency of late-stage installation with benzyloxyamine. The product characterization involved disulfide reduction, peptide mapping, and MS-MS (Supplementary Figs. 54 and  55). Subsequently, we showcased the potential of this transformation with derivatives of O-hydroxylamine such as ^19^F NMR probe **10a**, biotin **10b**, and a fluorophore **10c** (step 2a, Fig. 4b). The excellent overall conversion (85%, 87%, and 87%, respectively) over the two steps (steps 1 and 2a; route 1) while retaining the selectivity is noteworthy (Supplementary Figs. 56–59). ^19^F NMR probe attached insulin **11a** shows a sharp NMR signal at −62.80 ppm (Fig. 4 and Supplementary Fig. 66). The incubated mixture of biotinylated insulin **11b** and streptavidin (Stv-n) renders a sharp band at ~70 kDa for Stv-**11b** complex (Supplementary Fig. 67). The coumarin attached insulin **11c** results in fluorescence emission at 428 nm (excitation at 333 nm, Supplementary Fig. 68).

Our next goal was to integrate a purification protocol with the aminoalcohol chemistry (steps 1, 2b, and 3; route 2; Fig. 4b). Initially, we mixed the reaction mixture of step 1 (**9** + **6d**) with hydrazide functionalized resin (step 2b, Fig. 4b). This step exhibits excellent efficiency and results in > 95% single-site immobilization of the labeled protein (**9**). The unreacted protein (**6d**) was recovered with > 95% efficiency and recycled. Finally, the immobilized protein (**13**) was released with O-hydroxylamine derivatives (**10a**–**10c**) from resin through transoximization (step 3, Fig. 4b). The centrifugal spin concentration results in the analytically pure single-site tagged protein (Supplementary Figs. 60 and  61). The single-site immobilized labeled insulin rendered efficient parallel installation of ^19^F NMR probe, biotin, and fluorophore (three steps, two purifications; 54%, 57%, and 58% overall isolated yields; Supplementary Figs. 60 and  61). The coumarin tagged insulin (**11c**) exhibits excellent product stability over a broad range of pH (3–11) at 25 °C for 36 h (Supplementary Fig. 76).

### Bioactivity assays

**Fig. 5:**
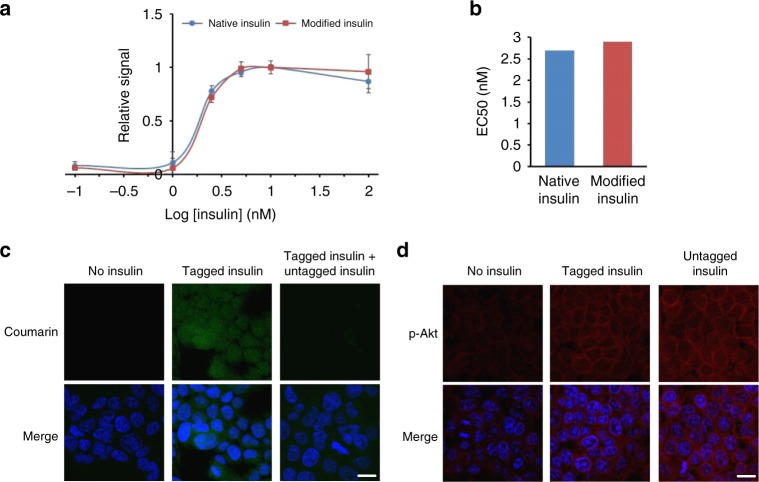
Insulin bioactivity assay. **a**, Bioactivity measurement for native and modified insulin in a dose-dependent pAkt cell-based assay. The error bar represents standard error of mean (SEM). The assays were repeated at least three times (*n* = 3). **b** EC50 value determination from (**a**), absolute values obtained by extrapolation of graphs in **a** are represented by a simple bar graph. **c** Uptake of tagged insulin (green) and mixture of untagged and tagged insulin in cells. Chromatin (blue). Also see, Supplementary Fig. 77. **d** Activation of IR signaling and pAkt (red) accumulation in HEK293T cells after insulin treatment (scale bar:10 µm). Source data of Fig. 5 panel **a** provided as Source Data file

Subsequently, we asked how the labeling would affect the structure and activity of insulin. The earlier investigations suggest that N^α^-NH_2_ (Gly) of chain A is important for insulin structure and function^39,40^. The circular dichroism of coumarin tagged insulin (**11c**) confirmed the conservation of structure (Supplementary Fig. 69). We examined the activity in cell-based assays using **11c** through its ability to activate insulin receptor (IR) mediated signaling. Activation of insulin-dependent IR-signaling was assessed by the increase in levels of phospho-Akt (pSer-473) and subsequent uptake of insulin inside the cells. The prior is detected by phospho-Akt (pAkt) specific antibodies on western blotting and immunofluorescence. The latter is assessed through the coumarin fluorophore signal inside cells. While the untreated (mock) cells exhibit basal pAkt reactivity, cells treated with **11c** or untagged insulin (**6d**) render enhanced phospho-Akt (pAkt) reactivity relative to loading control protein (GAPDH) (Supplementary Fig. 78a). The quantitation of pAkt signals relative to loading control GAPDH is represented in Supplementary Fig. 78b. These observations directed us towards the estimation of EC50 values for native and modified insulin^41^. Both native and modified insulin activated the insulin receptor and pAkt generation with similar efficacy (Fig. 5a). The EC50 values were estimated to be 2.7 and 2.9 nM for native (**6d**) and modified insulin (**S29**), respectively (Fig. 5b). In the cellular uptake assays, we observed that cells treated with **11c** display coumarin signal accumulated throughout the cells indicating their regular uptake (Fig. 5c, 1st row, middle panel). This signal can be competed out in the presence of excess untagged insulin (**11c**:**6d**, 1:3; Fig. 5c, 1st row, right panel). Concomitant to this, we observe a similar increase in pAkt signals (pSer-473) in tagged or untagged insulin-treated cells (Fig. 5d, 1^st^ row middle and right panels). These observations unambiguously establish that aminoalcohol derivatization of N-Gly in insulin results in no adverse effect on its capability to activate the insulin receptor signaling. Next, we investigated the SUMOylation reaction that provides a versatile tool to identify and characterize novel SUMO enzymes and their substrates^42^. We reconstituted the SUMOylation reaction with modified SUMO1 and monitored the SUMO1-substrate conjugate. The data concludes that the N-Gly modification of SUMO1 does not interfere with its biochemical activity (for details, see Supplementary Fig. 79).

### Residue-specific labeling of a protein in cell lysate

**Fig. 6:**
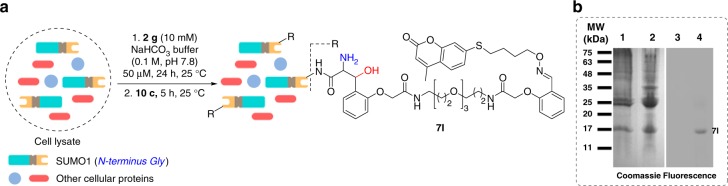
Residue-specific labeling of a protein in the cell lysate. **a** Selective labeling of N-Gly SUMO1 with coumarin tag in cell lysate. **b** 12% SDS-PAGE of cell lysate (overexpressed with SUMO1) and coumarin-tagged SUMO1 **7l** in cell lysate followed by coomassie staining and fluorescence imaging. MW—molecular weight, Lanes 1 and 2: cell lysate before and after reaction (Coomassie); lanes 3 and 4: cell lysate before and after reaction (fluorescence). The band at ~17 kDa (*m/z* 11.6 kDa) in lanes corresponds to the SUMO1. The band at ~17 kDa (*m/z* 11.6 kDa) shown by fluorescence imaging in lane 4 confirms the selective tagging of SUMO1 (**7l**). Source data of Fig. 6 panel **b** provided as Source Data file

Finally, we challenged the methodology to precisely label the N-terminus Gly containing protein in a cell lysate prepared from *E. coli* BL21 (DE3) cells overexpressing GST-SUMO1. Proteolytic digest generates N-terminal Gly containing SUMO1 in a mixture of multiple other proteins present in the lysate sample (lanes 1 and 2, Fig. 6). Such a lysate was vortexed with the symmetrical bis-aldehyde **2g**. The late-stage installation of coumarin on the N-terminal Gly residue is visualized by SDS-PAGE and fluorescence imaging. The labeling of SUMO1 is observed without noticeable participation of any other protein (lane 4, Fig. 6).

## Discussion

In summary, we deliver a methodology that enables single-site residue-specific labeling of a protein. We demonstrated that a systematic multi-step approach could provide the platform to solve the multifaceted selectivity challenges. The chemoselective generation of an electrophilic intermediate followed by site- and residue-specific formation of a latent nucleophile is the key to success. The reaction proceeds exclusively with the N-terminus Gly residue of a protein. The selectivity remains uncompromised even when the method is extended to a protein in the cell lysate. The reaction excludes the N-terminus α-amine of other proteinogenic amino acids and multiple Lys residues bearing ε-amine. The absence of competing reactions is one of the most noteworthy features of this chemical technology. The symmetric bis-aldehyde reagent allows installation of various tags on single-site labeled protein. A user-friendly purification protocol enables the isolation of analytically pure tagged protein. The overall yield of the product after three steps is remarkable. The protocol offers convenient imaging of insulin in HEK293T cells without perturbing its structure and activity.

## Methods

### Single-site labeling of a protein

In a 1.5 mL microcentrifuge tube, protein (3 nmol) was mixed with sodium bicarbonate buffer (120 µl, 0.1 M, pH 7.8). To this solution, 2-(2-formylphenoxy) acetic acid **2e** (1500 nmol) in DMSO (30 µl) from a freshly prepared stock solution was added and vortexed at 25 °C. The overall concentration of protein and 2-(2-formylphenoxy)acetic acid was 20 µM and 10 mM respectively. After 24–48 h, the reaction mixture was diluted with acetonitrile:water (10:90, 3000 µl). Unreacted 2-(2-formylphenoxy)acetic acid and salts were removed by using Amicon® Ultra-0.5 mL 3-kDa or 10-kDa MWCO centrifugal spin concentrator. The protein mixture was further washed with Millipore Grade I water (5 × 0.4 mL). The sample was analyzed by ESI-MS or MALDI-ToF-MS. The aqueous sample was concentrated by lyophilization before subjecting it to digestion, peptide mapping, and sequencing by MS-MS.

### Late-stage tagging and purification of a protein

Hydrazide beads (200 µl, hydrazide resin loading: 16 µmol mL^−1^) were taken in a 5 mL fritted polypropylene chromatography column with end tip closures. Phosphate buffer (0.1 M, pH 7.0, 5 × 1 mL) was used to wash the beads. The beads were re-suspended in phosphate buffer (100 µl, 0.1 M, pH 7.0). Protein mixture (250 µM) in phosphate buffer (150 µl, 0.1 M, pH 7.0) and aniline (100 mM) in phosphate buffer (100 µl, 0.1 M, pH 7.0) were added to the beads followed by end-to-end rotation (30 rpm, rotary mixer) at 25 °C. The progress of the immobilization of the labeled protein on hydrazide resin was monitored by UV-absorbance of the supernatant. After 8–10 h, the supernatant was collected and the beads were washed with phosphate buffer (0.3 M, pH 7.3, 4 × 1 mL) and KCl (1 M, 3 × 1 mL) to remove the adsorbed protein from resin. The beads were further washed with the phosphate buffer (0.3 M, pH 7.0, 4 × 1 mL) and re-suspended (phosphate buffer, 200 µl, 0.3 M, pH 7.0). To release the labeled protein from its immobilized derivative, aniline (100 mM) in phosphate buffer (100 µl, 0.3 M, pH 7.0) and coumarin or fluoro or biotin derivatives (only one at a time) of O-hydroxylamine (50 µl, 150 mM in DMSO) were added followed by vortex at 25 °C for 6–8 h. The supernatant was collected while the salts, aniline, and O-hydroxylamine were removed using the spin concentrator (3 kDa MWCO). The purity of the labeled protein was confirmed by ESI-MS. The purification protocol renders analytically pure tagged protein.

### Residue-specific labeling of a protein in cell lysate

The cell lysate was prepared from *E. coli* BL21 (DE3) cells overexpressing GST-SUMO1 (section 2d in Supplementary information). Protein concentration was determined by BCA assay and adjusted to 1.0 mg mL^−1^. Protein (10 nmol) in sodium bicarbonate buffer (120 µl, 0.1 M, pH 7.8) was taken in a 1.5 mL microcentrifuge tube. To this solution, N,N’-(((oxybis(ethane-2,1-diyl))bis(oxy))bis(propane-3,1-diyl))bis(2-(2-formylphenoxy)acetamide) **2g** (5000 nmol) in DMSO (30 µl) from a freshly prepared stock solution was added and vortexed at 25 °C. After 24 h, the reaction mixture was diluted with acetonitrile:buffer (10:90, 1500 µl). Unreacted N,N’-(((oxybis(ethane-2,1-diyl))bis(oxy))bis(propane-3,1-diyl))bis(2-(2-formylphenoxy)acetamide) **2g** and salts were removed by spin concentrator (0.5 mL 3-kDa MWCO). The solution was further washed with sodium bicarbonate buffer (0.1 M, pH 7.8) and concentrated to 160 µl. To the concentrated sample in sodium bicarbonate buffer, derivatives of O-hydroxylamine 7-((3-(aminooxy)propyl)thio)-4-methyl-2H-chromen-2-one (2 µmol) **10c** in DMSO (40 µl) from a freshly prepared stock solution was added to convert mono-labeled protein to its oxime derivative for 3–6 h. The excess of O-alkoxyamine and salts were removed by the spin concentrator. The sample was analyzed by SDS-PAGE.

### Biological assays

*Imaging assays*: The HEK293T (Cell Repository, NCCS, Pune India) cells were grown in a six-well plate with coverslips in Dulbecco’s modified Eagle’s medium (DMEM) containing 1% serum for 24 h. Subsequently, the cells were washed twice with PBS and treated with coumarin tagged **11c** and untagged insulin **6d** (3 µg mL^−1^, 0.5 µM) for 30 min in 10% FBS containing DMEM media. For competition experiment, tagged and untagged insulin were used in 1:3 ratios in final 2.0 µM insulin concentration. Post-treatment, cells were again washed twice with PBS and fixed using 100% chilled methanol for 15 min at −20 °C. The cells were then rehydrated and permeabilized with rehydration buffer (10 mM Tris, 150 mM NaCl, 0.1% TritonX-100) for 10 min. For coumarin tagged insulin imaging, nuclei were stained with Hoechst 33342 (Invitrogen) directly after permeabilization and images were taken. For pAkt imaging, cells were blocked with 5% Normal Goat Serum (NGS) for 30 min at room temperature after rehydration. The cells were stained overnight with pAkt antibodies (1:200, Cell Signaling Technology) at 4 °C. After primary antibody incubation, cells were washed three times with PBS-T (5 min each). Alexa Fluor-568 conjugated goat anti-rabbit IgG (1:800, Life Technologies) secondary antibody was used against pAkt. After this, the nuclei were stained with Hoechst 33342 (Invitrogen), and fluorescence images were captured on APOTOME/Zeiss LSM 780 confocal microscope. All image analysis was performed using ZEN (Zeiss) or Image J software.

### EC50 analysis

For the assay, 50,000 human embryonic kidney cells (HEK293T) were grown in each well of a 96- well plate to ~50% confluence in DMEM supplemented with 10% fetal bovine serum (FBS) and antibiotics in a humidified incubator with 5% CO_2_ at 37 °C. The cells were serum starved for 12 h in no serum DMEM media and then treated with varying concentrations (100, 10, 5, 2.5, 1, 0.5, 0.1 nM) of insulin and modified insulin in no serum DMEM media for 40 min at 37 °C. After the treatment, the insulin-containing media was removed, cells were washed and intracellular level of pAkt Ser473 was measured using HTRF pAkt Ser473 kit (Cisbio, France, Catalogue No. 64AKSPET and Perkin Elmer, Catalogue No. TRF4002C) following the manufacturer's protocol. In brief, cells were lysed in 50 µl of supplemented lysis buffer under mild horizontal shaking at room temperature for 45 min. The homogenized cell lysate (16 µl) was added to 4 µl of the premixed antibody solutions in the HTRF 96-well detection plate. After 4 h of incubation at room temperature, the absorbance was recorded in a Cytation 5 multi-mode reader (BioTek Instrument, USA).

### Reporting summary

Further information on research design is available in the Nature Research Reporting Summary linked to this article.

## Supplementary information

Supplementary Information

Reporting Summary

Source Data

## Data Availability

All data supporting the findings of this study are available within the Article and its accompanying Supplementary Information file. The source data underlying Figs. 5a, 6b and Supplementary Figures 63b, 67, 68, 69, 72, 78a, 79c is provided in the Source Data file. Raw data for all the NMR spectra and HPLC-MS/ESI-MS/MALDI-ToF-MS can be accessed from the corresponding author upon reasonable request.
