# Supplementary material for: Single-site glycine-specific labeling of proteins
Source: Nat Commun. 2019 Jun 10;10:2539. doi: 10.1038/s41467-019-10503-7 (PMC6557831; doi:10.1038/s41467-019-10503-7)
Supplement: Supplementary file 1 — Supplementary Information [file 41467_2019_10503_MOESM1_ESM.pdf]

Single-site glycine-specific labeling of proteins

Rai et al.

Supplementary Information

Supplementary Figures

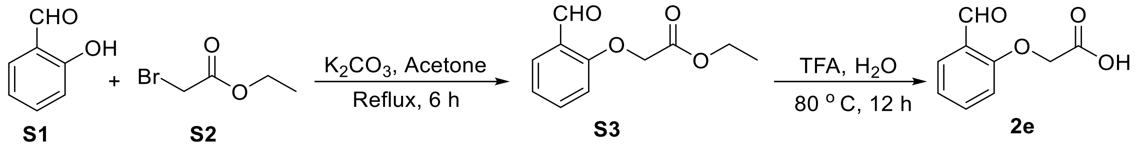

Supplementary Figure 1. Synthesis of 2-(2-formylphenoxy) acetic acid **2e**[3]

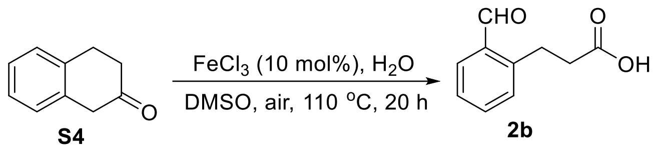

Supplementary Figure 2. Synthesis of 3-(2-formylphenyl)propanoic acid **2b**[4]

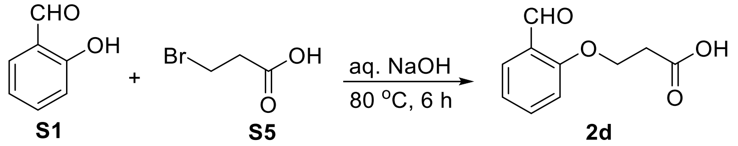

Supplementary Figure 3. Synthesis of 3-(2-formylphenoxy)propanoic acid **2d**

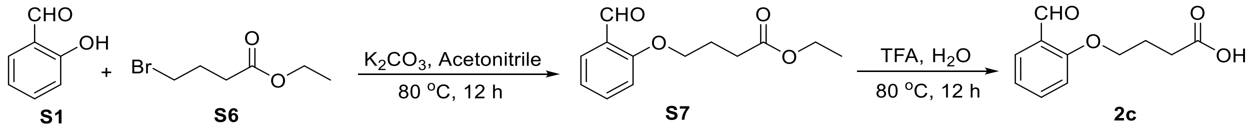

Supplementary Figure 4. Synthesis of 4-(2-formylphenoxy)butanoic acid **2c**

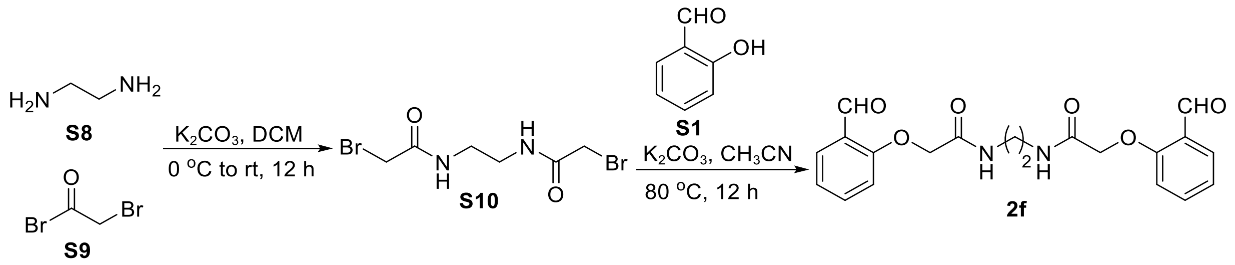

Supplementary Figure 5. Synthesis of N,N'-(ethane-1,2-diyl)bis(2-(2-formylphenoxy)acetamide) **2f**

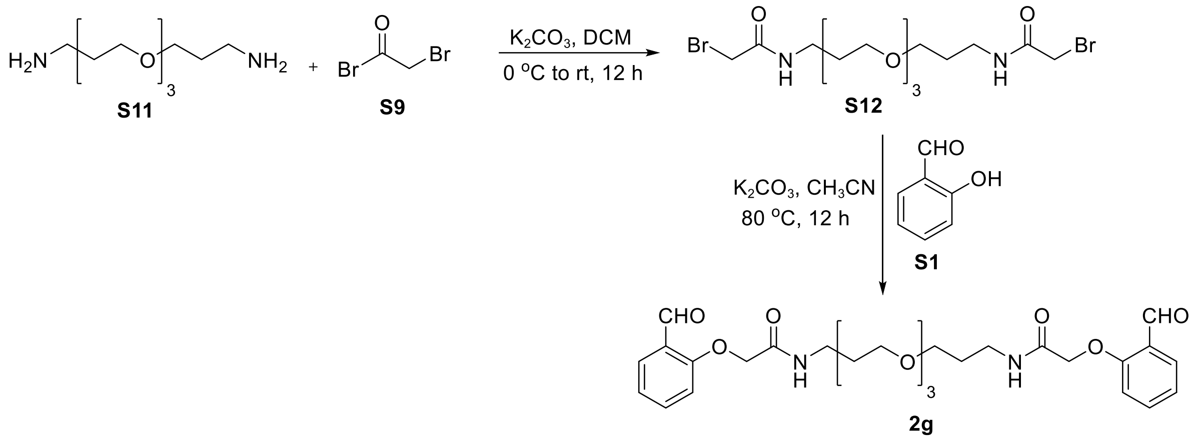

Supplementary Figure 6. Synthesis of compound **2g**

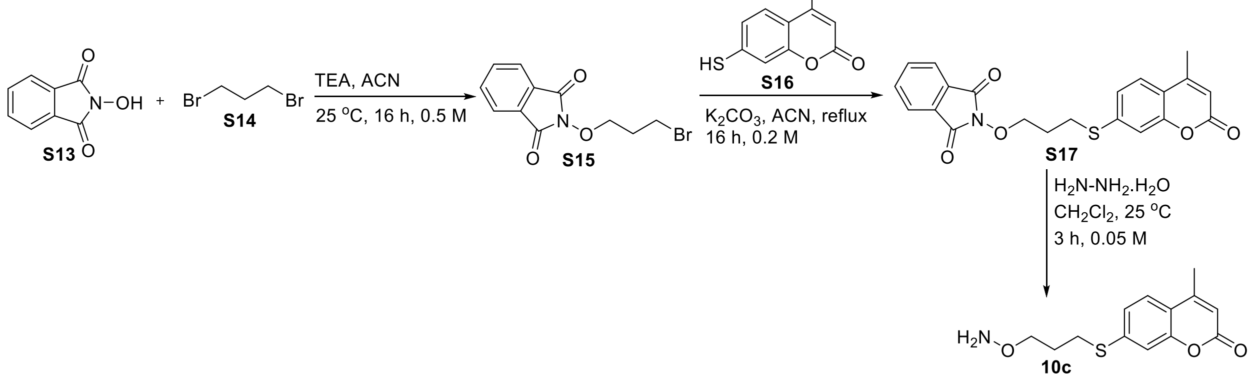

Supplementary Figure 7. Synthesis of 7-((3-(aminooxy)propyl)thio)-4-methyl-2H-chromen-2-one **10c**

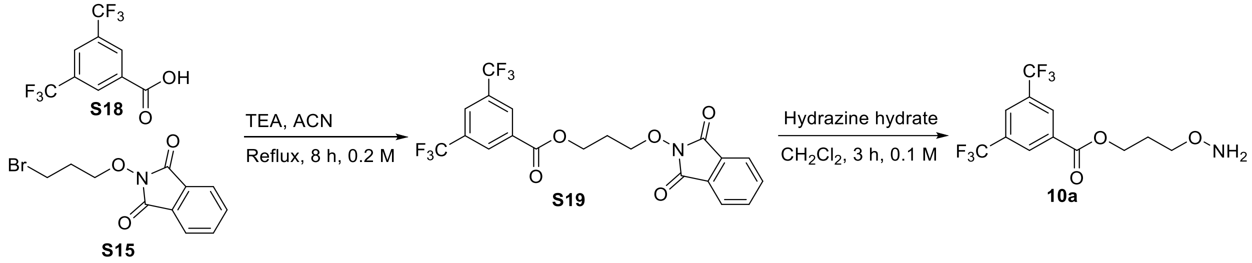

Supplementary Figure 8. Synthesis of 3-(aminooxy)propyl 3,5-bis(trifluoromethyl)benzoate **10a**

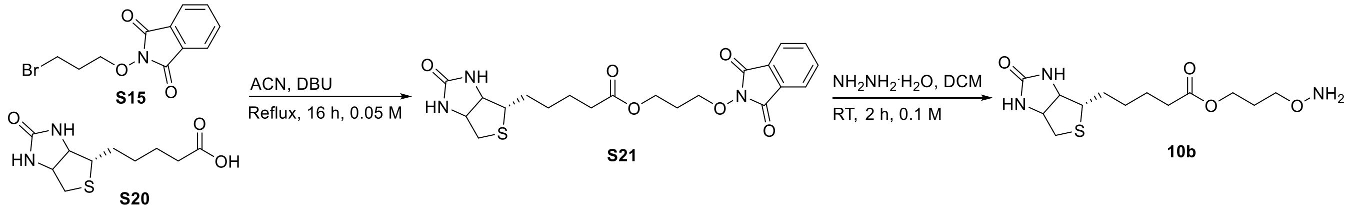

Supplementary Figure 9. Synthesis of compound **10b**

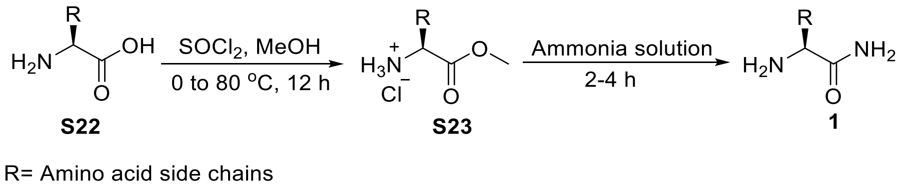

Supplementary Figure 10. Synthesis of amino acid amides **1**[7]

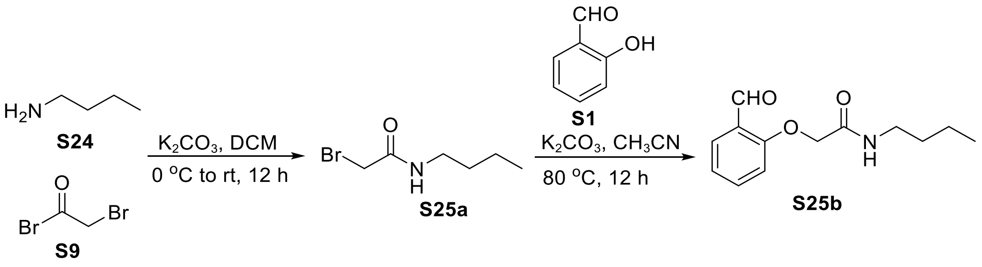

Supplementary Figure 11. Synthesis of N-butyl-2-(2-formylphenoxy)acetamide **S25b**

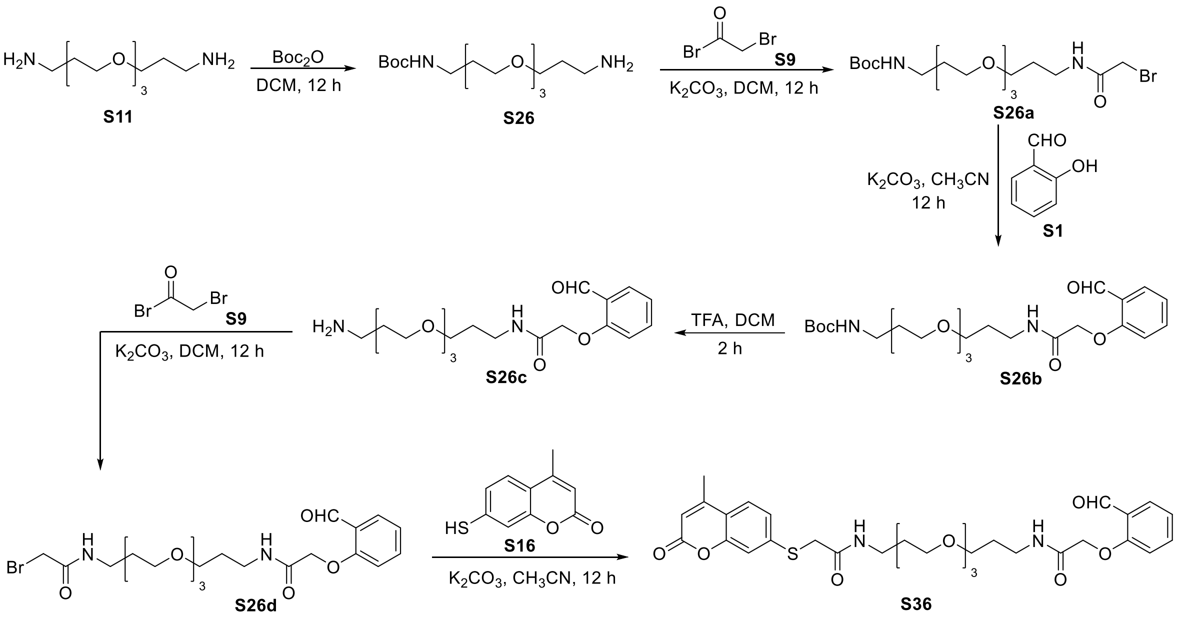

Supplementary Figure 12. Synthesis of compound **S36**

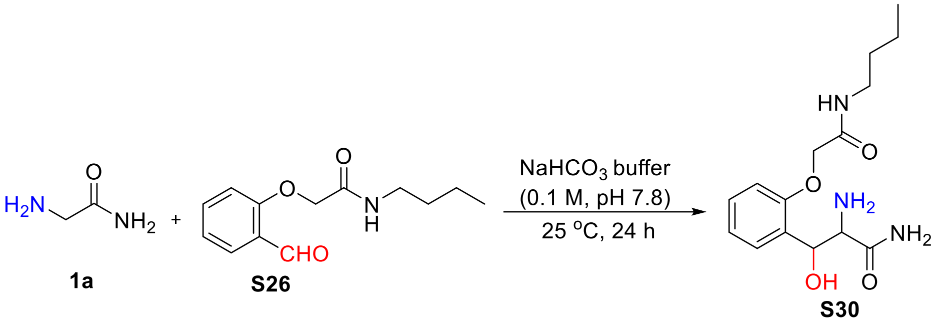

Supplementary Figure 13. Synthesis of compound **S30**

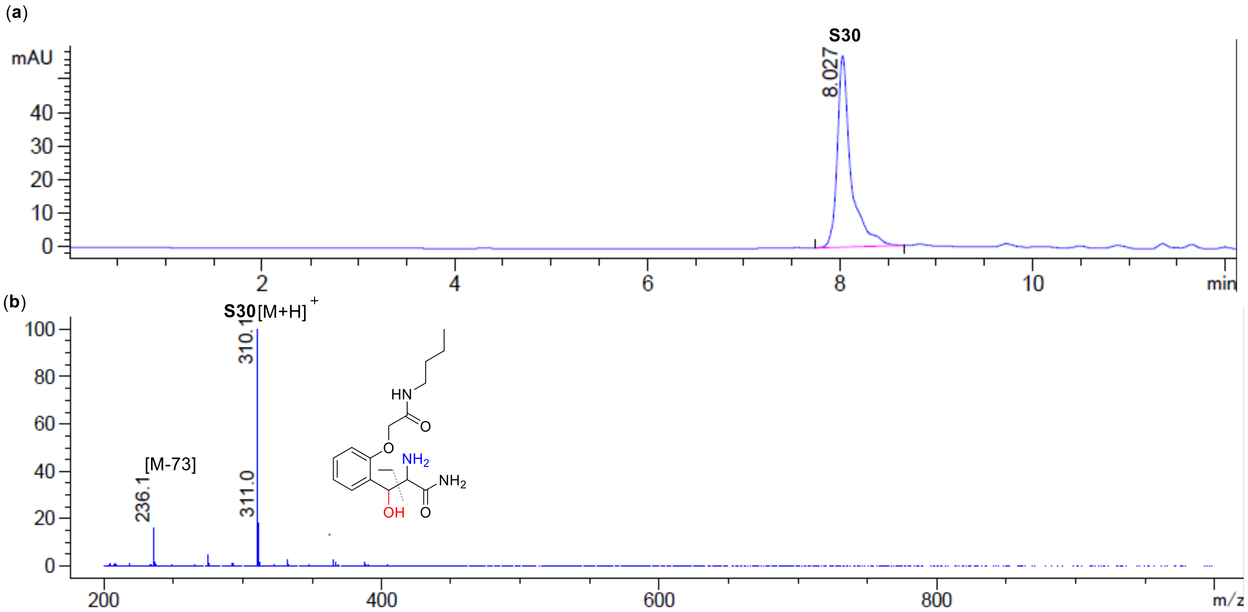

Supplementary Figure 14. LC and ESI-MS spectrum of compound **S30**

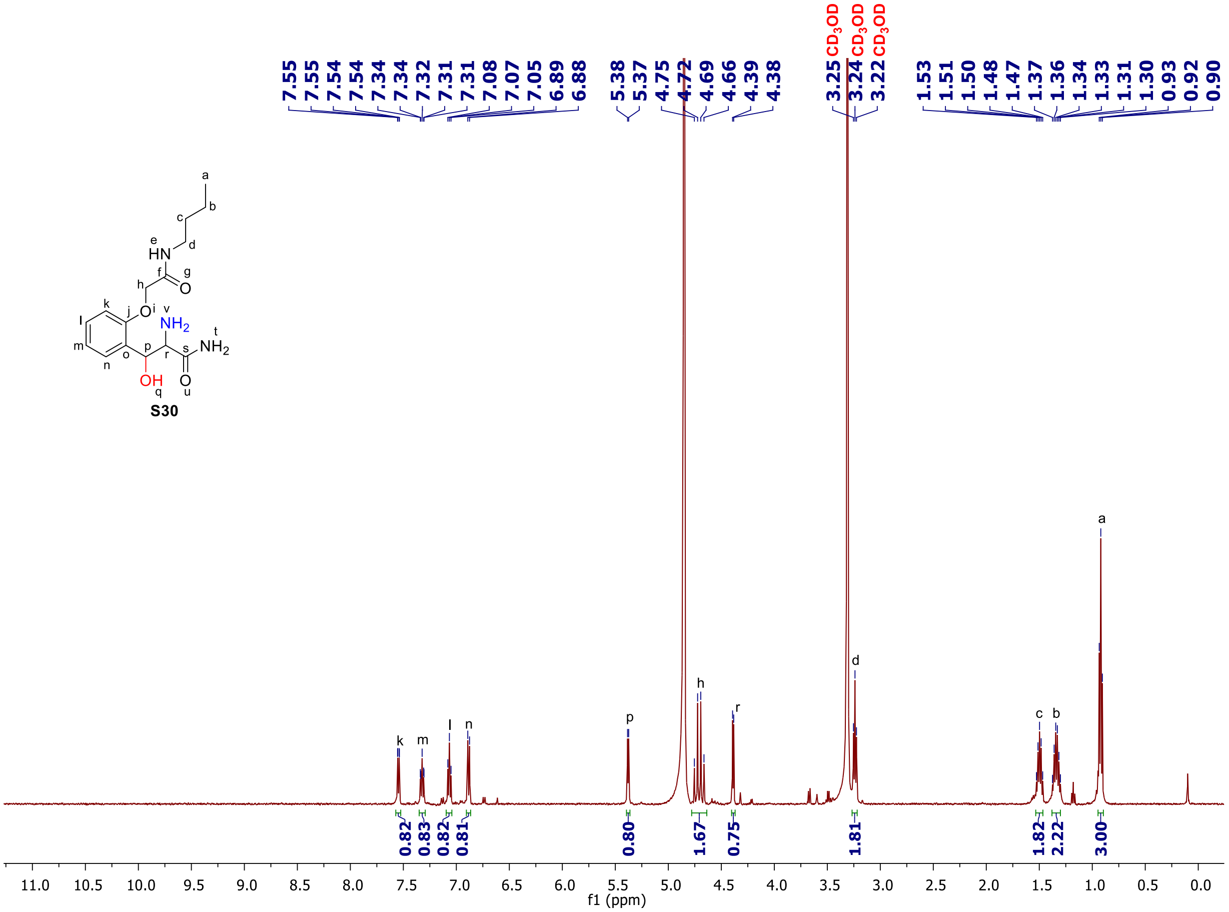

S30

Supplementary Figure 15. ^{1}H NMR spectrum in CD_3OD of compound **S30**

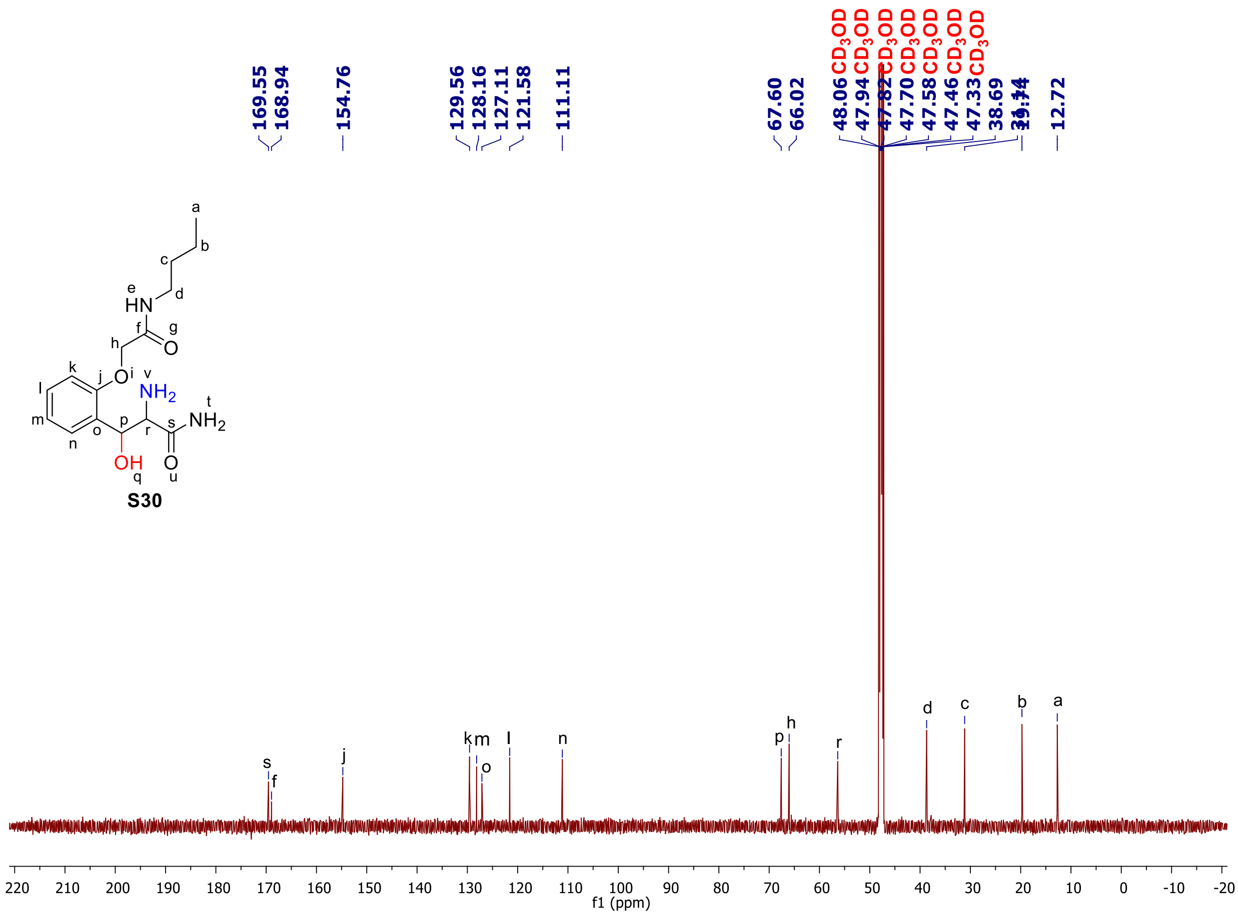

Supplementary Figure 16. ^{13}C NMR spectrum in CD_3OD of compound **S30**

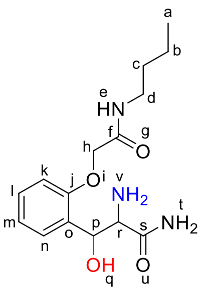

S30

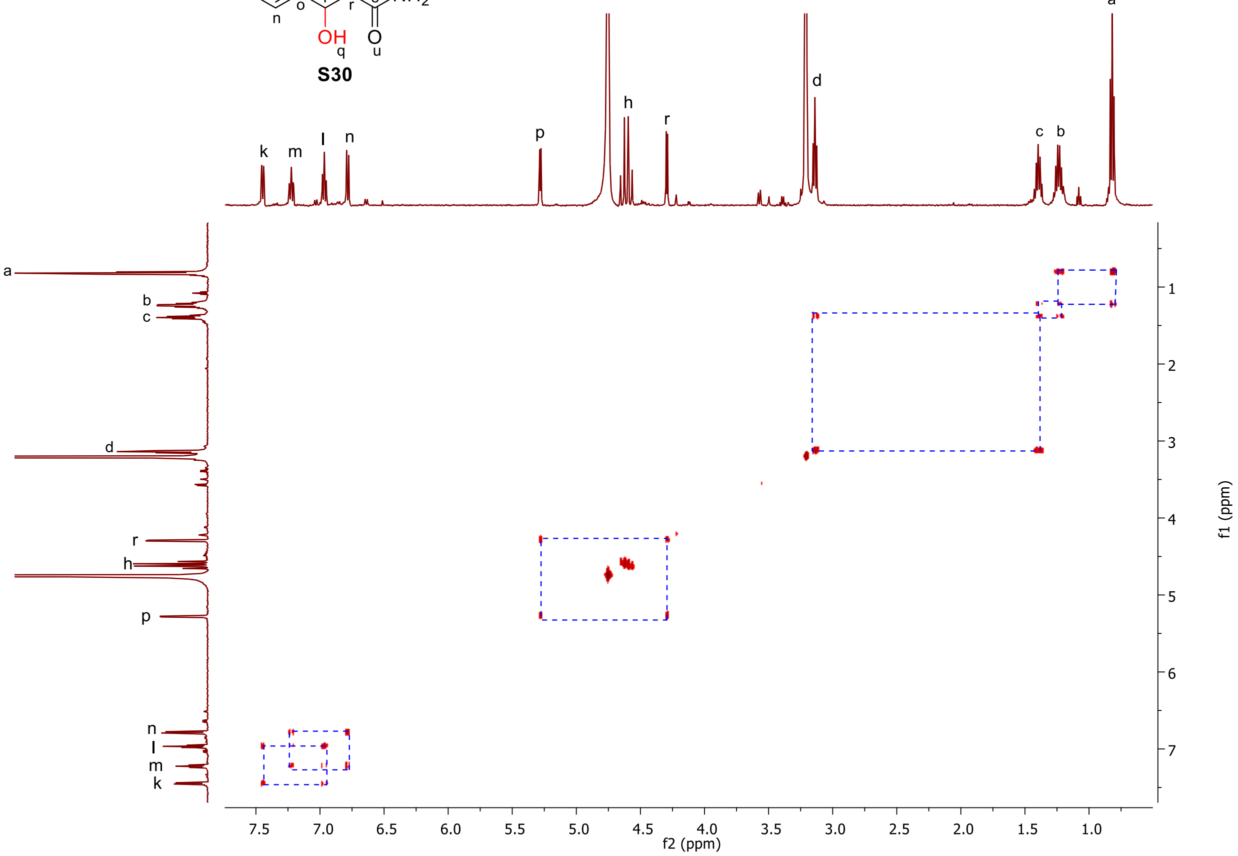

^{1}H-^{1}H correlation (COSY)

**^{1}H-^{1}H**	H_a	H_c	H_l	H_n	H_r
H_b	√	√			
H_d		√			
H_k			√		
H_m				√	
H_p					√

Supplementary Figure 17. ^{1}H-^{1}H correlation (COSY) spectrum in CD_3OD of compound **S30**

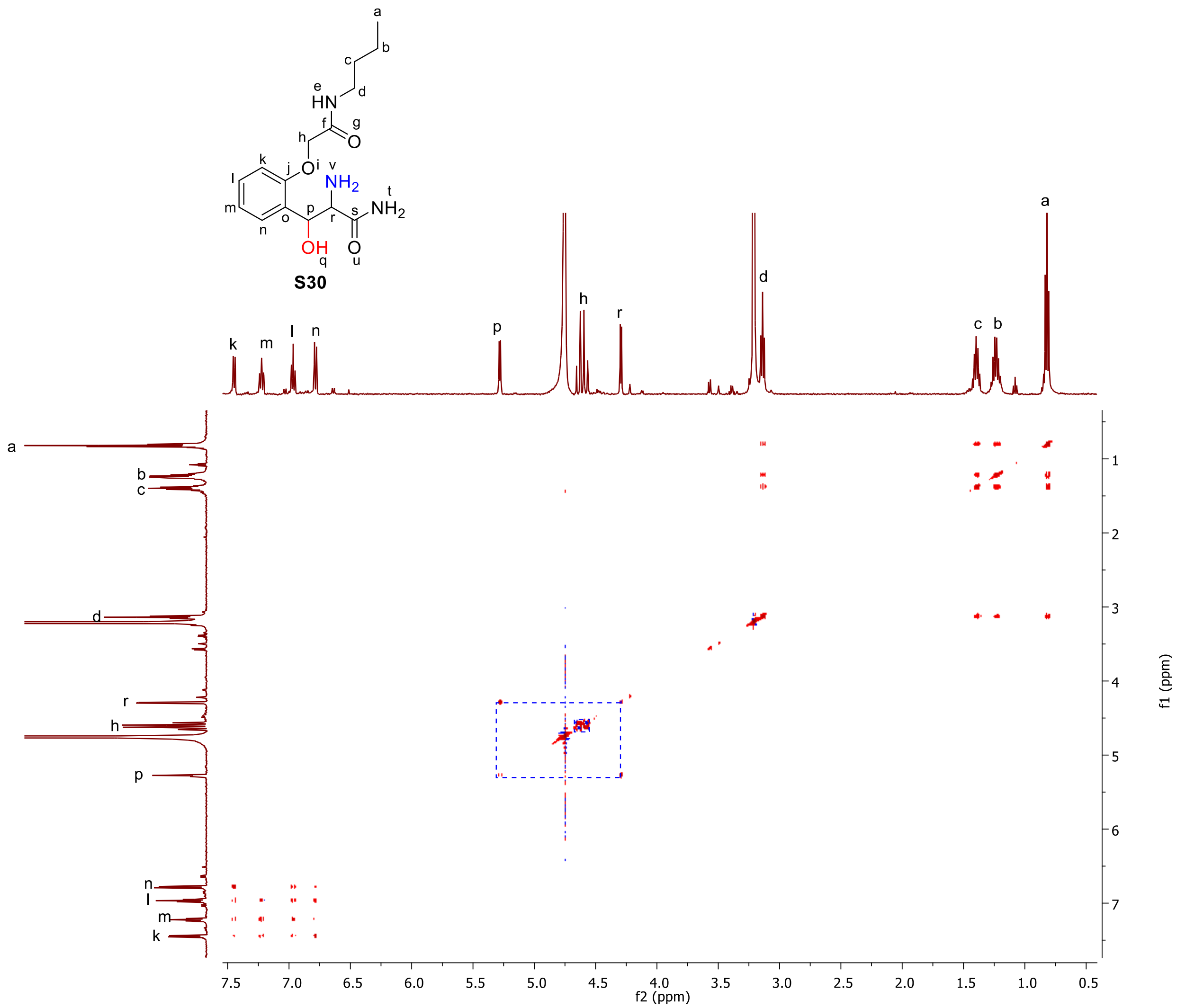

^{1}H-^{1}H correlation (TOCSY correlation other than COSY)

**^{1}H-^{1}H**	H_{h2}	H_r
H_{h1}	√	
H_p		√

Supplementary Figure 18. ^{1}H-^{1}H correlation (TOCSY) spectrum in CD_3OD of compound **S30**

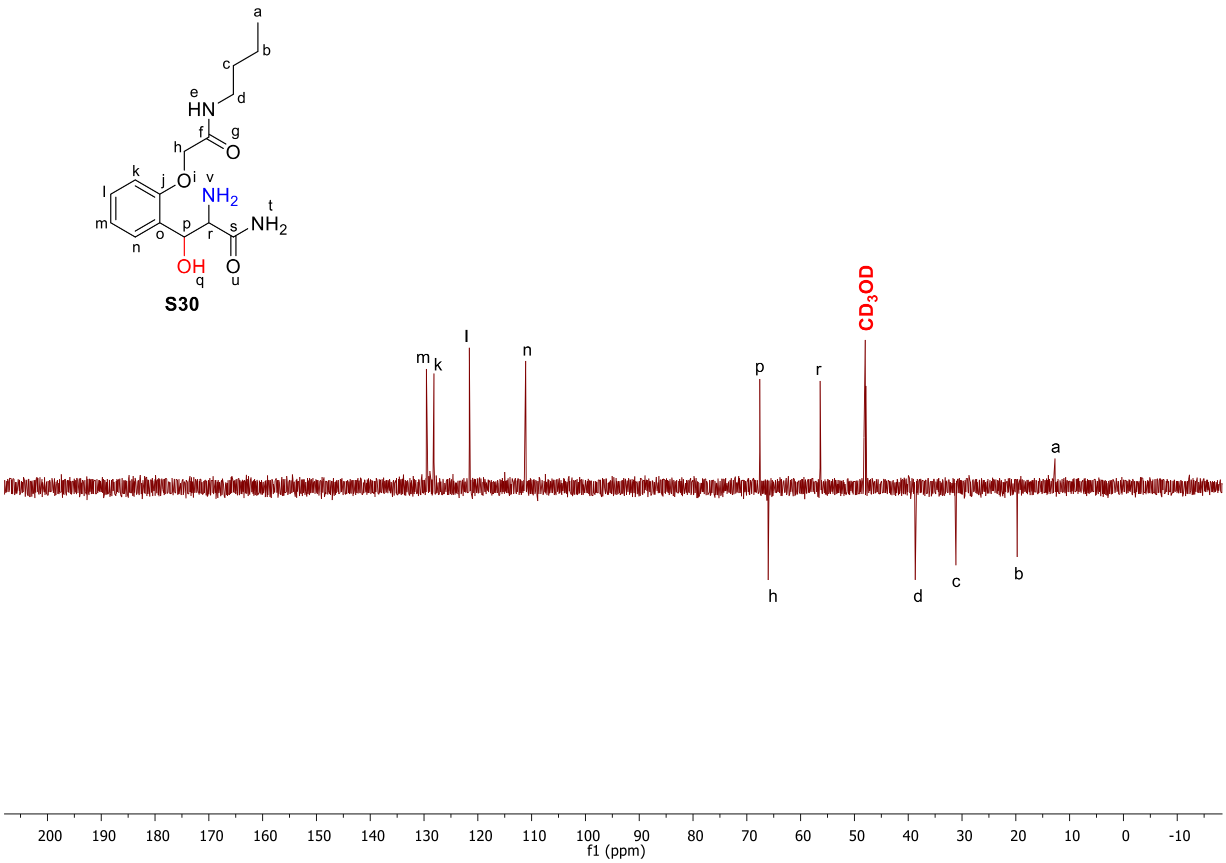

Supplementary Figure 19. DEPT-135 spectrum in CD_3OD of compound **S30**

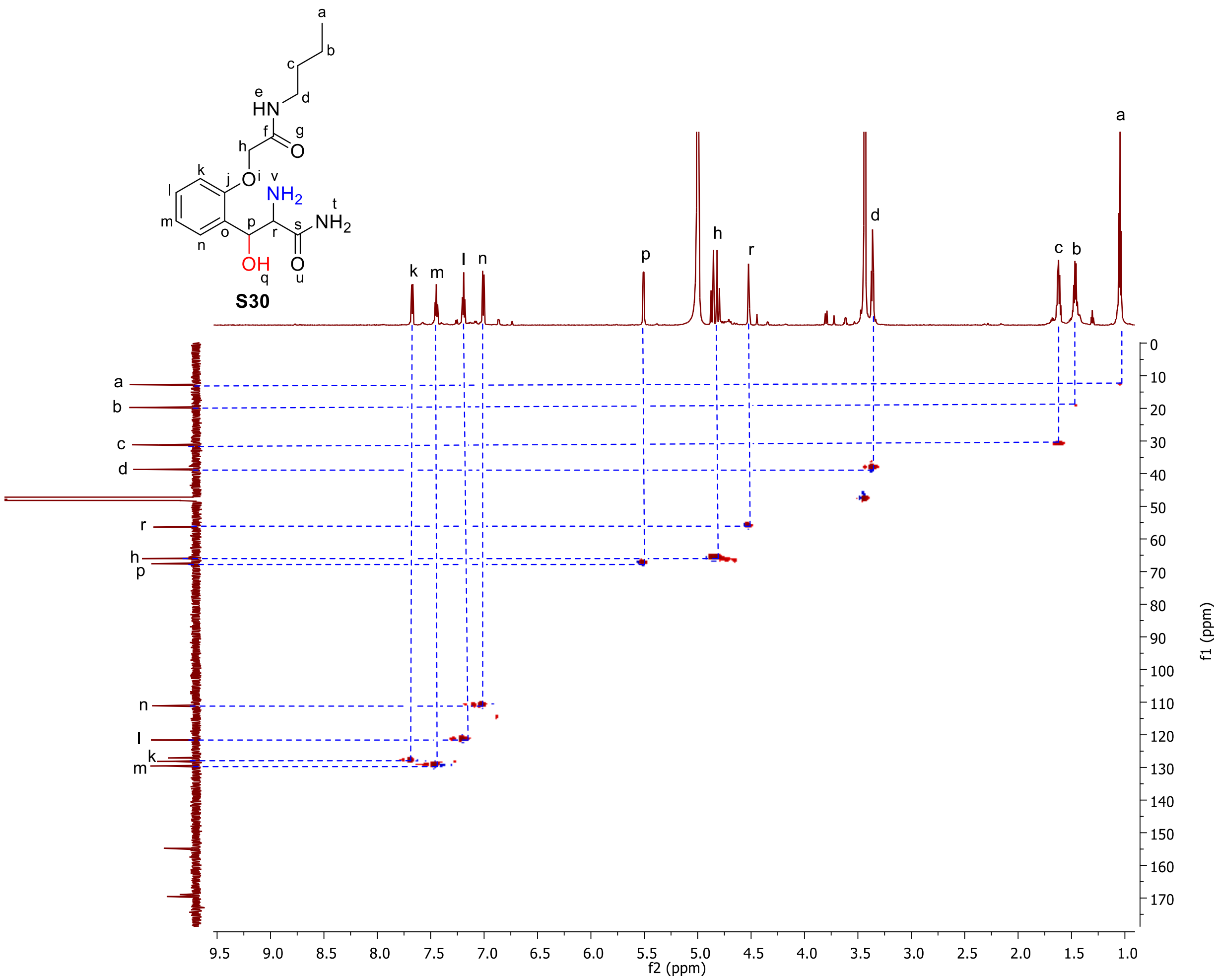

Supplementary Figure 20. ^{1}H-^{13}C correlation (HSQC) spectrum in CD_3OD of compound **S30**

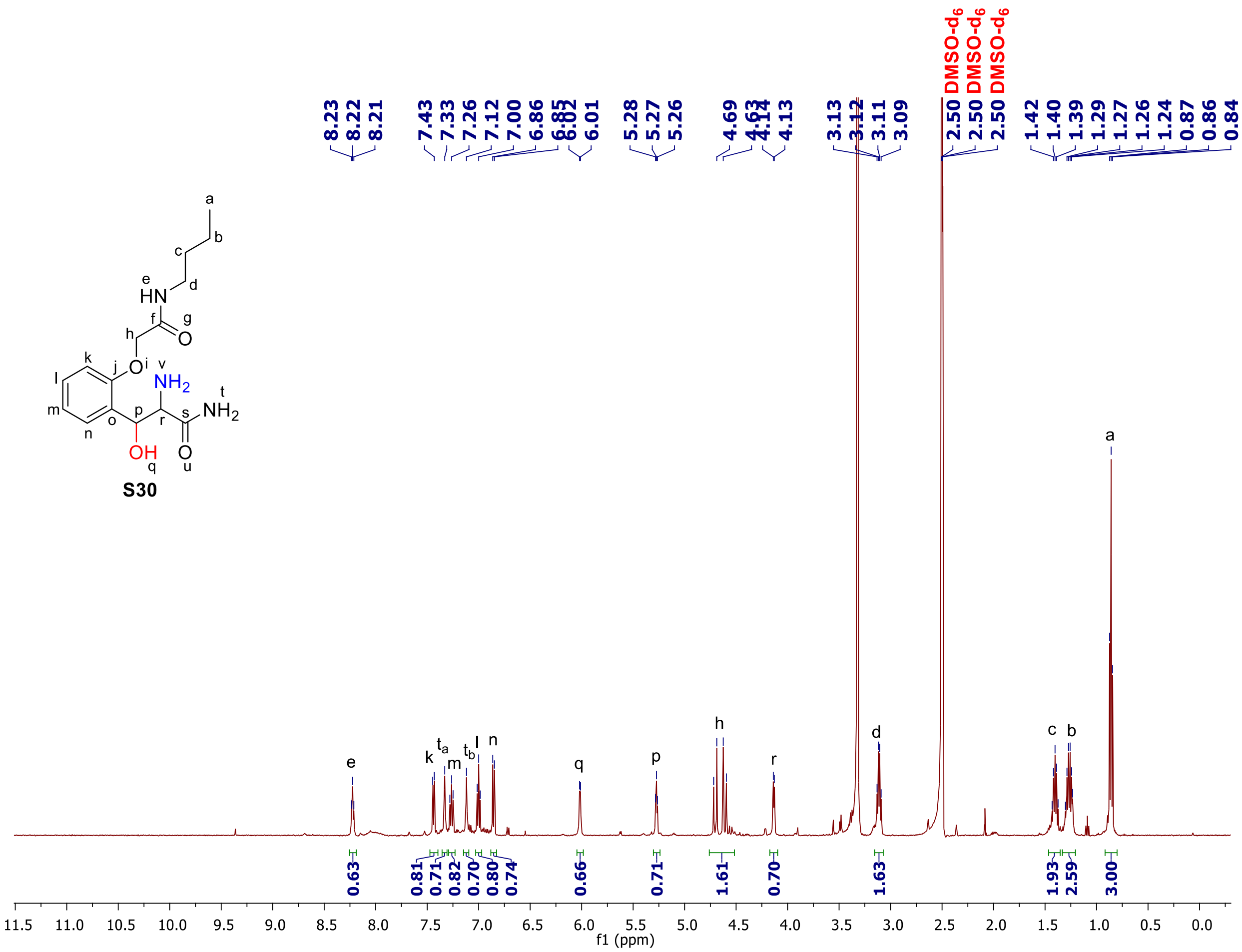

Supplementary Figure 21. ^{1}H NMR spectrum in DMSO-d_6 of compound **S30**

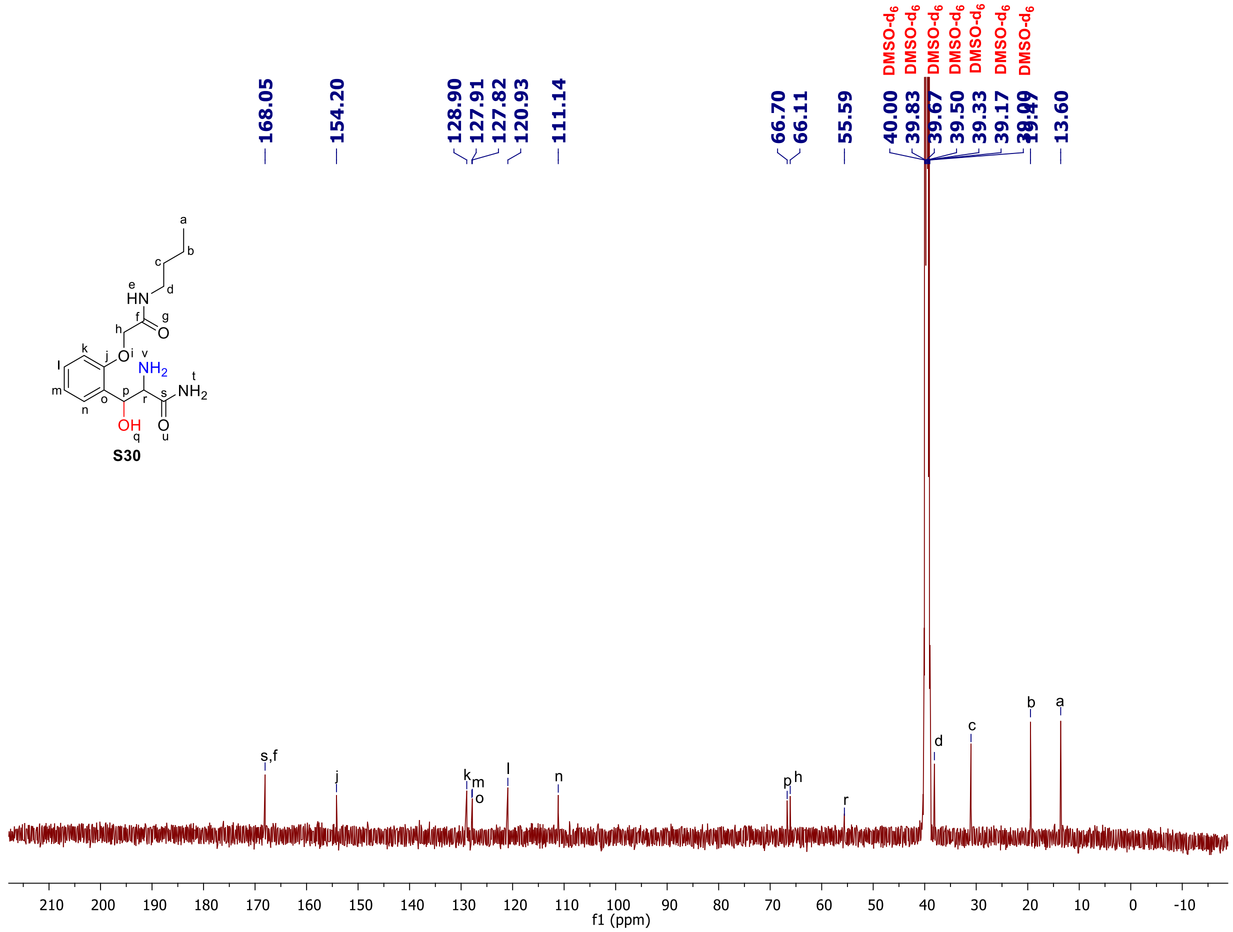

Supplementary Figure 22. ^{13}C NMR spectrum in DMSO-d$_6$ of compound **S30**

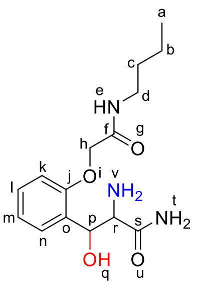

S30

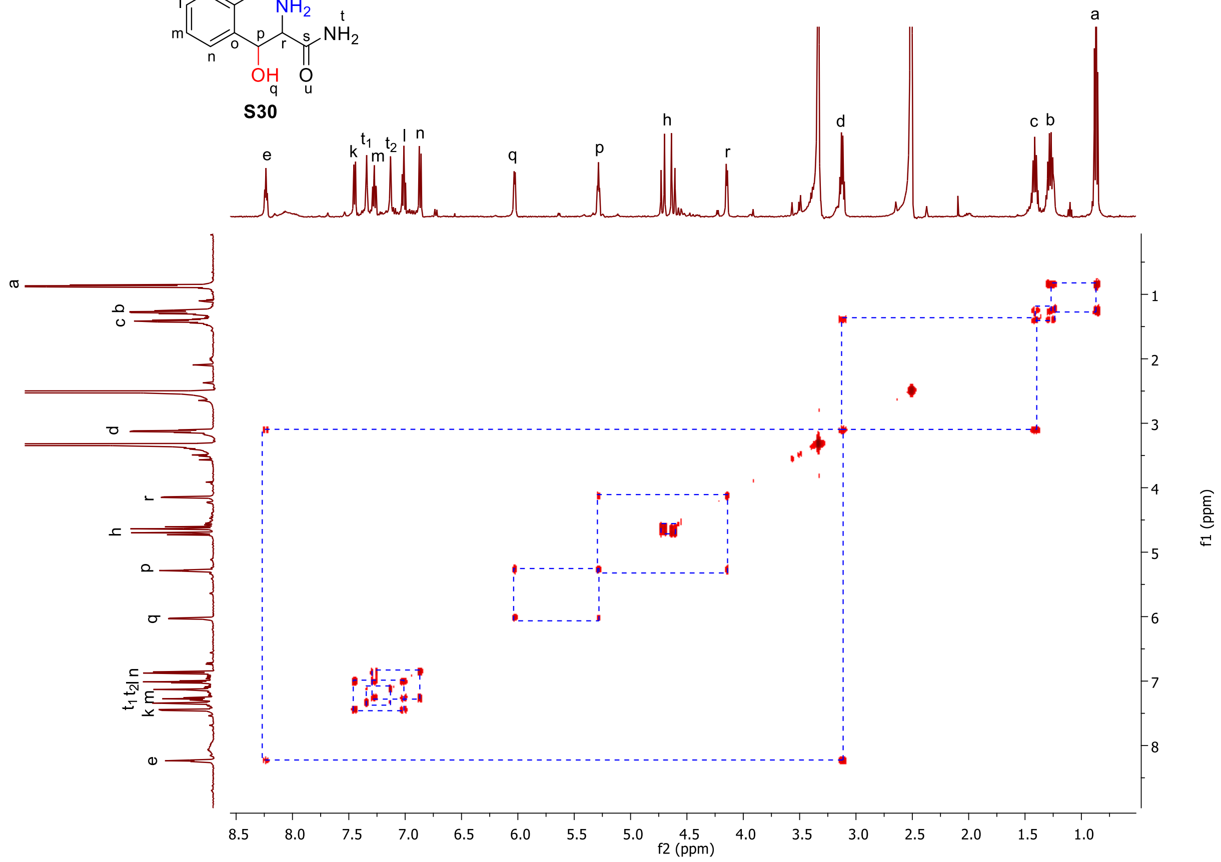

^{1}H-^{1}H correlation (COSY)

**^{1}H-^{1}H**	H_a	H_c	H_e	H_{h2}	H_l	H_q	H_n	H_r
H_b	√	√						
H_d		√	√					
H_{h1}				√				
H_k					√			
H_m							√	
H_p						√		√

Supplementary Figure 23. ^{1}H-^{1}H correlation (COSY) spectrum in DMSO-d_6 of compound **S30**

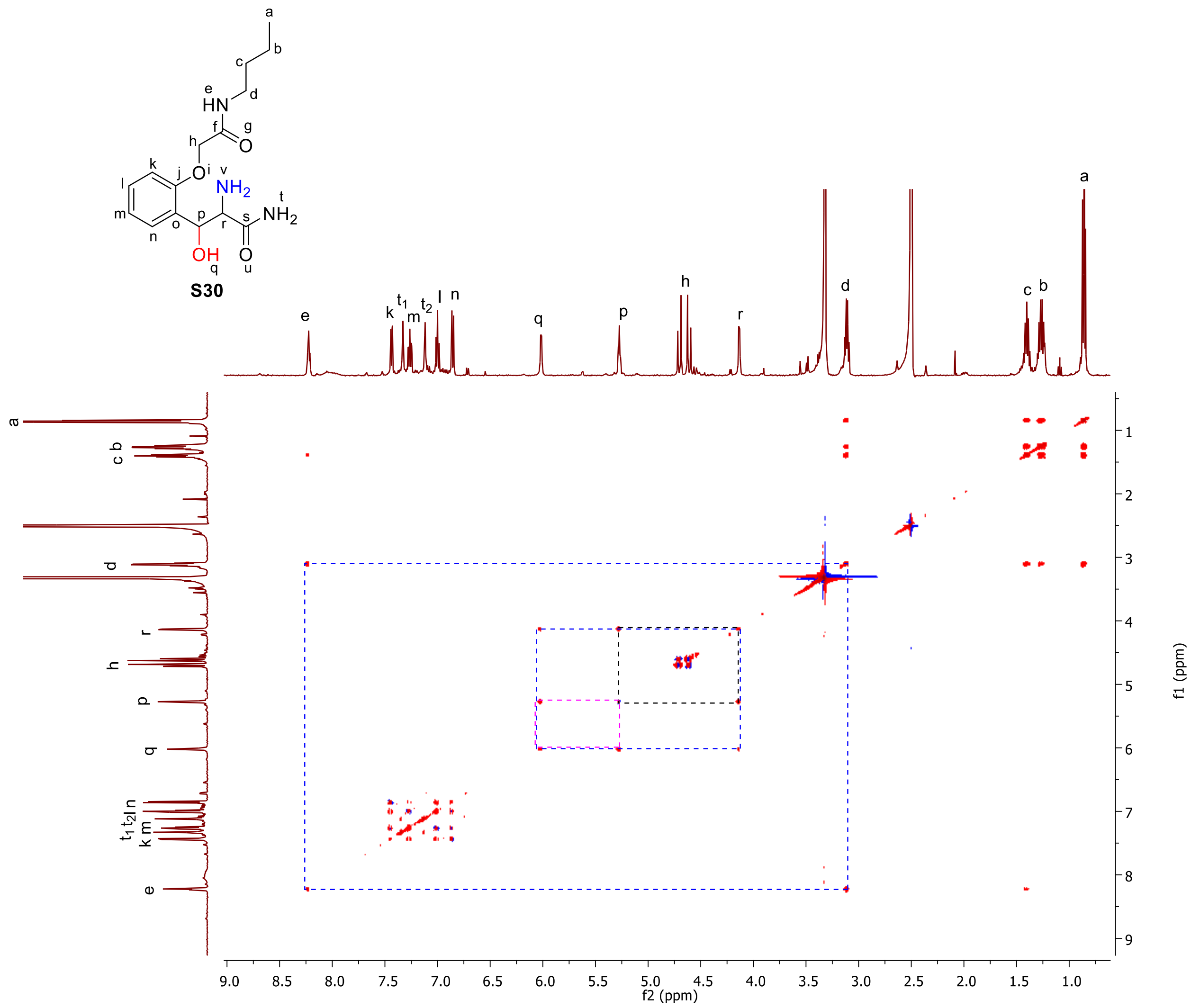

^{1}H-^{1}H correlation (TOCSY)

**^{1}H-^{1}H**	H_e	H_{h2}	H_q	H_r	H_{t2}
H_d	√	√			
H_{h1}		√			
H_p			√	√	
H_q				√	
H_{t1}					√

Supplementary Figure 24. ^{1}H-^{1}H correlation (TOCSY) spectrum in DMSO-d_6 of compound **S30**

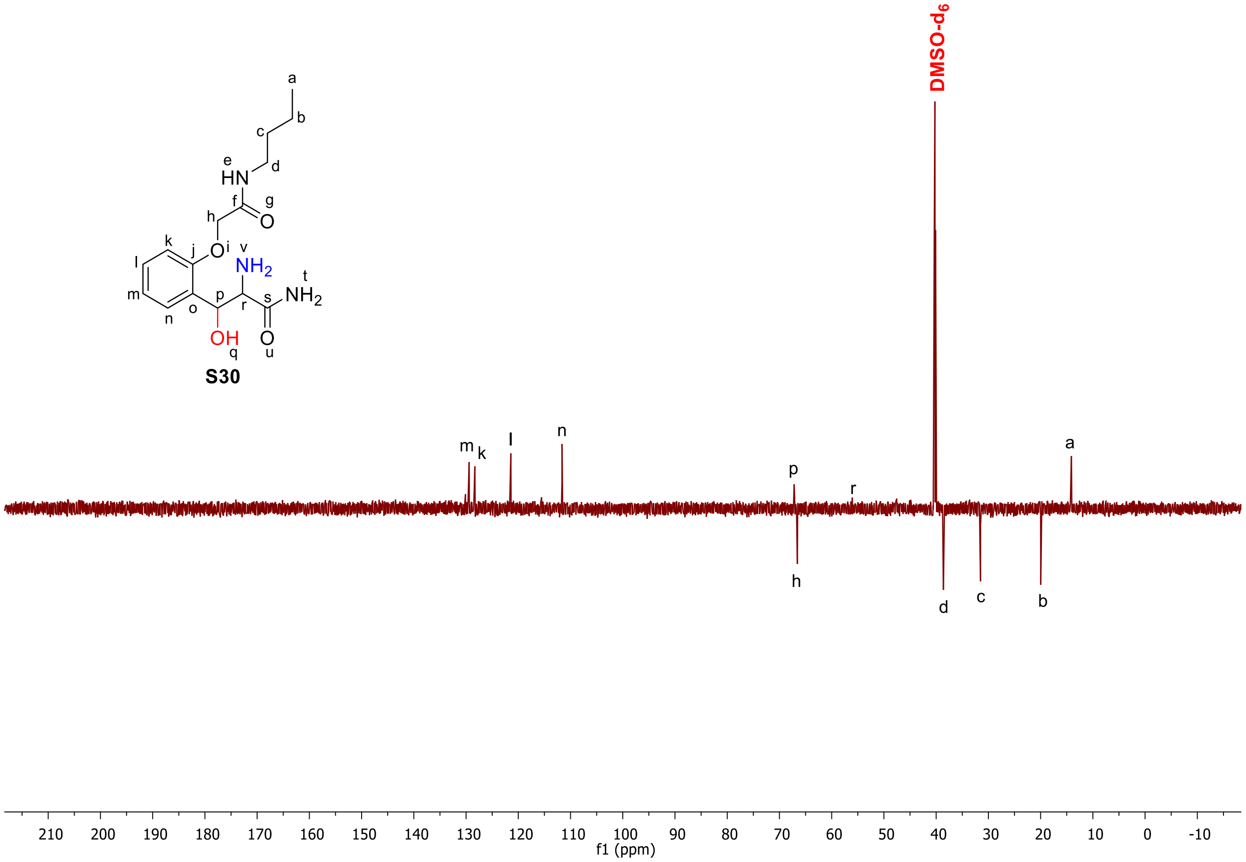

Supplementary Figure 25. DEPT-135 spectrum in DMSO-d_6 of compound **S30**

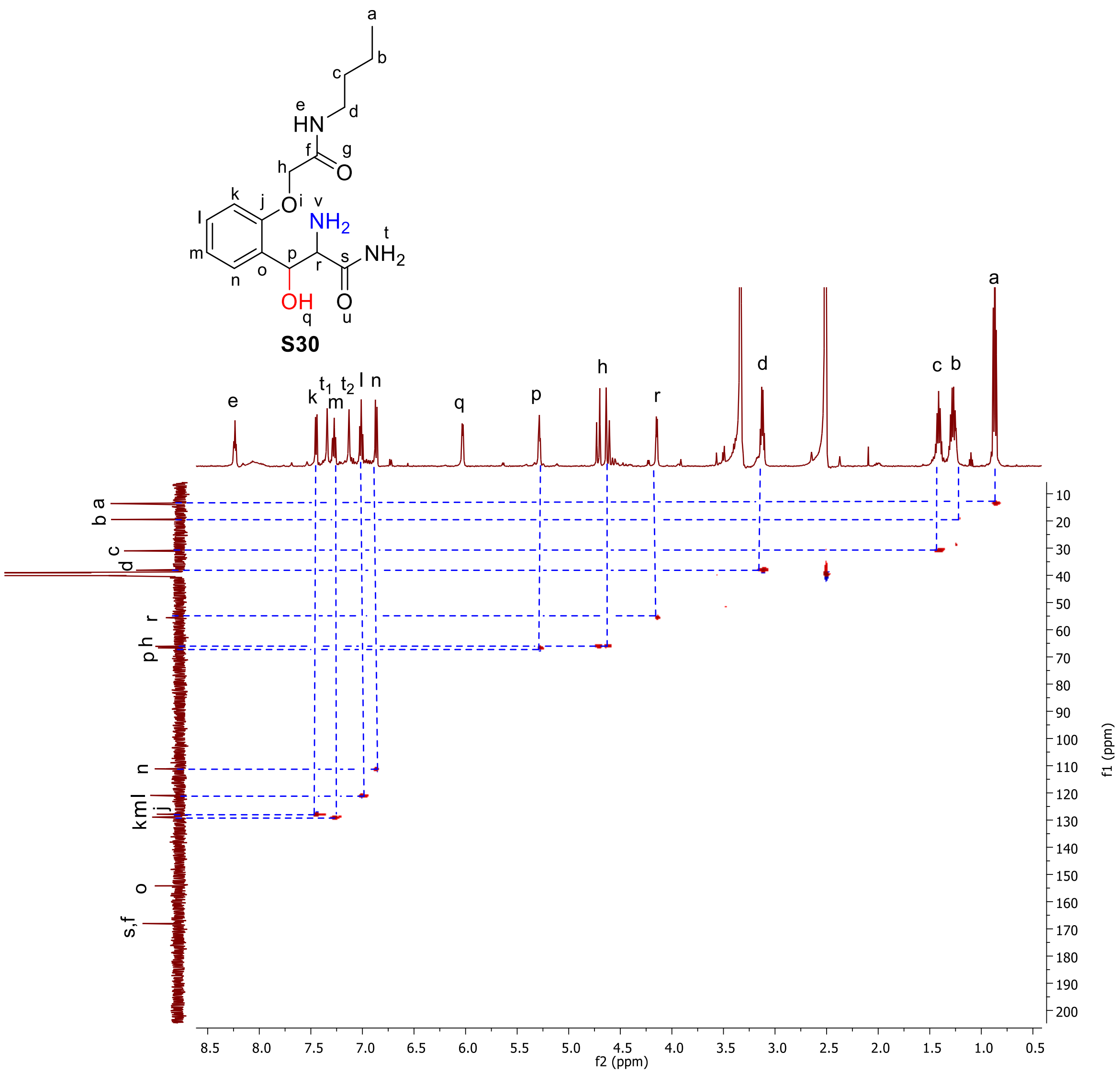

Supplementary Figure 26. ^{1}H-^{13}C correlation (HSQC) spectrum in DMSO-d_6 of compound **S30**

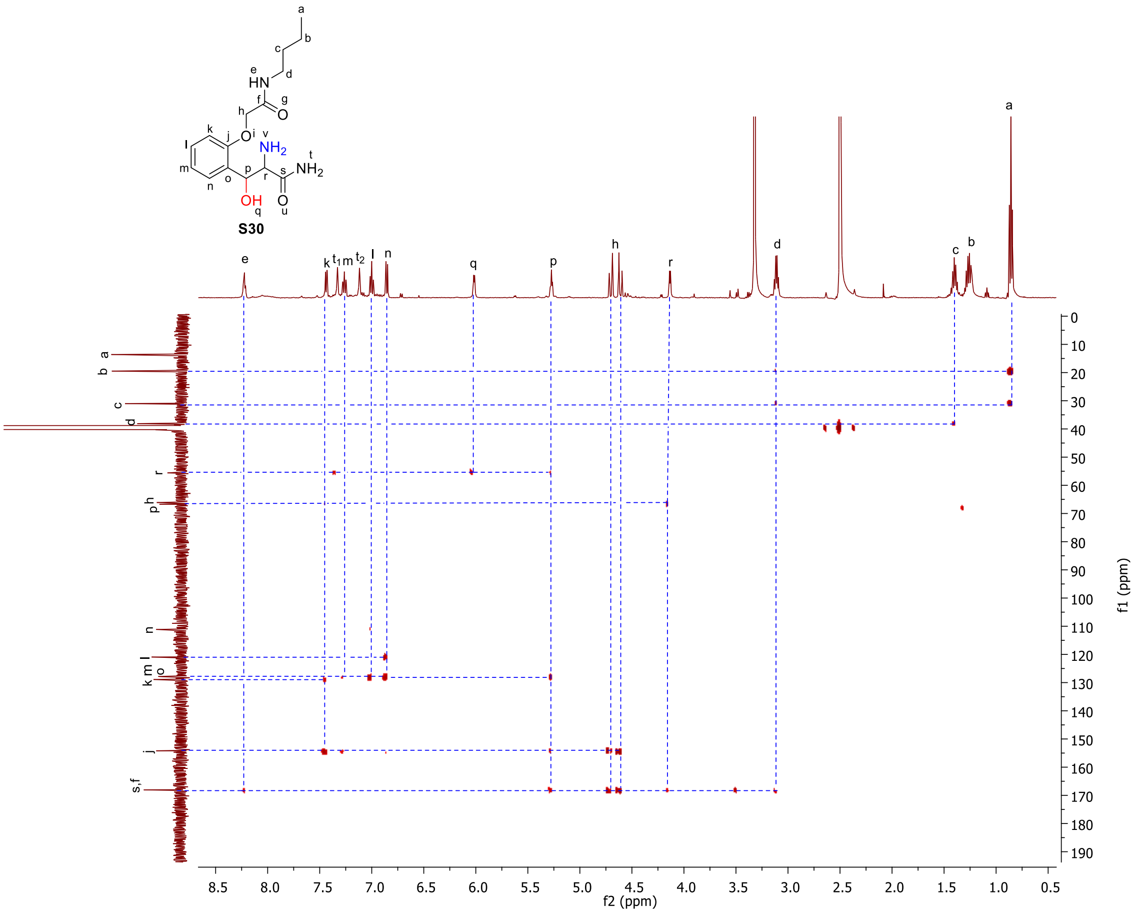

^{1}H-^{13}C correlation (HMBC)

**^{1}H-^{13}C**	H_a	H_c	H_d	H_h	H_p	H_q	H_r
C_b	√						
C_c	√						
C_d		√					
C_f			√	√			
C_j				√	√		
C_o					√		
C_p							√
C_r						√	

Supplementary Figure 27. ^{1}H-^{13}C correlation (HMBC) spectrum in DMSO-d_6 of compound **S30**

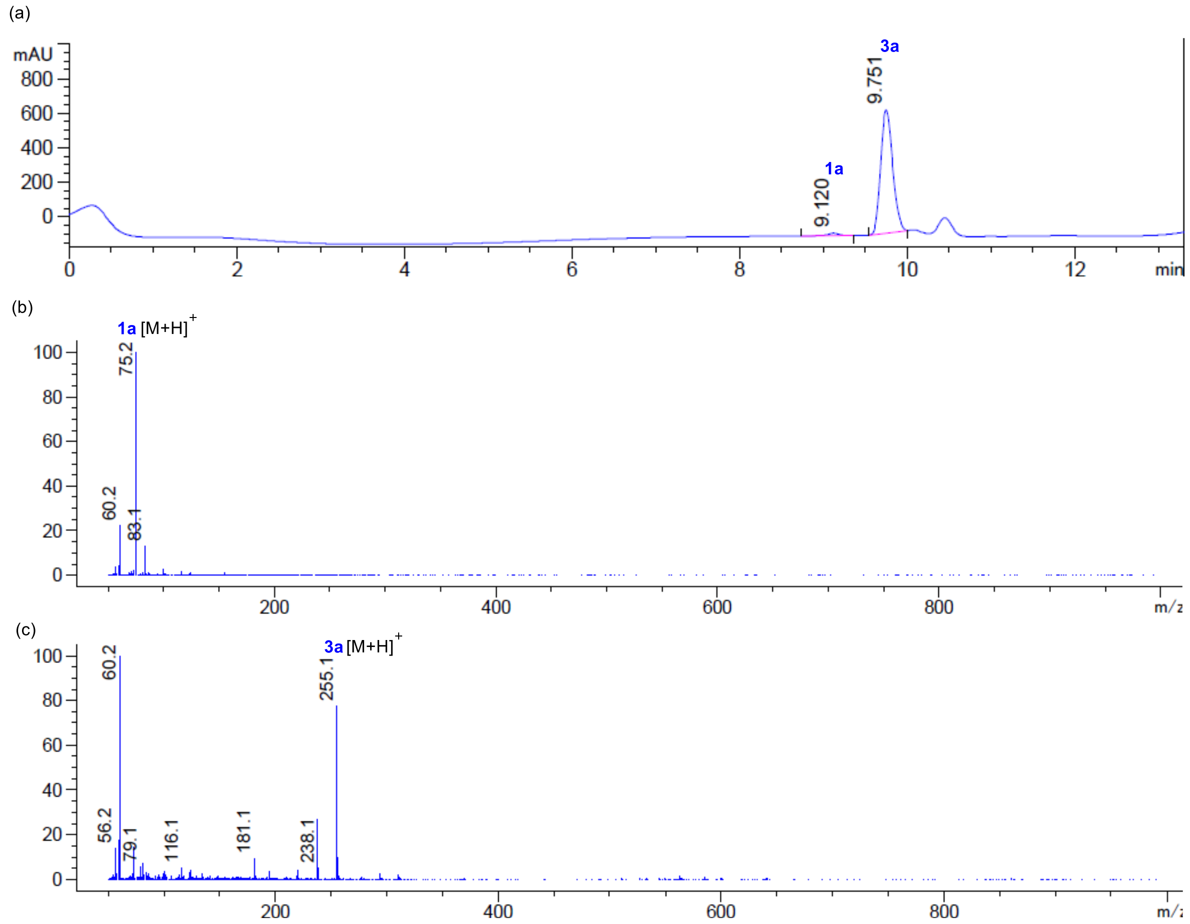

Supplementary Figure 28. LC-MS spectrum of compound **1a** and **3a**. (a) LC spectrum of **1a** and **3a**. (b) ESI-MS spectrum of **1a**. (c) ESI-MS spectrum of **3a**.

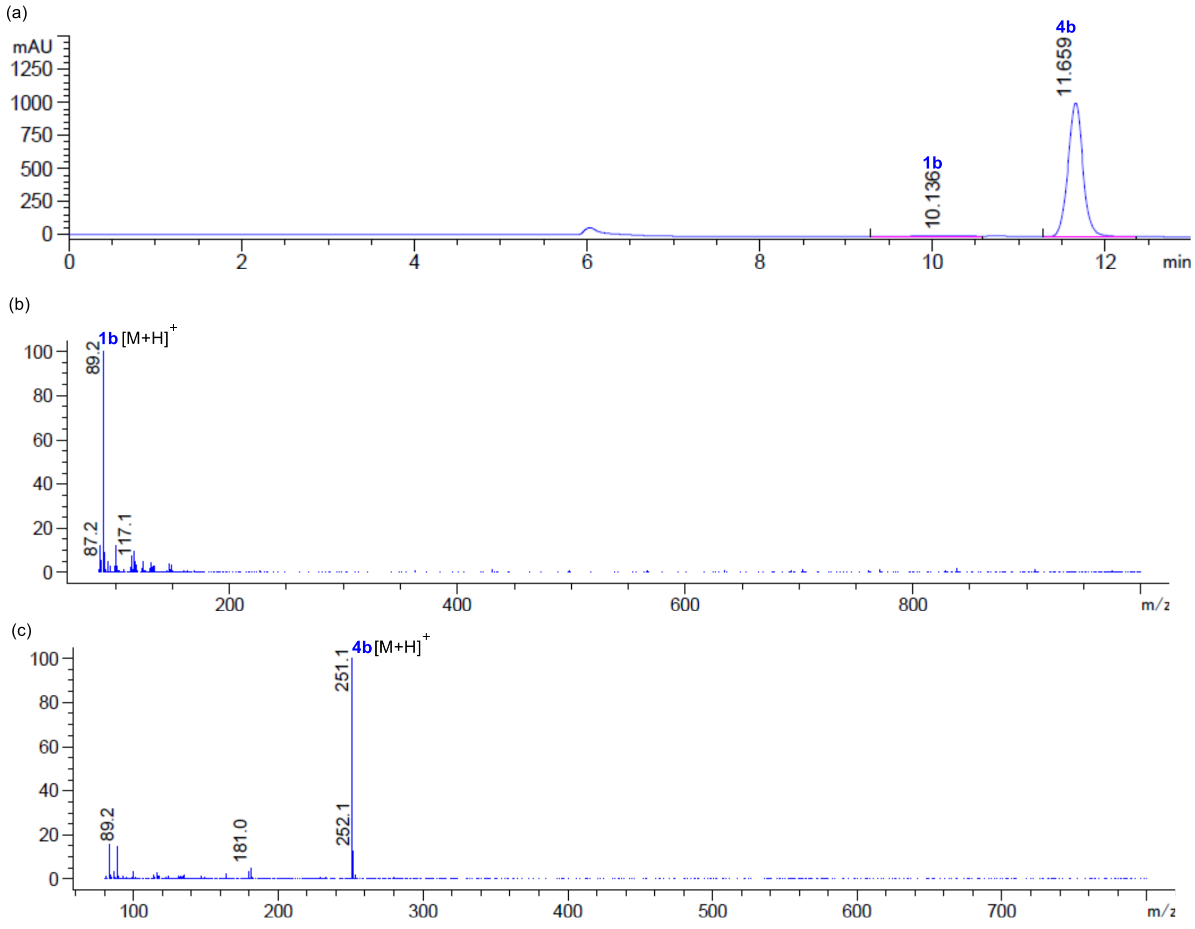

Supplementary Figure 29. LC-MS spectrum of compound **1b** and **4b**. (a) LC spectrum of **1b** and **4b**. (b) ESI-MS spectrum of **1b**. (c) ESI-MS spectrum of **4b**.

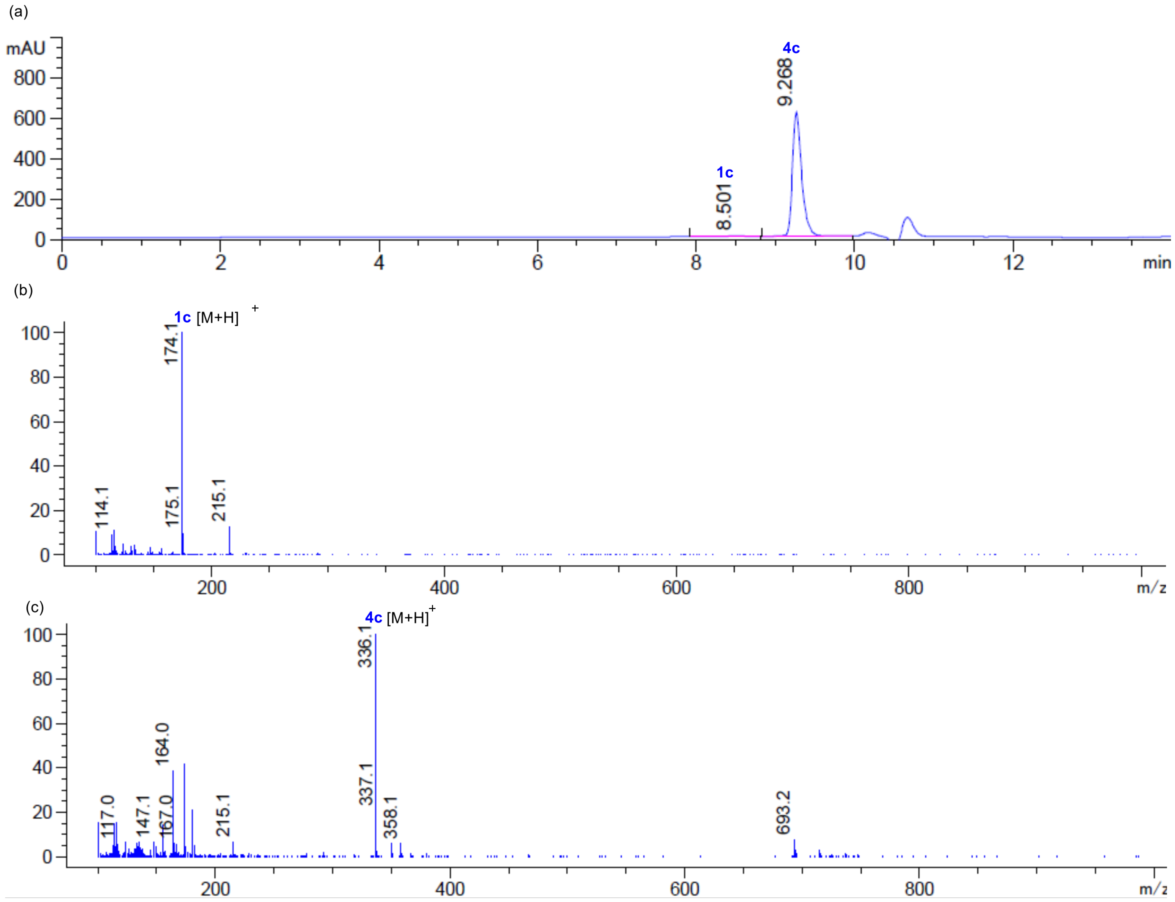

Supplementary Figure 30. LC-MS spectrum of compound **1c** and **4c**. (a) LC spectrum of **1c** and **4c**. (b) ESI-MS spectrum of **1c**. (c) ESI-MS spectrum of **4c**.

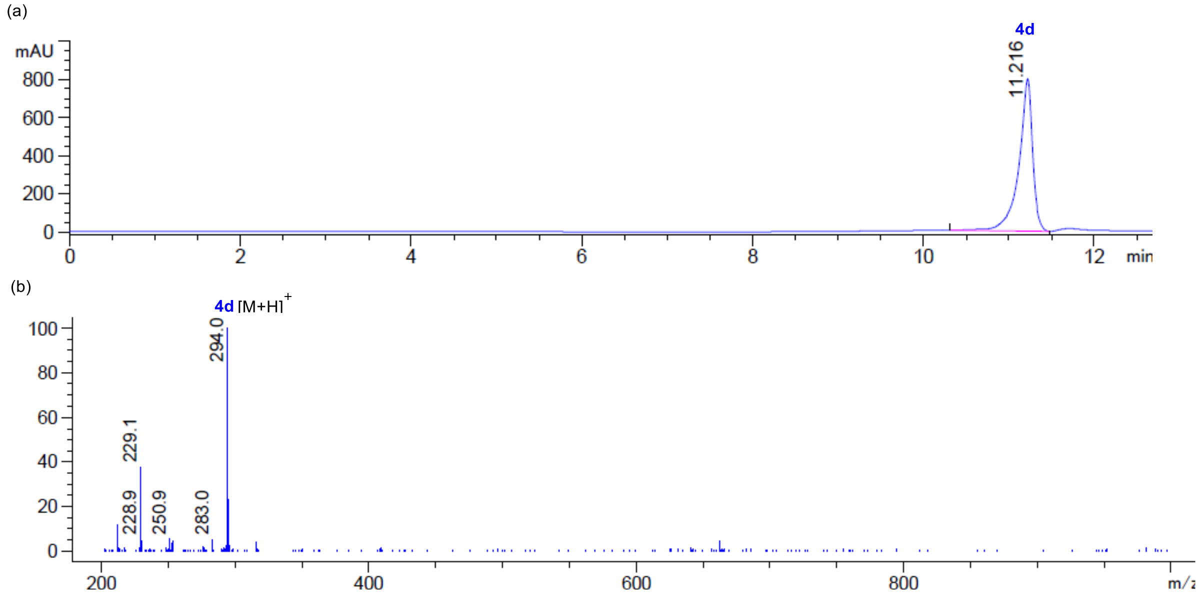

Supplementary Figure 31. LC-MS spectrum of compound **4d**. (a) LC spectrum of **4d**. (b) ESI-MS spectrum of **4d**.

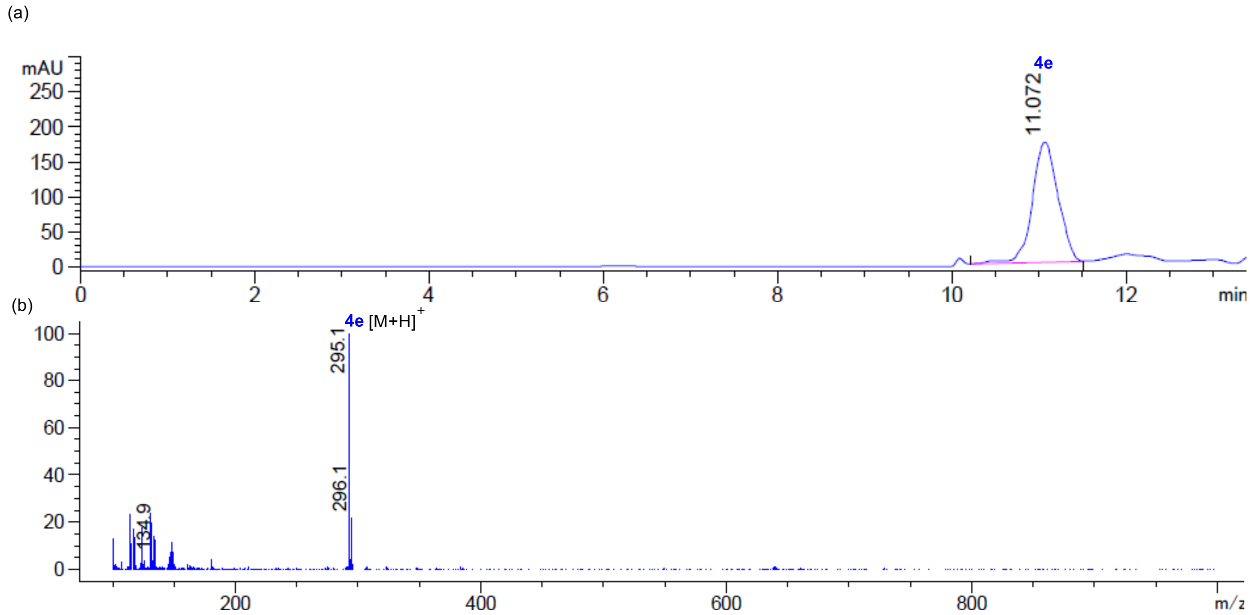

Supplementary Figure 32. LC-MS spectrum of compound **4e**. (a) LC spectrum of **4e**. (b) ESI-MS spectrum of **4e**.

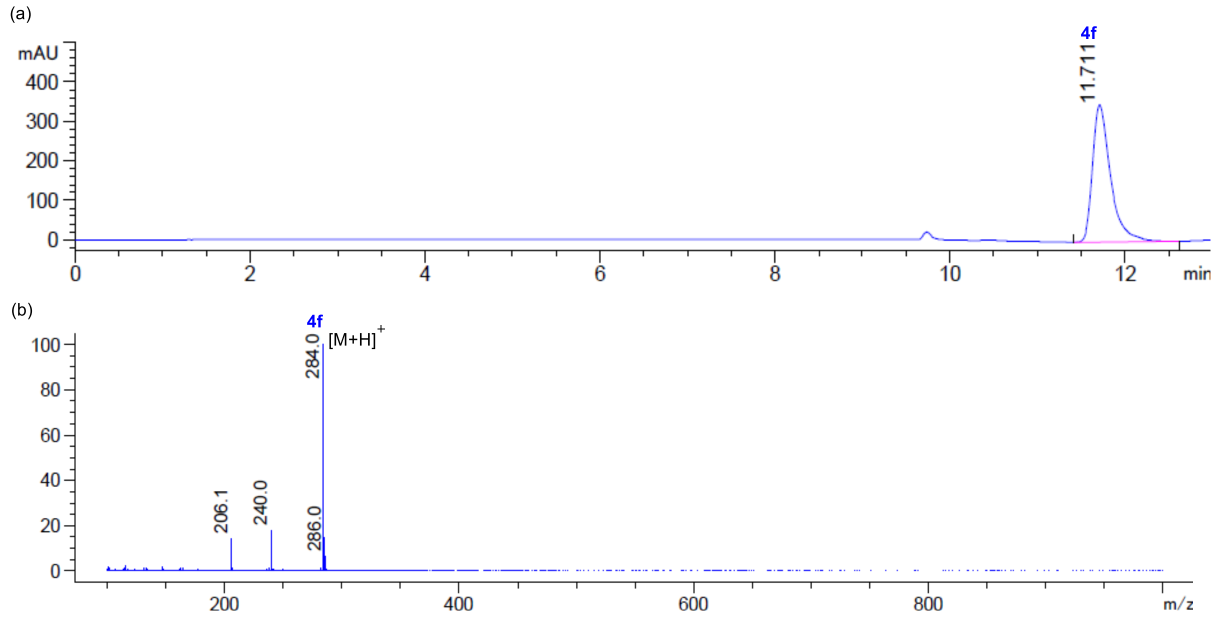

Supplementary Figure 33. LC-MS spectrum of compound **4f**. (a) LC spectrum of **4f**. (b) ESI-MS spectrum of **4f**.

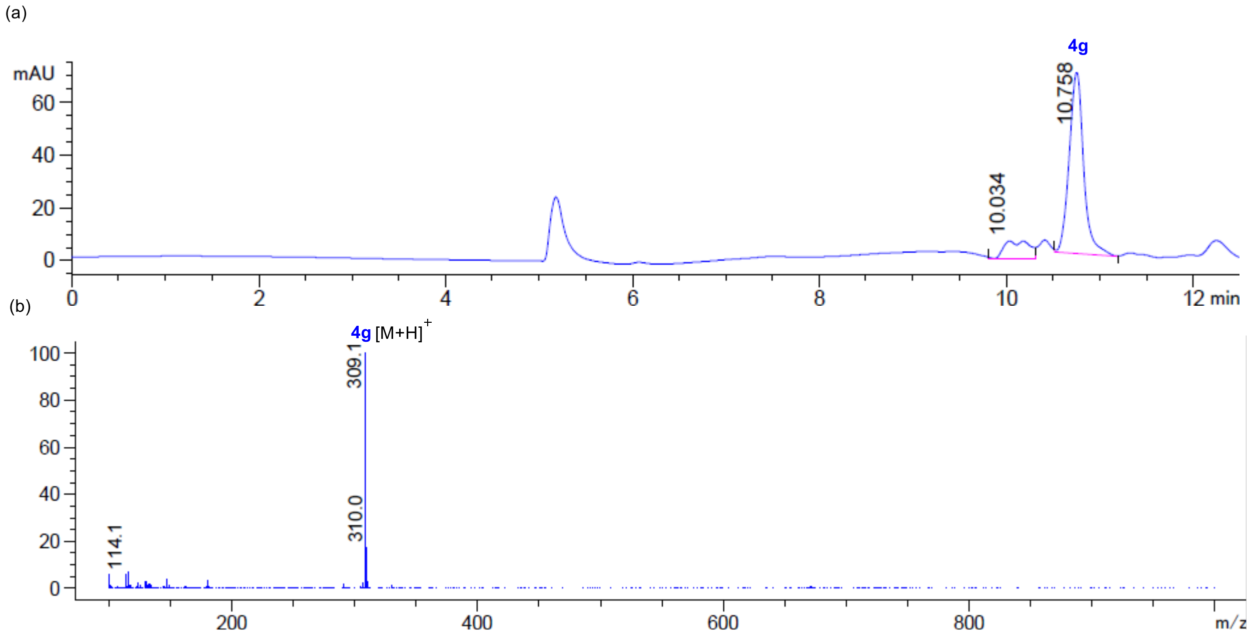

Supplementary Figure 34. LC-MS spectrum of compound **4g**. (a) LC spectrum of **4g**. (b) ESI-MS spectrum of **4g**.

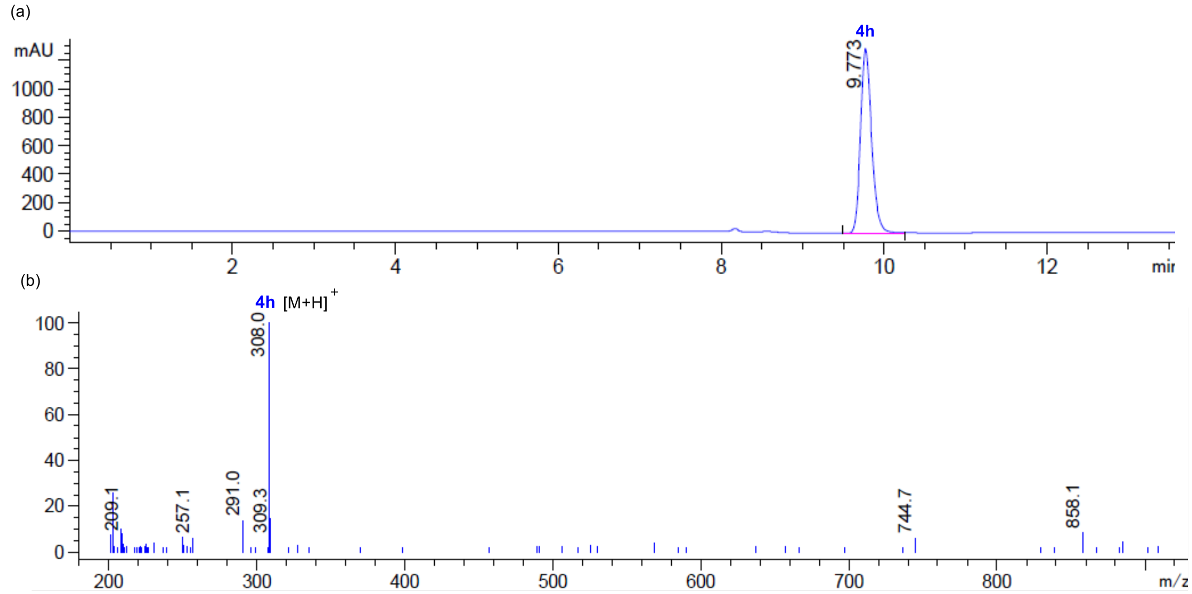

Supplementary Figure 35. LC-MS spectrum of compound **4h**. (a) LC spectrum of **4h**. (b) ESI-MS spectrum of **4h**.

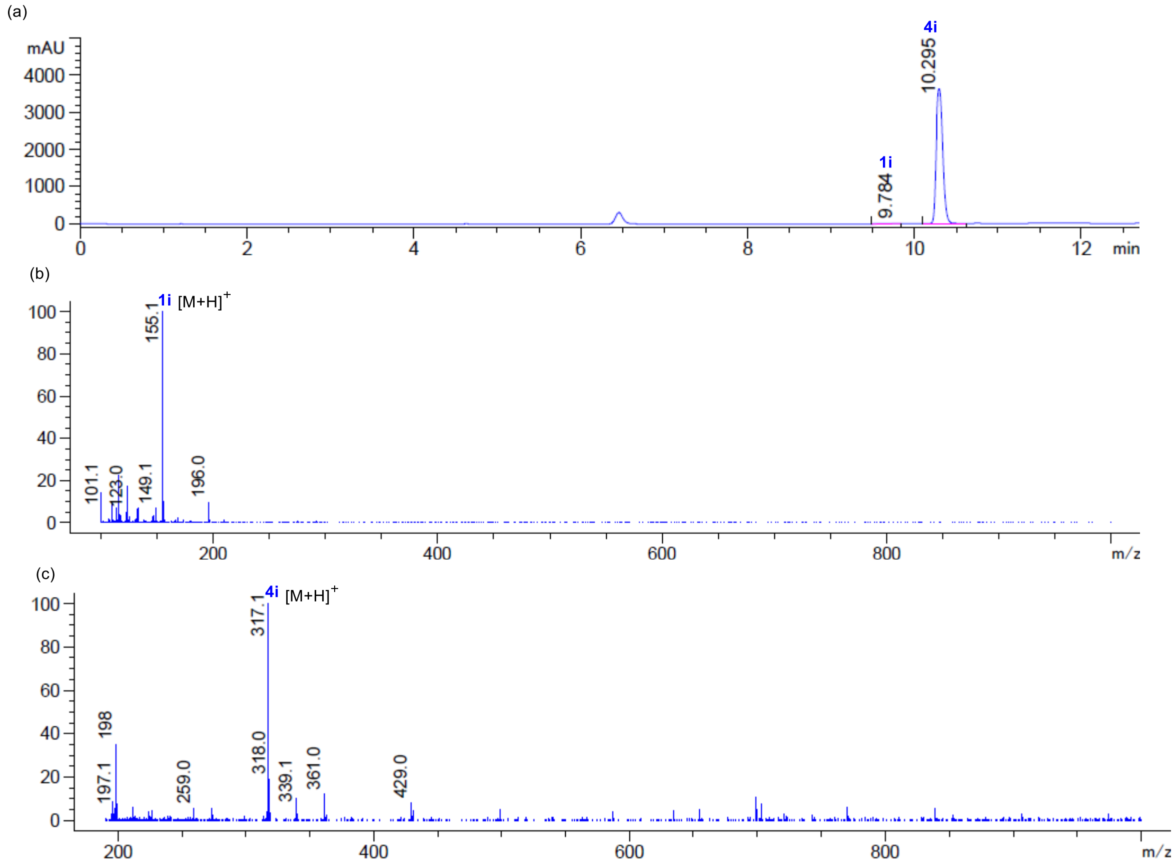

Supplementary Figure 36. LC-MS spectrum of compound **1i** and **4i**. (a) LC spectrum of **1i** and **4i**. (b) ESI-MS spectrum of **1i**. (c) ESI-MS spectrum of **4i**.

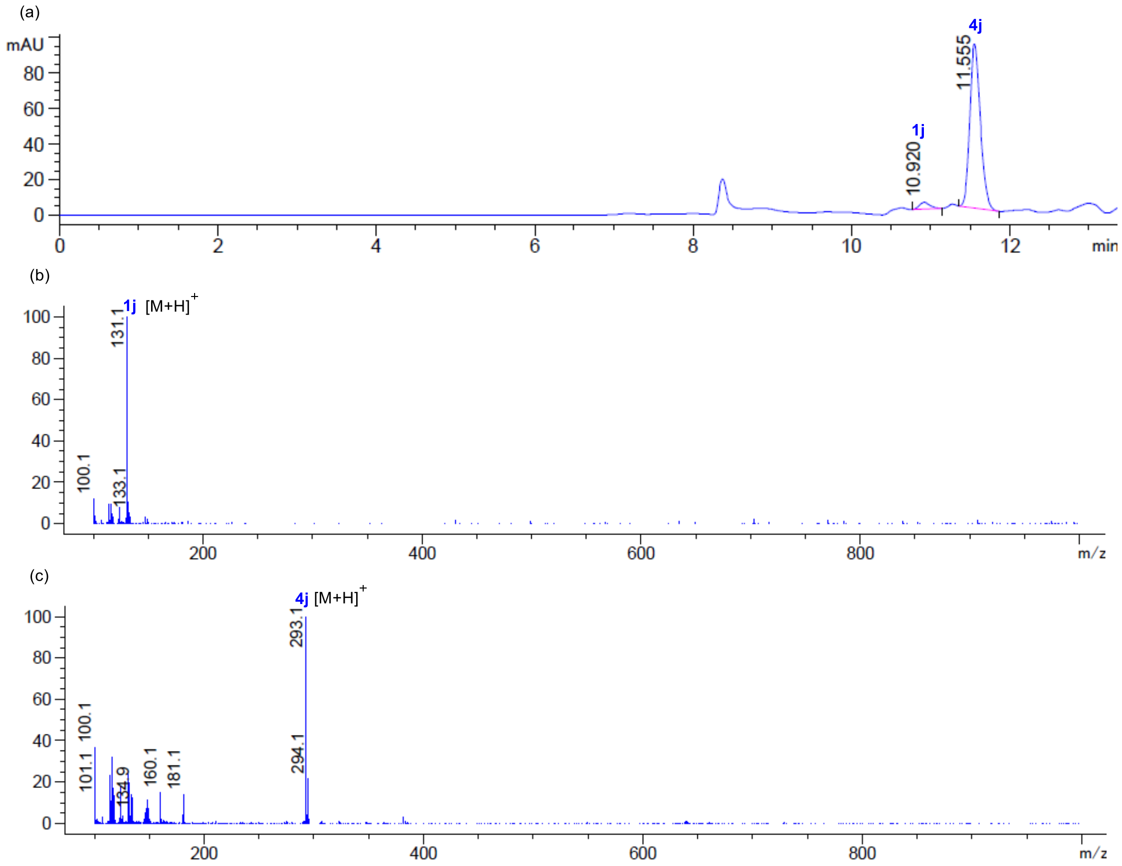

Supplementary Figure 37. LC-MS spectrum of compound **1j** and **4j**. (a) LC spectrum of **1j** and **4j**. (b) ESI-MS spectrum of **1j**. (c) ESI-MS spectrum of **4j**.

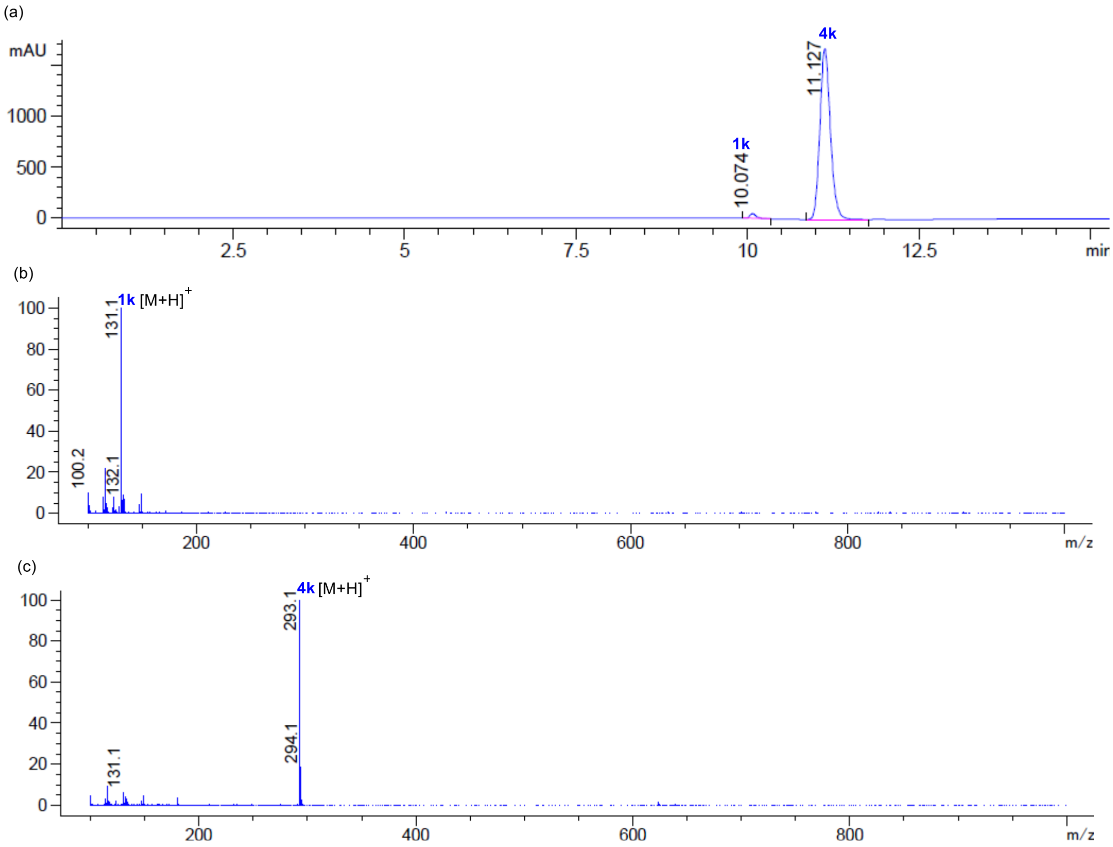

Supplementary Figure 38. LC-MS spectrum of compound **1k** and **4k**. (a) LC spectrum of **1k** and **4k**. (b) ESI-MS spectrum of **1k**. (c) ESI-MS spectrum of **4k**.

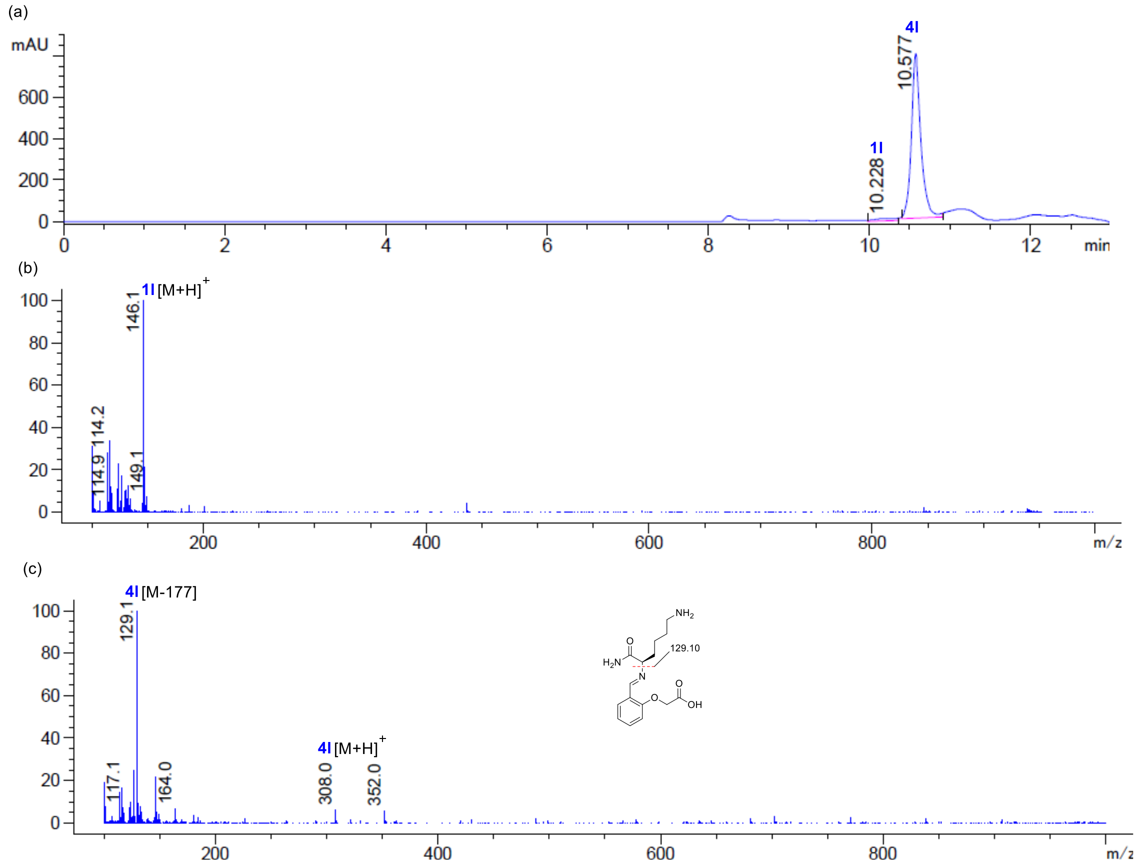

Supplementary Figure 39. LC-MS spectrum of compound **1l** and **4l**. (a) LC spectrum of **1l** and **4l**. (b) ESI-MS spectrum of **1l**. (c) ESI-MS spectrum of **4l**.

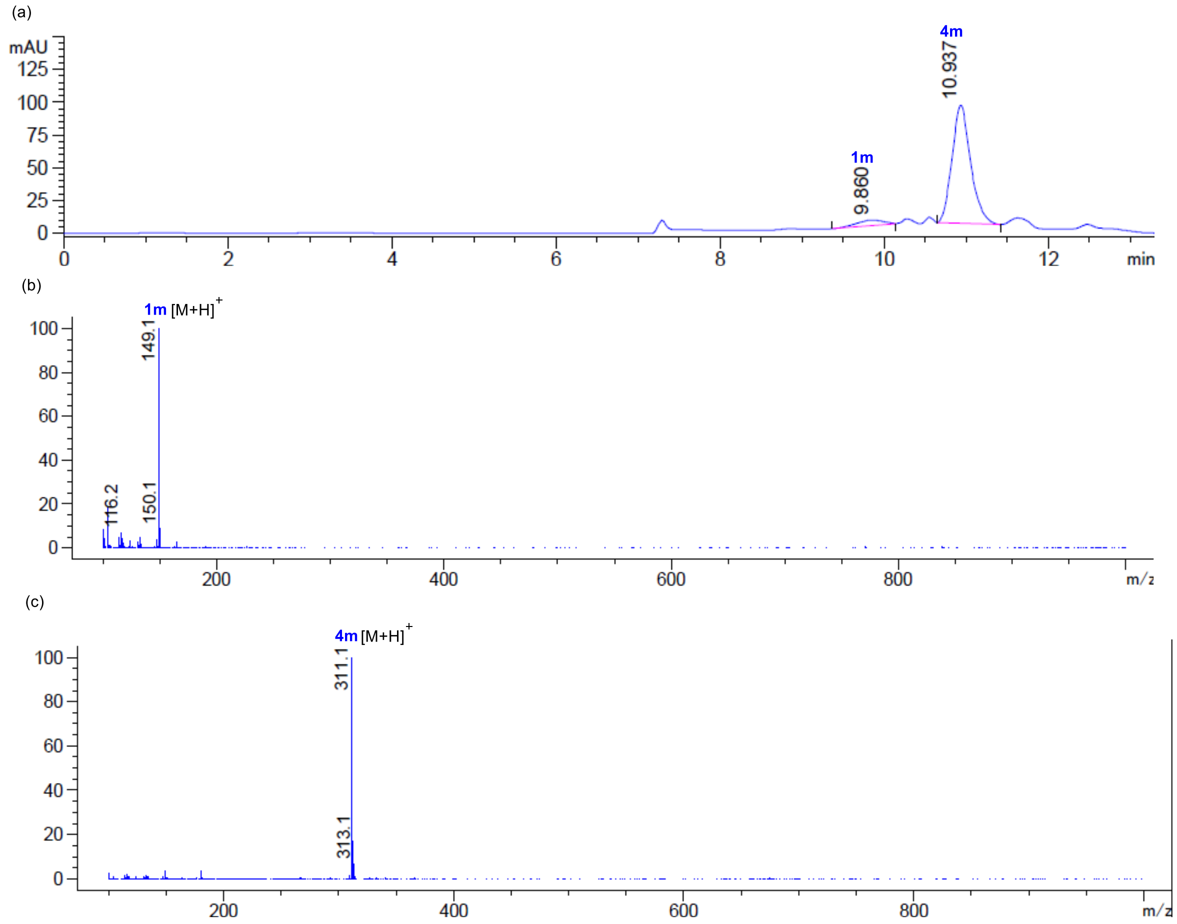

Supplementary Figure 40. LC-MS spectrum of compound **1m** and **4m**. (a) LC spectrum of **1m** and **4m**. (b) ESI-MS spectrum of **1m**. (c) ESI-MS spectrum of **4m**.

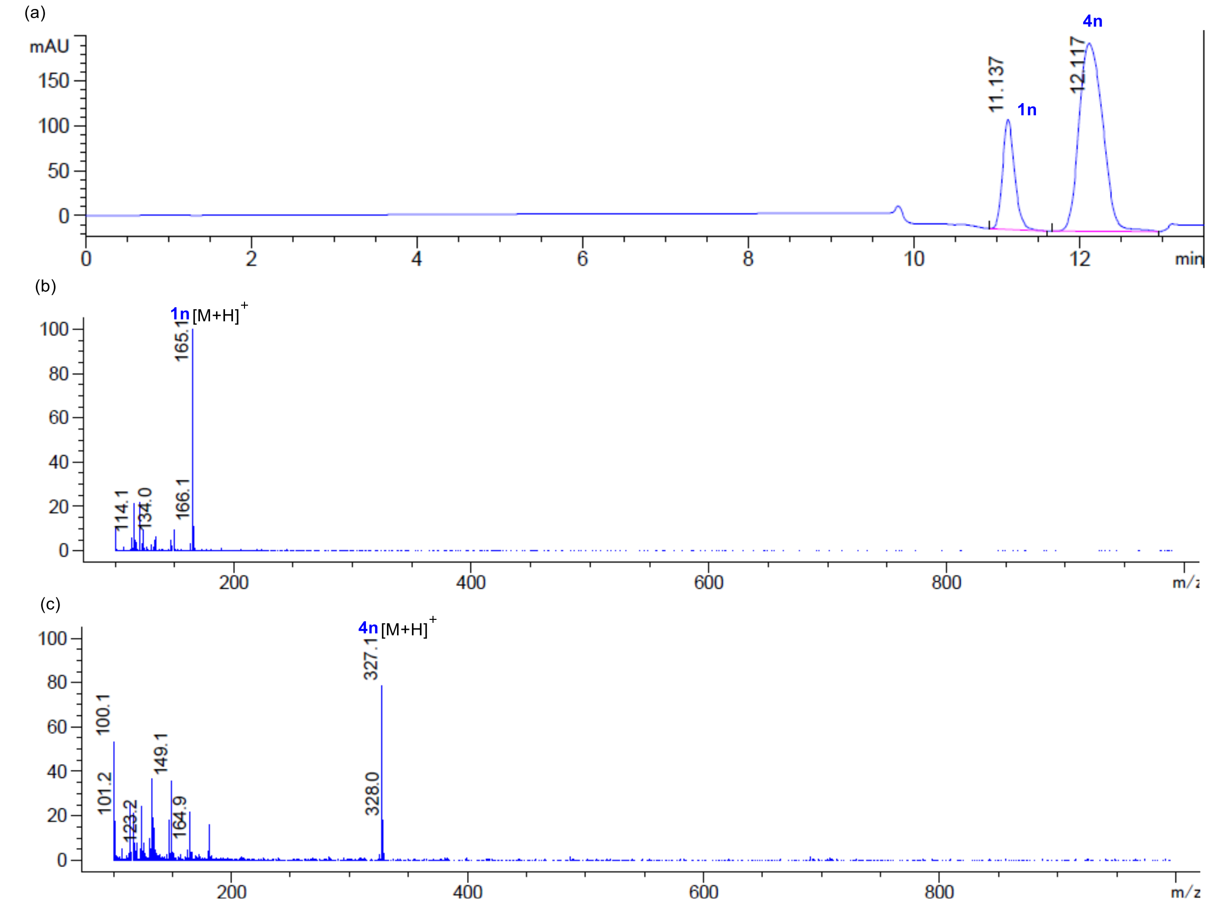

Supplementary Figure 41. LC-MS spectrum of compound **1n** and **4n**. (a) LC spectrum of **1n** and **4n**. (b) ESI-MS spectrum of **1n**. (c) ESI-MS spectrum of **4n**.

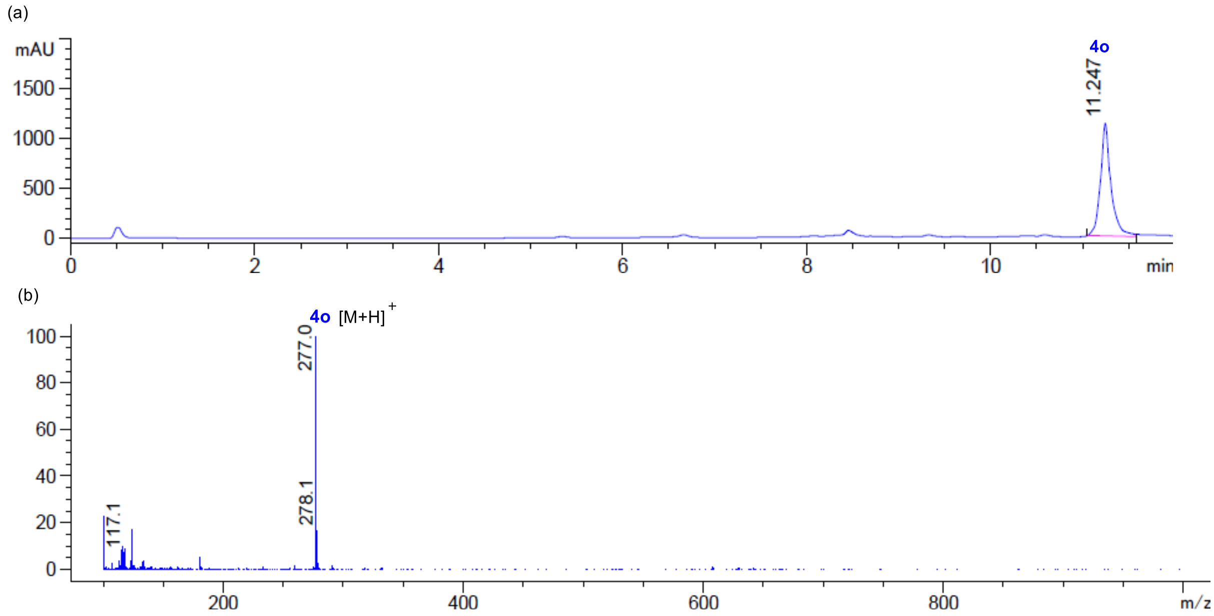

Supplementary Figure 42. LC-MS spectrum of compound **4o**. (a) LC spectrum of **4o**. (b) ESI-MS spectrum of **4o**.

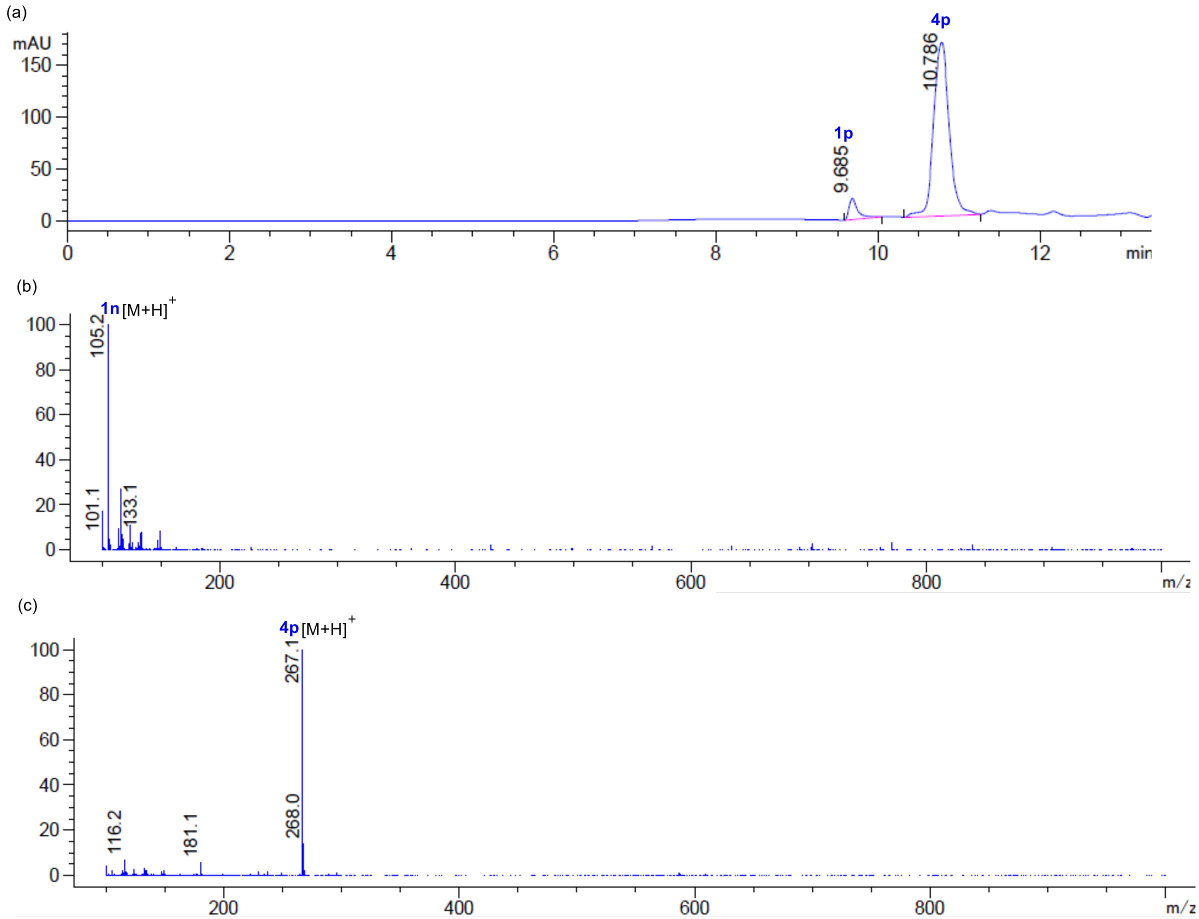

Supplementary Figure 43. LC-MS spectrum of compound **1p** and **4p**. (a) LC spectrum of **1p** and **4p**. (b) ESI-MS spectrum of **1p**. (c) ESI-MS spectrum of **4p**.

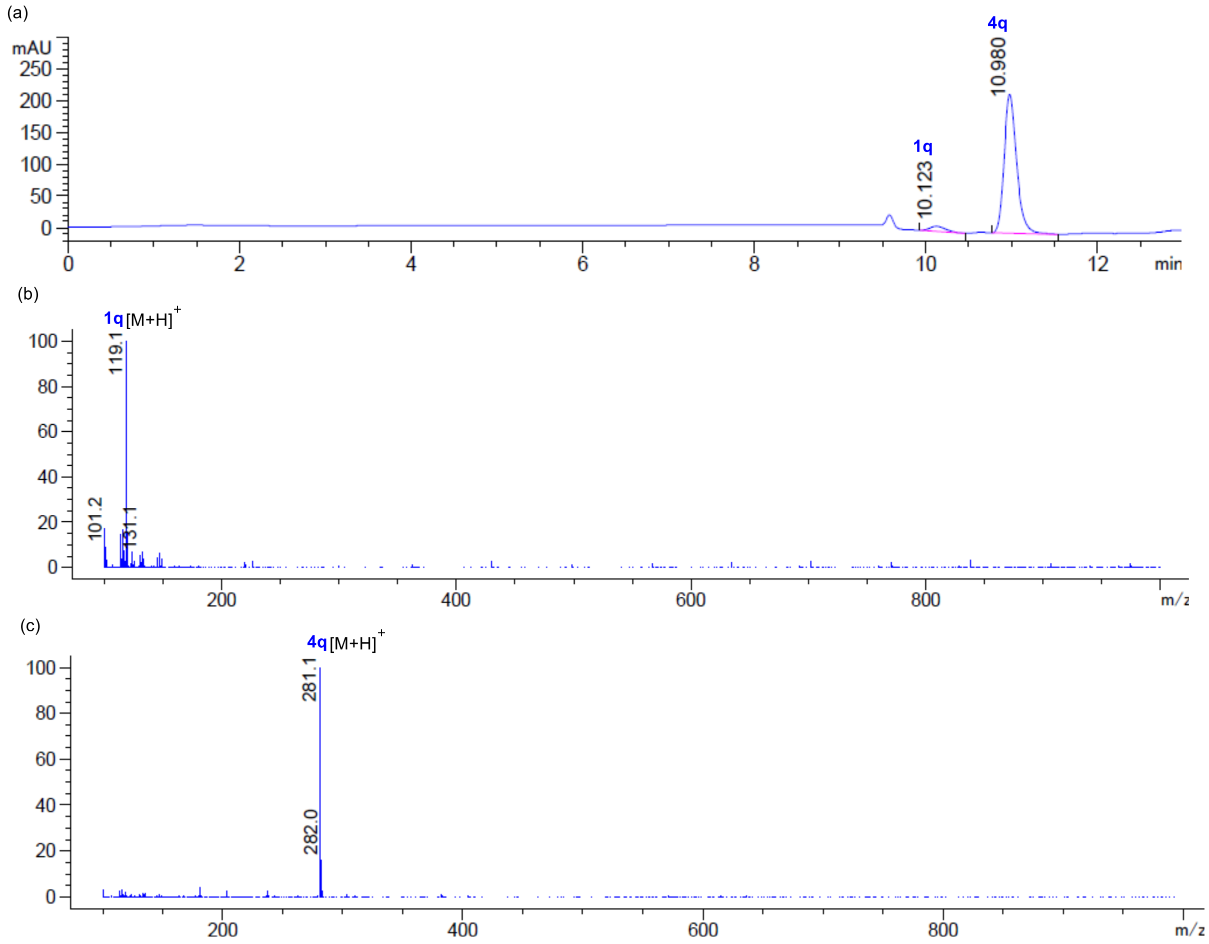

Supplementary Figure 44. LC-MS spectrum of compound **1q** and **4q**. (a) LC spectrum of **1q** and **4q**. (b) ESI-MS spectrum of **1q**. (c) ESI-MS spectrum of **4q**.

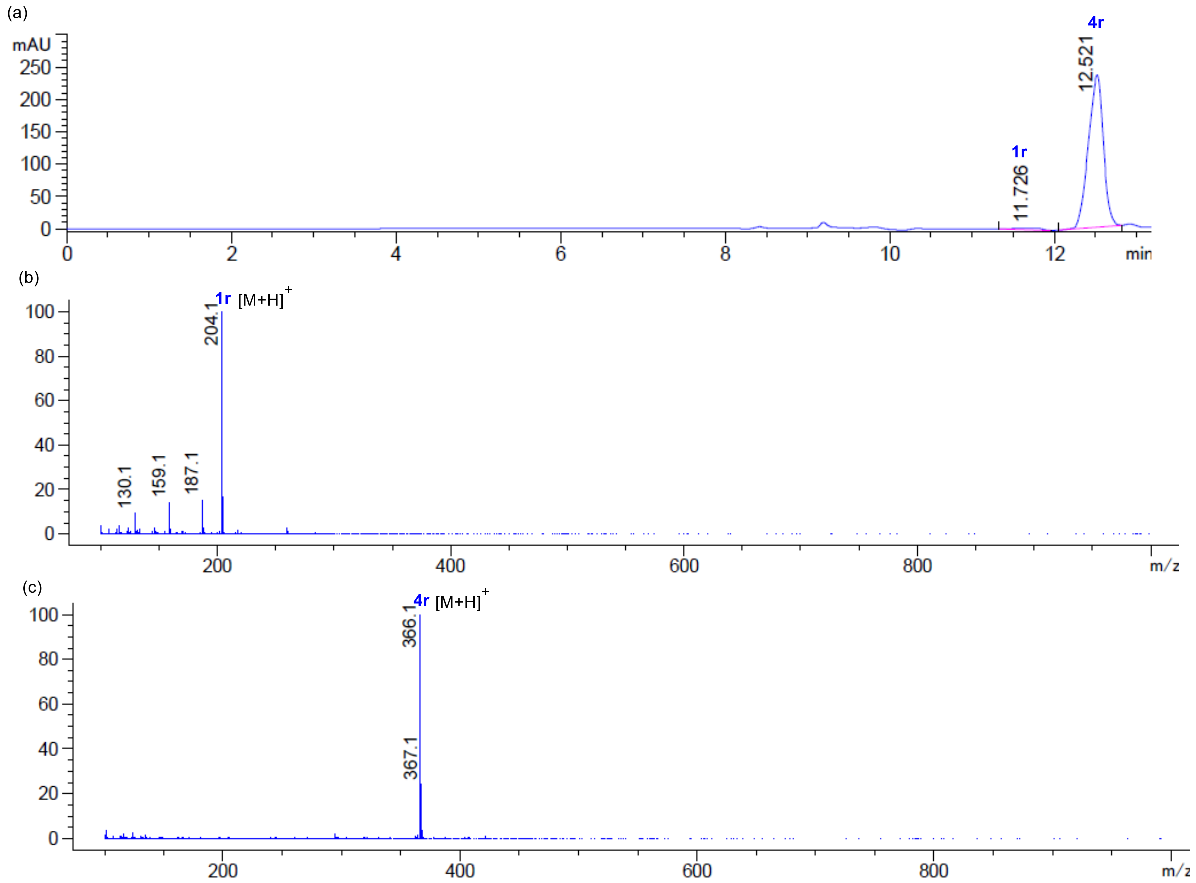

Supplementary Figure 45. LC-MS spectrum of compound **1r** and **4r**. (a) LC spectrum of **1r** and **4r**. (b) ESI-MS spectrum of **1r**. (c) ESI-MS spectrum of **4r**.

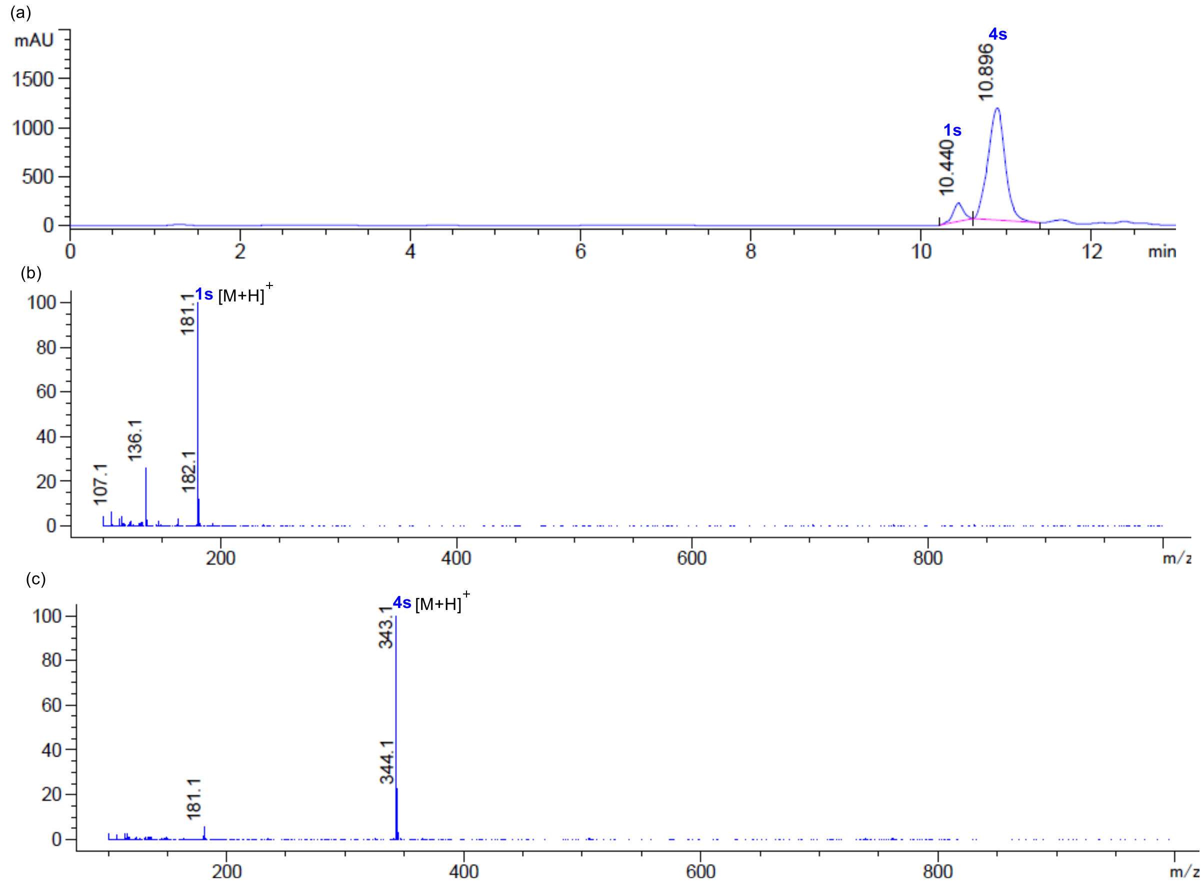

Supplementary Figure 46. LC-MS spectrum of compound **1s** and **4s**. (a) LC spectrum of **1s** and **4s**. (b) ESI-MS spectrum of **1s**. (c) ESI-MS spectrum of **4s**.

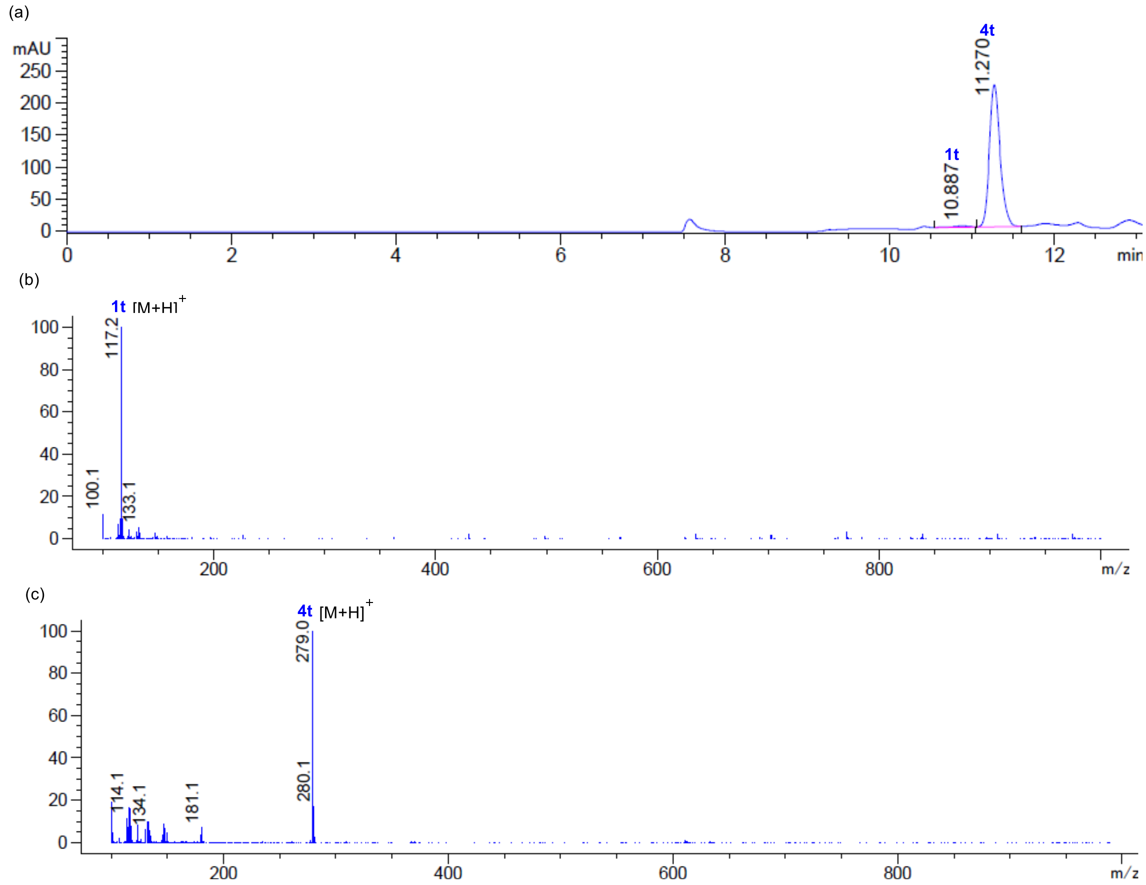

Supplementary Figure 47. LC-MS spectrum of compound **1t** and **4t**. (a) LC spectrum of **1t** and **4t**. (b) ESI-MS spectrum of **1t**. (c) ESI-MS spectrum of **4t**.

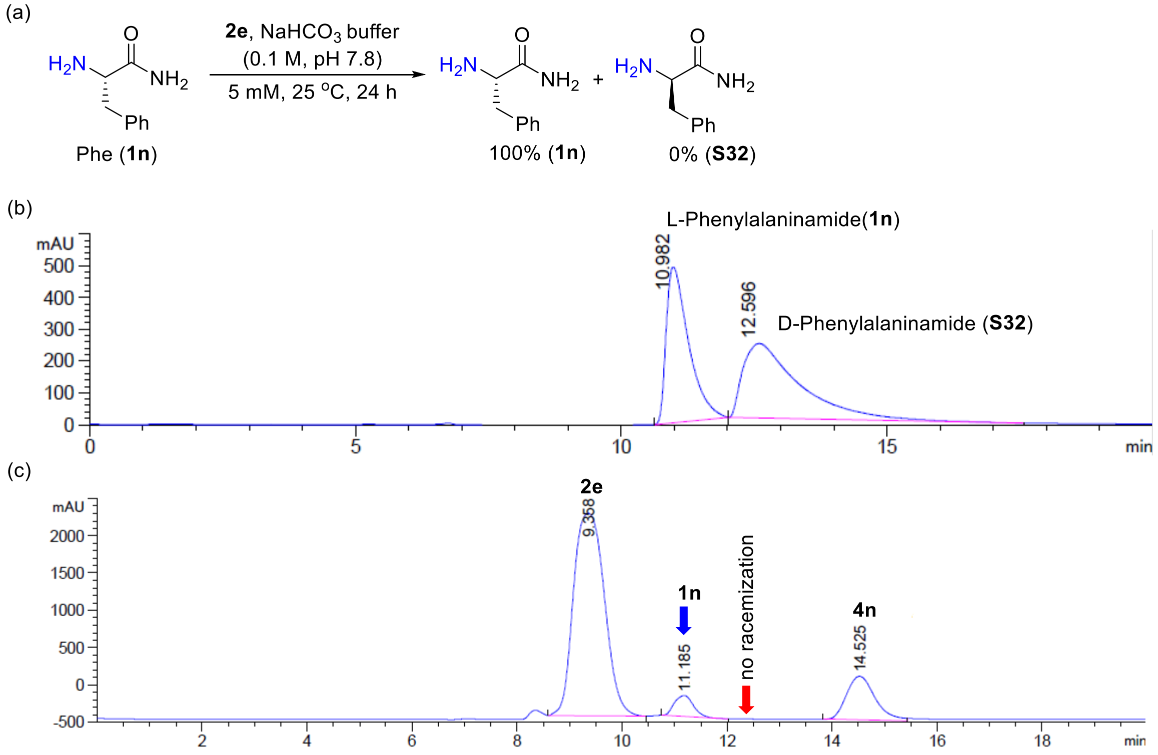

Supplementary Figure 48. HPLC profile **(a)** Stereo-stability of substituted amino acid (**1n**) under the reaction conditions. **(b)** Mixture of L-phenylalaninamide **1n** and D-phenylalaninamide **S32** (1:1) ratio. **(c)** Stereo-stability of substituted amino acid **(1n)** under the reaction conditions.

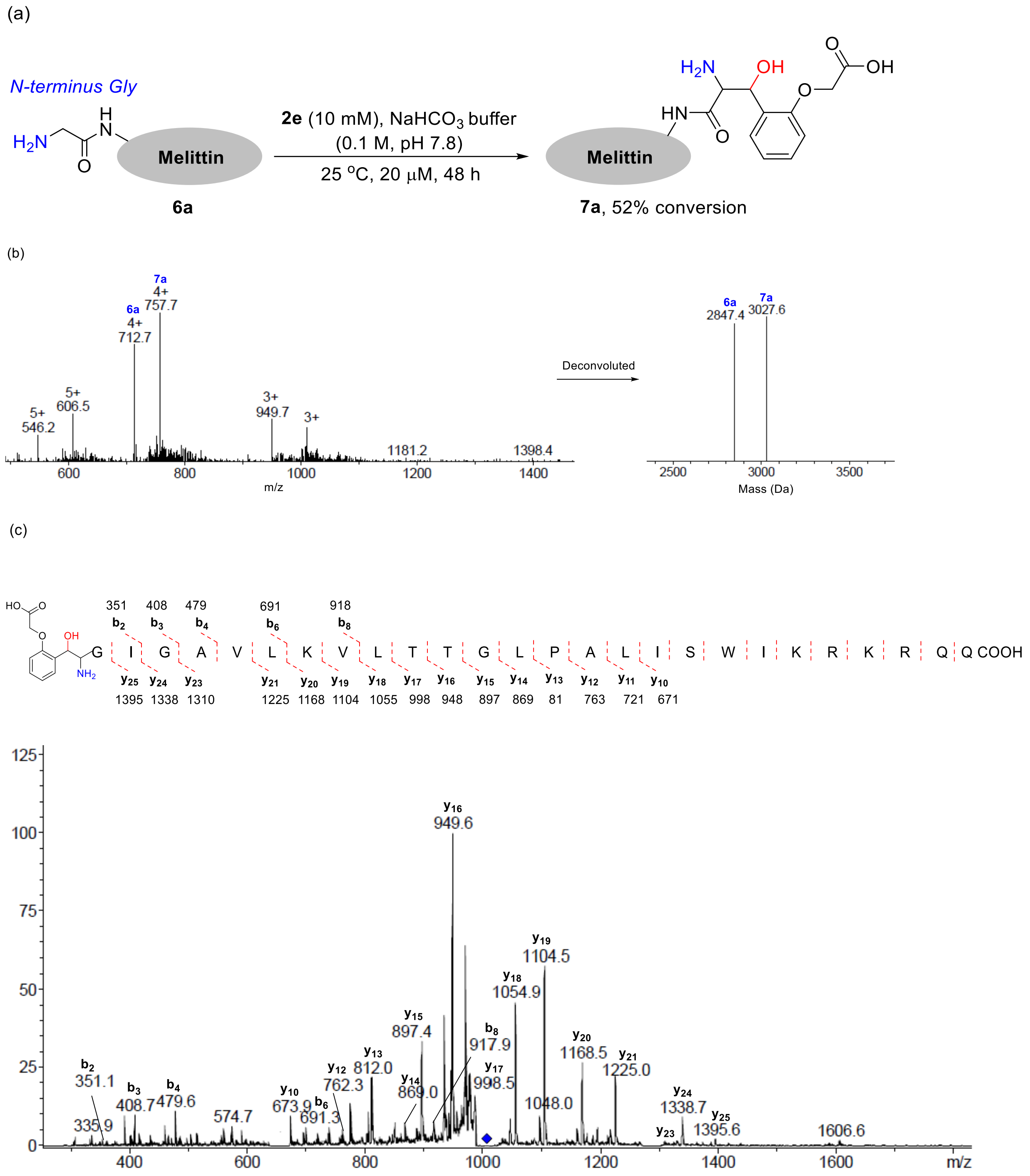

Supplementary Figure 49. Single-site labeling of Melittin **6a**. **(a)** N-terminus Gly labeling of Melittin with reagent **2e**. **(b)** ESI-MS spectra for melittin **6a** (1 equiv.) and aldehyde **2e** (500 equiv.), mono-labeled melittin **7a**. **(c)** MS-MS spectrum of labeled GIGAVLKVLTTGLPALISWIKRKRQQ (G1-Q26). Site of modification is N-terminus glycine (G1) in labeled melittin **7a**.

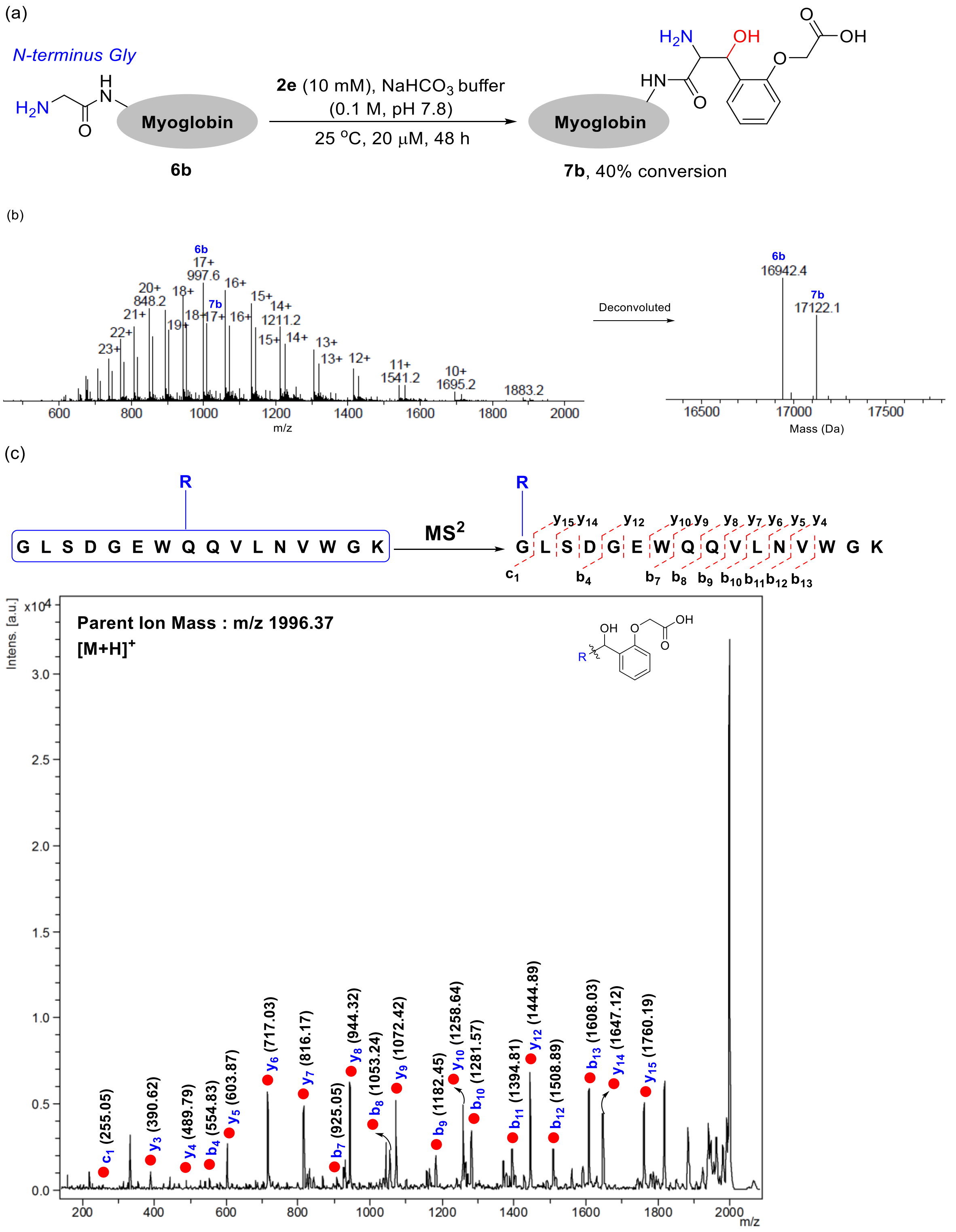

Supplementary Figure 50. Single-site labeling of Myoglobin **6b**. **(a)** N-terminus Gly labeling of Myoglobin with reagent **2e**. **(b)** ESI-MS spectra for myoglobin **6b** (1 equiv.) and aldehyde **2e** (500 equiv.), mono-labeled myoglobin **7b**. **(c)** MS-MS spectrum of labeled GLSDGEWQQVLNVWGK (G1-K16). Site of modification is N-terminus glycine (G1) in labeled myoglobin **7b**.

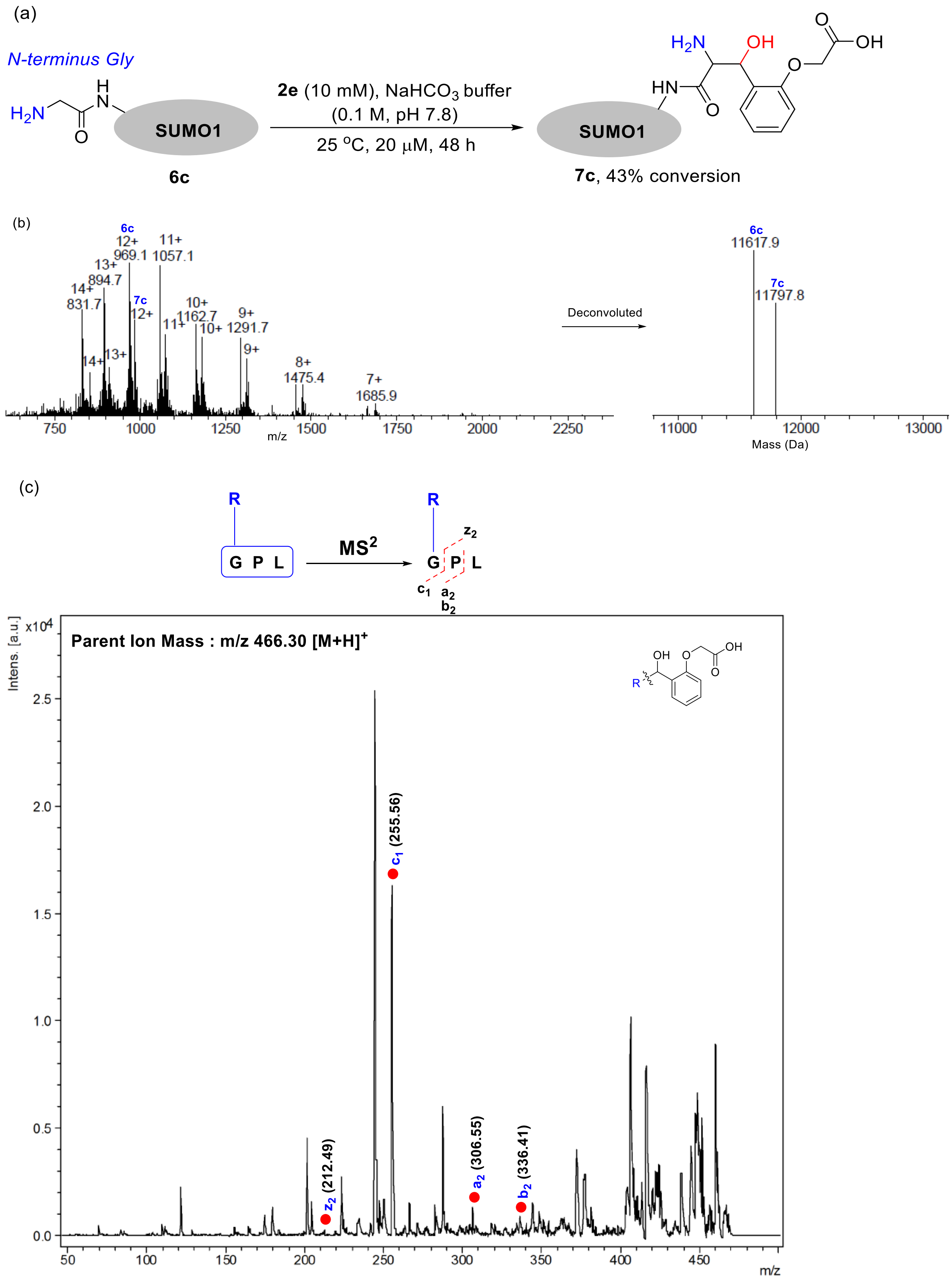

Supplementary Figure 51. Single-site labeling of SUMO1 **6c**. **(a)** N-terminus Gly labeling of SUMO1 with reagent **2e**. **(a)** ESI-MS spectra for SUMO1 **6c** (1 equiv.) and aldehyde **2e** (500 equiv.), mono-labeled SUMO1 **7c**. **(b)** MS-MS spectrum of labeled GPL (G1-L3). Site of modification is N-terminus glycine (G1) in labeled SUMO1 **7c**.

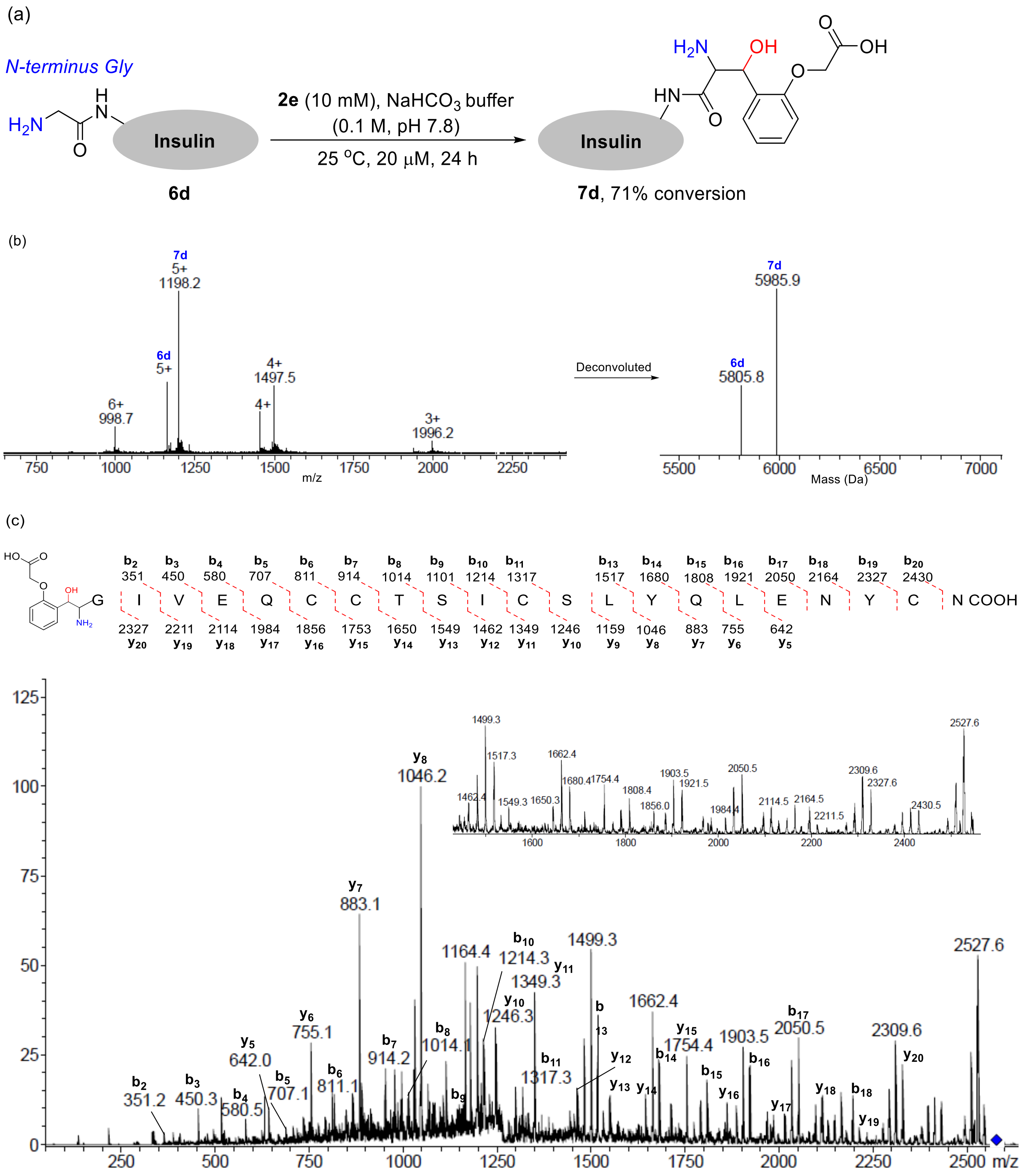

Supplementary Figure 52. Single-site labeling of insulin **6d**. **(a)** N-terminus Gly labeling of insulin with reagent **2e**. **(b)** ESI-MS spectra for insulin **6d** (1 equiv.) and aldehyde **2e** (500 equiv.), mono-labeled insulin **7d**. **(c)** MS-MS spectrum of labeled GLSDGEWQQVLNVWGK (G1-K16). Site of modification is N-terminus glycine (G1) in labeled insulin **7d**.

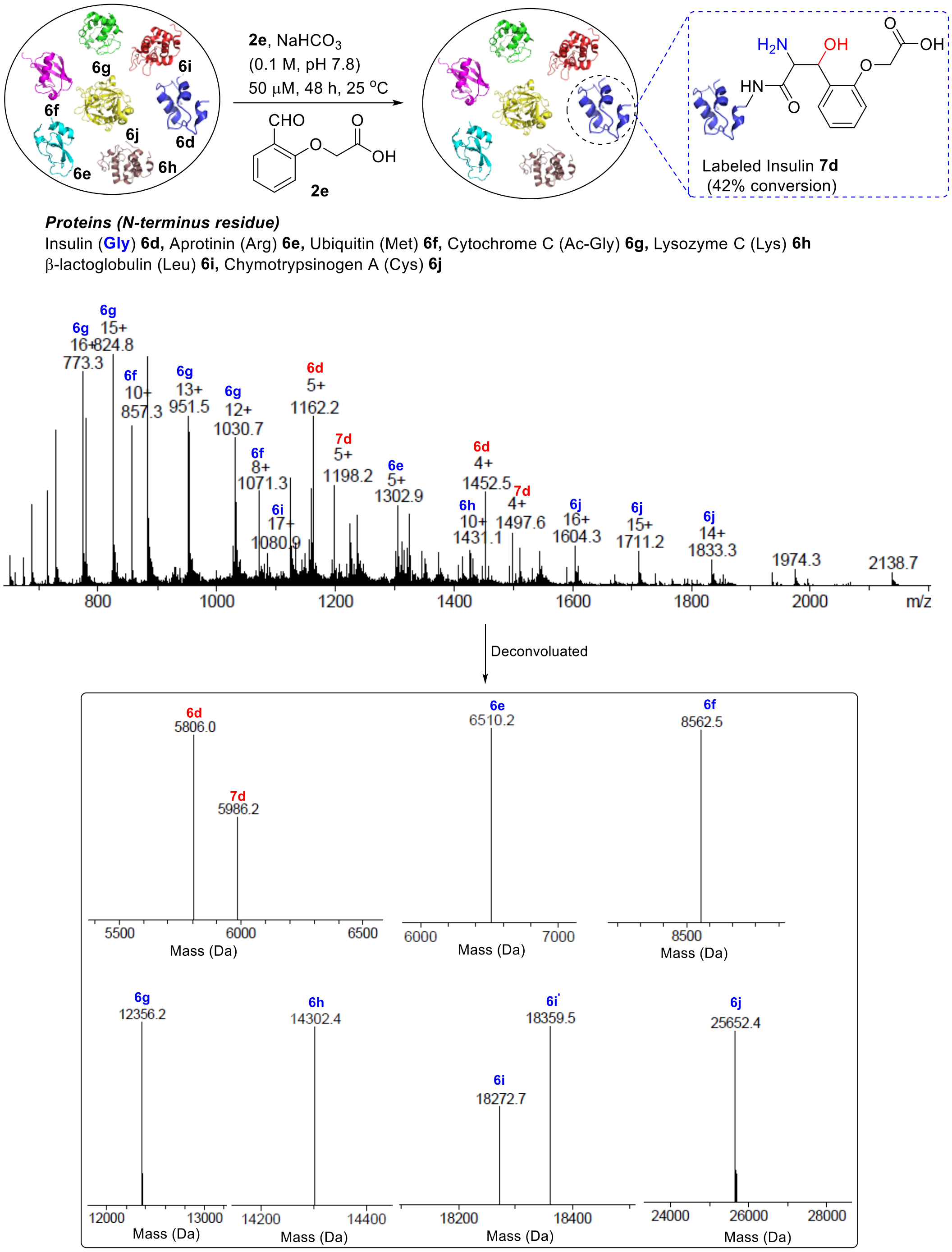

Supplementary Figure 53. Single-site, single-protein labeling in a protein mixture. ESI-MS data suggests that mono labeled Insulin (**7d**) was found in a protein mixture. The commercially available β-lactoglobulin (Sigma-Aldrich, L3908) contains variants A and B. No modification was observed for both the variants.

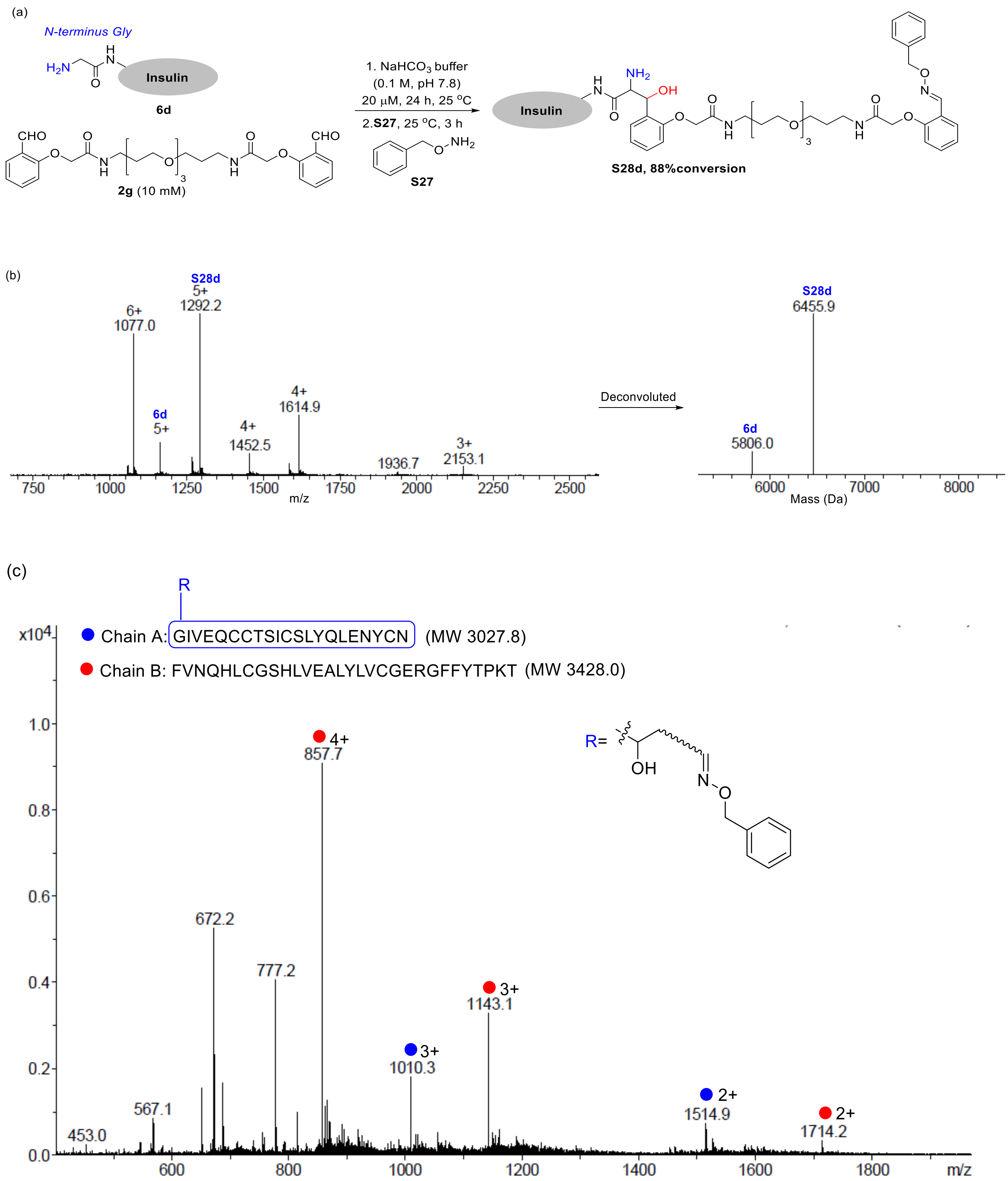

Supplementary Figure 54. Single-site installation of insulin with benzyloxyamine **S27**. **(a)** N-terminus Gly labeling of insulin with reagent **2g**. **(b)** ESI-MS spectra for insulin **6d** (1 equiv.) and aldehyde **2g** (500 equiv.), mono-labeled insulin **S28d** after oxime formation. **(c)** Peptide mapping: MS spectra of mono labled insulin **S28d** after DTT reduction.

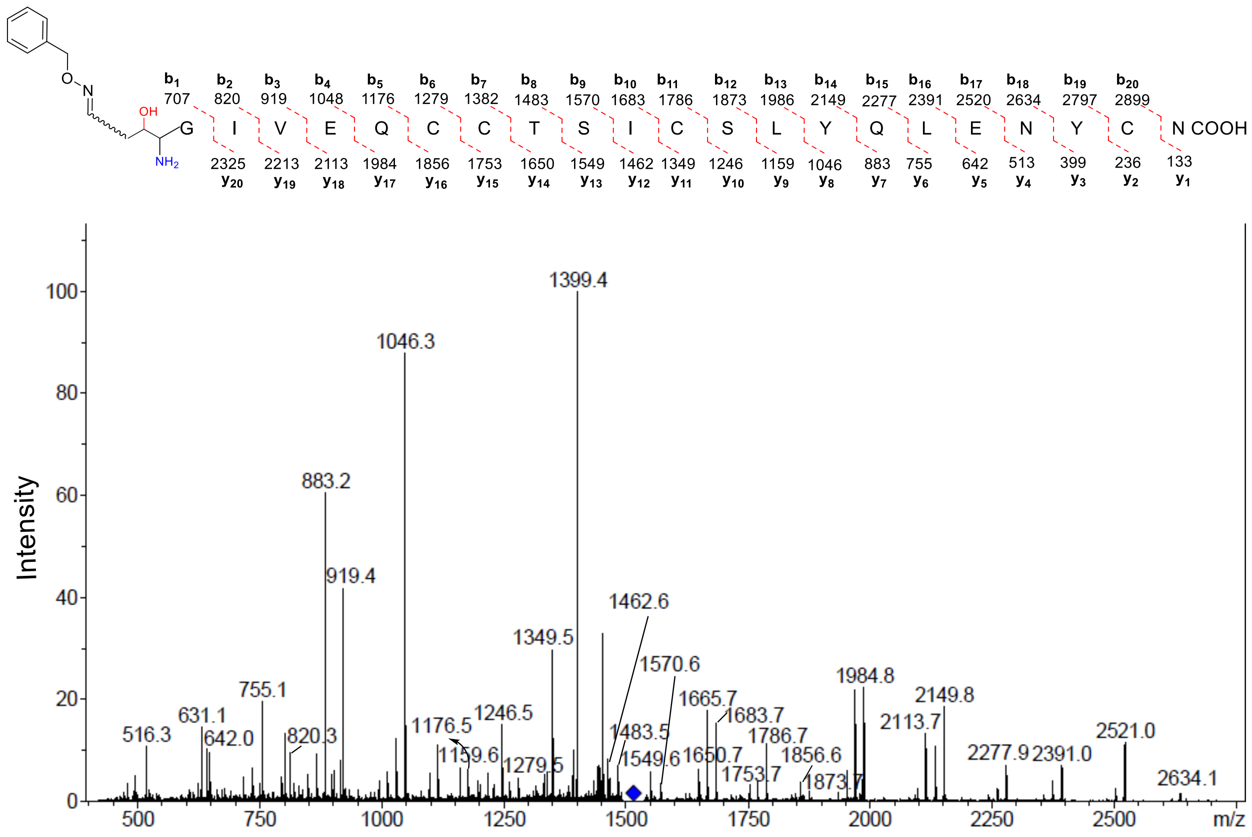

Supplementary Figure 55. Single-site installation of insulin with benzyloxyamine S27. MS-MS spectrum of label GIVEQCCTSICSLYQLENYCN (G1-N21). Site of modification is N-terminus glycine (G1, chain A) in labeled insulin **S28d**.

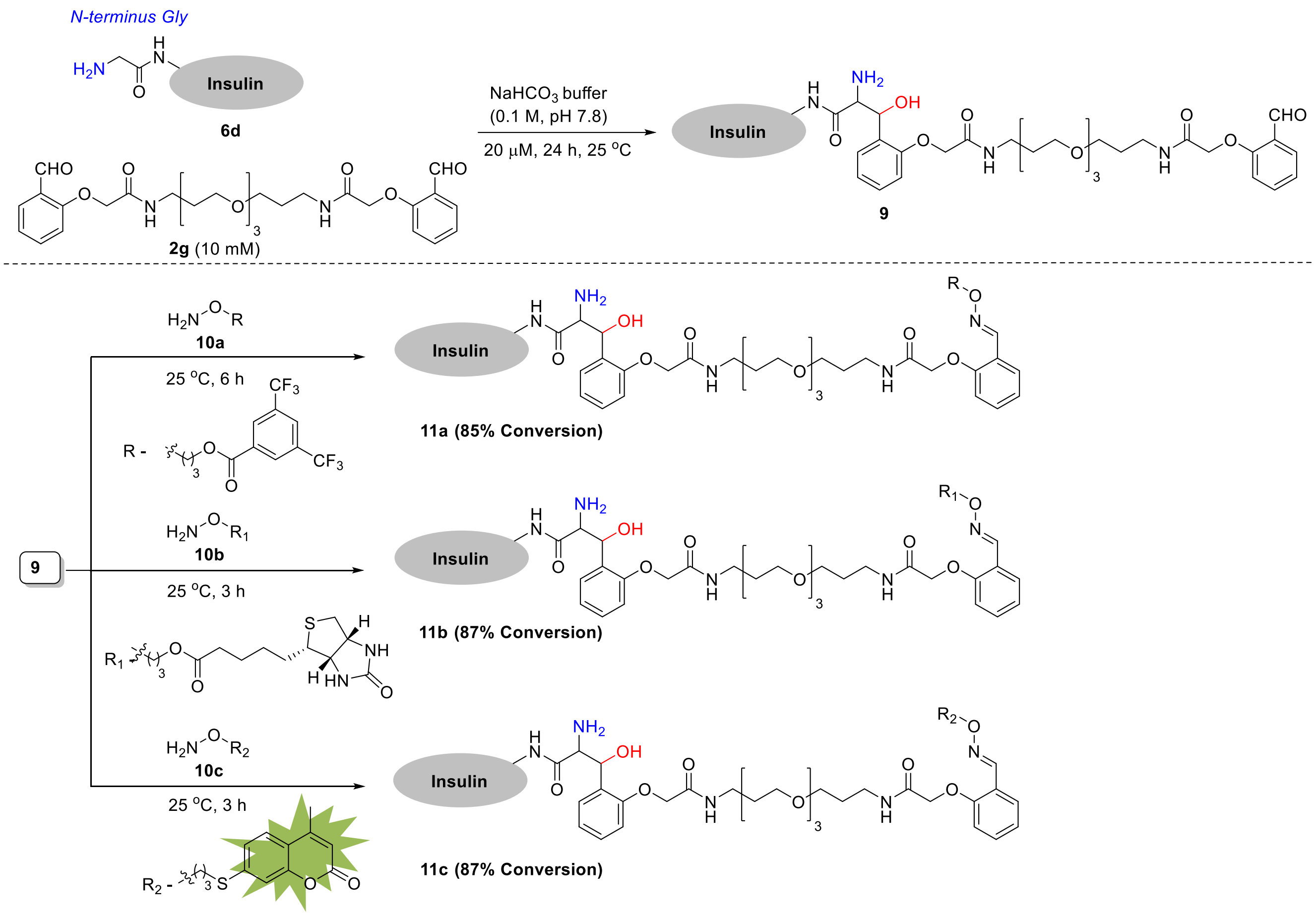

Supplementary Figure 56. Single-site installation of tags. Late stage bio-orthogonal reactions allow installation of an NMR tag, affinity tag, and a fluorophore. The protein **6d** (20 µM) is vortexed with reagent **2g** (10 mM) for 24 h. Subsequently, unreacted reagent **2g** was removed from the reaction mixture and **9** was vortexed with derivatives of O-hydroxylamine (**10a-10c**, 10 mM) for 3-6 h.

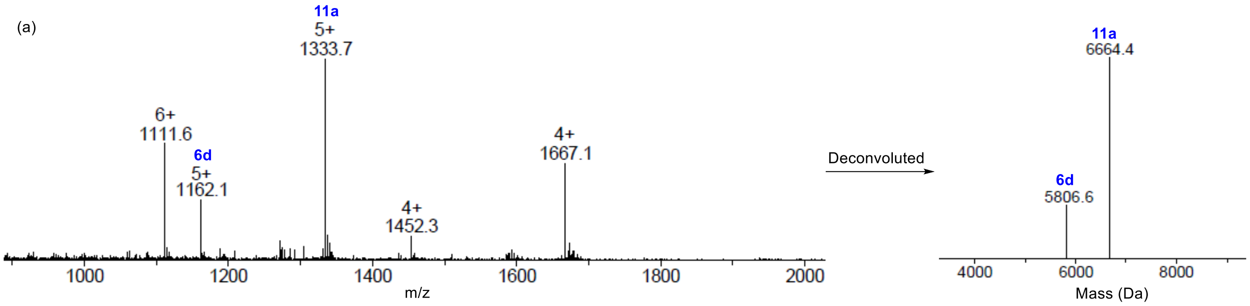

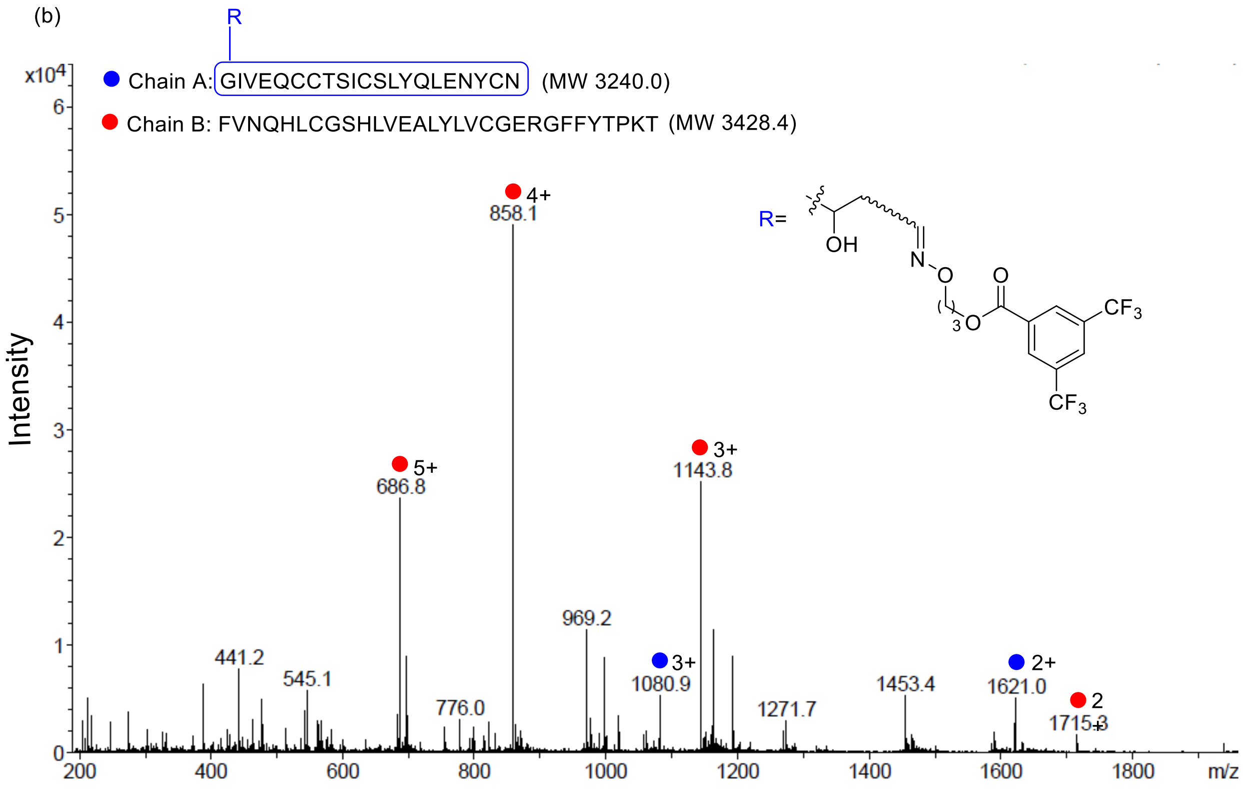

Supplementary Figure 57. Single-site installation of insulin with 10a. **(a)** ESI-MS spectra for insulin **6d** (1 equiv.) and aldehyde **2g** (500 equiv.), mono-labeled insulin **11a** after oxime formation. **(b)** Peptide mapping of ^{19}F-NMR probe tagged insulin **11a** after DTT reduction; ^{19}F-NMR probe tag is presented in chain A of labeled insulin **11a**.

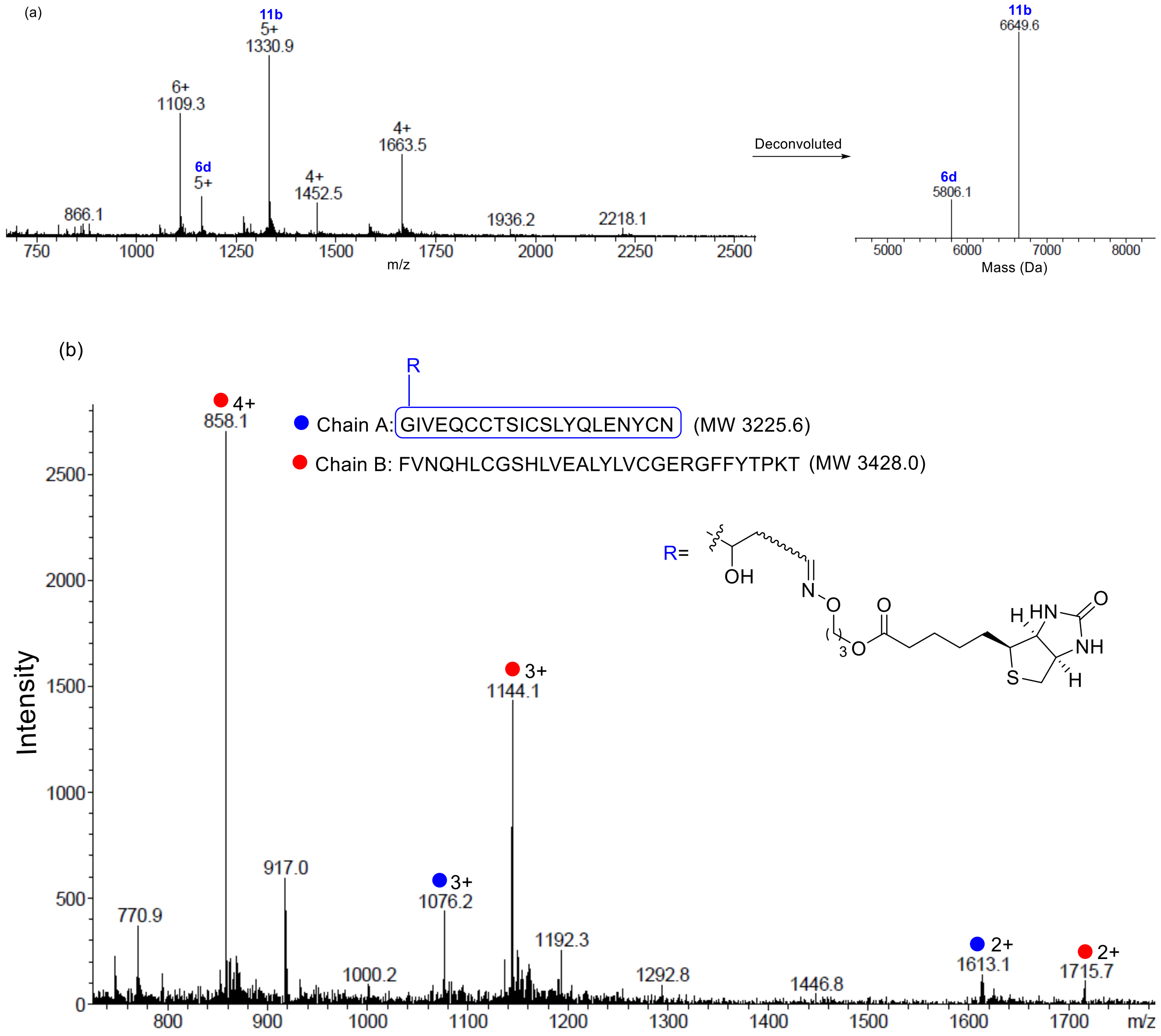

Supplementary Figure 58. Single-site installation of insulin with **10b**. **(a)** ESI-MS spectra for insulin **6d** (1 equiv.) and aldehyde **2g** (500 equiv.), mono-labeled insulin **11b** after oxime formation. **(b)** Peptide mapping of biotin tagged insulin **11b** after DTT reduction; biotin tag is presented in chain A of labeled insulin **11b**.

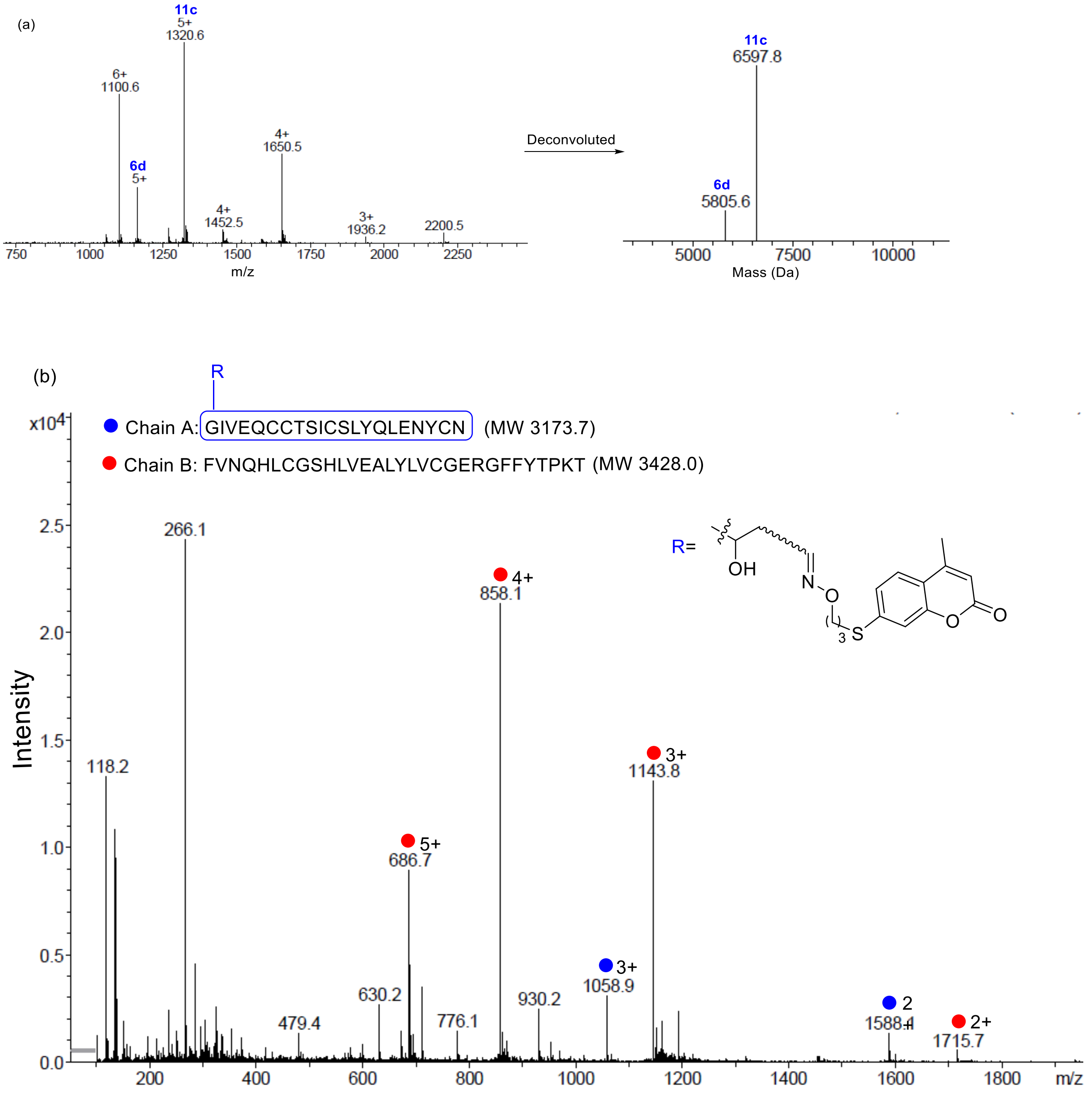

Supplementary Figure 59. Single-site installation of insulin with 10c. **(a)** ESI-MS spectra for insulin **6d** (1 equiv.) and aldehyde **2g** (500 equiv.), mono-labeled insulin **11c** after oxime formation. **(b)** Peptide mapping of coumarin tagged insulin **11c** after DTT reduction; coumarin tag is presented in chain A of labeled insulin **11c**.

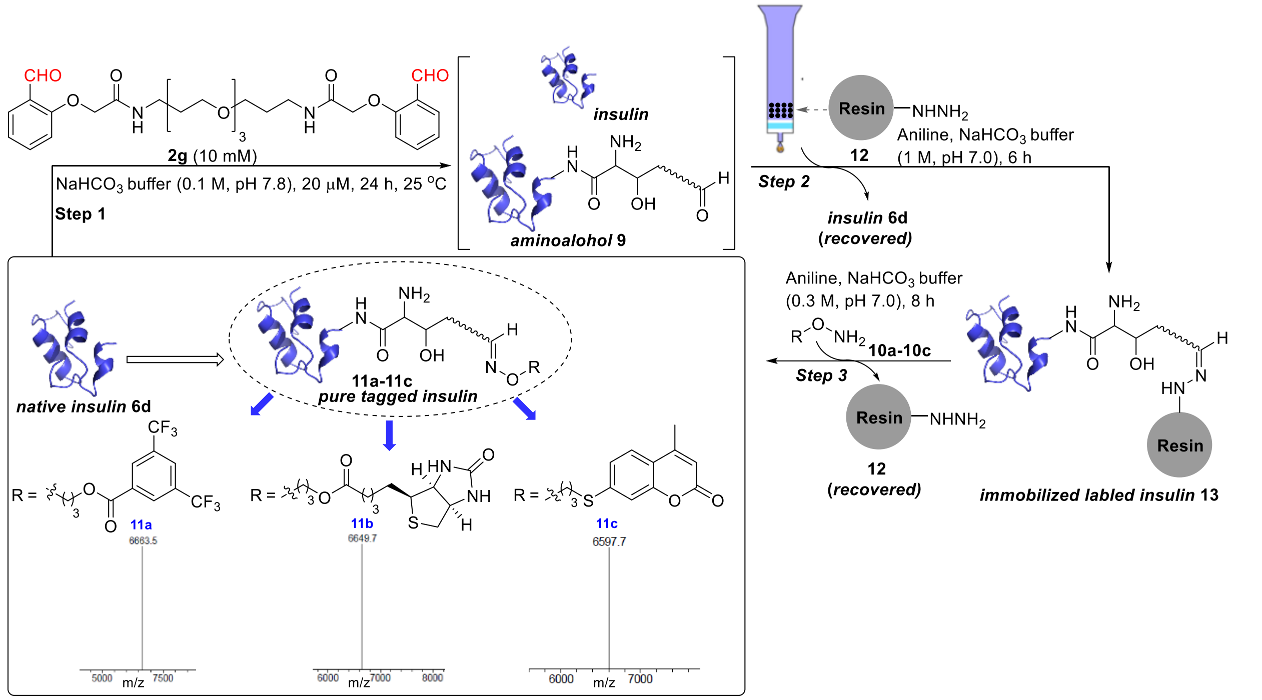

Supplementary Figure 60. Purification of single-site tagged insulin from the reaction mixture. Step 1: Aminoalcohol (**9**) formation. Step 2: Immobilization of the labeled insulin **9** on hydrazide resin. Step 3: Transoximization of **13** with derivatives of O-hydroxylamine (**10a-10c**) releases the tagged labeled protein (**11a-11c**) in analytically pure.

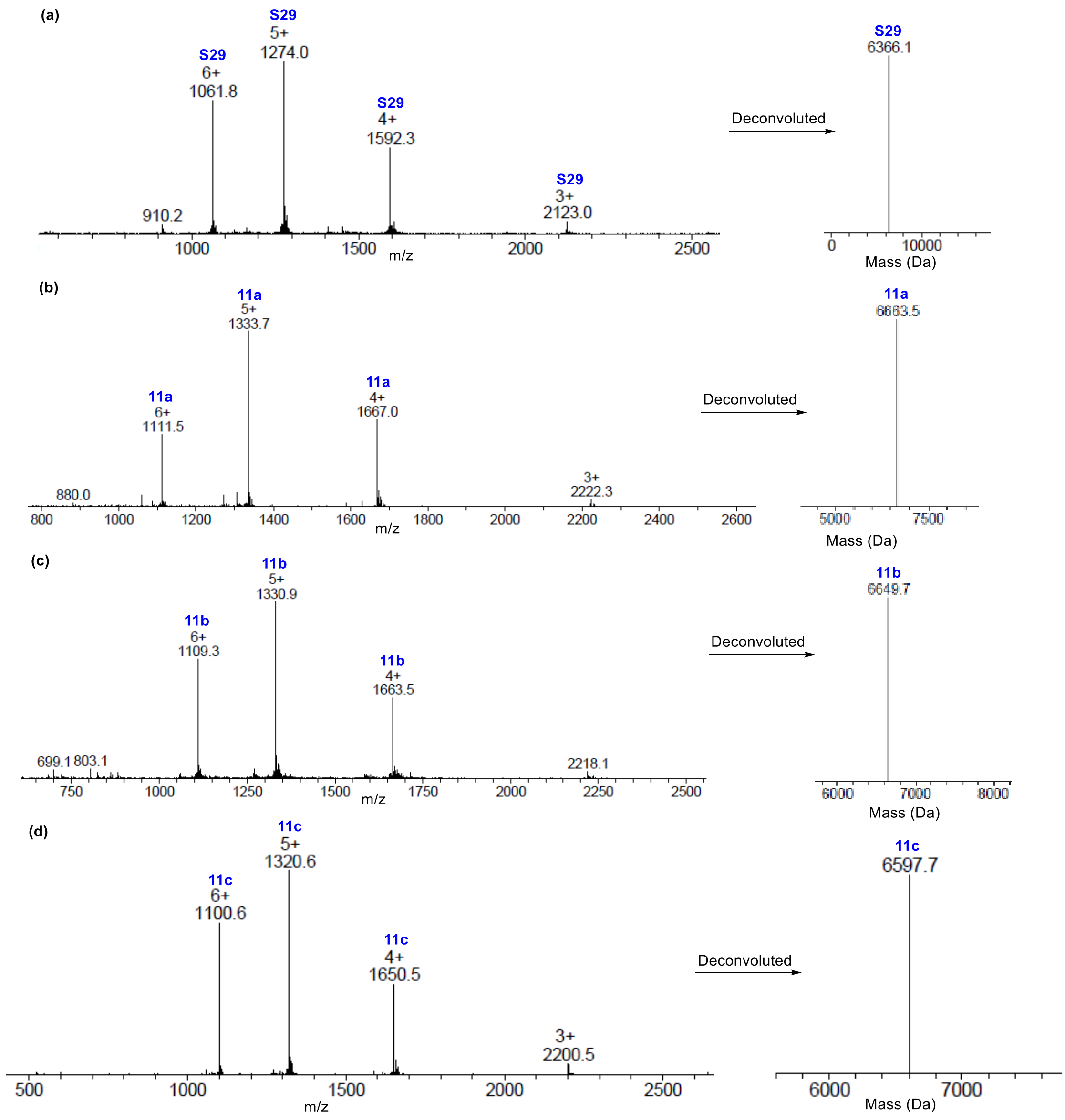

Supplementary Figure 61. Purification of the labeled protein with tagging. **(a)** ESI-MS spectra of purified insulin tagged with O-hydroxylamine **S29** **(b)** ^{19}F NMR probe tagged insulin **11a**, **(c)** biotin tagged insulin **11b**, and **(d)** coumarin tagged insulin **11c**.

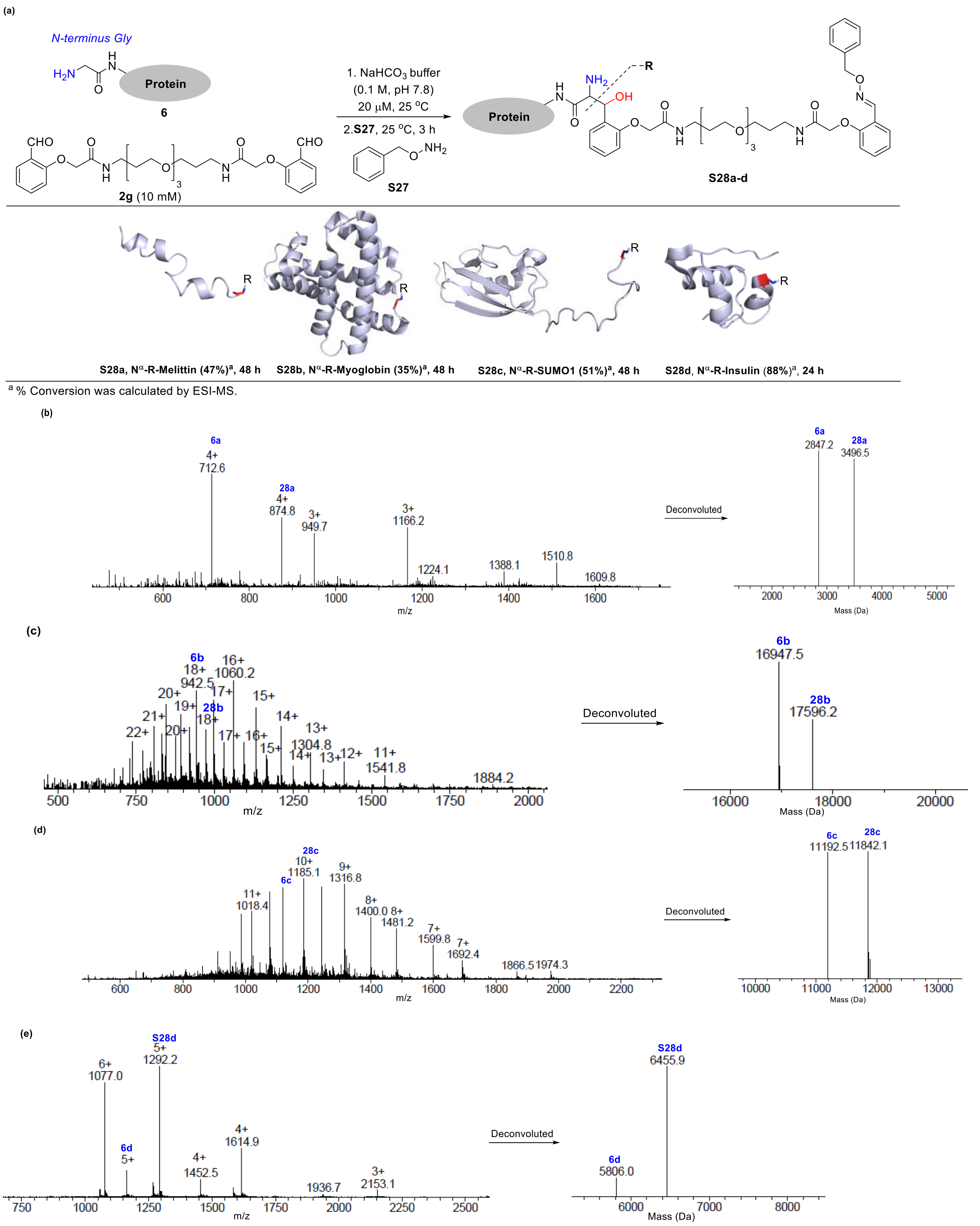

Supplementary Figure 62. Single-site labeling of N-terminus Gly proteins. **(a)** N-terminus Gly labeling of proteins (**6a-6d**) with **2g. (b)** ESI-MS spectrum of labeled of melittin **S28a. (c)** ESI-MS spectrum of labeled myoglobin **S28b. (d)** ESI-MS spectrum of labeled SUMO1 **S28c. (e)** ESI-MS spectrum of labeled Insulin **S28d.**

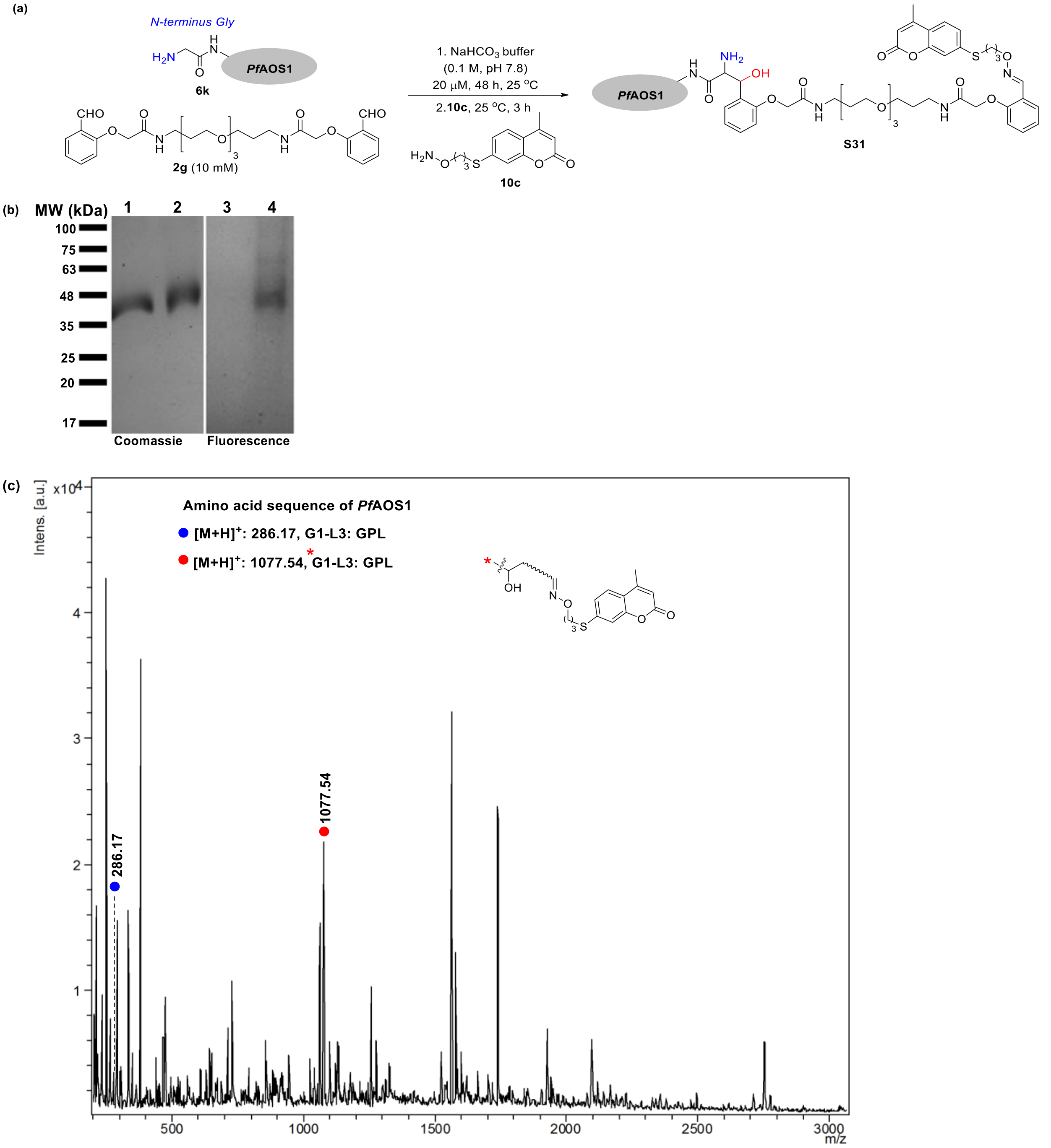

Supplementary Figure 63. Single-site labeling of N-terminus Gly *Pf*AOS1 6k. **(a)** N-terminus Gly labeling of *Pf*AOS1 **6k** with **2g**. **(b)** 12% SDS-PAGE of *Pf*AOS1 (Plasmodium falciparum AOS1) **6k** and coumarin-tagged *Pf*AOS1 **S31** followed by Coomassie staining and fluorescence imaging. MW - Molecular Weight, SDS-PAGE: lane 1 - *Pf*AOS1 **6k**, lane 2 - coumarin-tagged *Pf*AOS1 **S31**, lane 3 - *Pf*AOS1 **6k** and lane 4 - coumarin-tagged *Pf*AOS1 **S31**. The band at ~40 kDa corresponds to the *Pf*AOS1 **6k**. The band ~40 kDa shown by fluorescence imaging confirms the labeling of *Pf*AOS1. **(c)** Peptide mapping: MS spectra of digest of N-terminus Gly labeling of *Pf*AOS1 **S31**. Source data of Figure 63 panel **b** provided in a Source Data file

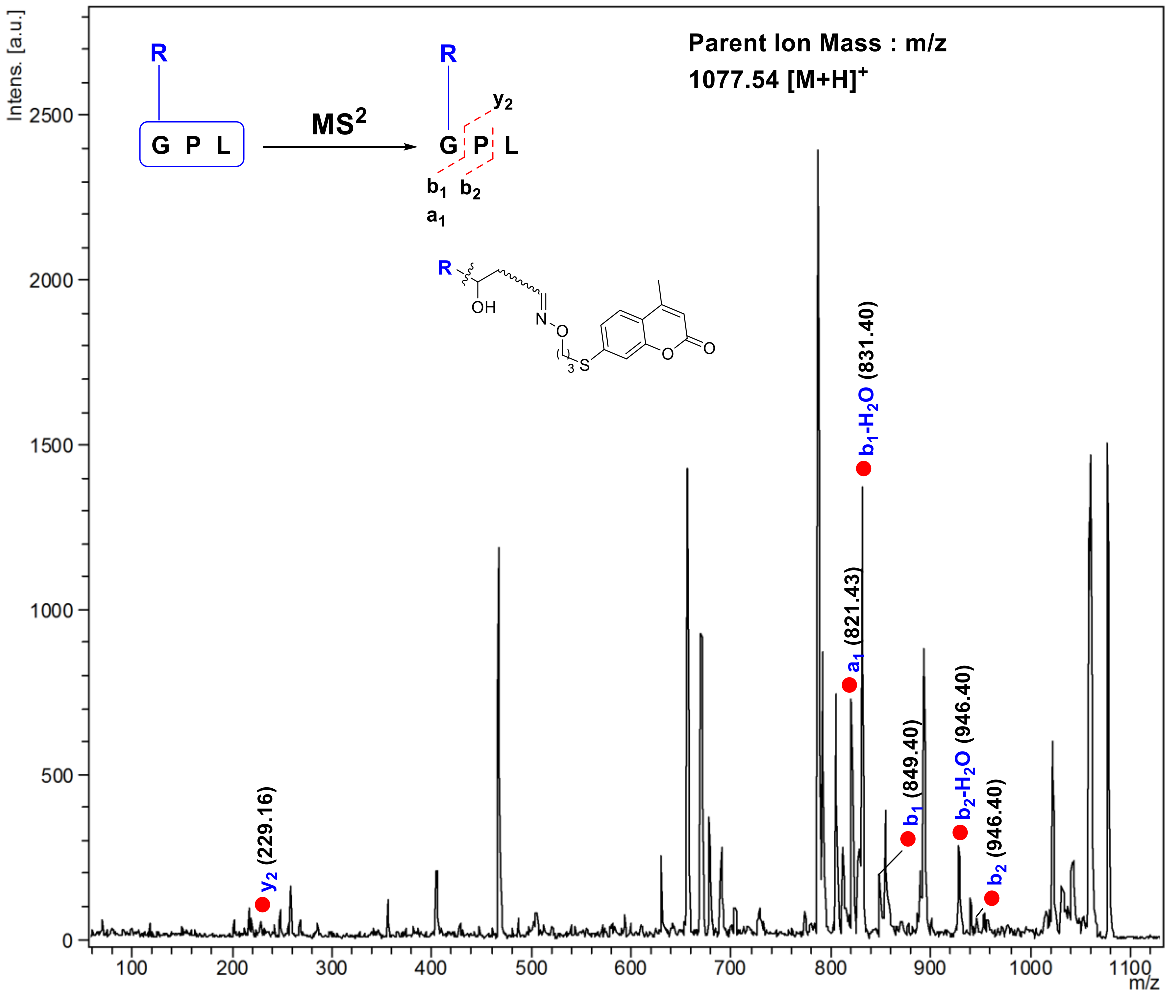

Supplementary Figure 64. Single-site labeling of N-terminus Gly *Pf*AOS1 6k. MS-MS spectrum of labeled GPL (G1-L3). Site of modification is N-terminus glycine (G1) in labeled PfAOS1 **S31**.

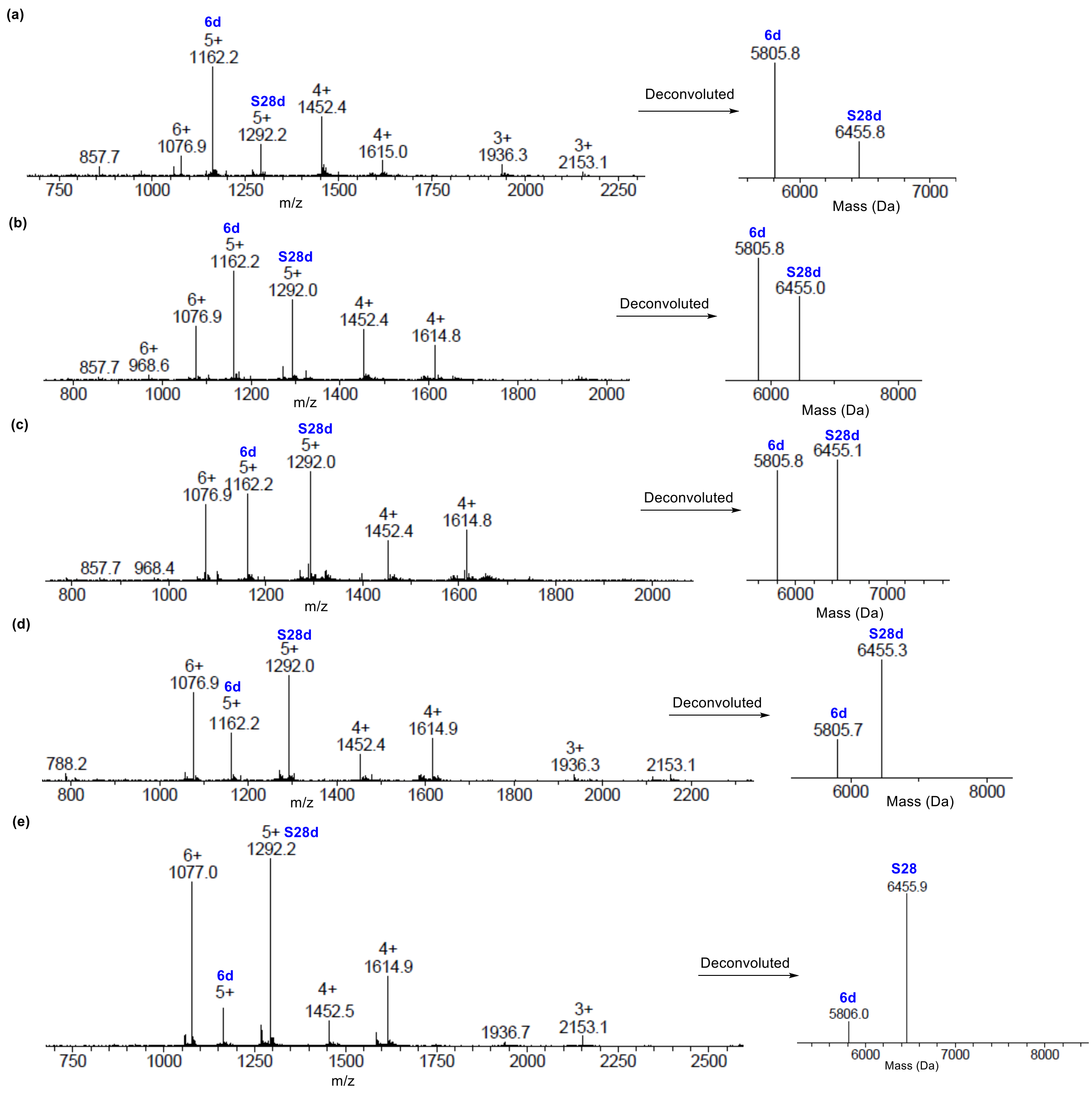

Supplementary Figure 65. Optimization of the stoichiometry of reagent 2g. **(a)** ESI-MS spectra of labeled insulin with 10 equiv. of reagent **2g.** **(b)** ESI-MS spectra of labeled insulin with 50 equiv. of reagent **2g.** **(c)** ESI-MS spectra of labeled insulin with 100 equiv. of reagent **2g.** **(d)** ESI-MS spectra of labeled insulin with 300 equiv. of reagent **2g.** **(e)** ESI-MS spectra of labeled insulin with 500 equiv. of reagent **2g.**

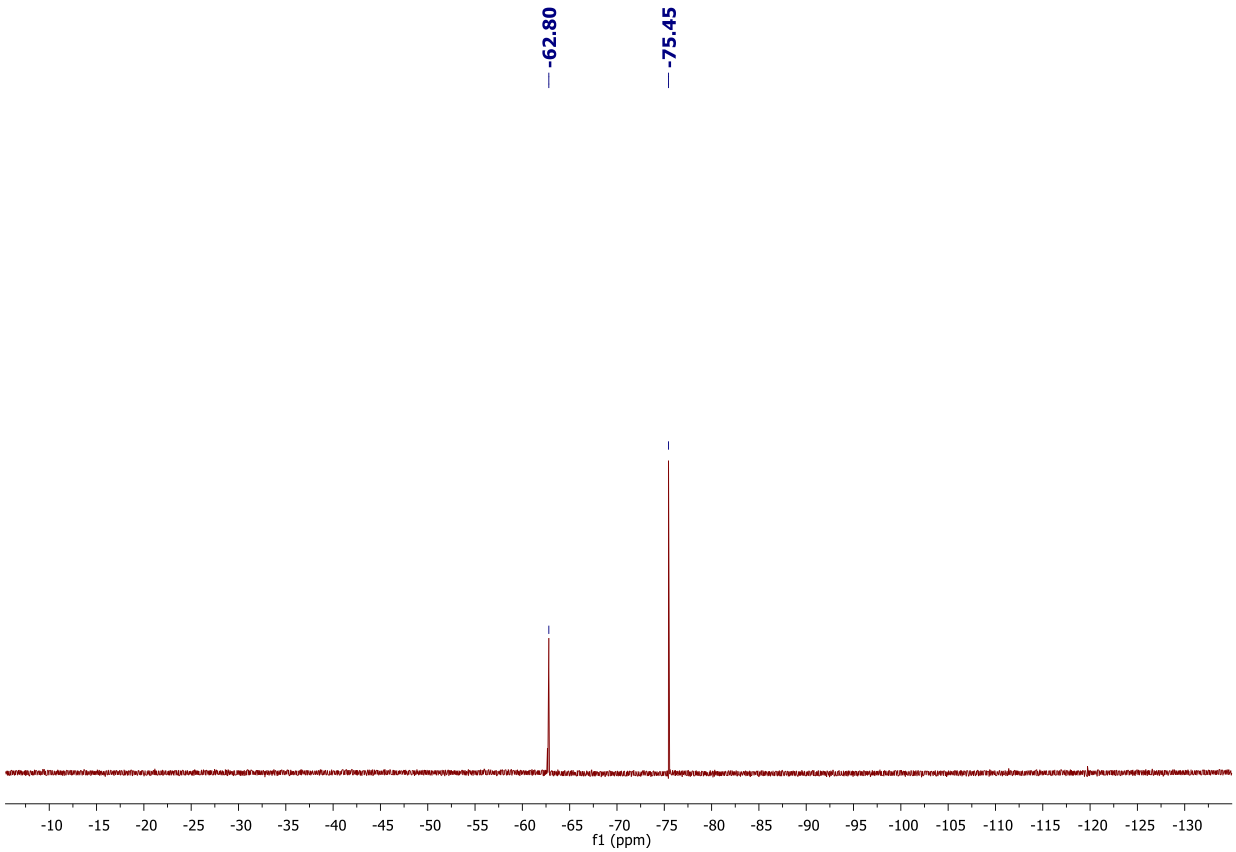

Supplementary Figure 66. ^{19}F NMR data of NMR probe tagged Insulin 11a. ^{19}F-NMR probe attached Insulin (**11a**) shows a sharp signal at -62.80 ppm by ^{19}F-NMR spectroscopy. TFA (0.2 mM) was used as internal standard, -75.45 ppm). Sample was recorded in phosphate buffer (0.1 M, pH 7.0): D_2O (9:1).

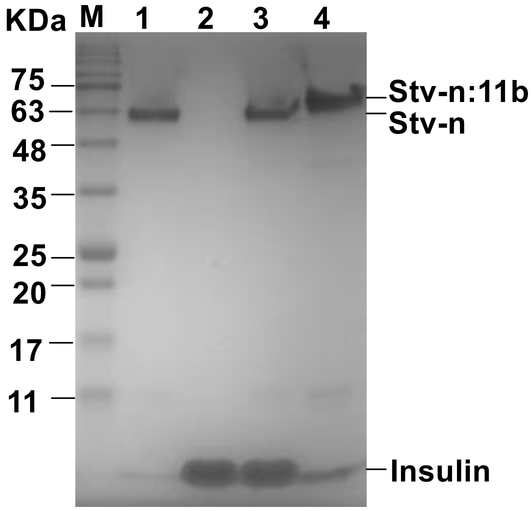

Supplementary Figure 67. SDS-PAGE of labeled insulin **11b**. Detection of biotin tagged Insulin by SDS-PAGE and Streptavidin (Stv-n) induced band-shift analysis. (For ESI-MS data, see Supplementary Figure 58). 12% SDS-PAGE followed by Coomassie staining identifies the biotin tagged Insulin **11b**. The complex (lane 4) of Streptavidin (Stv-n) and **11b** appeared as a single band (~70 kDa). MW - Molecular Weight, SDS-PAGE: lane 1 - native Streptavidin (Stv-n), lane 2 - native Insulin (**6d**), lane 3 - Stv-n and **6d** and lane 4 - Stv-n and **11b**. The incubation of biotin tagged Insulin **11b** and Stv-n (1:4) in sample buffer were done at 25 ^{0}C for 15 minutes before subjecting to SDS-PAGE. In the similar way, control sample (lane 3) was also performed. 2 × SDS sample buffer (non-reducing): 100 mM Tris–HCl, pH 6.8, 4% SDS, 20% glycerol, 0.1% bromophenol blue). Source data of Figure 67 provided in a Source Data file

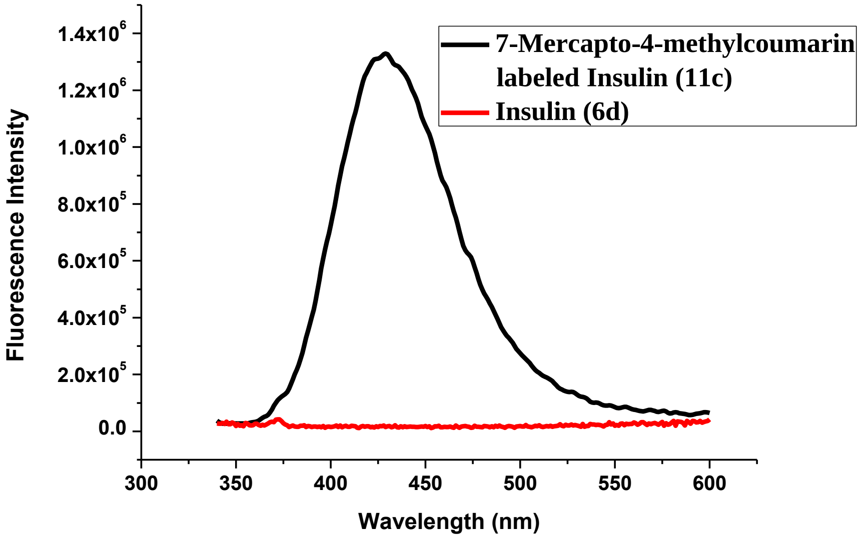

Supplementary Figure 68. Fluorescence data of coumarin tagged insulin 11c. Steady-state fluorescence spectra of Insulin (**6d**) and coumarin tagged Insulin (**11c**). In phosphate buffer (0.1 M, pH 7.0), **11c** exhibits absorption and emission band peaked at 336 nm and 428 nm, respectively. Source data of Figure 68 provided in a Source Data file

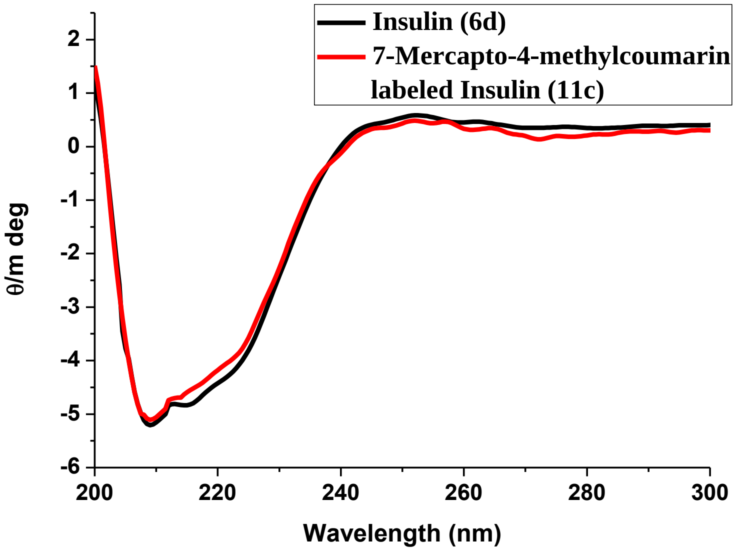

Supplementary Figure 69. CD data of tagged insulin 11c. Effect of labeling on the structure of proteins. Circular dichroism (CD) spectra of (a) Insulin **6d** (black line), labeled insulin **11c** (red line) in phosphate buffer (0.1 M, pH 7.0) at concentration 0.1 mg/ml. The concentration of labeled protein is kept constant with respect to the native protein in each case. Source data of Figure 69 provided in a Source Data file

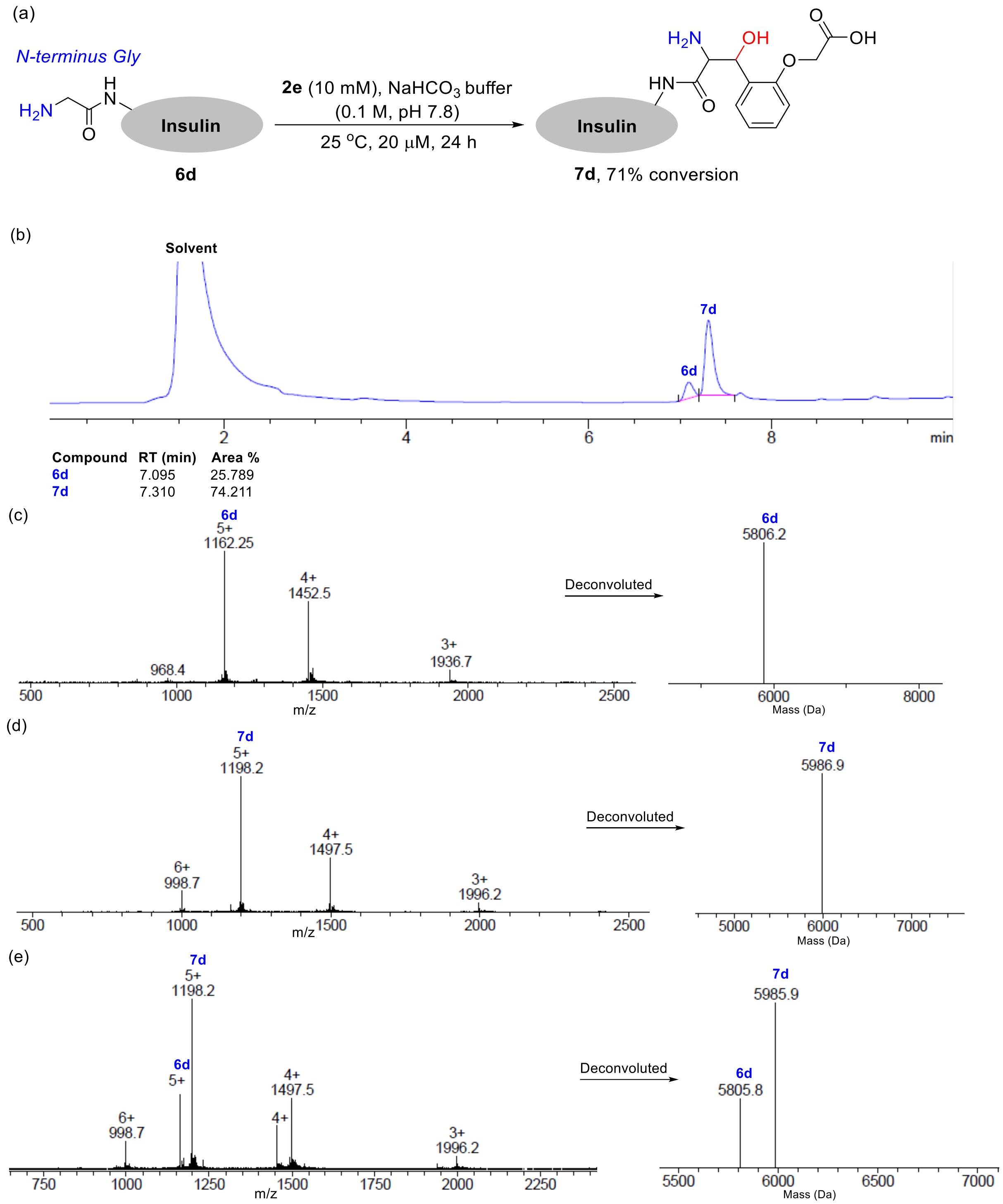

Supplementary Figure 70. Method development for estimation of % conversion. The example includes single-site labeling of insulin (**7d**, Supplementary Figure 52). **(a)** N-terminus Gly labeling of insulin with reagent **2e**. **(b)** HPLC spectrum for mono-labeled insulin **7d.** **(c)** ESI-MS spectrum for insulin **6d** (LC peak at 7.1 min). **(d)** ESI-MS spectrum for mono-labeled insulin **7d** (LC peak at 7.3 min). **(e)** ESI-MS spectrum after overall integration of **6d** and **7d** (combined LC peaks at 7.1 and 7.3 min).

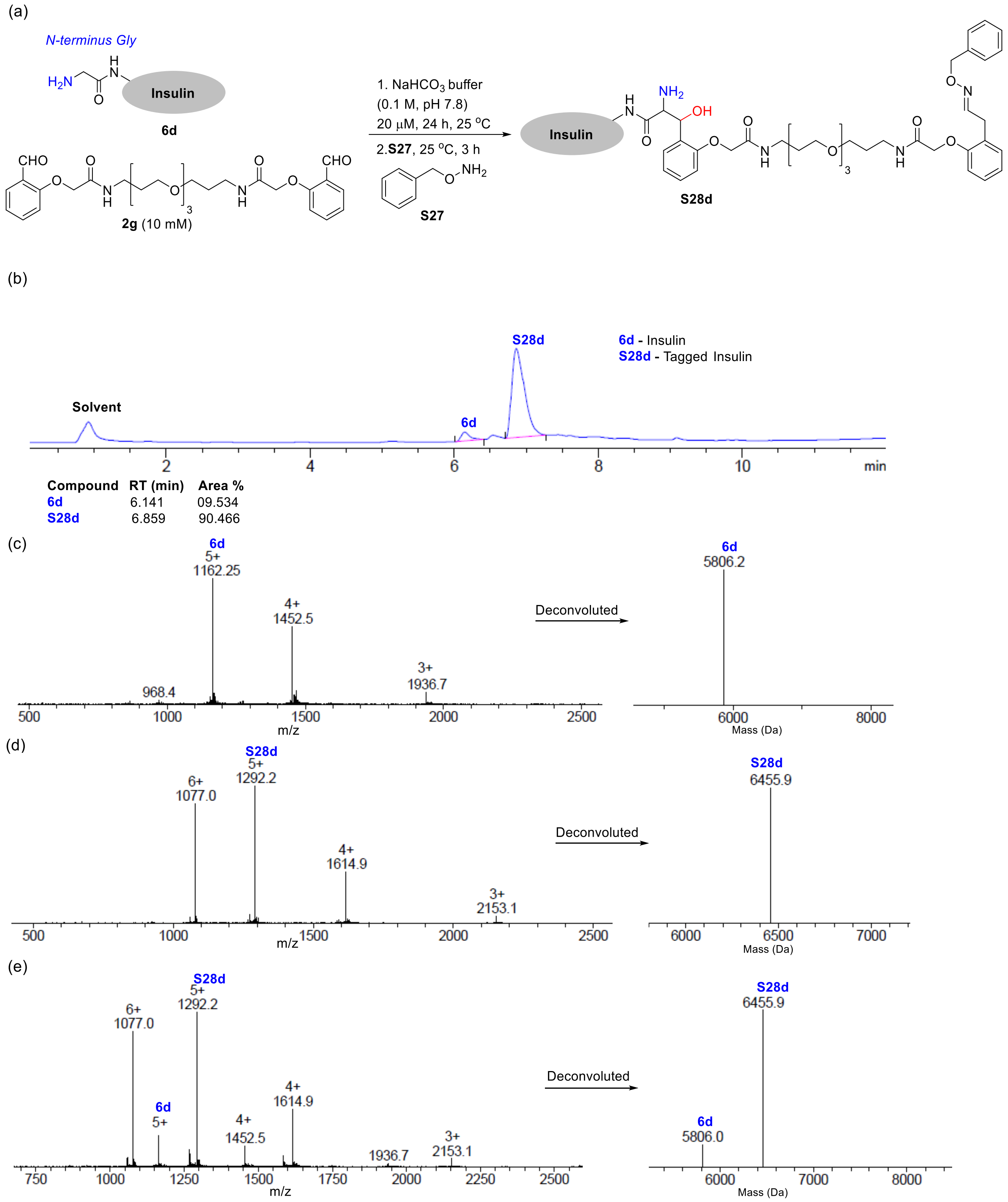

Supplementary Figure 71. Method development for estimation of % conversion. The example includes single-site labeling of insulin (**S28d**, Supplementary Figure 54). **(a)** N-terminus Gly labeling of insulin with reagent **2g**. **(b)** HPLC spectrum for mono-labeled insulin **S28d.** **(c)** ESI-MS spectrum for insulin **6d** (LC

peak at 6.1 min). **(d)** ESI-MS spectrum for mono-labeled insulin **S28d** (LC peak at 6.9 min). **(e)** ESI-MS spectrum after overall integration of **6d** and **S28d** (combined LC peaks at 6.1 and 6.9 min).

(a)

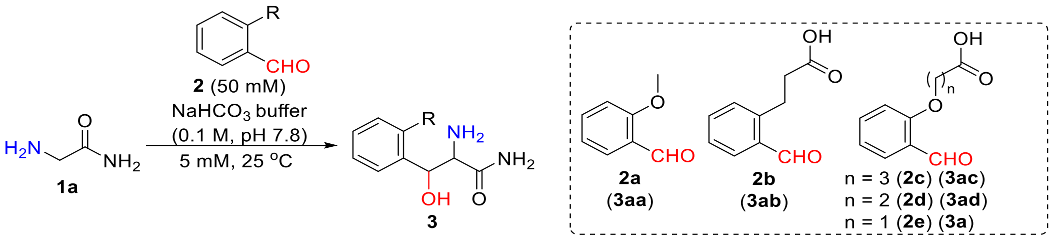

(b)

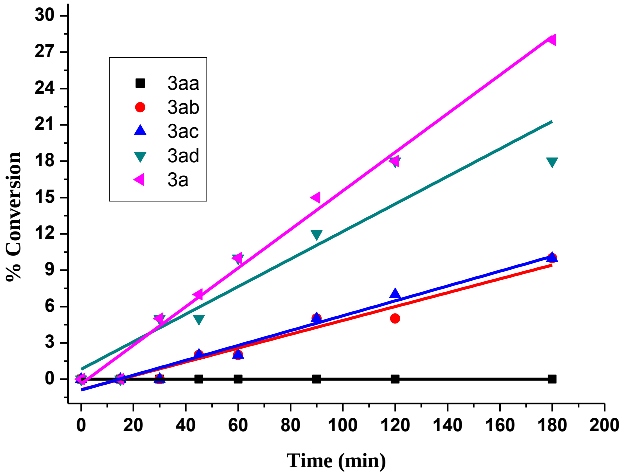

Supplementary Figure 72. Initial rates of aminoalcohol formation. **(a)** Reaction of glycinamide with different reagents (**2a, 2b, 2c, 2d, 2e**). **(b)** Conversion of **1a** to aminoalcohol (**3aa**, **3ab**, **3ac**, **3ad**, and **3a**) with time. Source data of Figure 72 provided in a Source Data file

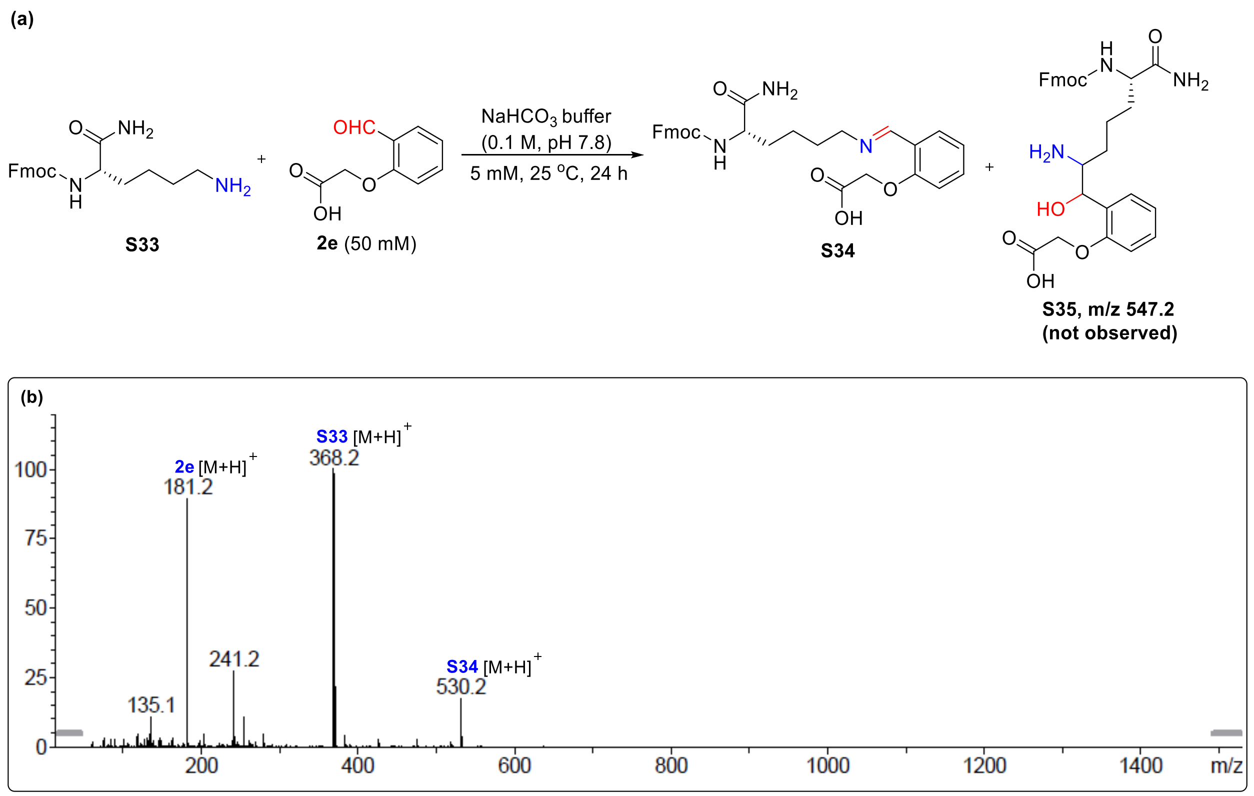

Supplementary Figure 73. Rationale for selectivity between N^{α}-NH_2 (N-terminus) and N^{ε}-NH_2 (Lys). **(a)** Reaction of Fmoc-Lys-NH_2 with reagent 2e. **(b)** ESI-MS spectrum of **S34**.

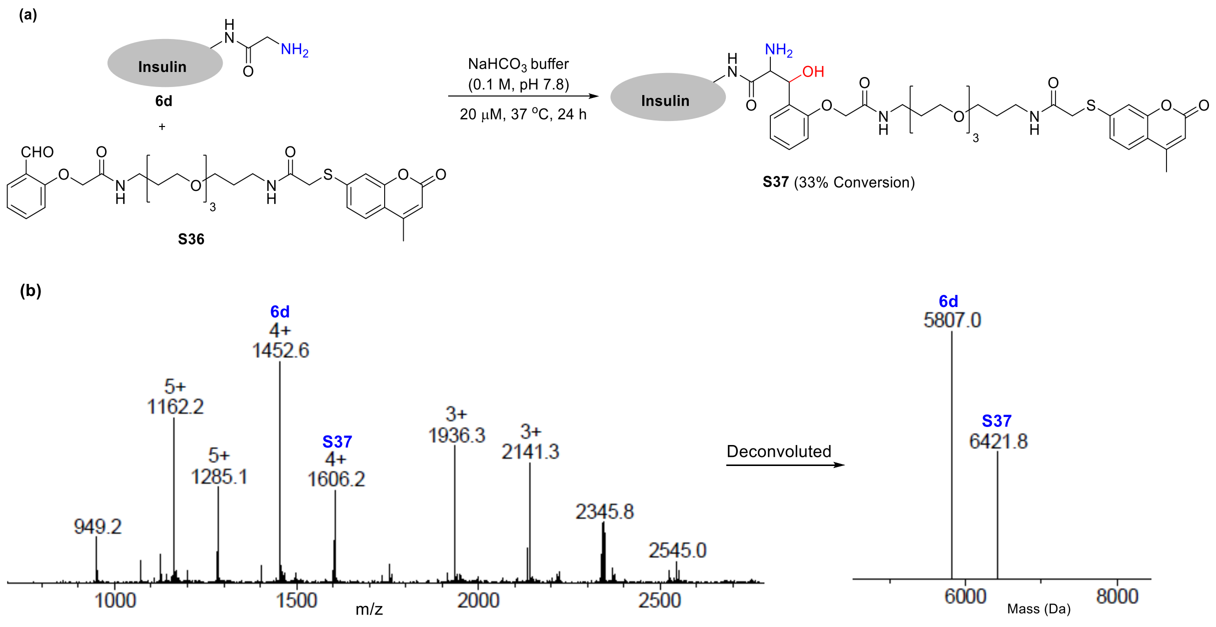

Supplementary Figure 74. N-terminus Gly labeling of insulin. **(a)** Single-step installation of the probe of interest. **(b)** ESI-MS spectrum for mono-labeled insulin **S37**.

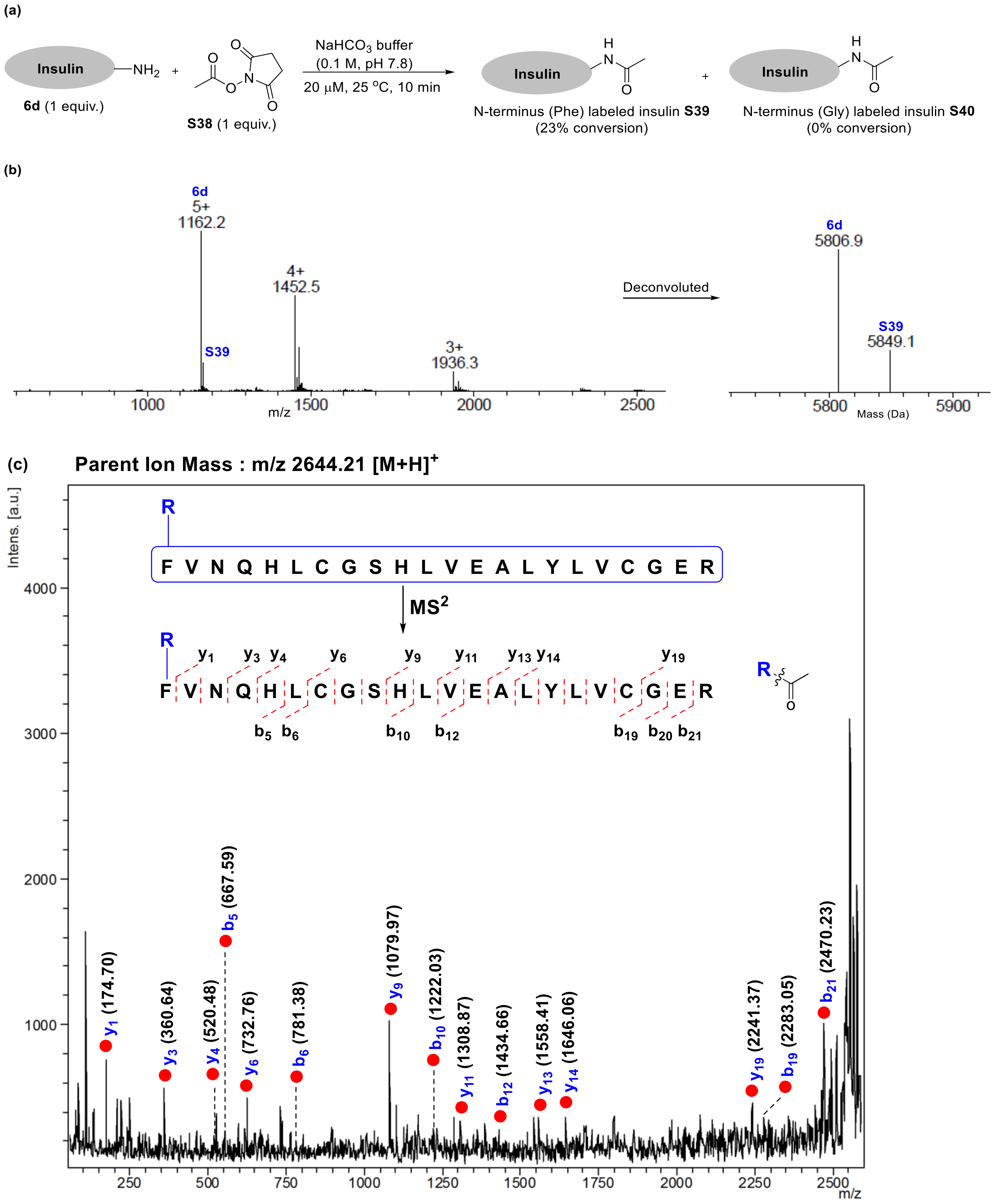

Supplementary Figure 75. N-terminus (Phe) labeling of insulin. **(a)** Labeling of insulin with N-hydroxy succinamide ester. **(b)** ESI-MS spectrum for mono-labeled insulin **S39**. **(b)** MS-MS spectrum of labeled FVNQHLCGSHLVEALYLVCGER (F1-R22). Site of modification is N-terminus (Phe) in labeled insulin **S39**.

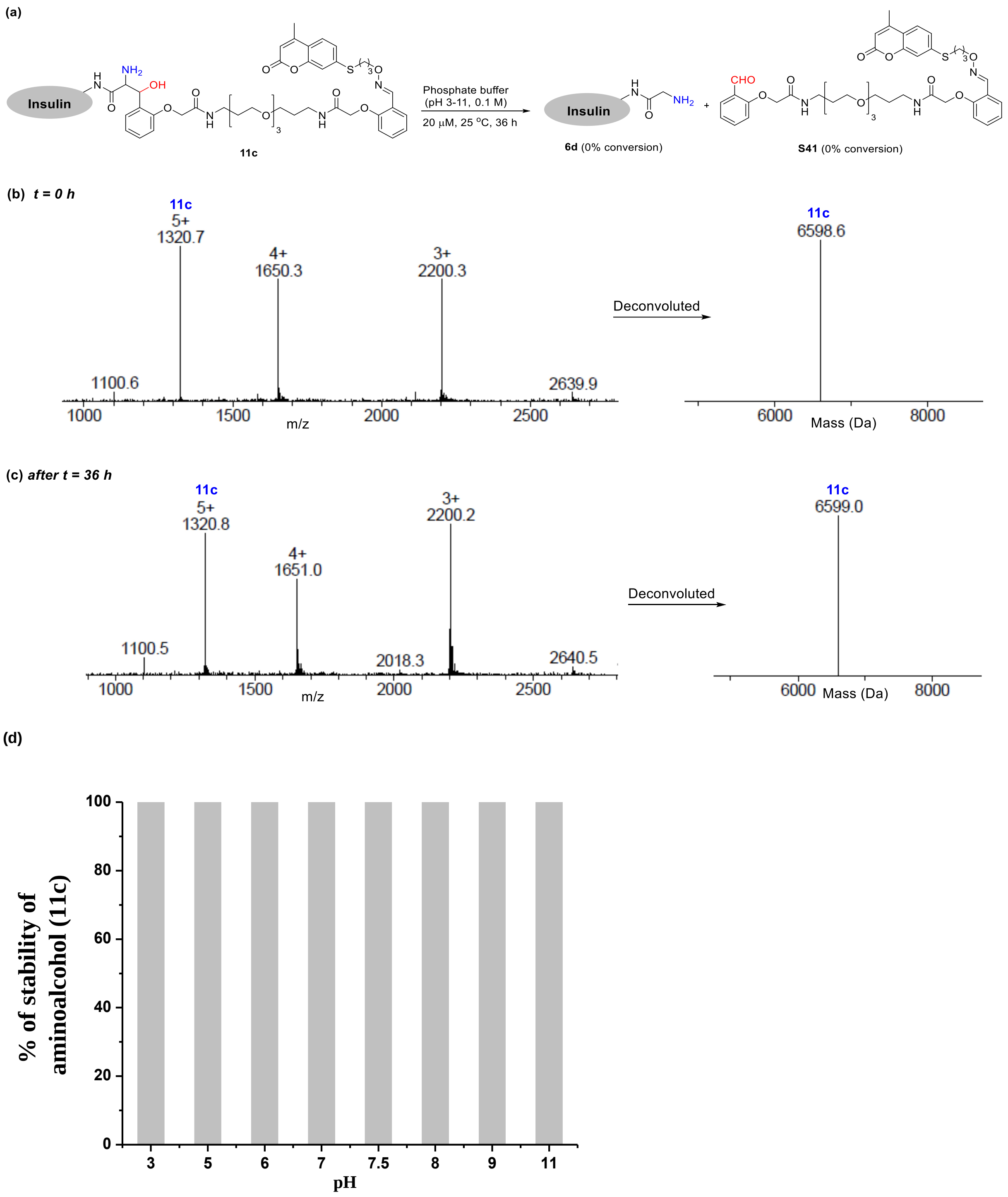

Supplementary Figure 76. Stability of tagged protein. **(a)** Coumarin tagged insulin **11c** (20 μM) was incubated at 25 °C in 100 mM phosphate buffer at pH 3-11. ESI-MS data of **11c** at: **(b)** 0 h, and **(c)** 36 h. **(d)** Stability of **11c** at pH 3-11 after 36 h (grey bars). The conversions are estimated by ESI-MS and the data points are normalized as per the modification at t = 0 h.

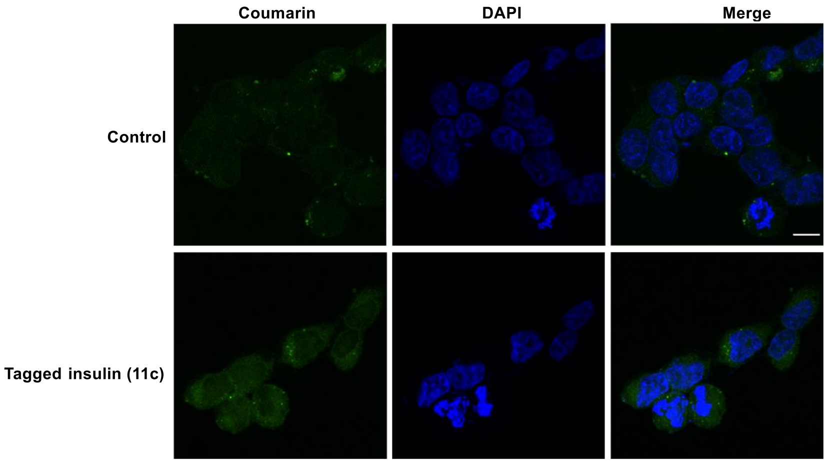

Supplementary Figure 77. Insulin imaging assay. Uptake of tagged insulin (green) in cells. Chromatin (blue) (scale bar:10 µm). It re-emphasizes the results detailed in Figure 5c. In another trial, we examined the activity in cell-based assays using coumarin tagged insulin (**11c**) through its ability to activate insulin receptor (IR) mediated signaling. In the cellular uptake assays, the mock treated cells exhibit poor autofluorescent signals (Supplementary Figure 77, 1st row, first panel) and in contrast, cells treated with **11c** display coumarin signal accumulated throughout the cells (significantly more in the cytoplasm) indicating their regular uptake (Supplementary Figure 77, 2nd row, first panel).

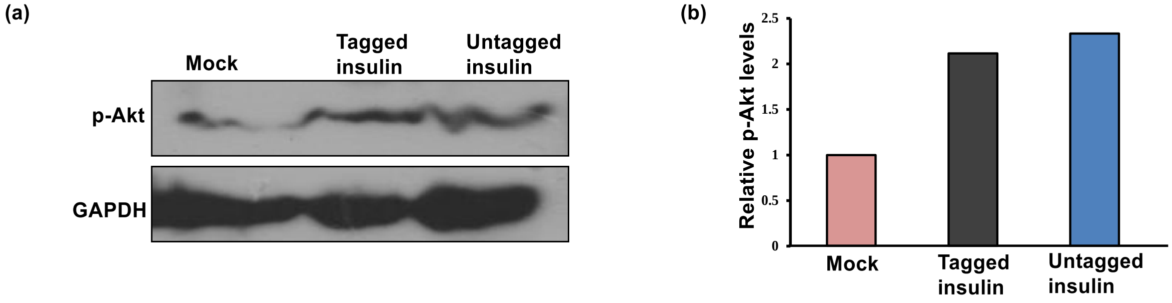

Supplementary Figure 78. Western blot analysis. **(a)** Western blot analysis of pAkt and GAPDH in HEK293T cells lysates. **(b)** Quantification of pAkt signal relative to GAPDH. Source data of Figure 78 panel **a** provided in a Source Data file

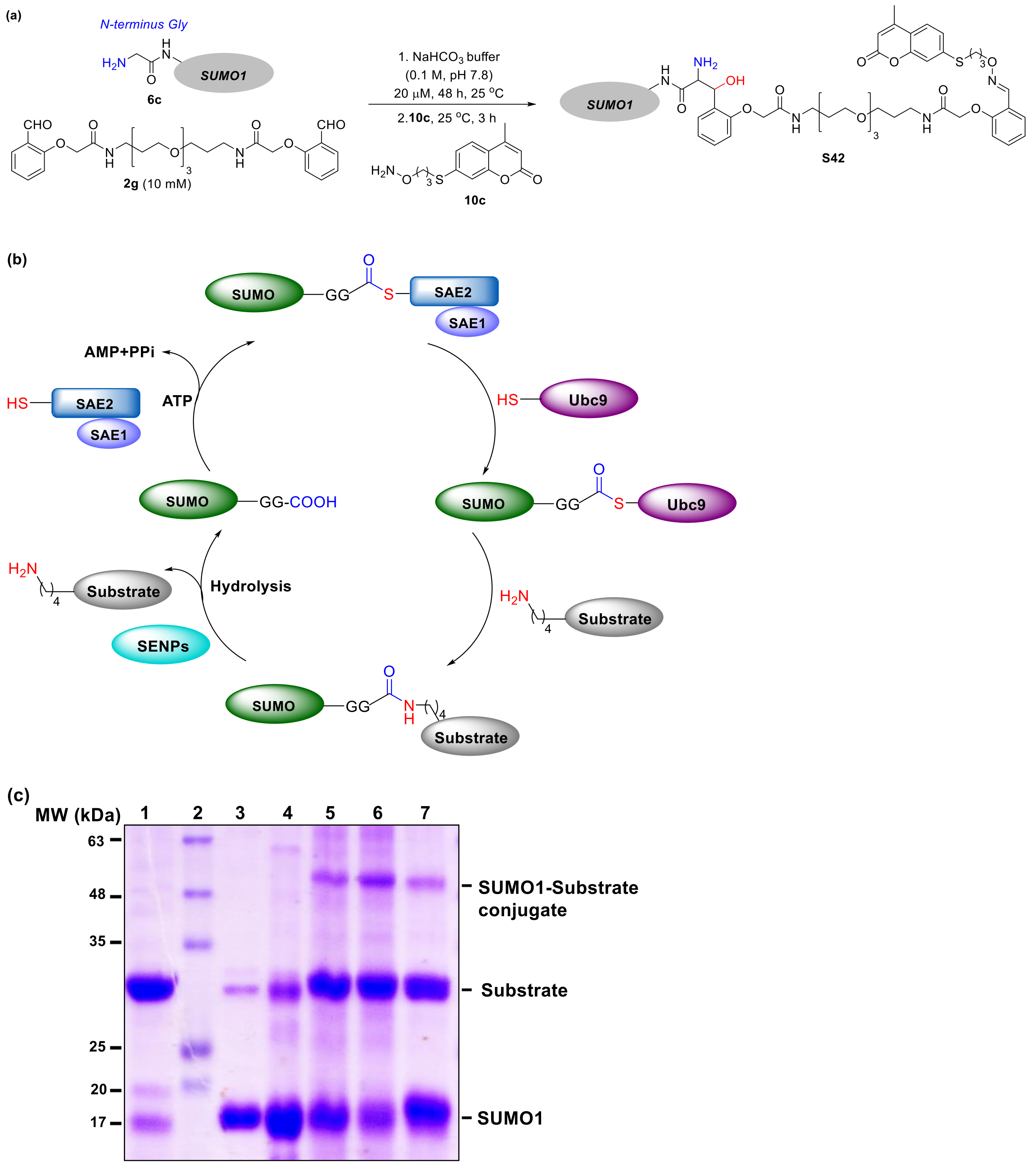

Supplementary Figure 79. Activity analysis of coumarin tagged SUMO1. **(a)** N-terminus Gly tagging of SUMO1 (**6c**). **(b)** Schematic representation of a SUMOylation reaction. **(c)** Activity analysis of coumarin tagged SUMO1 in an *in vitro* SUMOylation reaction. 12% SDS-PAGE of coumarin modified SUMO1 **S42** followed by Coomassie staining. MW- Molecular Weight, SDS-PAGE: lane 1- SUMO1 + substrate (no enzymes), lane 2- Ladder, lane 3- Input native SUMO1, lane 4- Input modified SUMO1, lane 5- SUMOylation with modified SUMO1 (4 h), lane 6- SUMOylation with modified SUMO1 (12 h), lane 7- SUMOylation with native SUMO1 (12 h). Source data of Figure 79 panel **c** provided in a Source Data file

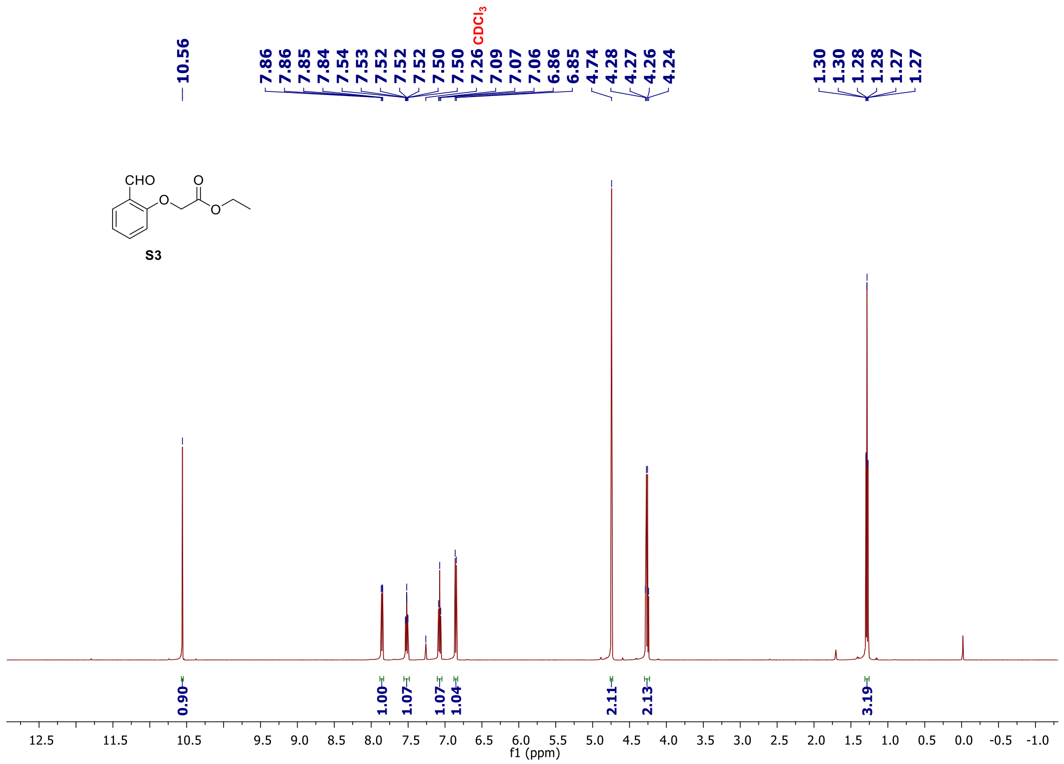

Supplementary Figure 80. ^{1}H NMR spectrum in $CDCl_3$ of compound **S3**

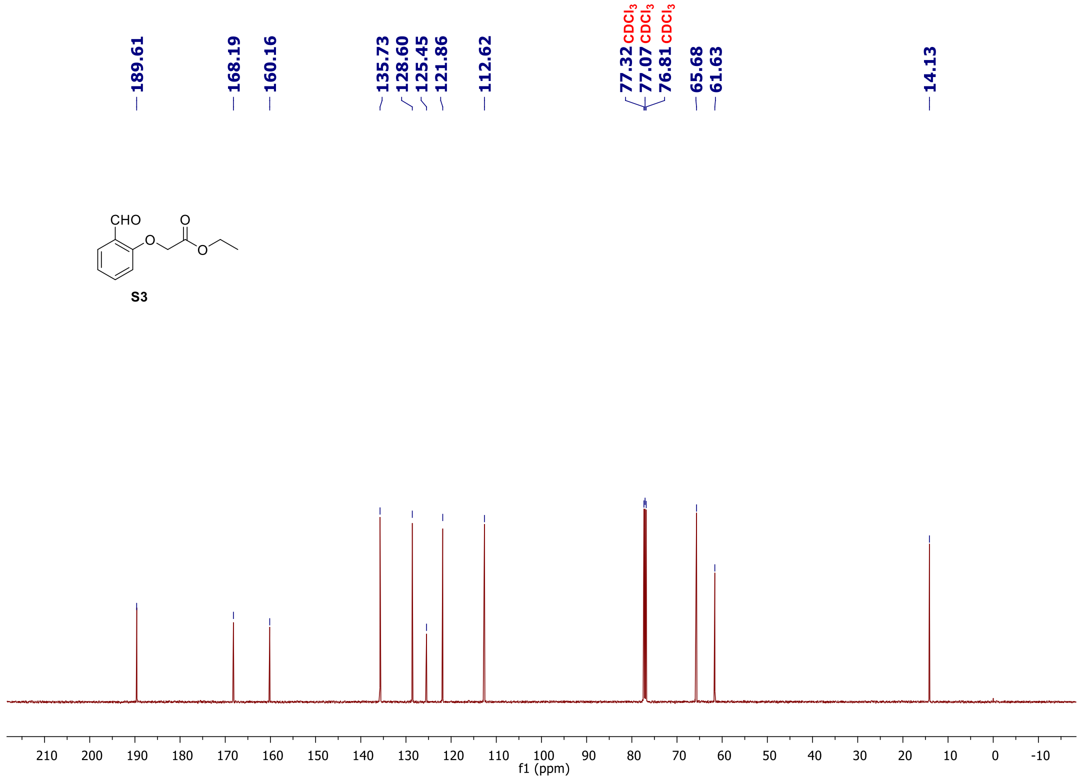

Supplementary Figure 81. ^{13}C NMR spectrum in $CDCl_3$ of compound **S3**

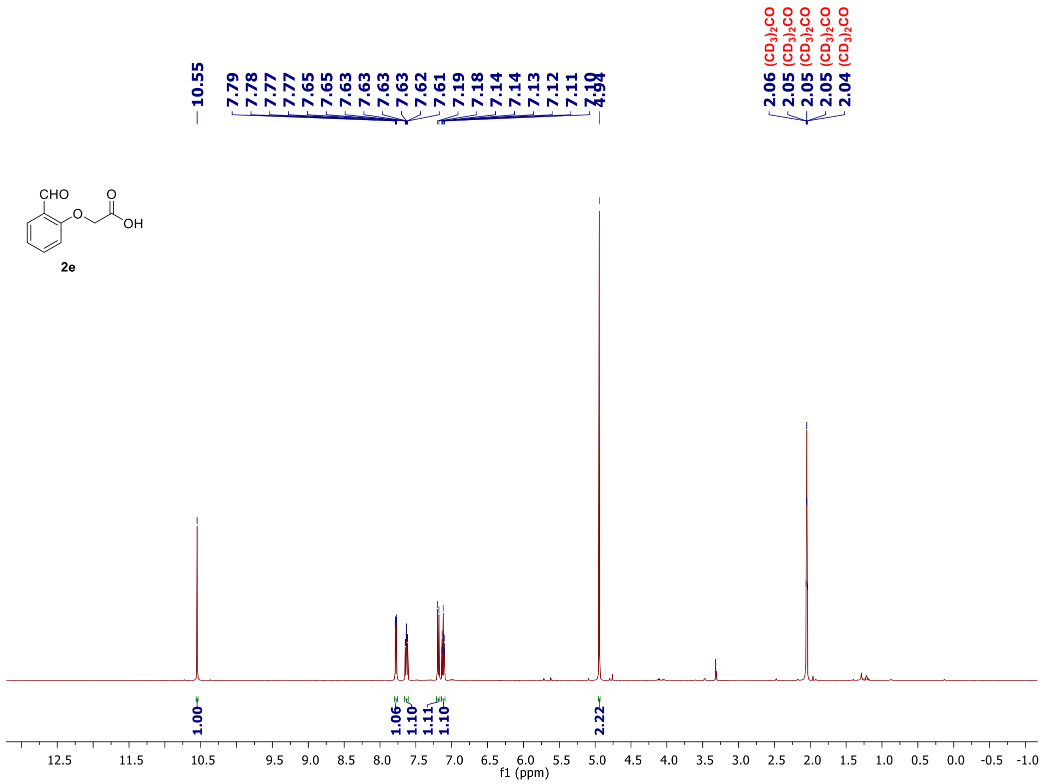

Supplementary Figure 82. ^{1}H NMR spectrum in $(CD_3)_2CO$ of compound **2e**

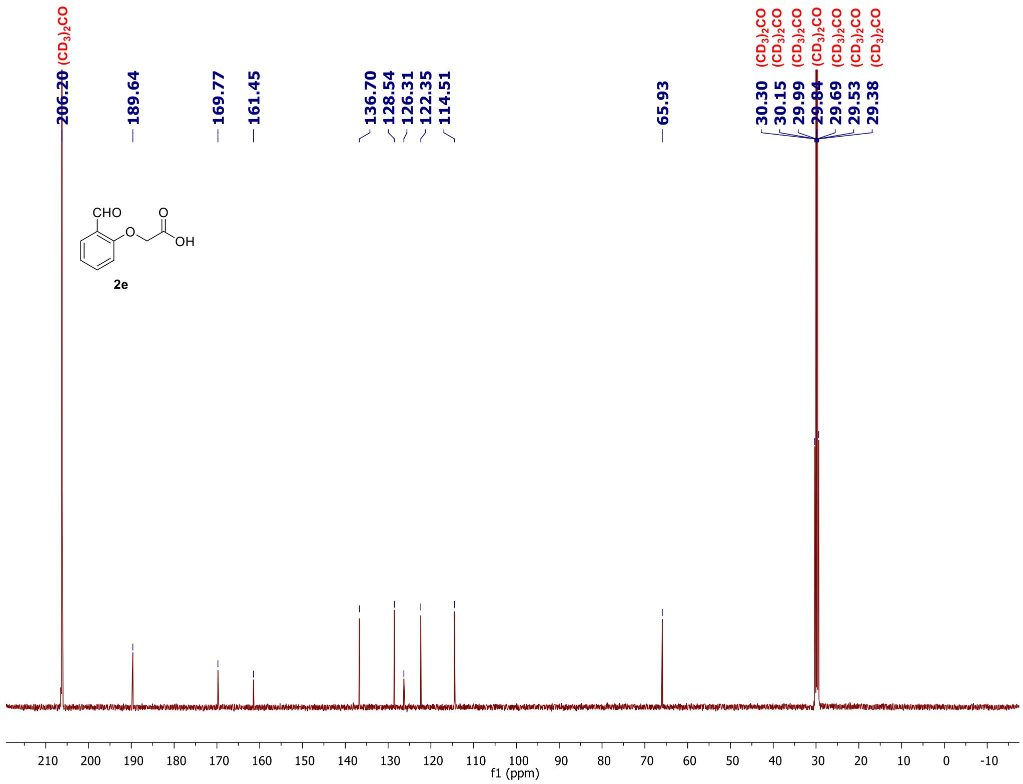

Supplementary Figure 83. ^{13}C NMR spectrum in $(CD_3)_2CO$ of compound **2e**

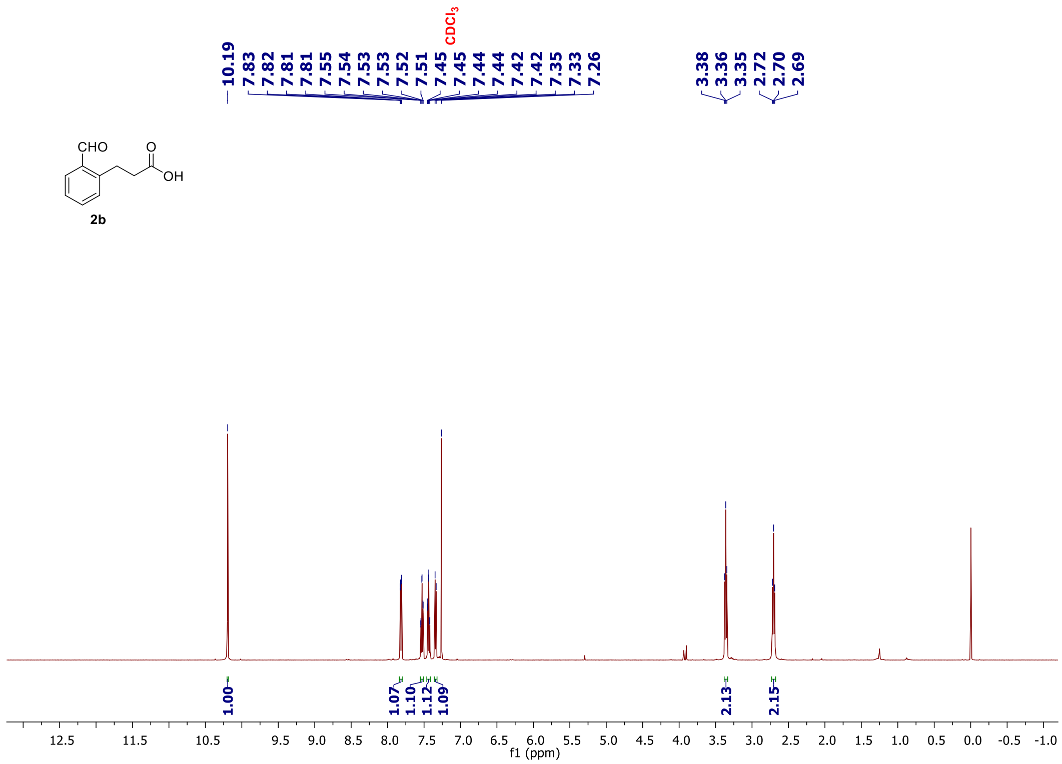

Supplementary Figure 84. ^{1}H NMR spectrum in $CDCl_3$ of compound **2b**

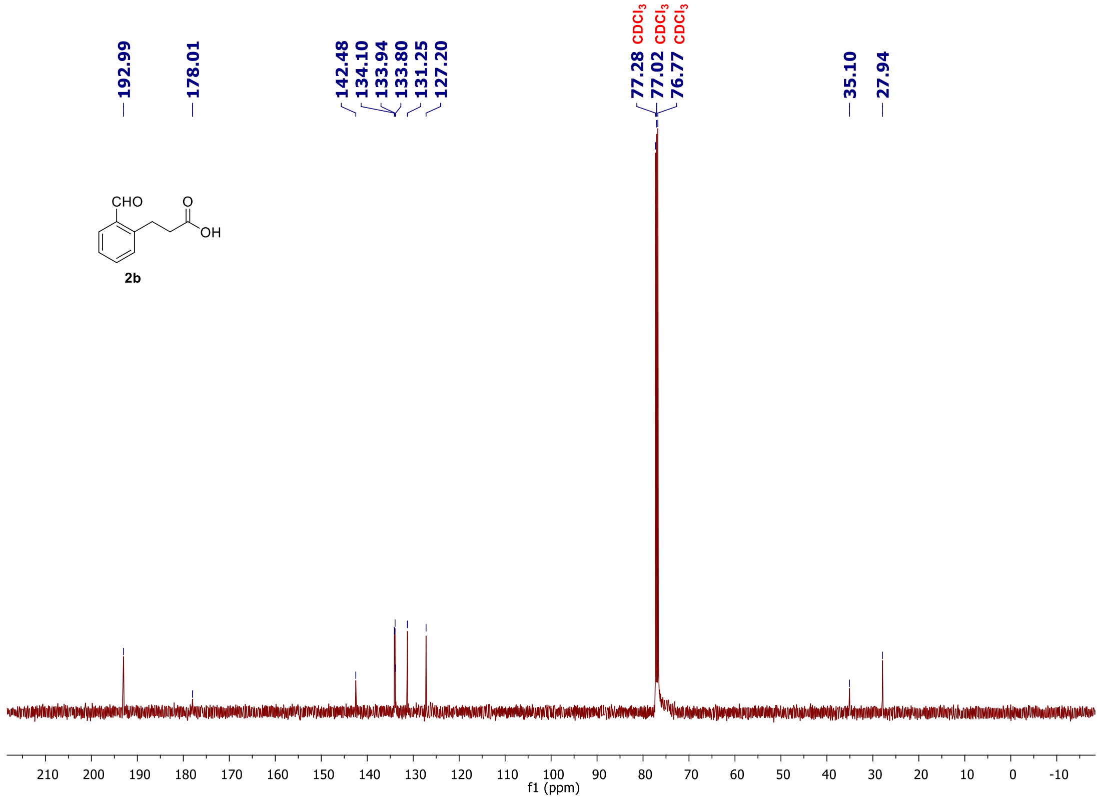

Supplementary Figure 85. ^{13}C NMR spectrum in $CDCl_3$ of compound **2b**

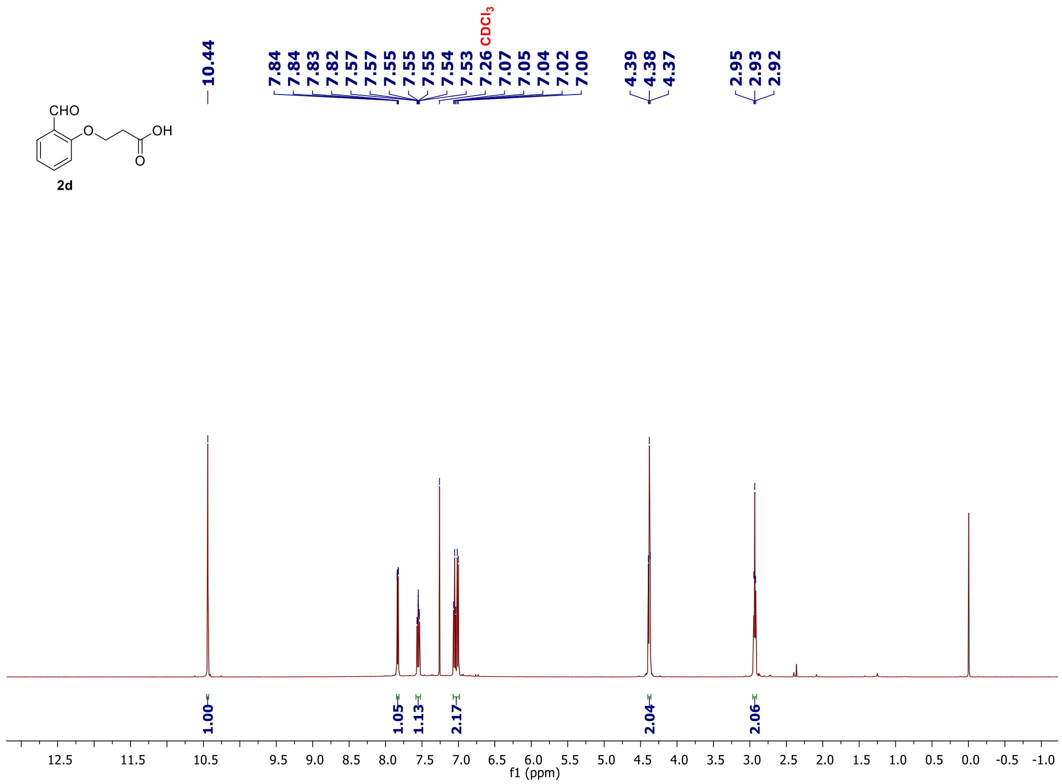

Supplementary Figure 86. ^{1}H NMR spectrum in $CDCl_3$ of compound **2d**

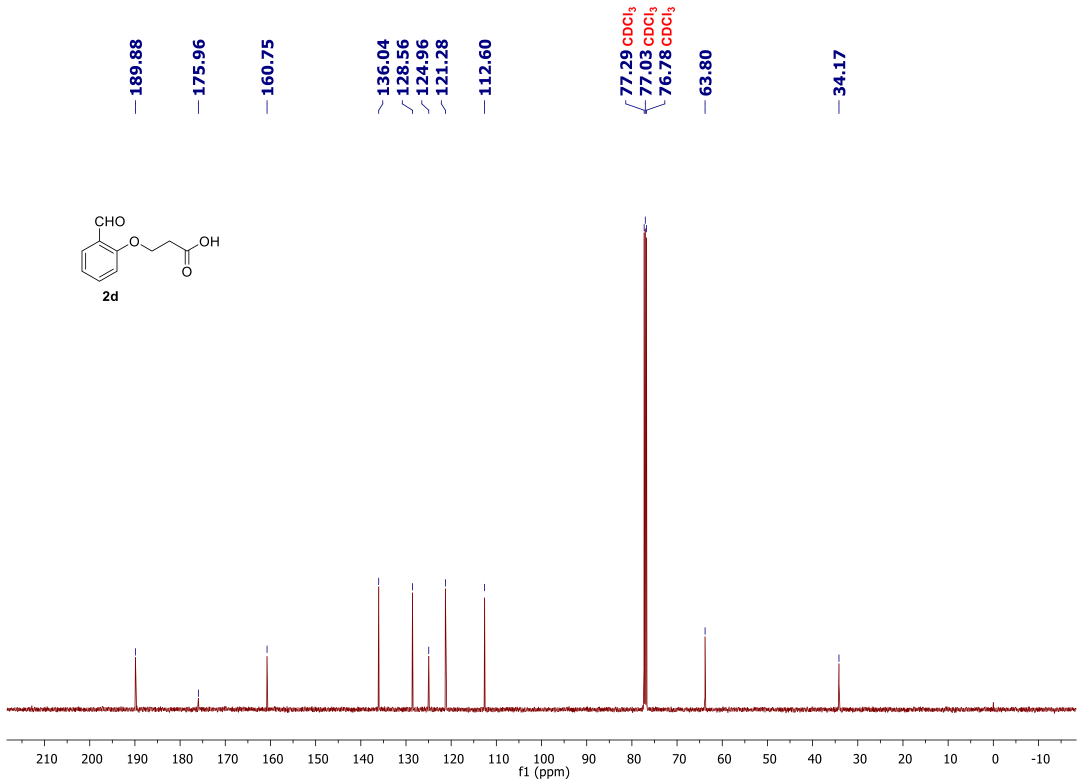

Supplementary Figure 87. ^{13}C NMR spectrum in $CDCl_3$ of compound **2d**

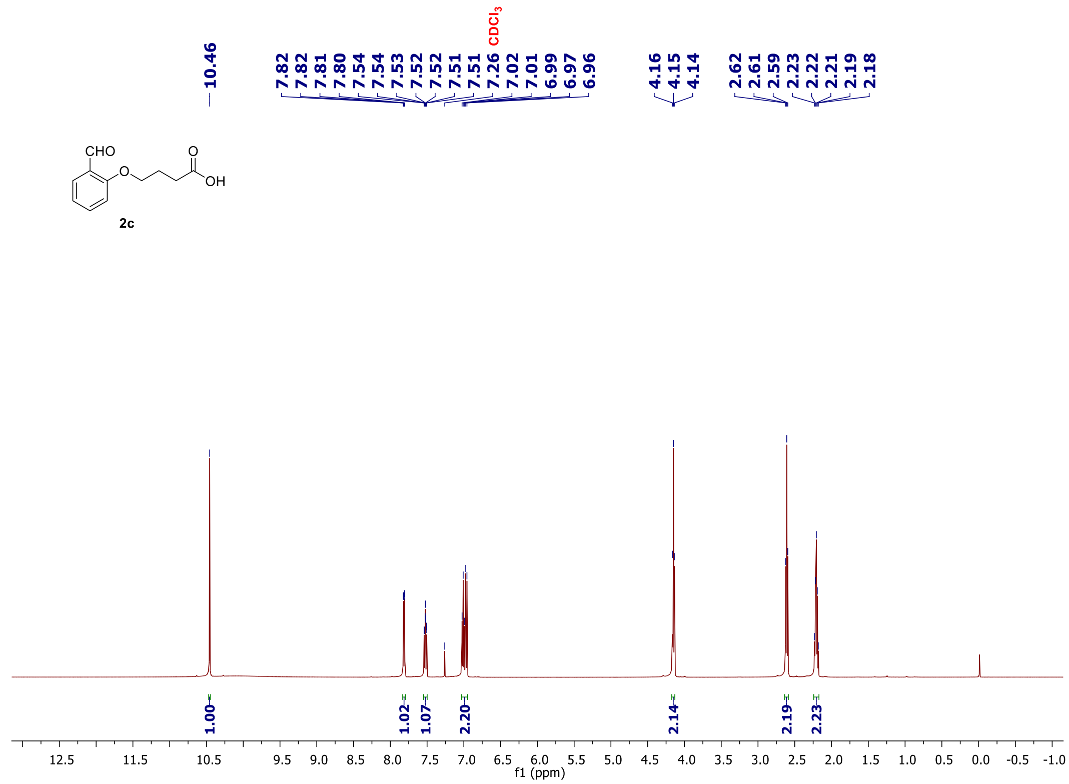

Supplementary Figure 88. ^{1}H NMR spectrum in $CDCl_3$ of compound **2c**

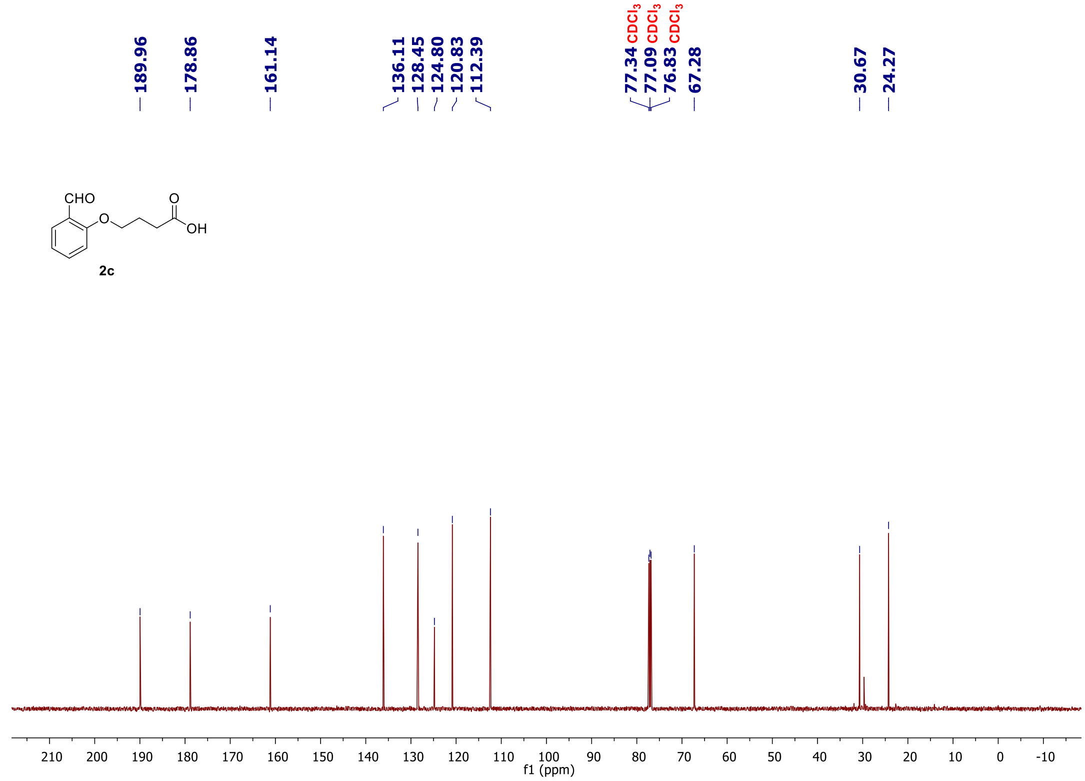

Supplementary Figure 89. ^{13}C NMR spectrum in $CDCl_3$ of compound **2c**

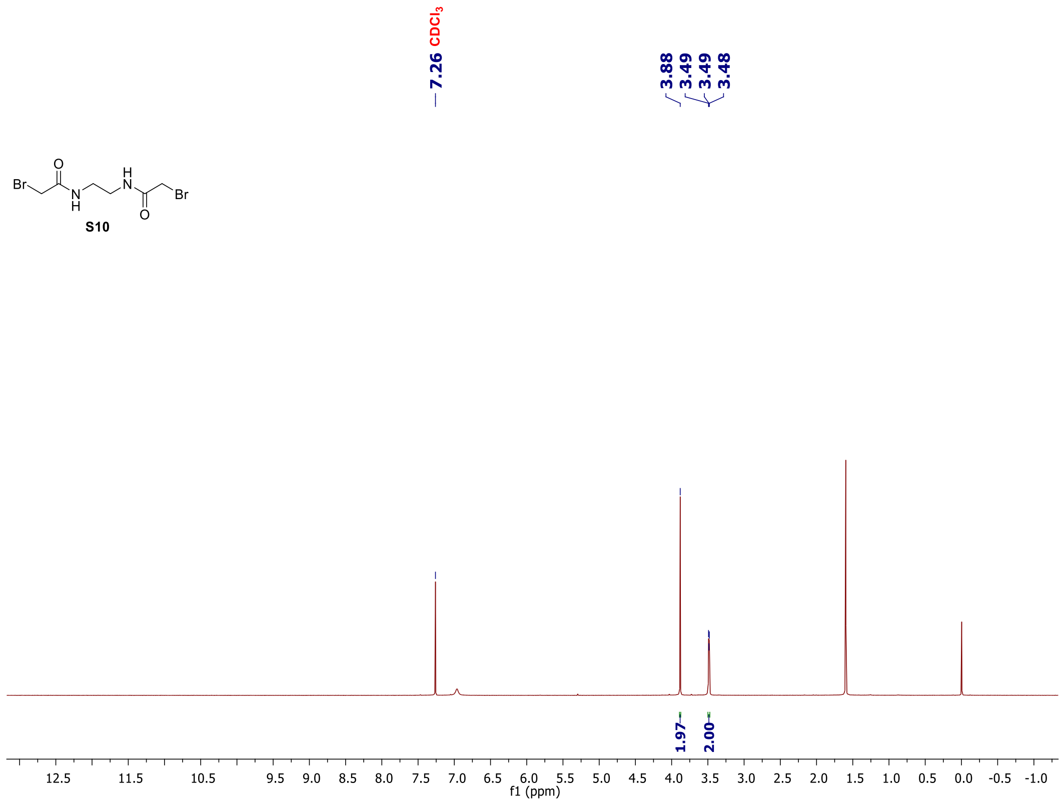

Supplementary Figure 90. ^{1}H NMR spectrum in $CDCl_3$ of compound **S10**

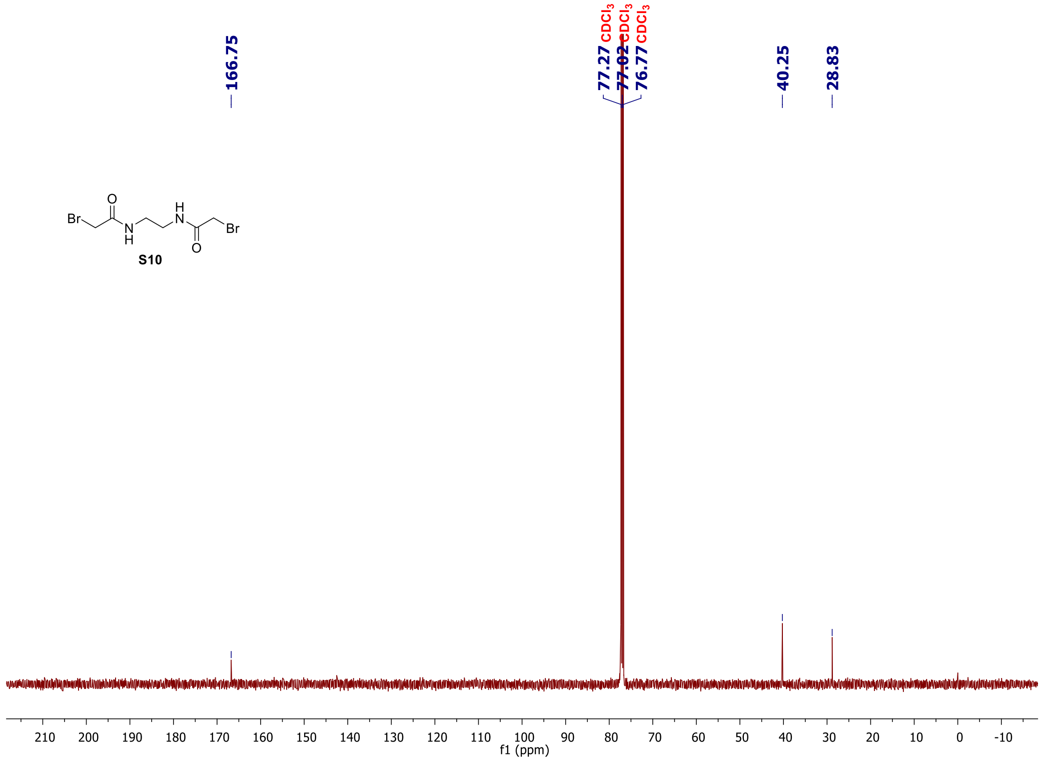

Supplementary Figure 91. ^{13}C NMR spectrum in $CDCl_3$ of compound **S10**

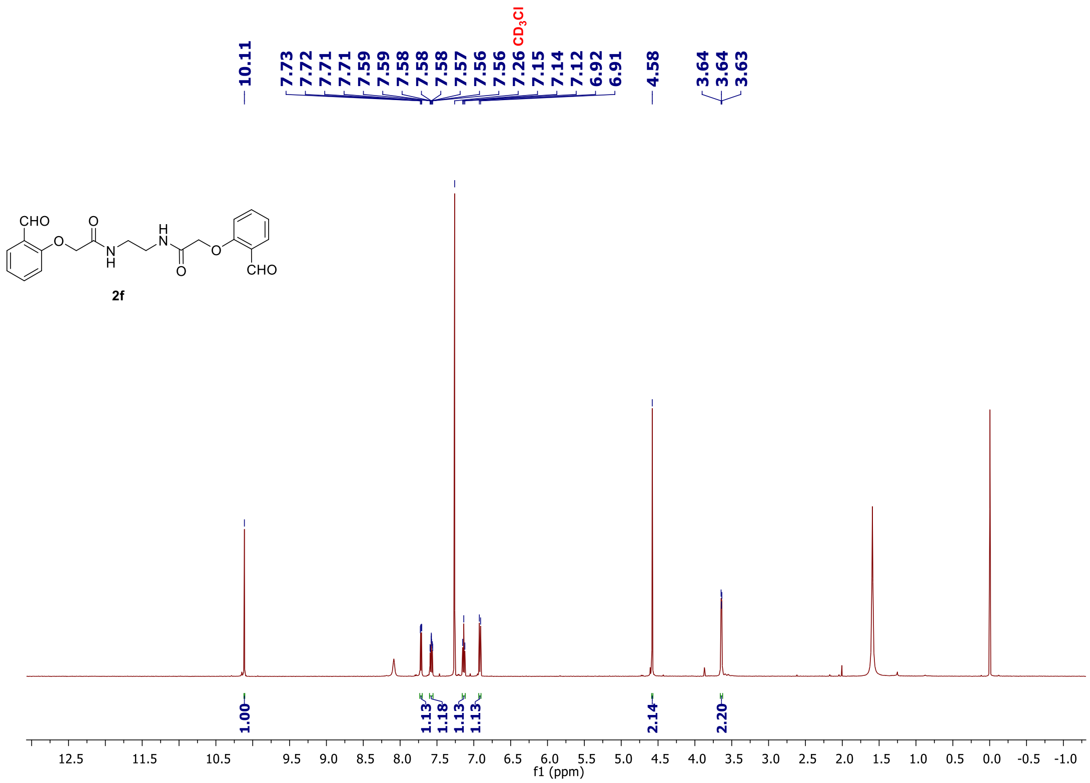

Supplementary Figure 92. ^{1}H NMR spectrum in $CDCl_3$ of compound **2f**

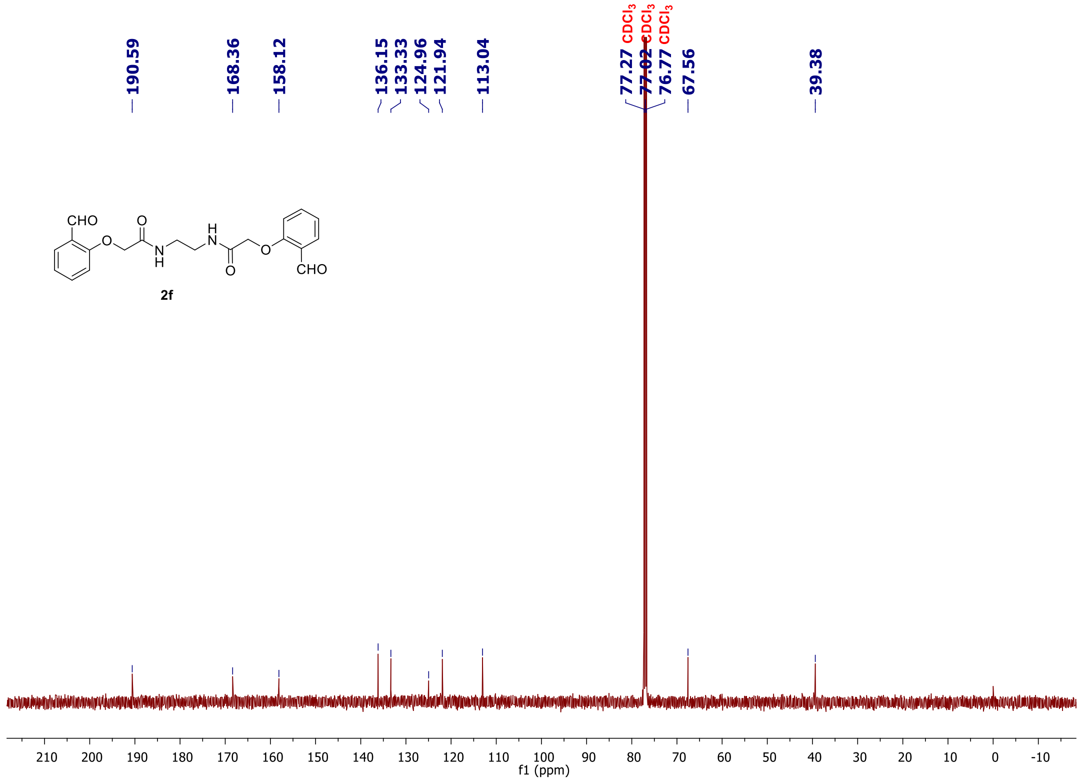

Supplementary Figure 93. ^{13}C NMR spectrum in $CDCl_3$ of compound **2f**

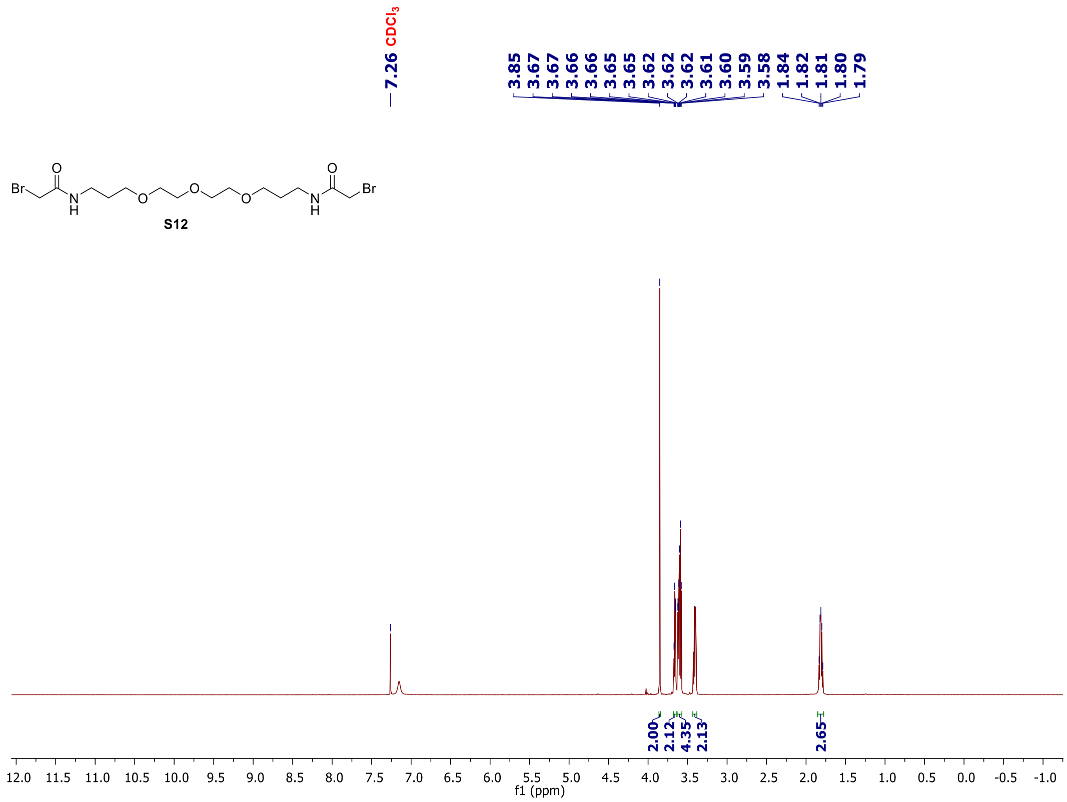

Supplementary Figure 94. ^{1}H NMR spectrum in $CDCl_3$ of compound **S12**

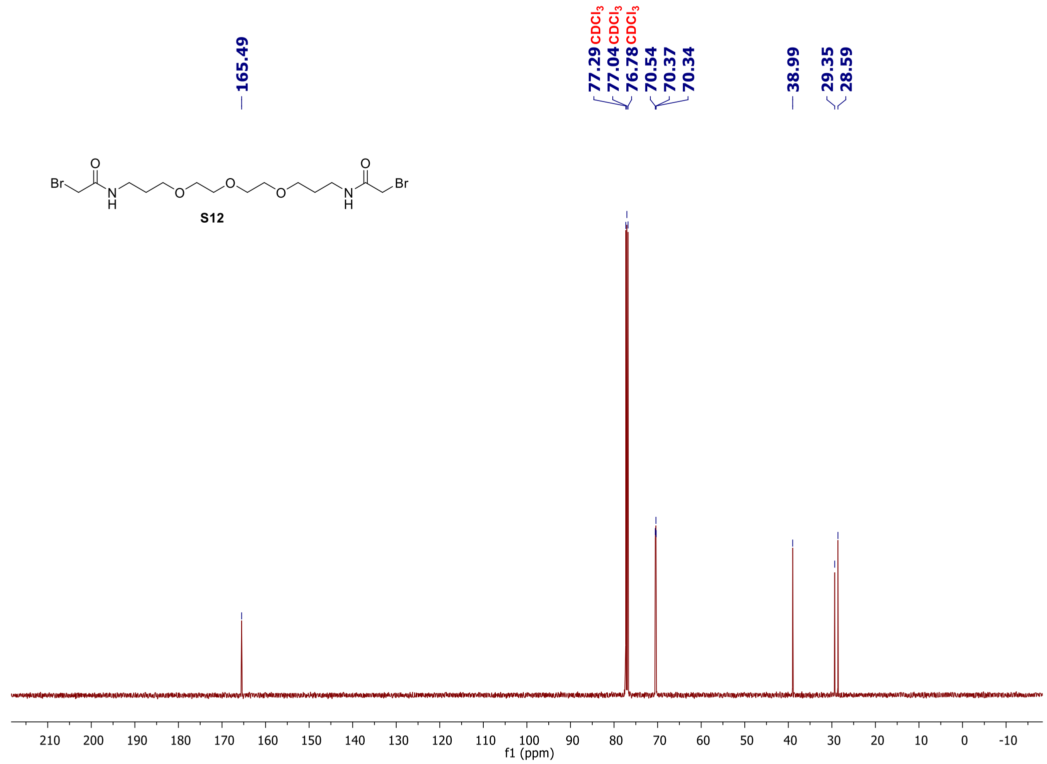

Supplementary Figure 95. ^{13}C NMR spectrum in $CDCl_3$ of compound **S12**

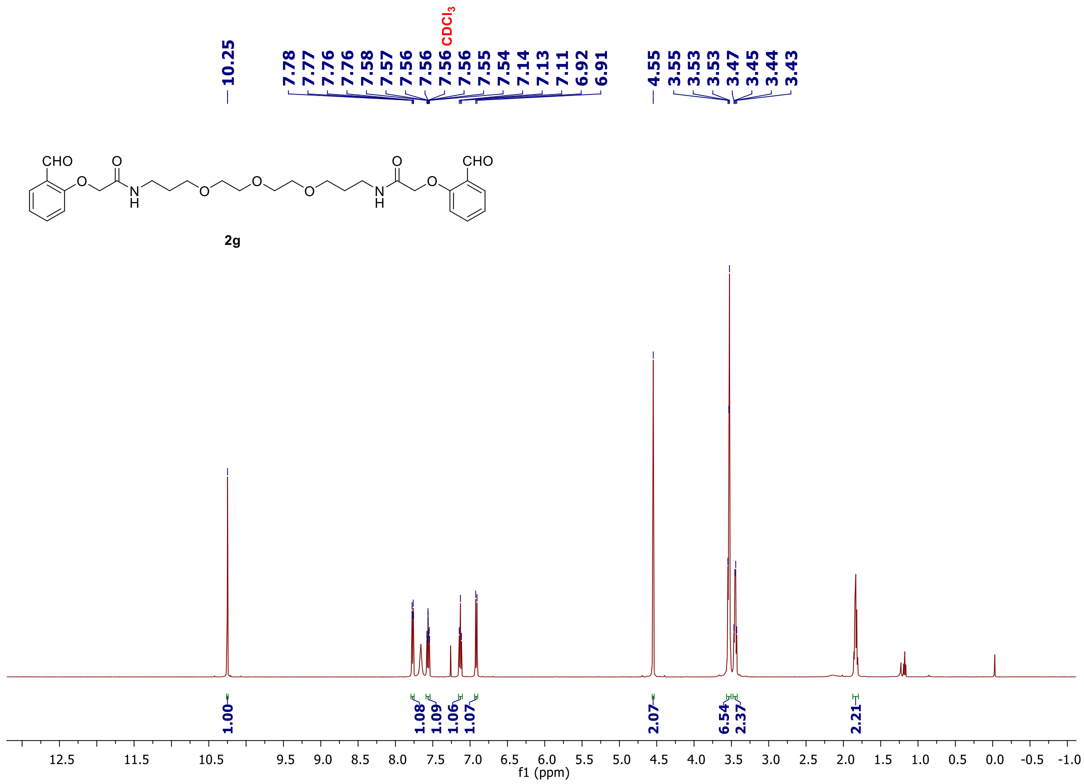

Supplementary Figure 96. ^{1}H NMR spectrum in $CDCl_3$ of compound **2g**

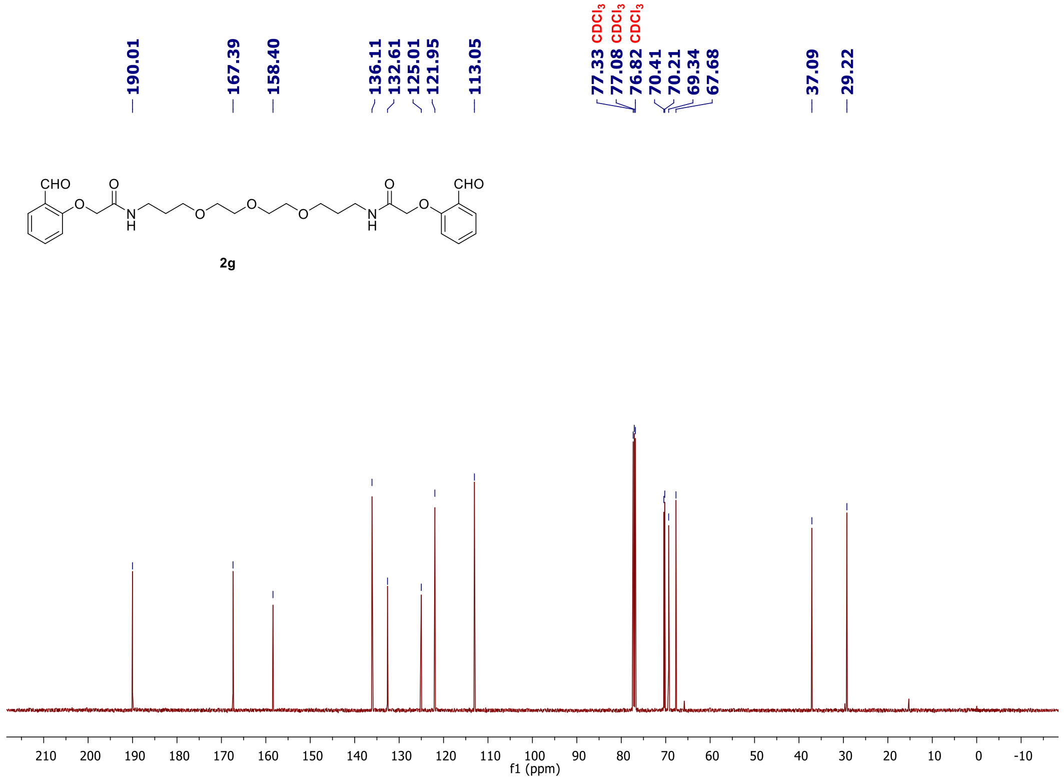

Supplementary Figure 97. ^{13}C NMR spectrum in $CDCl_3$ of compound **2g**

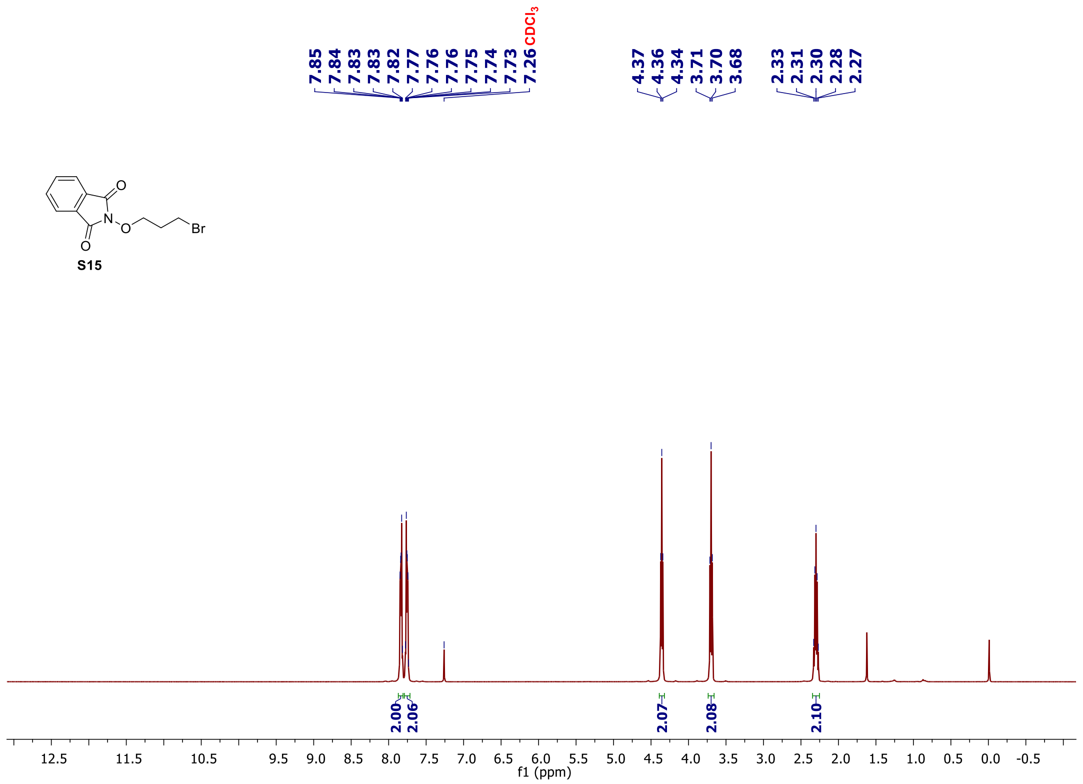

Supplementary Figure 98. ^{1}H NMR spectrum in $CDCl_3$ of compound **S15**

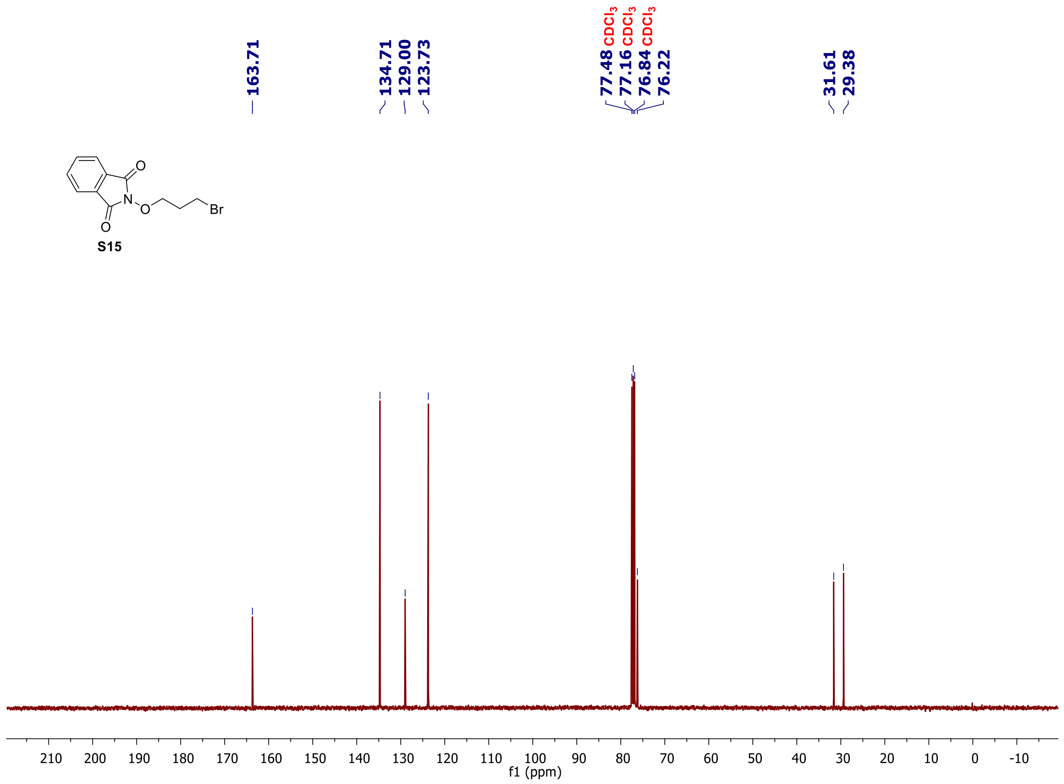

Supplementary Figure 99. ^{13}C NMR spectrum in $CDCl_3$ of compound **S15**

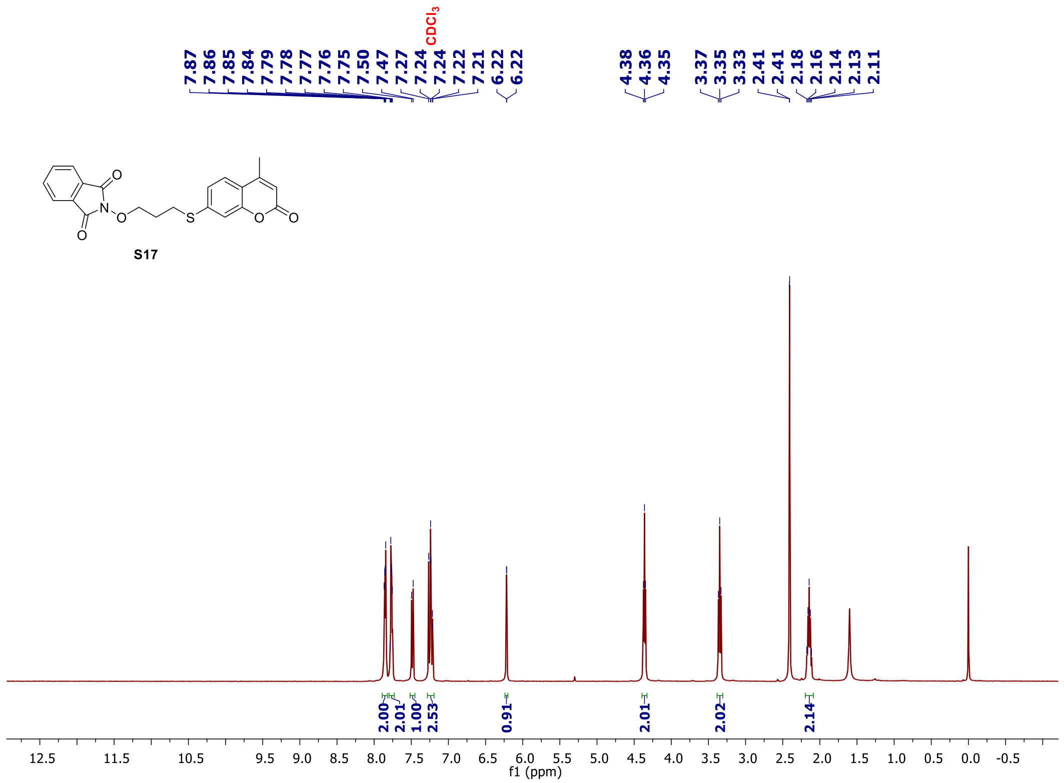

Supplementary Figure 100. ^{1}H NMR spectrum in $CDCl_3$ of compound **S17**

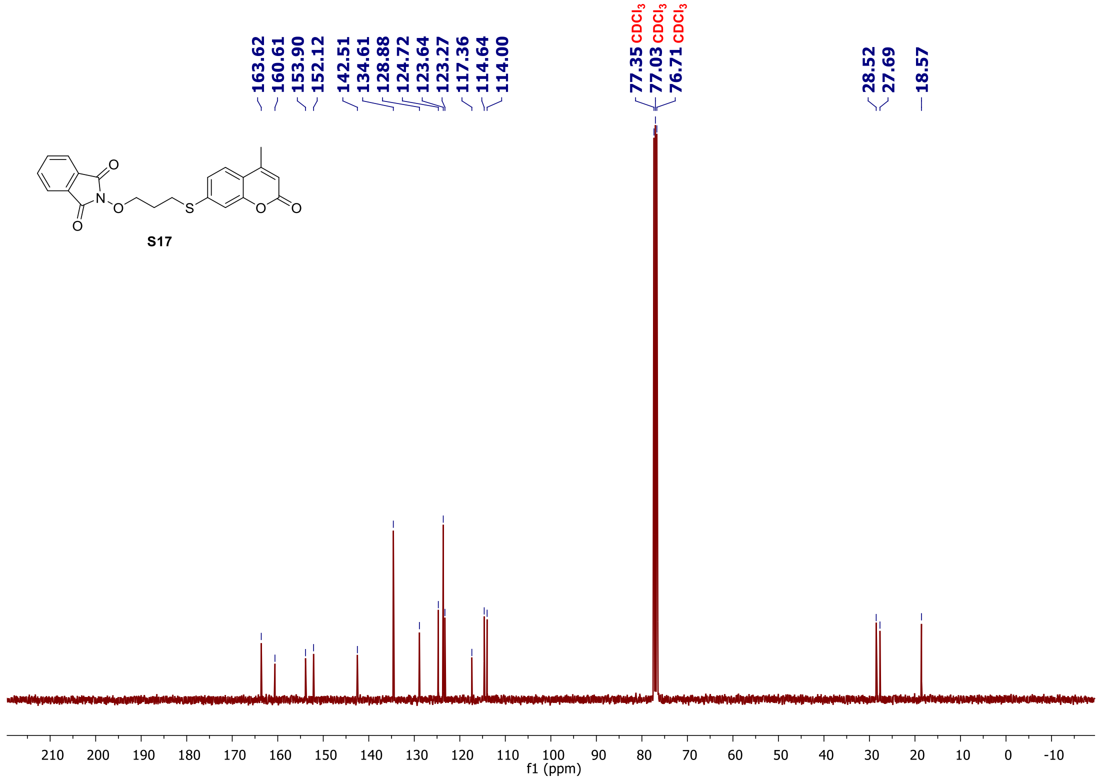

Supplementary Figure 101. ^{13}C NMR spectrum in $CDCl_3$ of compound **S17**

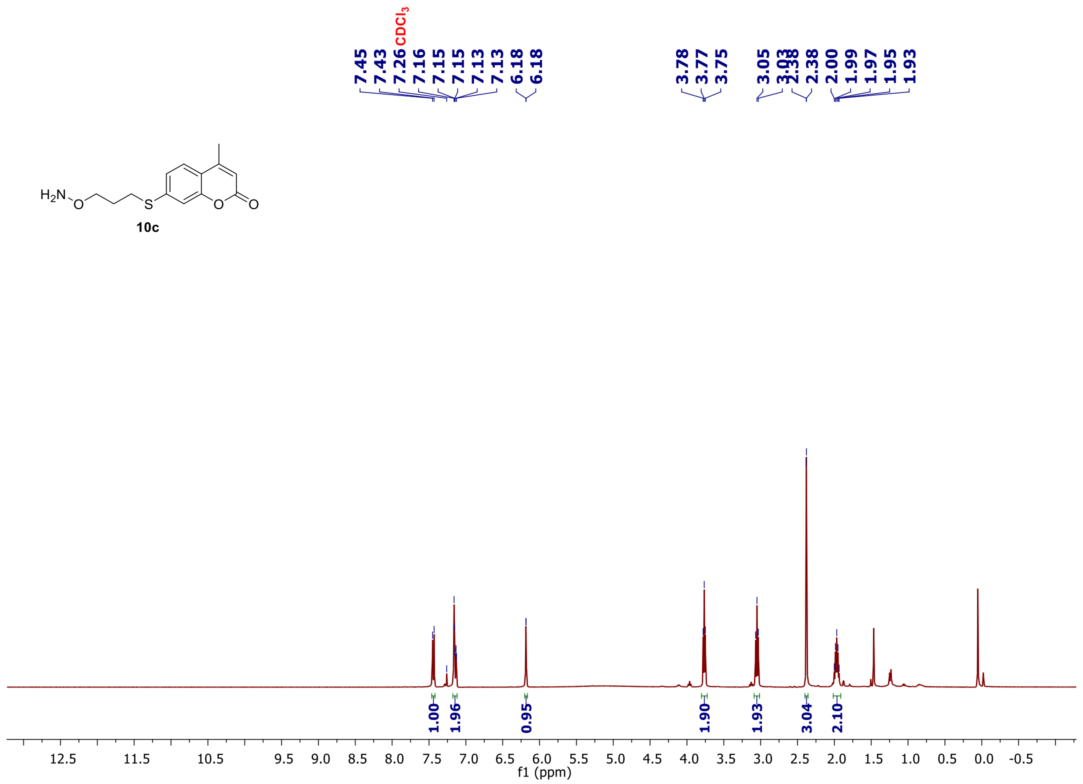

Supplementary Figure 102. ^{1}H NMR spectrum in $CDCl_3$ of compound **10c**

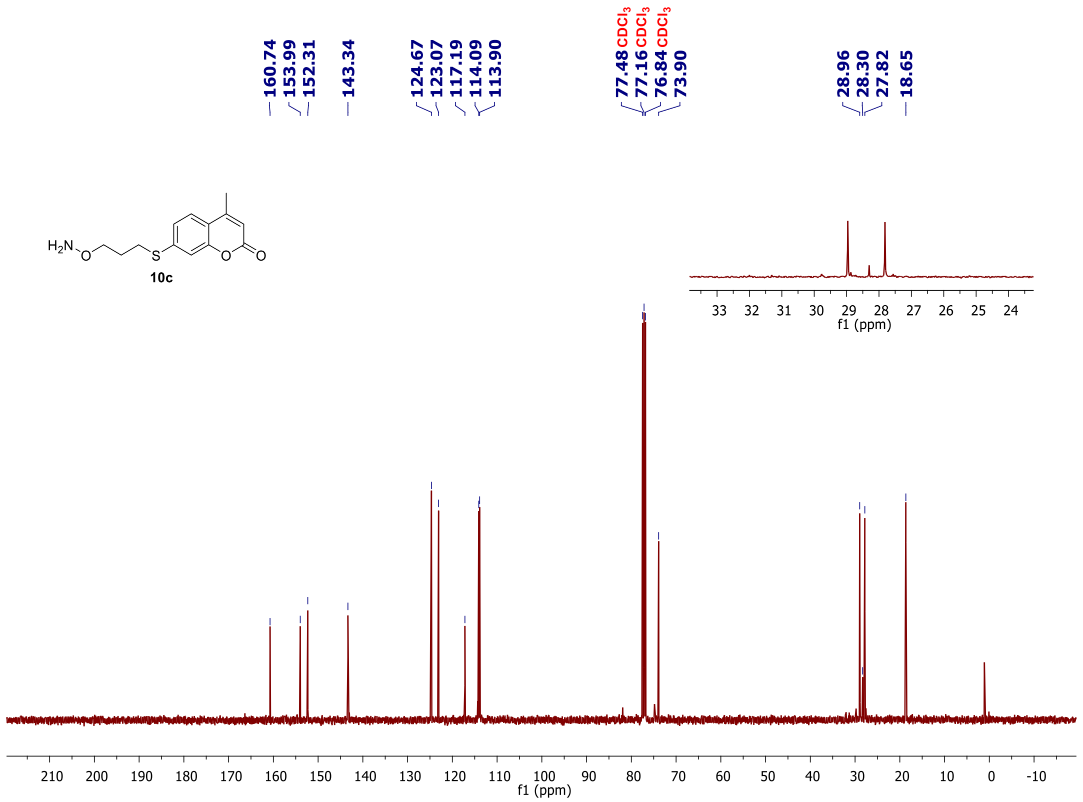

Supplementary Figure 103. ^{13}C NMR spectrum in $CDCl_3$ of compound **10c**

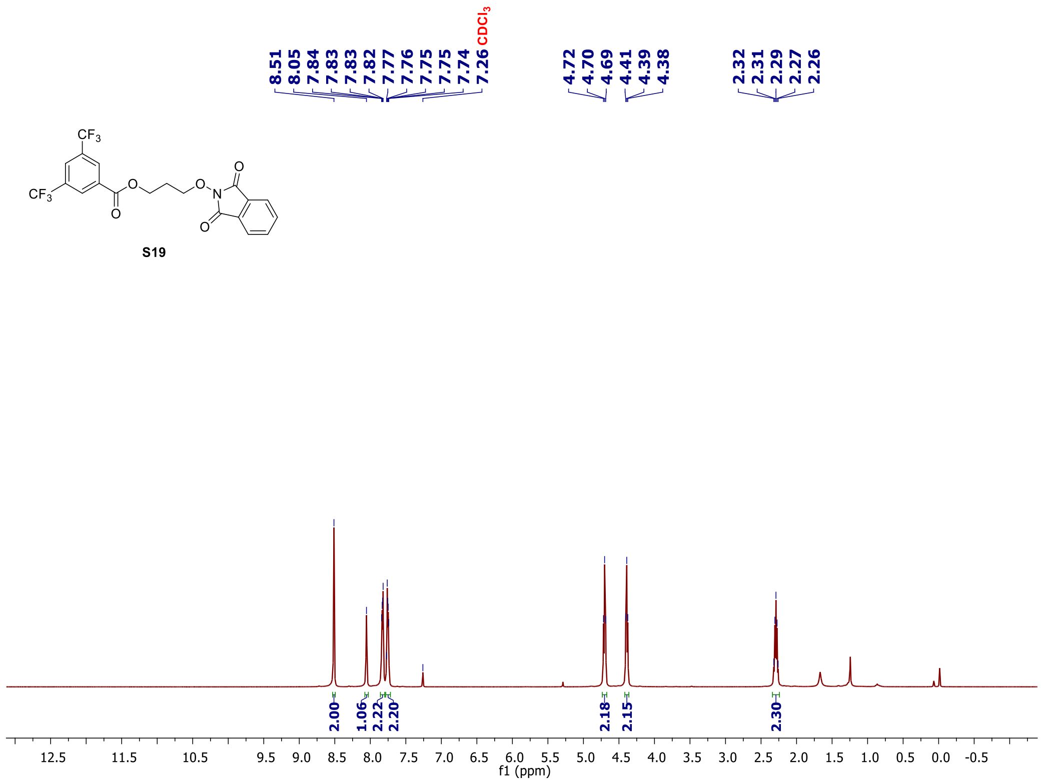

Supplementary Figure 104. ^{1}H NMR spectrum in $CDCl_3$ of compound **S19**

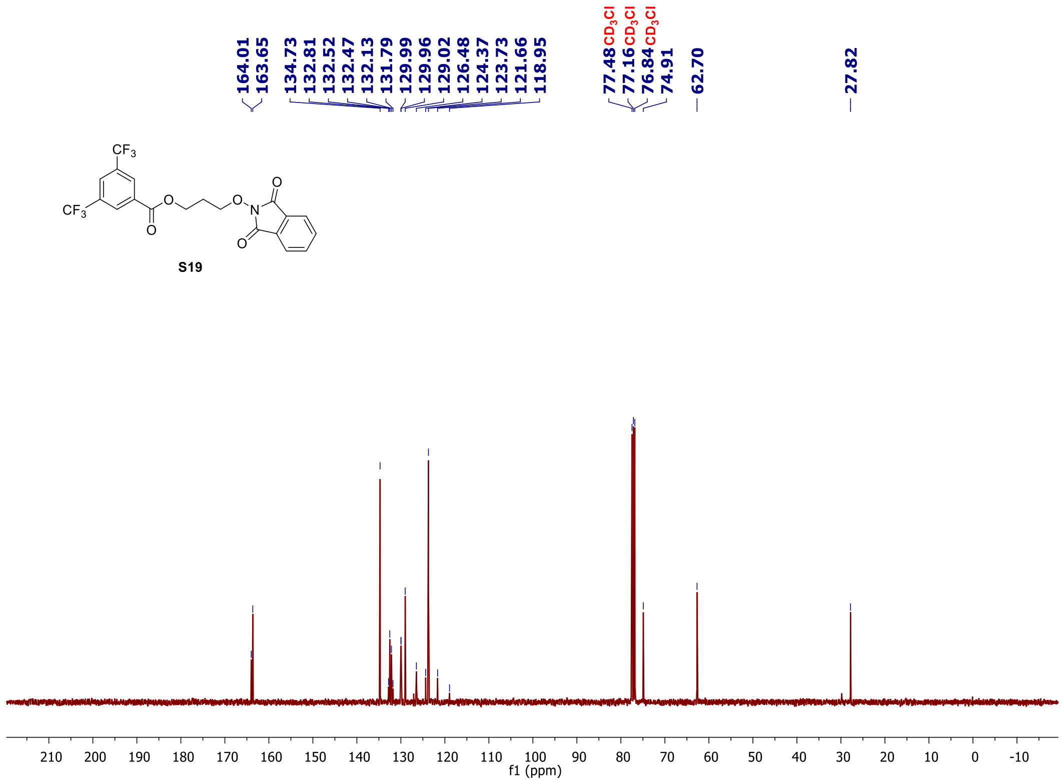

Supplementary Figure 105. ^{13}C NMR spectrum in $CDCl_3$ of compound **S19**

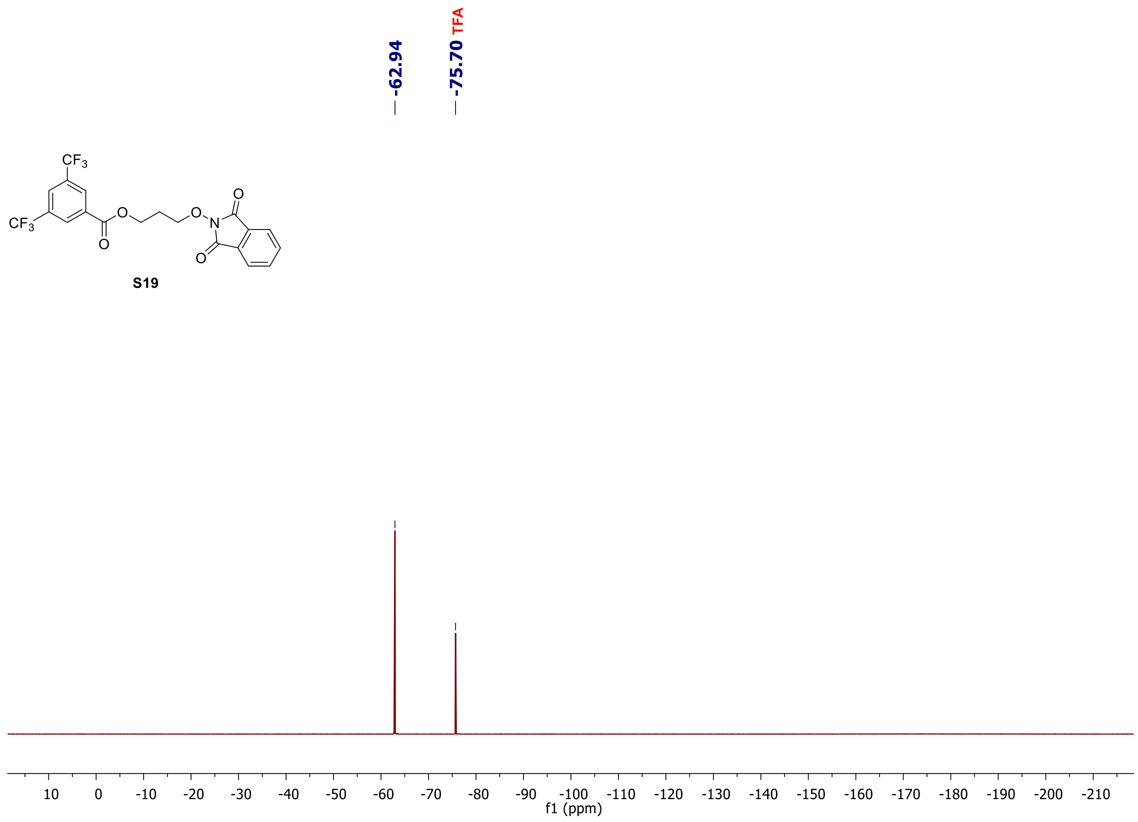

Supplementary Figure 106. ^{19}F NMR spectrum of compound **S19**

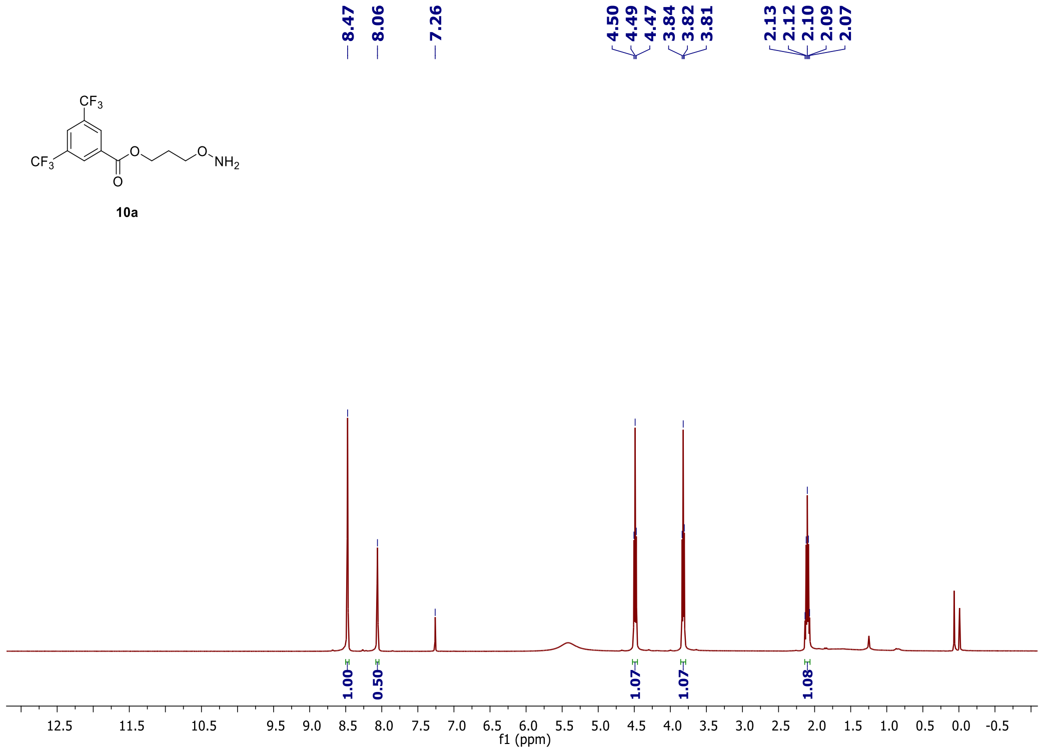

Supplementary Figure 107. ^{1}H NMR spectrum in $CDCl_3$ of compound **10a**

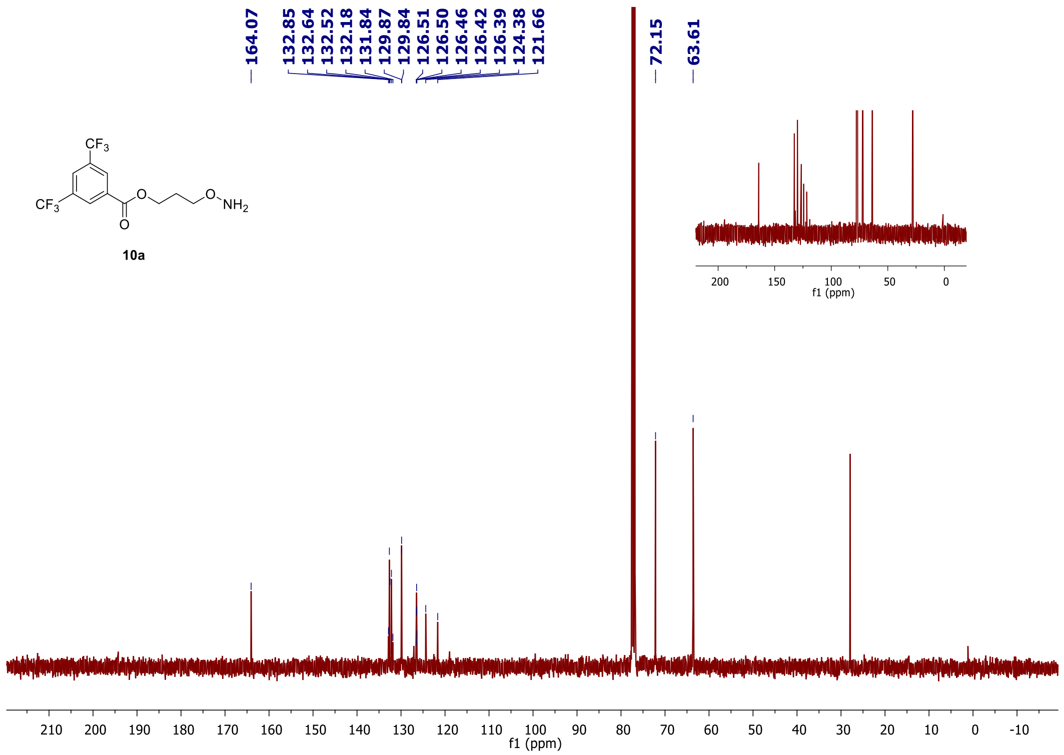

Supplementary Figure 108. ^{13}C NMR spectrum in $CDCl_3$ of compound **10a**

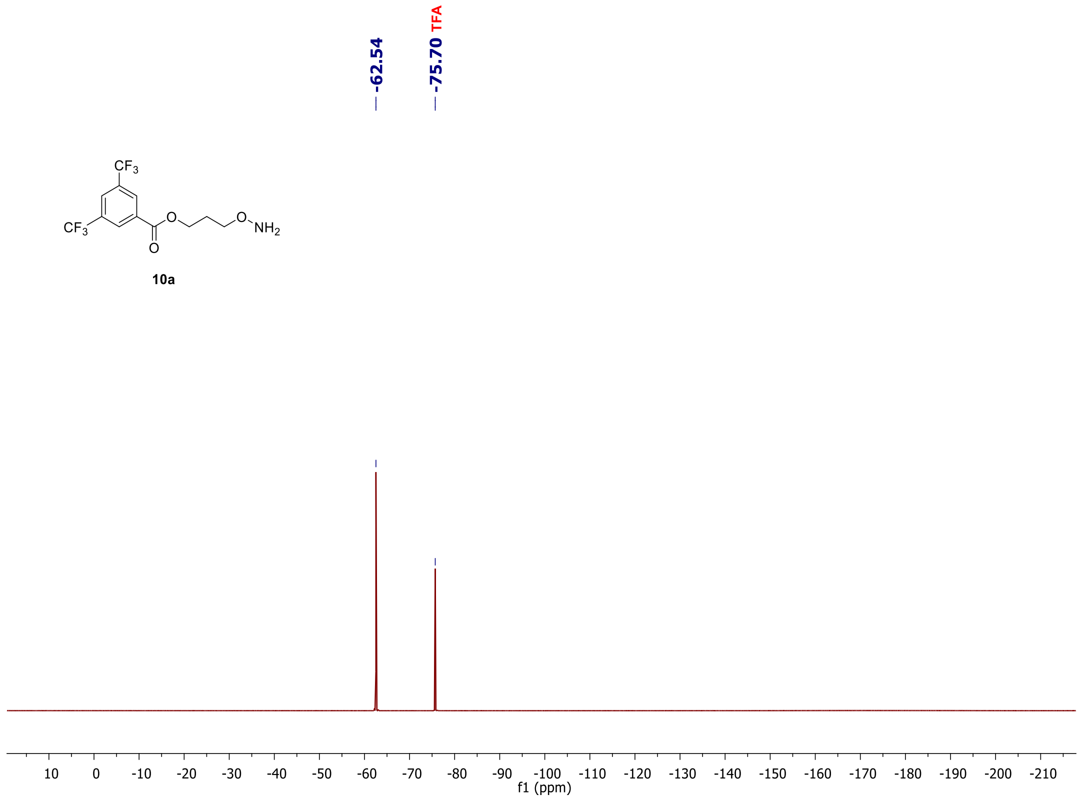

Supplementary Figure 109. ^{19}F NMR spectrum of compound **10a**

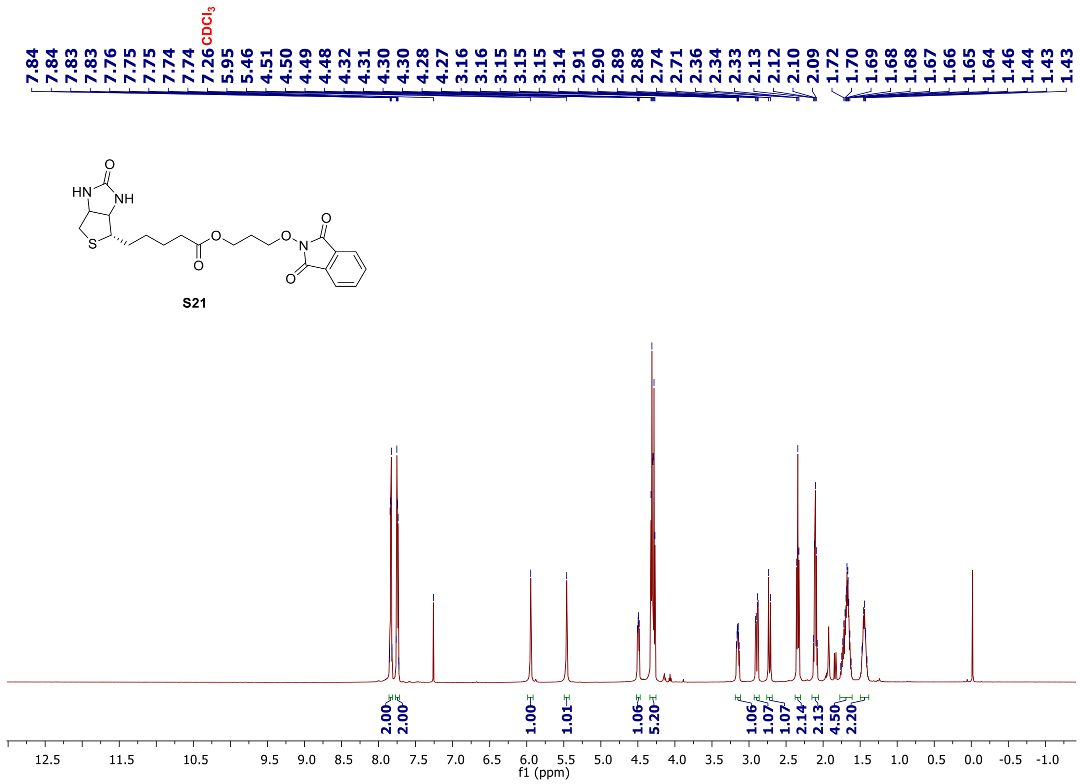

Supplementary Figure 110. ^{1}H NMR spectrum in $CDCl_3$ of compound **S21**

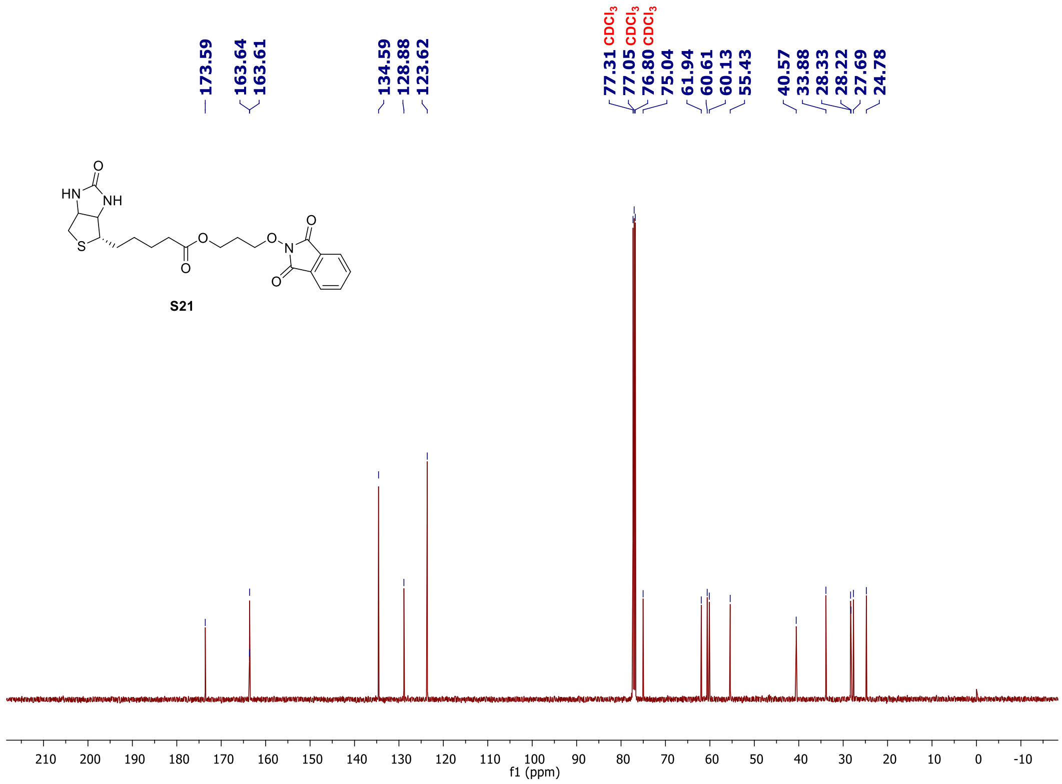

Supplementary Figure 111. ^{13}C NMR spectrum in $CDCl_3$ of compound **S21**

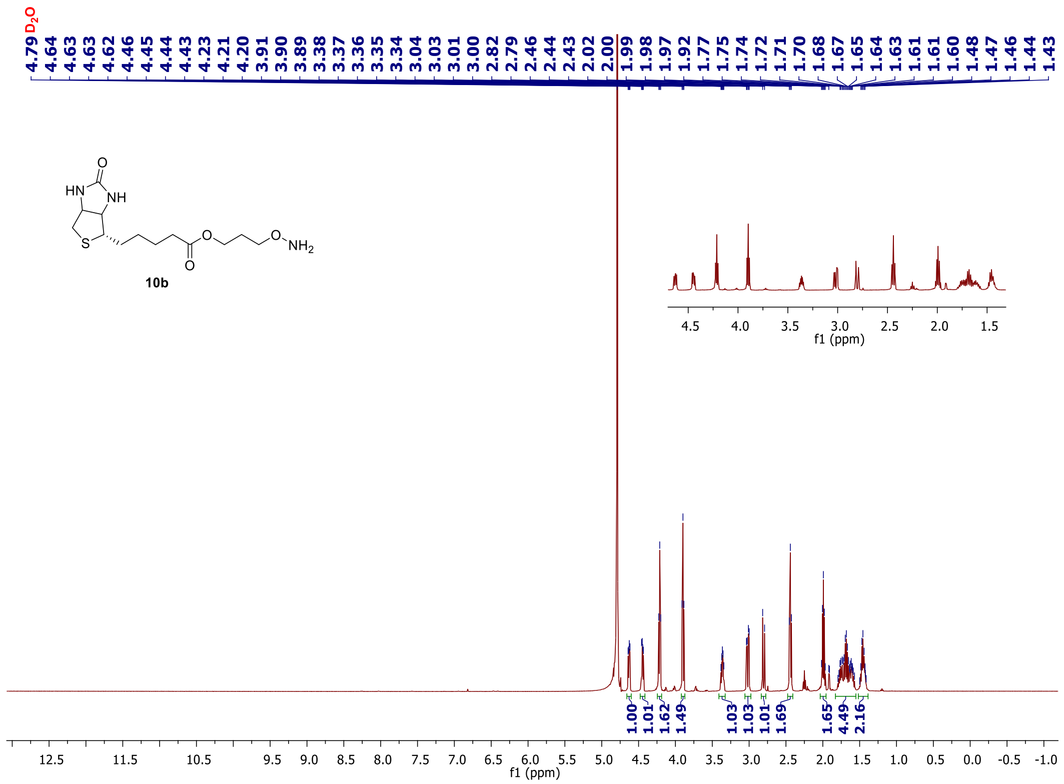

Supplementary Figure 112. ^{1}H NMR spectrum in D_2O of compound **10b**

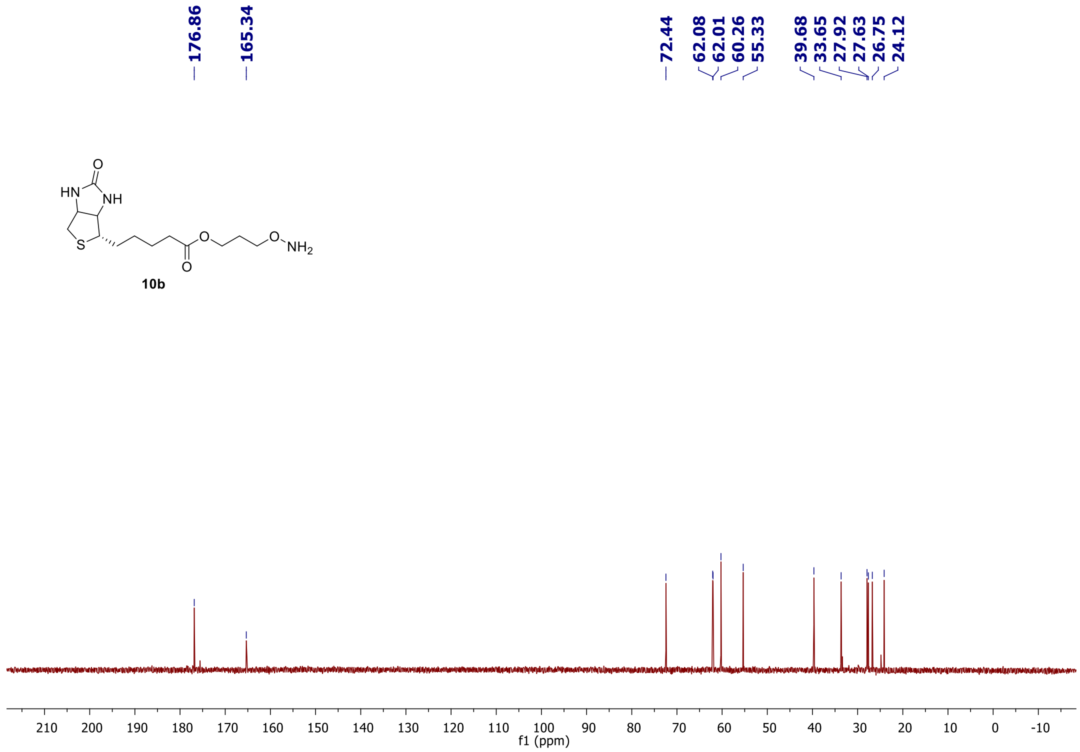

Supplementary Figure 113. ^{13}C NMR spectrum in D_2O of compound **10b**

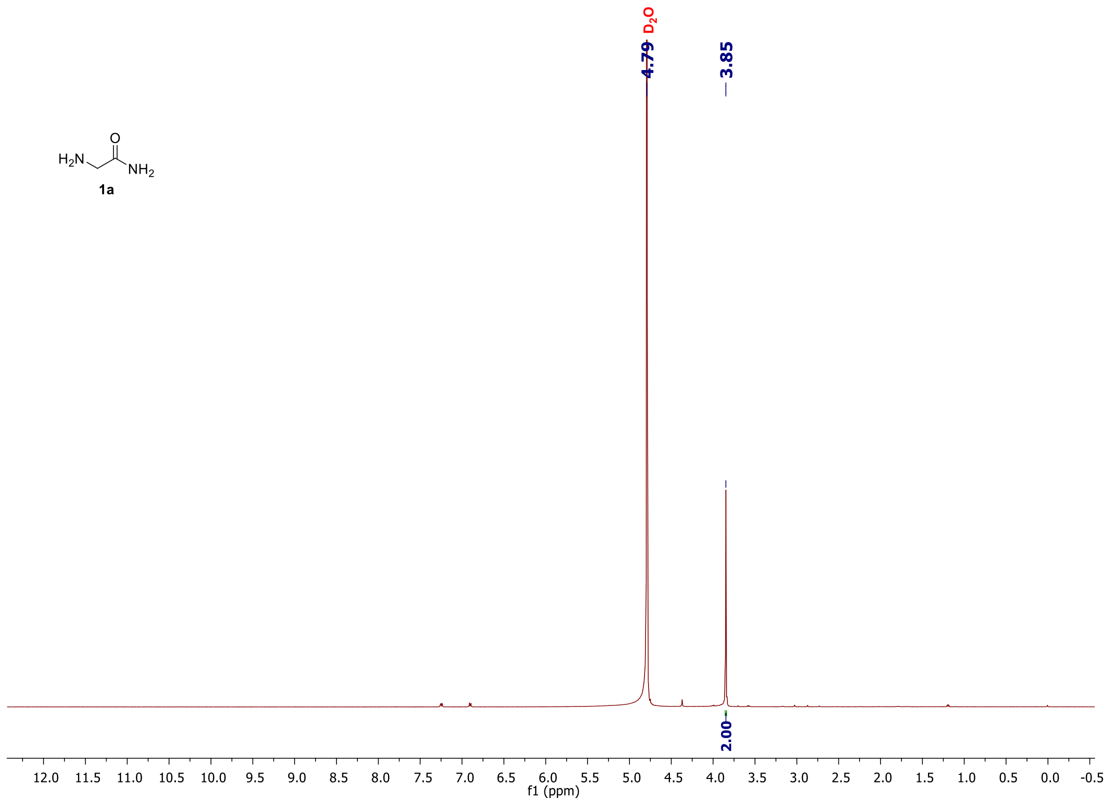

Supplementary Figure 114. ^{1}H NMR spectrum in D_2O of compound **1a**

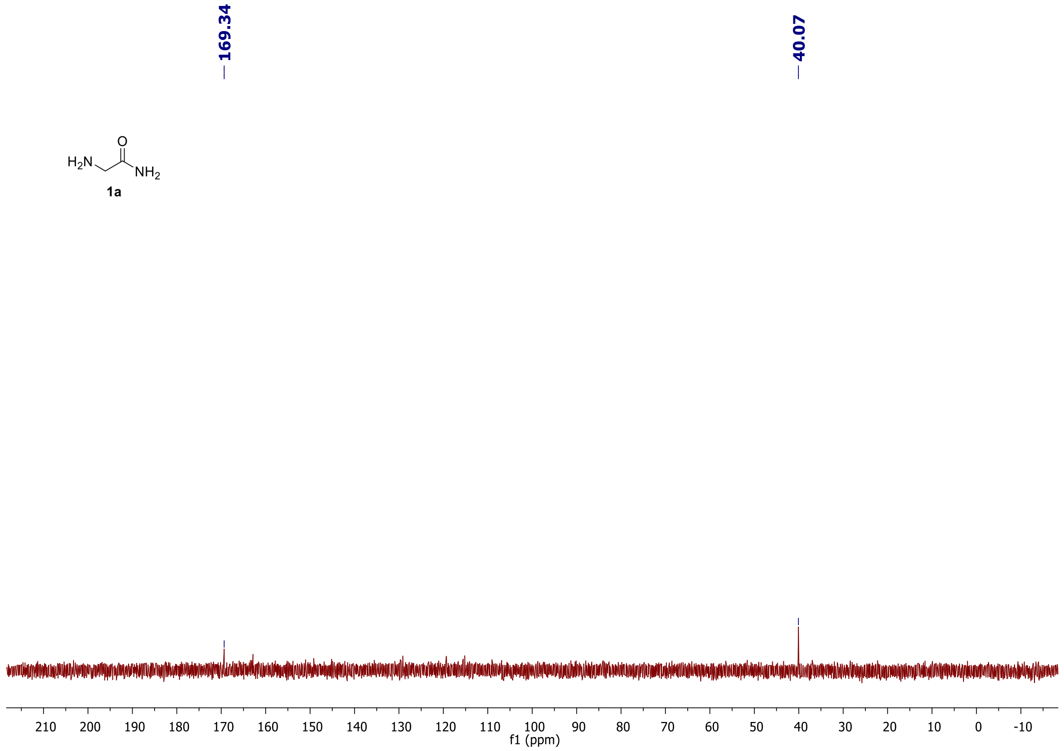

Supplementary Figure 115. ^{13}C NMR spectrum in D_2O of compound **1a**

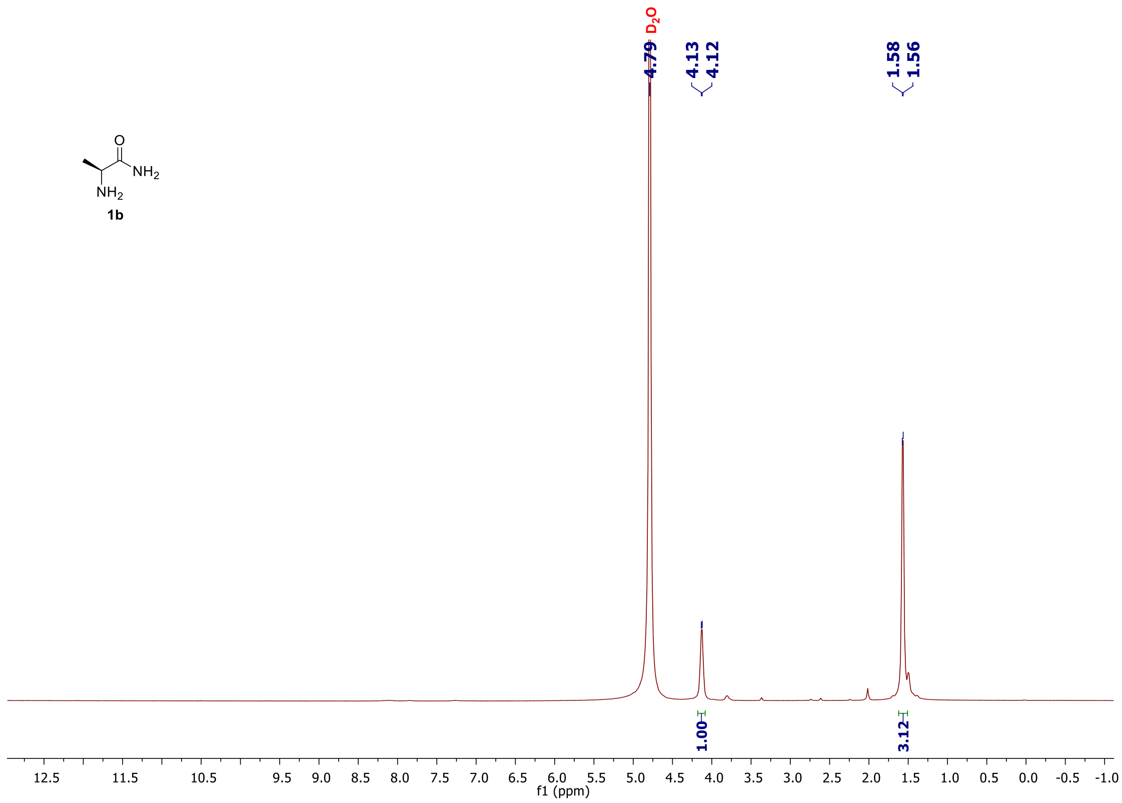

Supplementary Figure 116. ^{1}H NMR spectrum in D_2O of compound **1b**

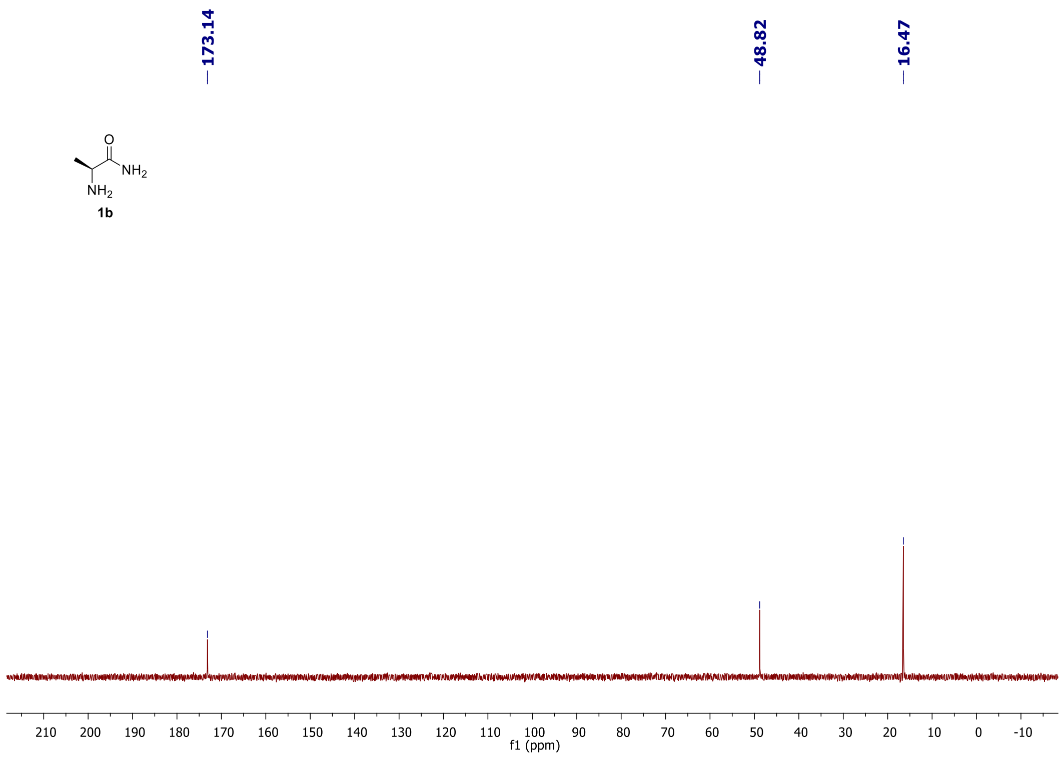

Supplementary Figure 117. ^{13}C NMR spectrum in D_2O of compound **1b**

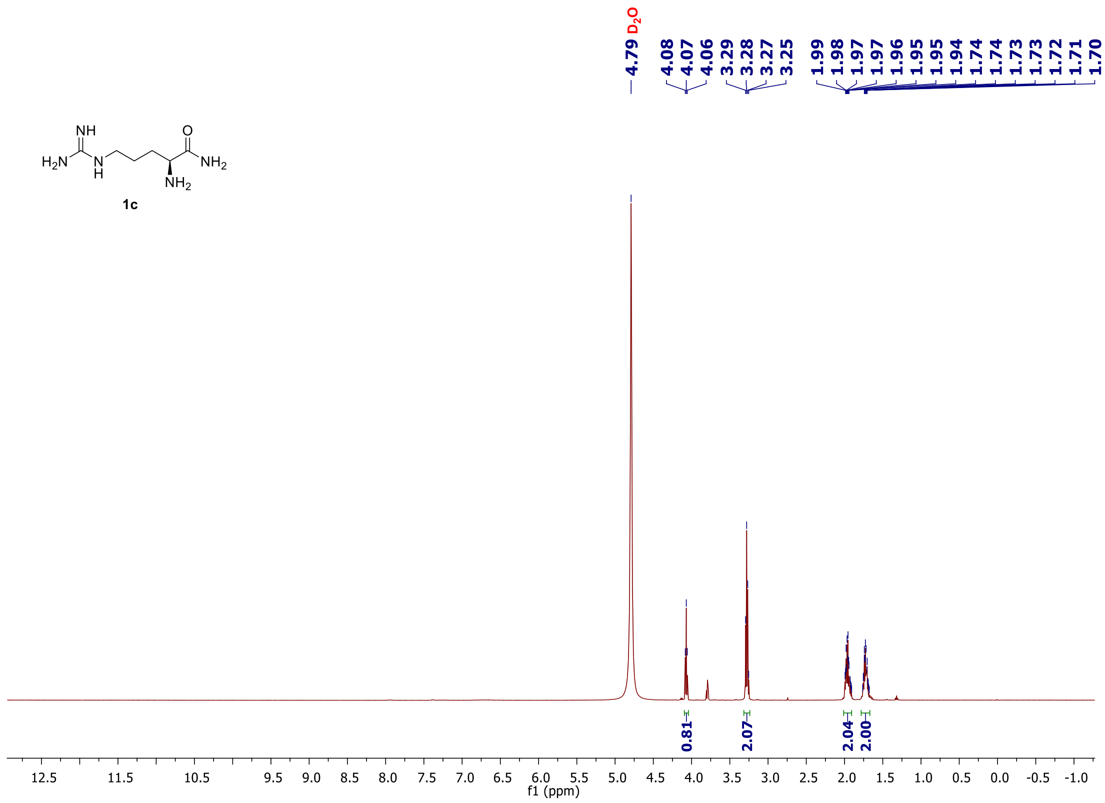

Supplementary Figure 118. ^{1}H NMR spectrum in D_2O of compound **1c**

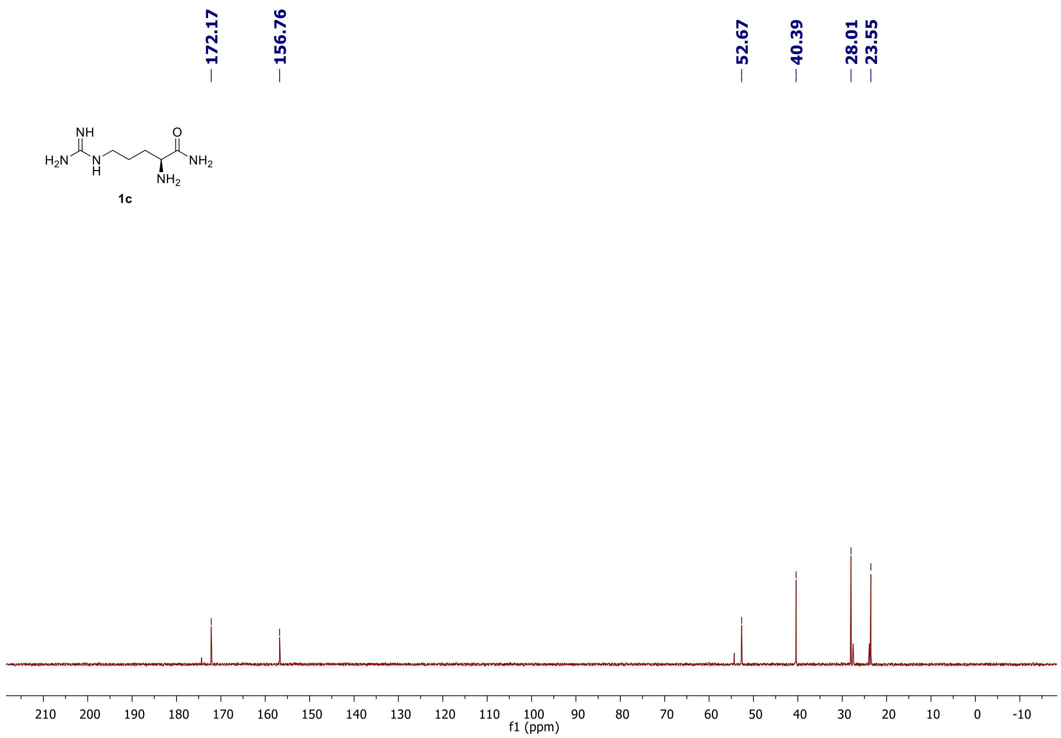

Supplementary Figure 119. ^{13}C NMR spectrum in D_2O of compound **1c**

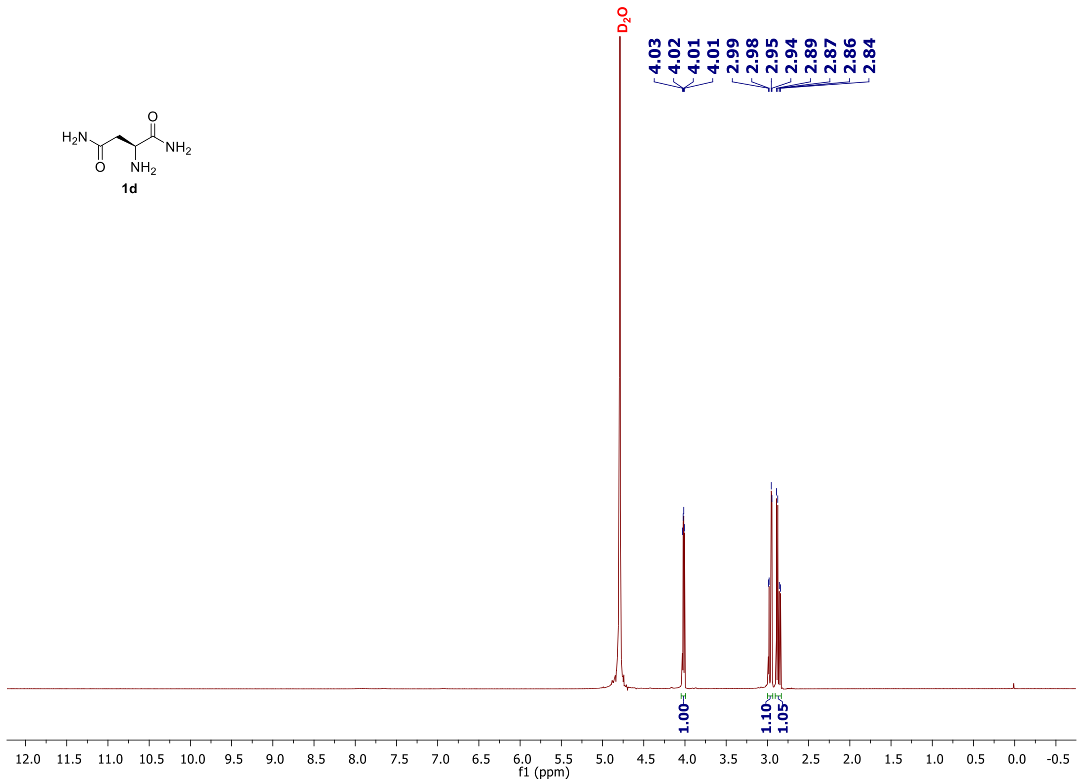

Supplementary Figure 120. ^{1}H NMR spectrum in D_2O of compound **1d**

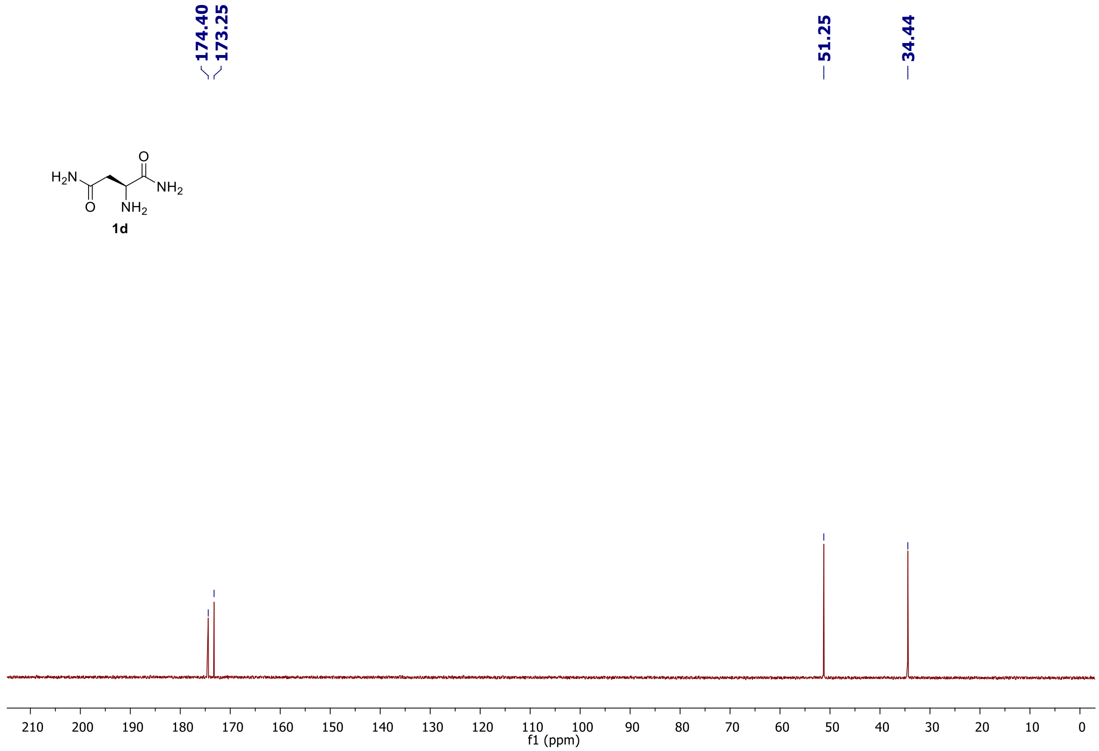

Supplementary Figure 121. ^{13}C NMR spectrum in D_2O of compound **1d**

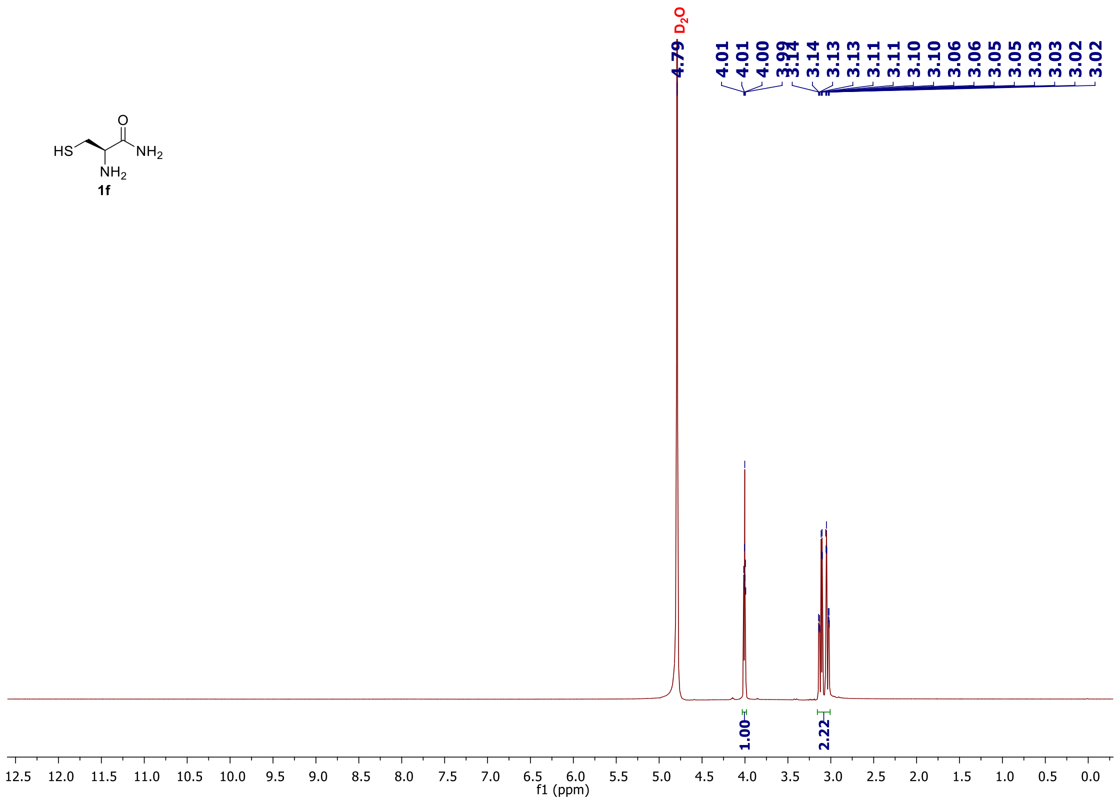

Supplementary Figure 122. ^{1}H NMR spectrum in D_2O of compound **1f**

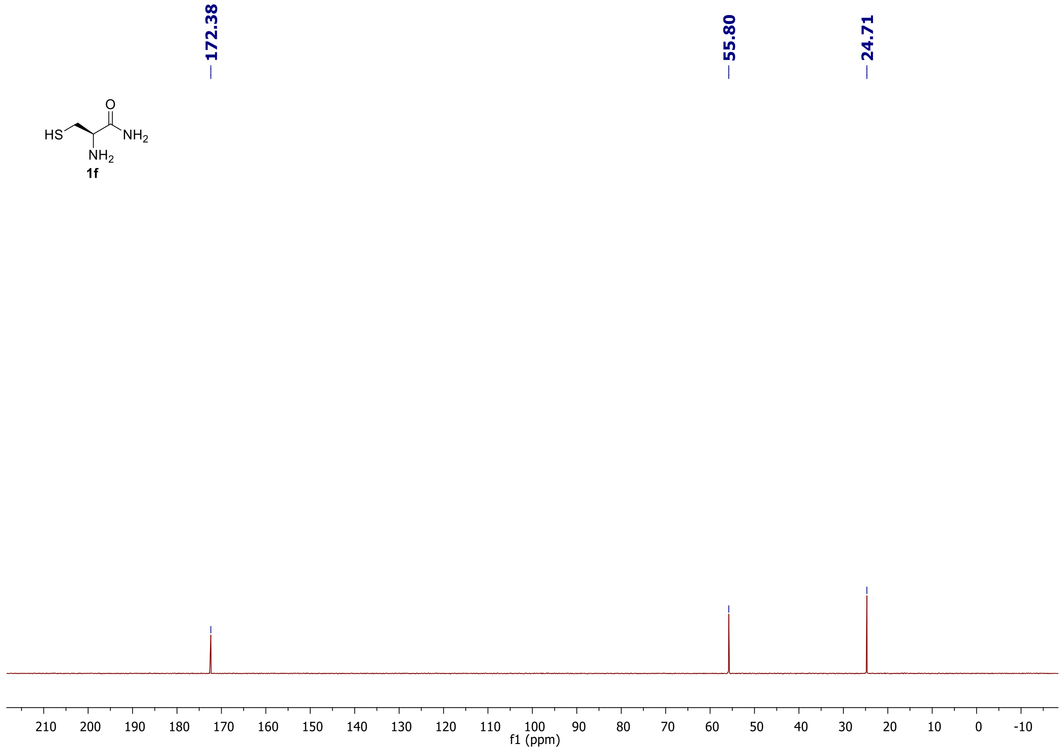

Supplementary Figure 123. ^{13}C NMR spectrum in D_2O of compound **1f**

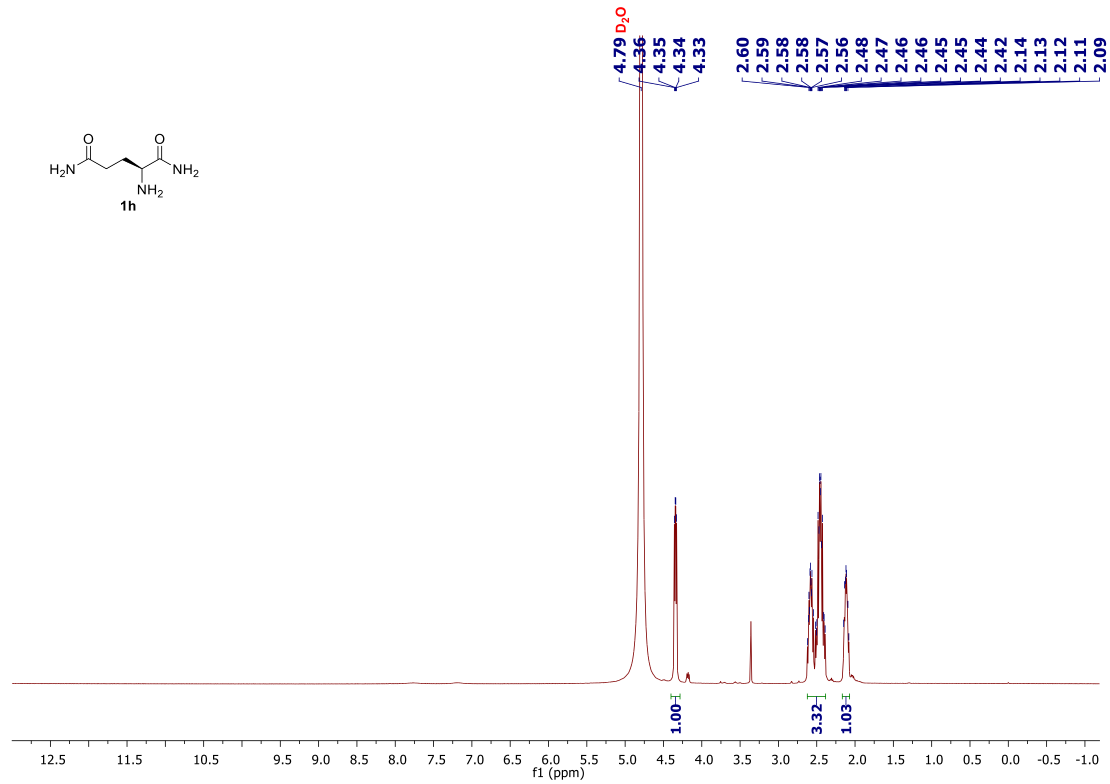

Supplementary Figure 124. ^{1}H NMR spectrum in D_2O of compound **1h**

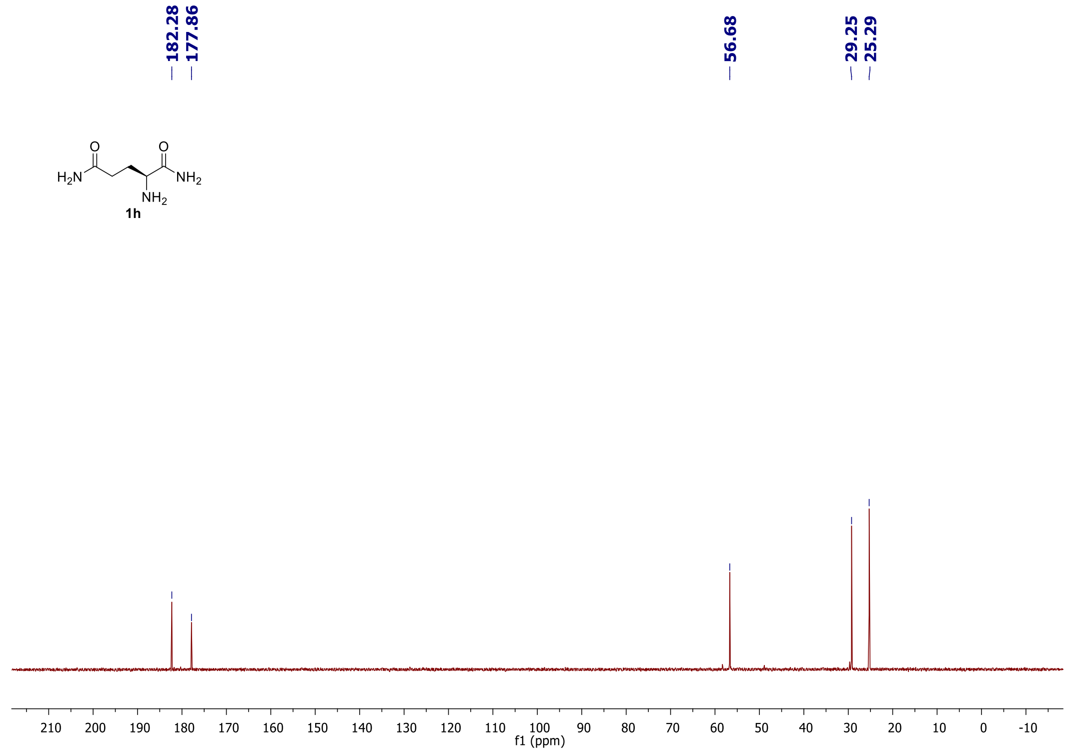

Supplementary Figure 125. ^{13}C NMR spectrum in D_2O of compound **1h**

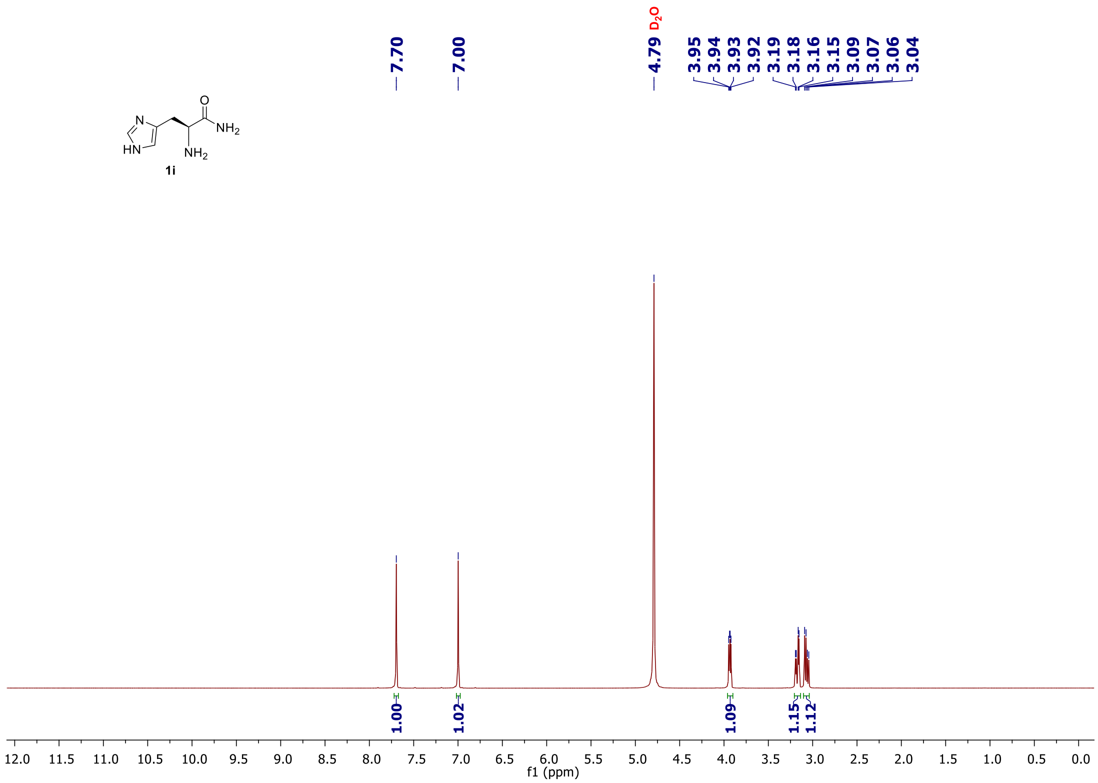

Supplementary Figure 126. ^{1}H NMR spectrum in D_2O of compound **1i**

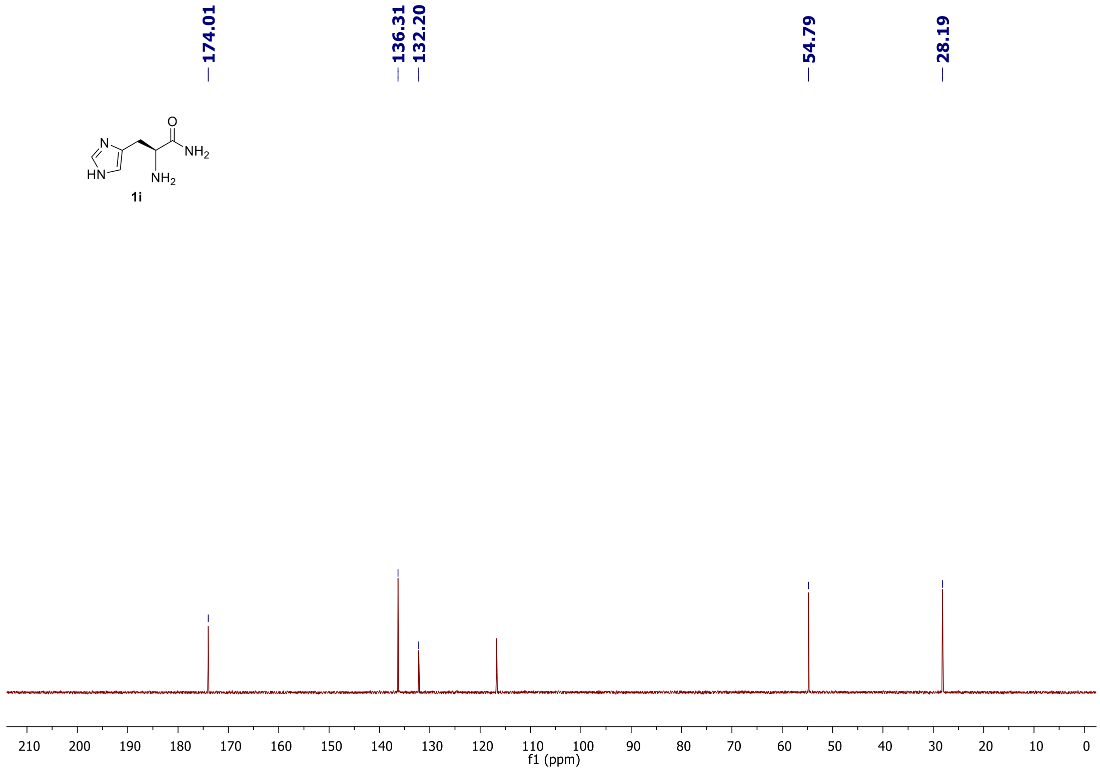

Supplementary Figure 127. ^{13}C NMR spectrum in D_2O of compound **1i**

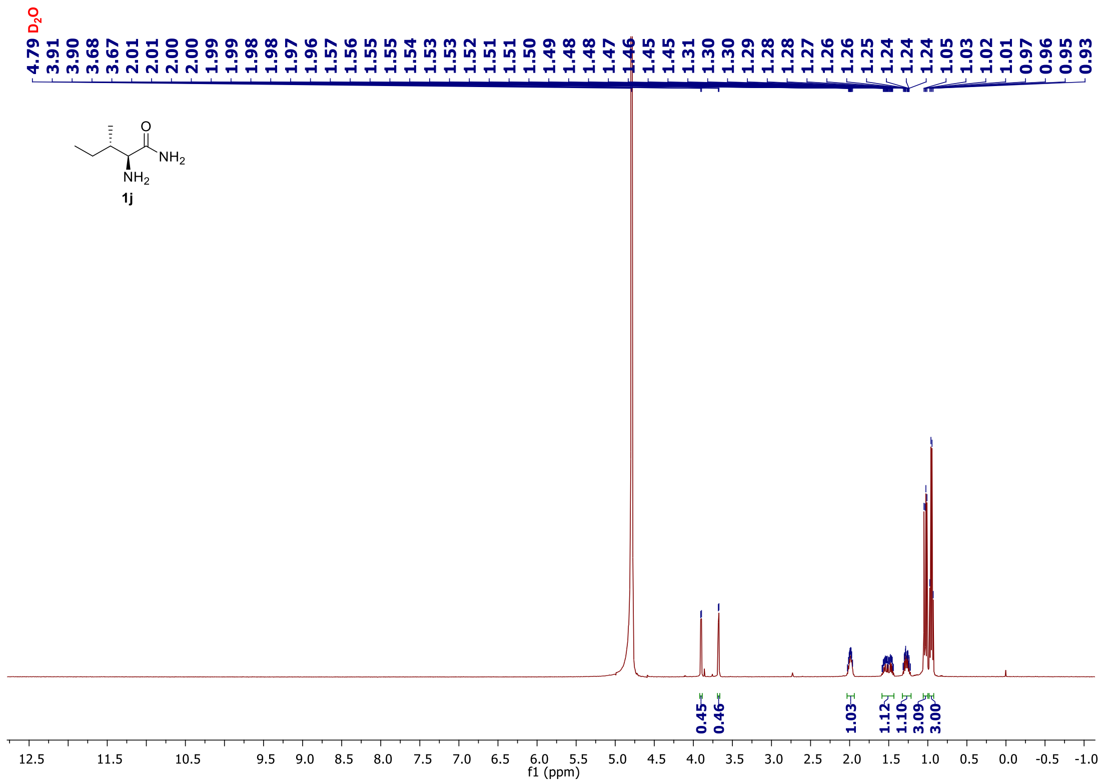

Supplementary Figure 128. ^{1}H NMR spectrum in D_2O of compound **1j**

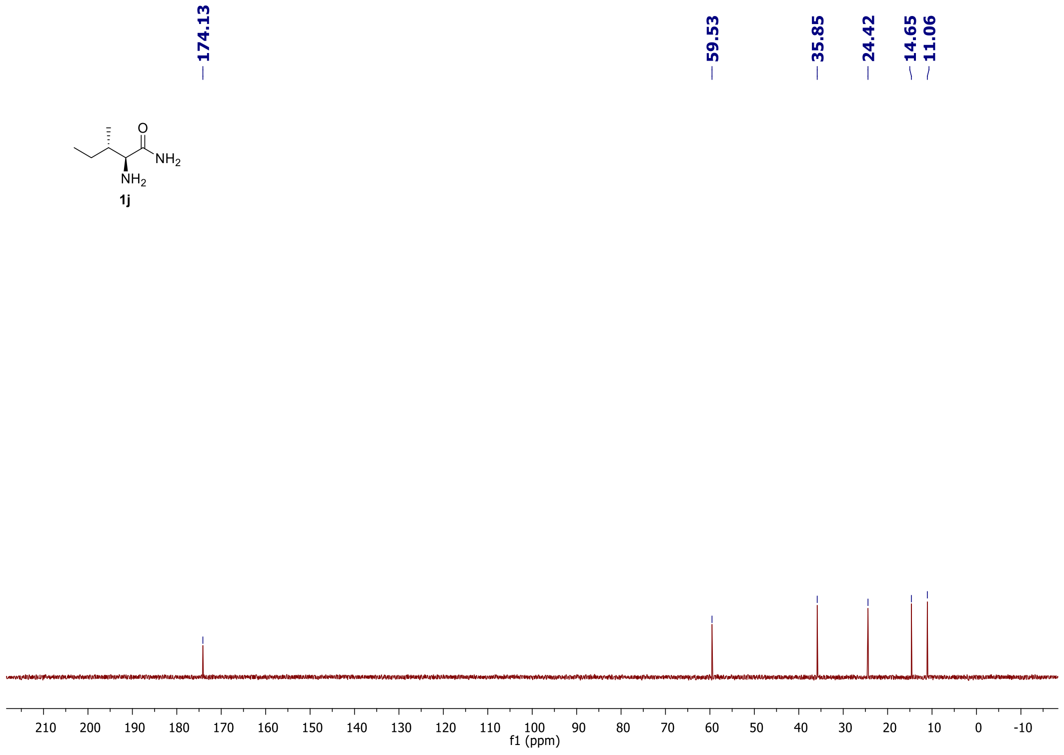

Supplementary Figure 129. ^{13}C NMR spectrum in D_2O of compound 1**j**

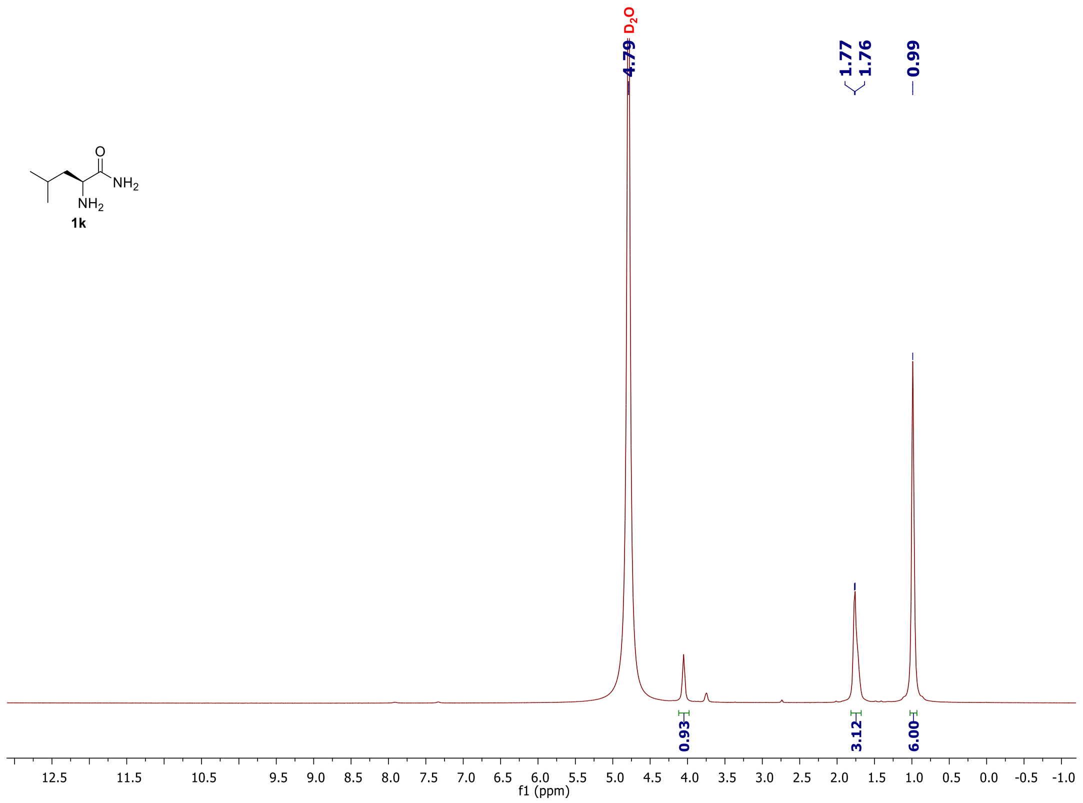

Supplementary Figure 130. ^{1}H NMR spectrum in D_2O of compound **1k**

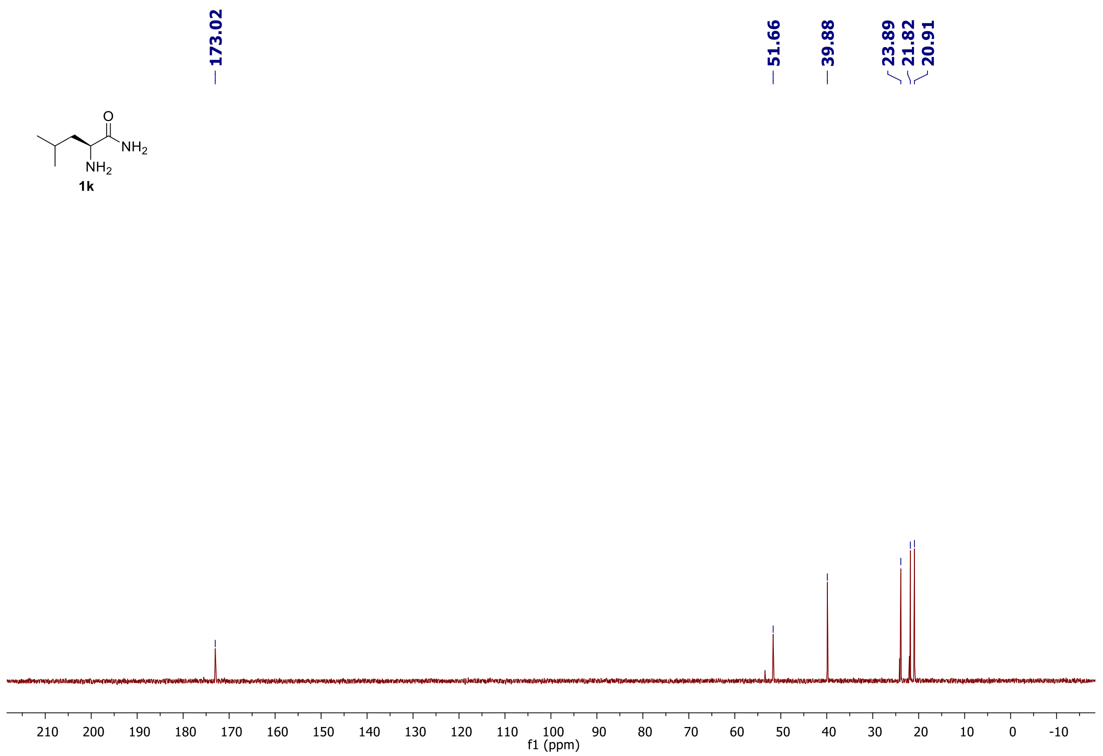

Supplementary Figure 131. ^{13}C NMR spectrum in D_2O of compound **1k**

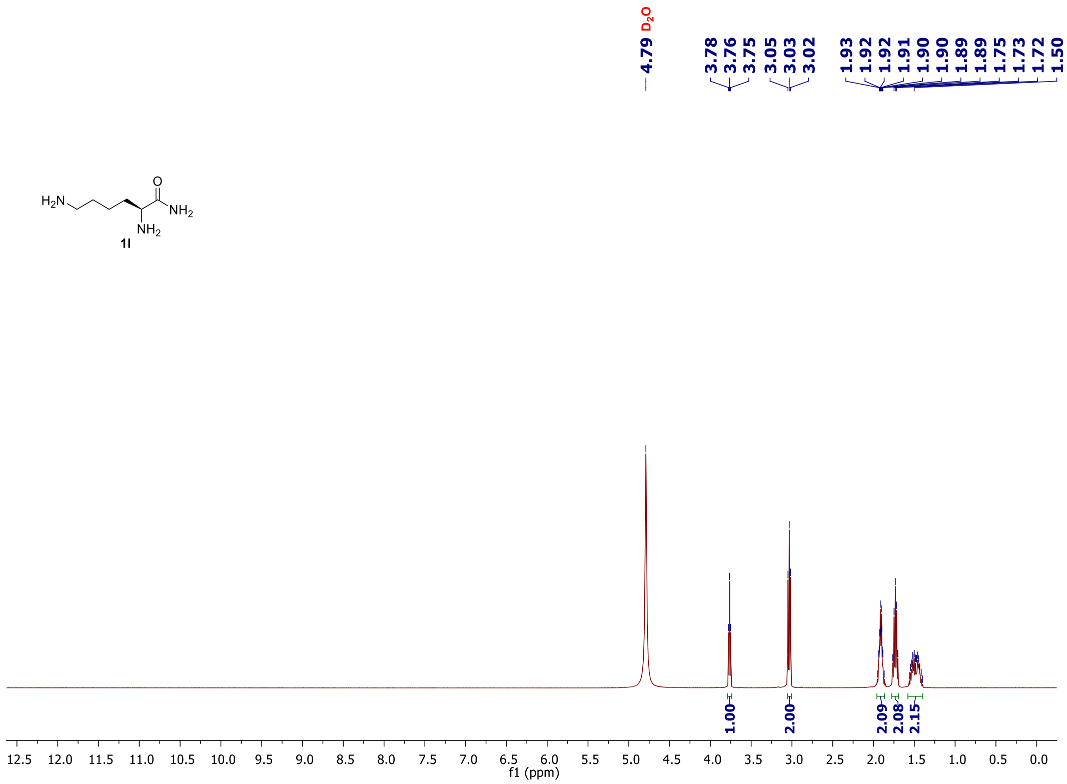

Supplementary Figure 132. ^{1}H NMR spectrum in D_2O of compound **1l**

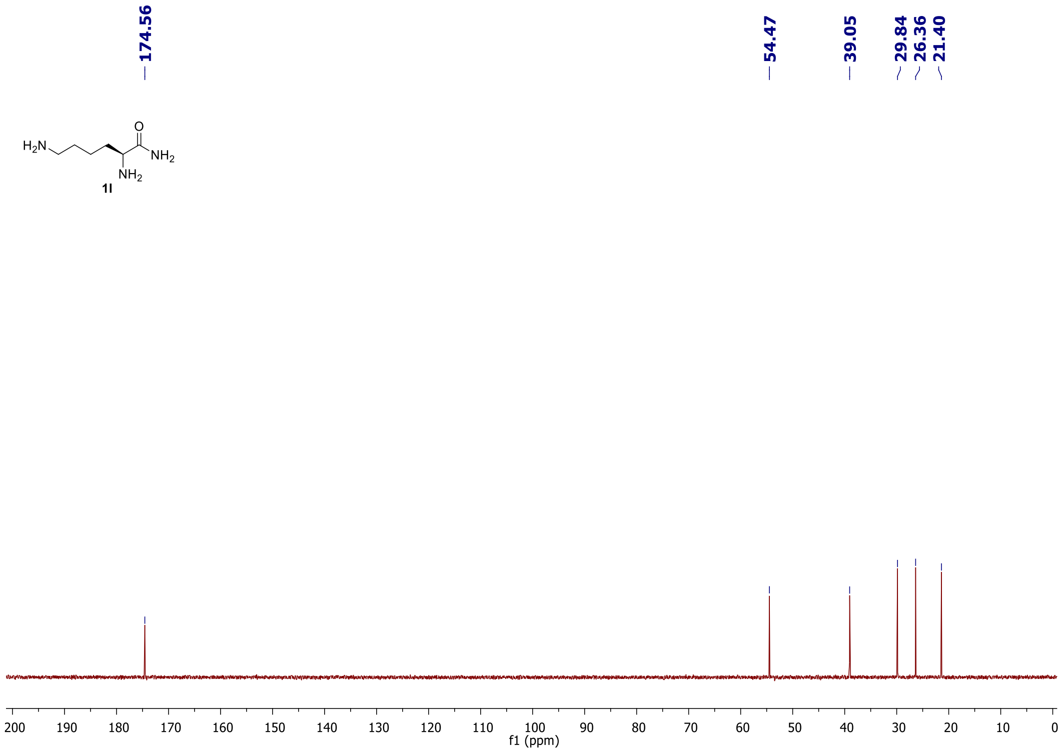

Supplementary Figure 133. ^{13}C NMR spectrum in D_2O of compound **1l**

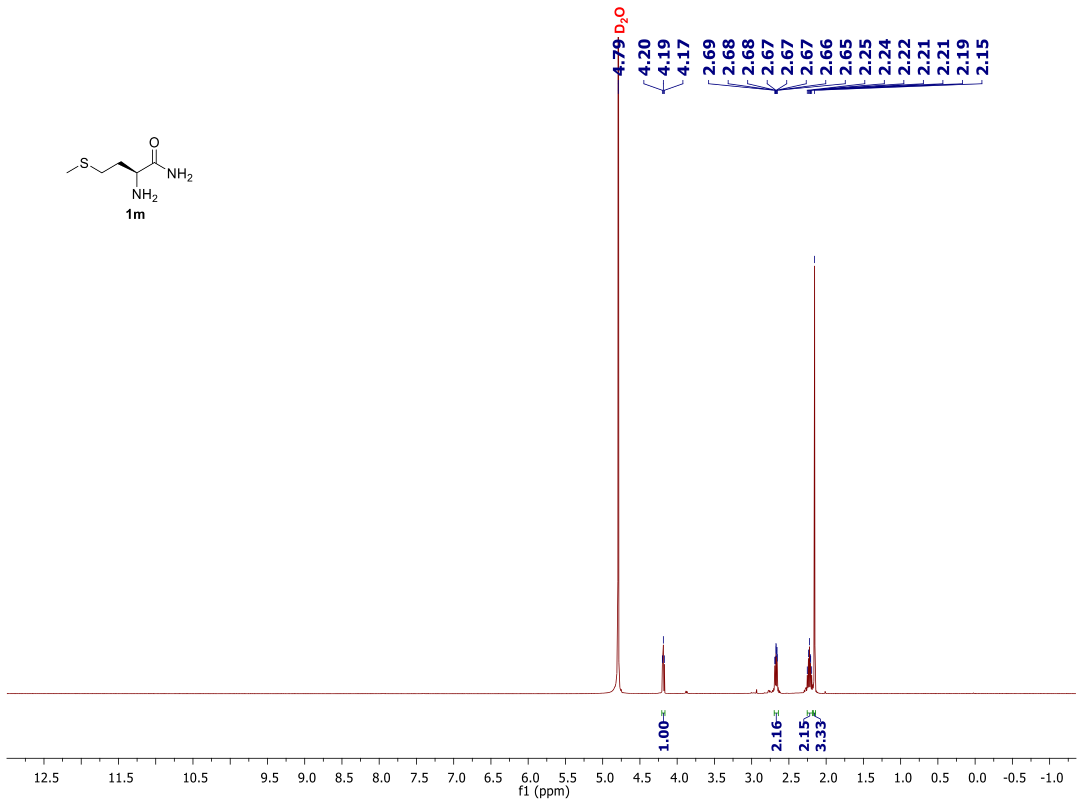

Supplementary Figure 134. ^{1}H NMR spectrum in D_2O of compound **1m**

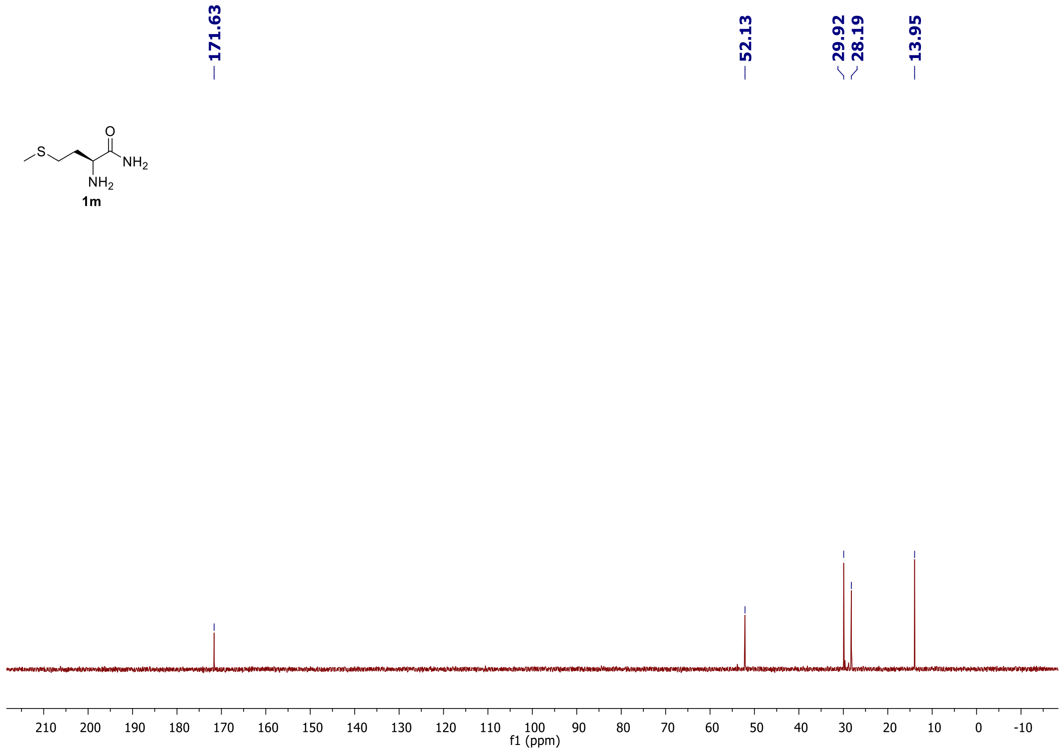

Supplementary Figure 135. ^{13}C NMR spectrum in D_2O of compound **1m**

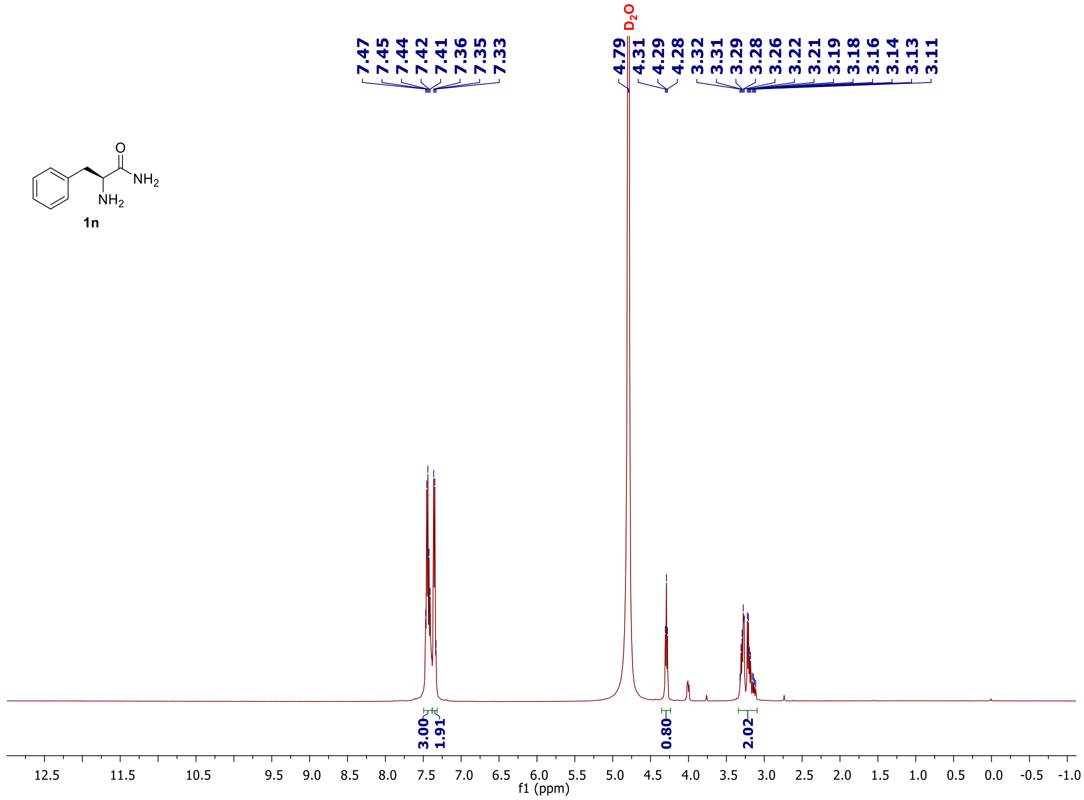

Supplementary Figure 136. ^{1}H NMR spectrum in D_2O of compound **1n**

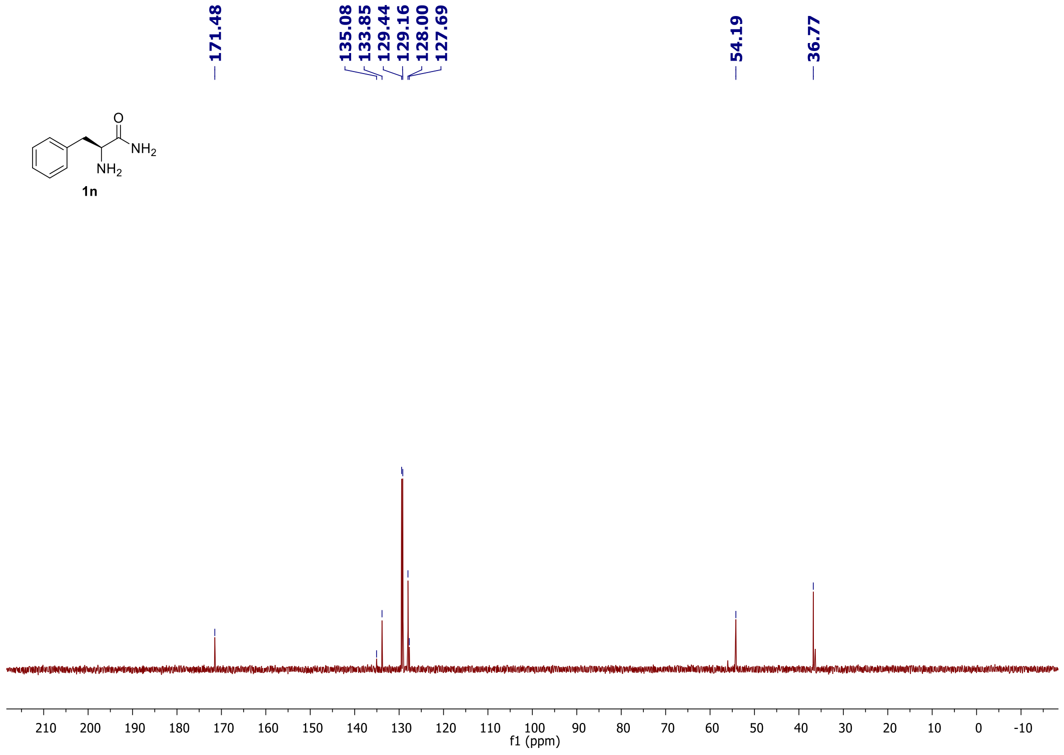

Supplementary Figure 137. ^{13}C NMR spectrum in D_2O of compound **1n**

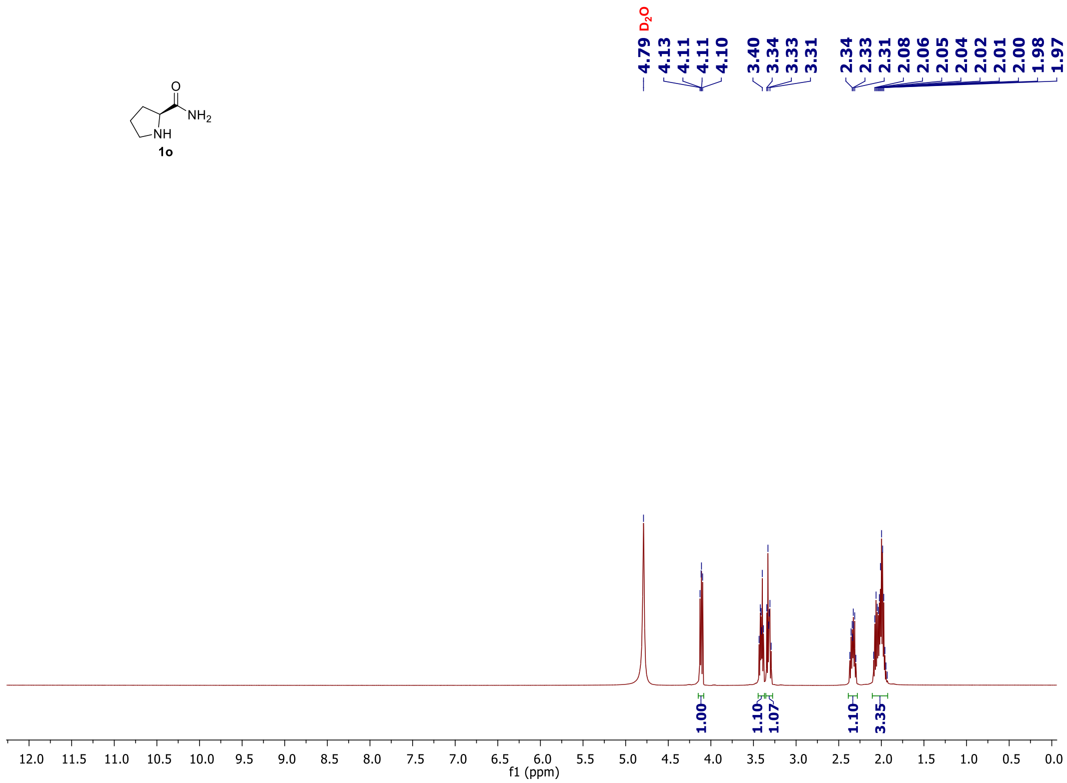

Supplementary Figure 138. ^{1}H NMR spectrum in D_2O of compound **1o**

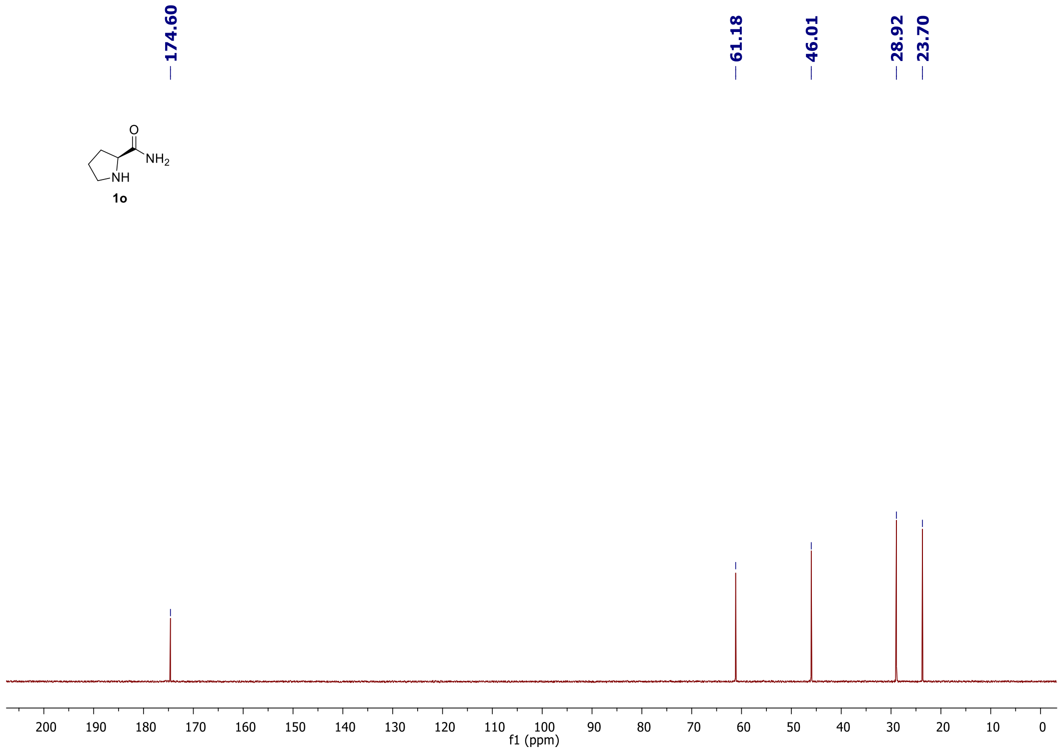

Supplementary Figure 139. ^{13}C NMR spectrum in D_2O of compound **1o**

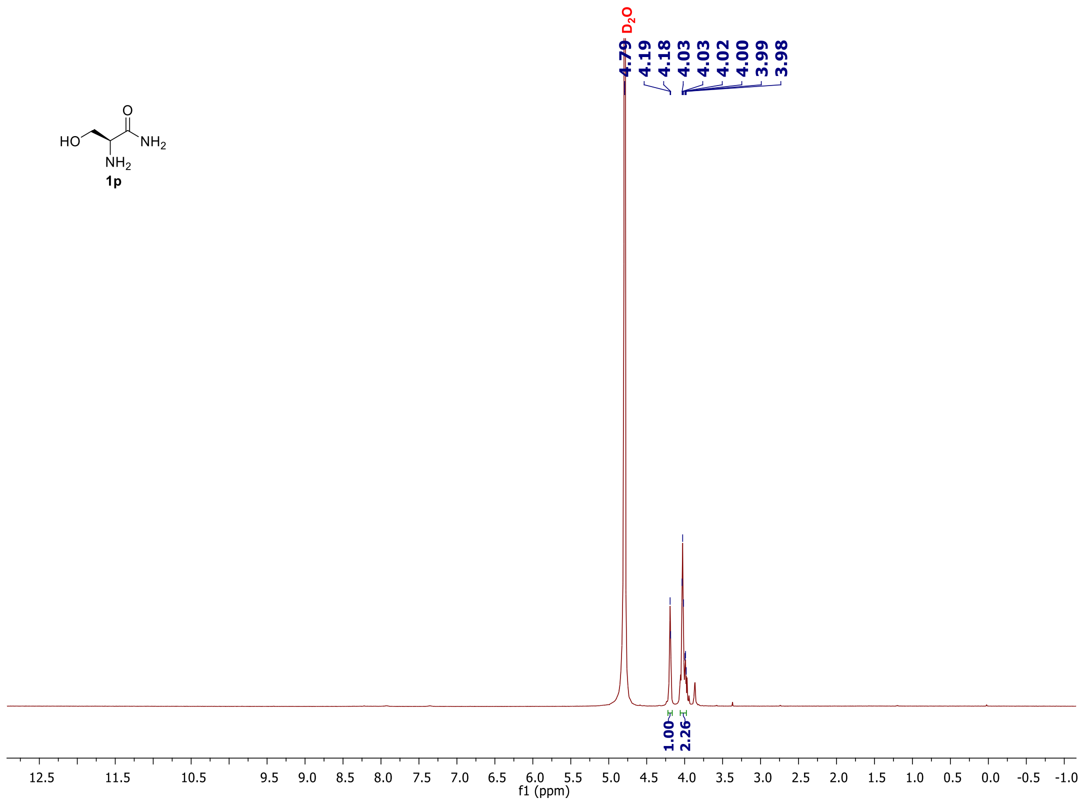

Supplementary Figure 140. ^{1}H NMR spectrum in D_2O of compound **1p**

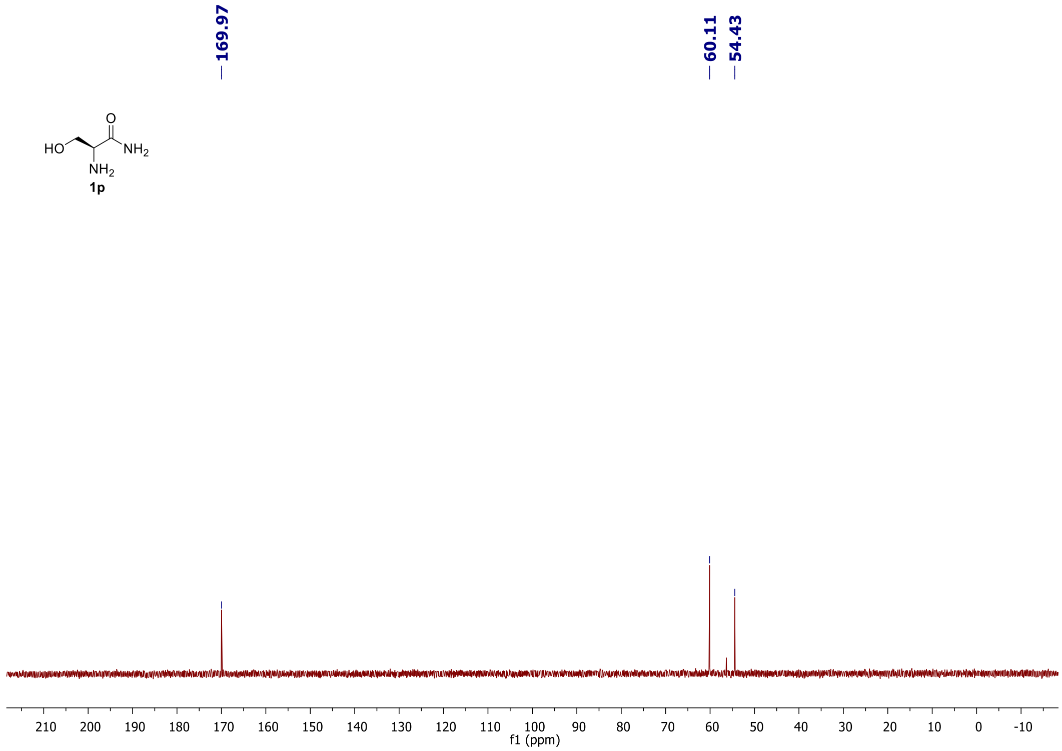

Supplementary Figure 141. ^{13}C NMR spectrum in D_2O of compound **1p**

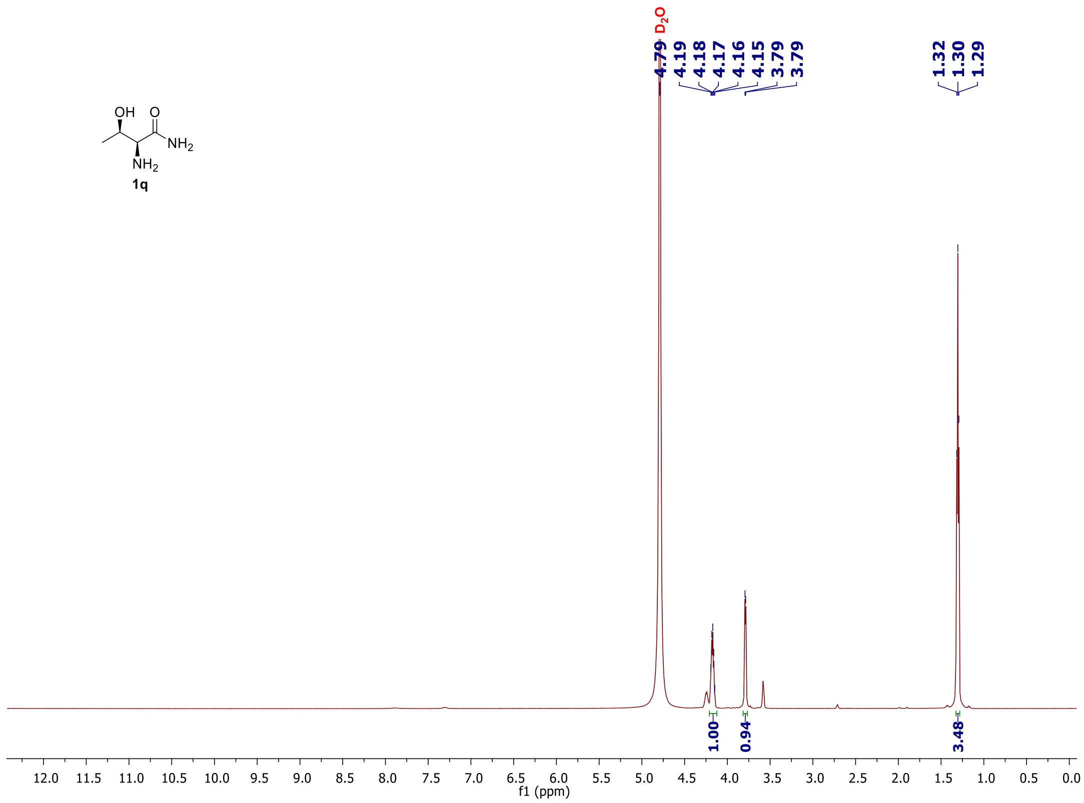

Supplementary Figure 142. ^{1}H NMR spectrum in D_2O of compound **1q**

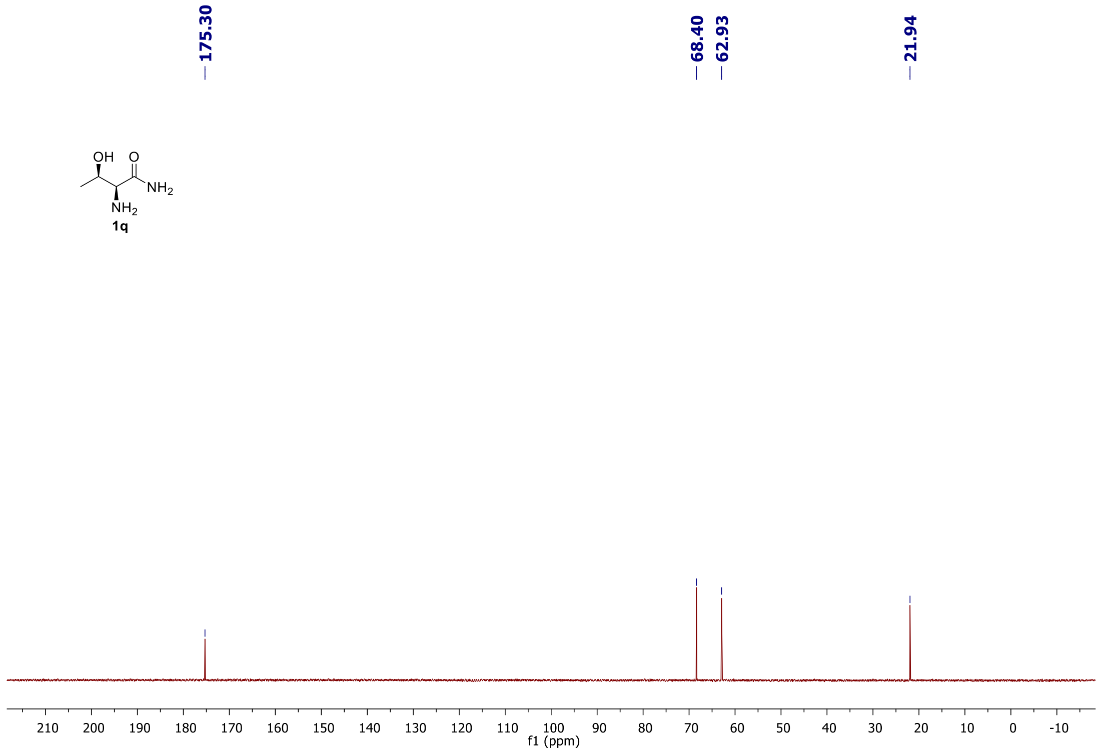

Supplementary Figure 143. ^{13}C NMR spectrum in D_2O of compound **1q**

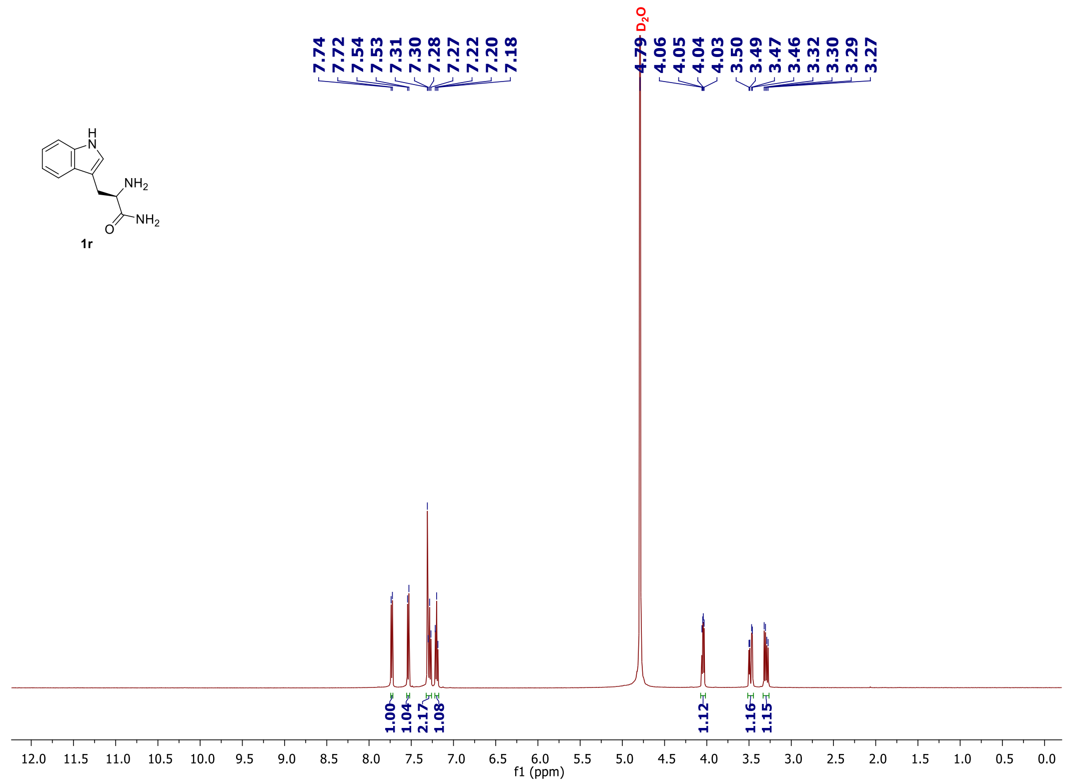

Supplementary Figure 144. ^{1}H NMR spectrum in D_2O of compound **1r**

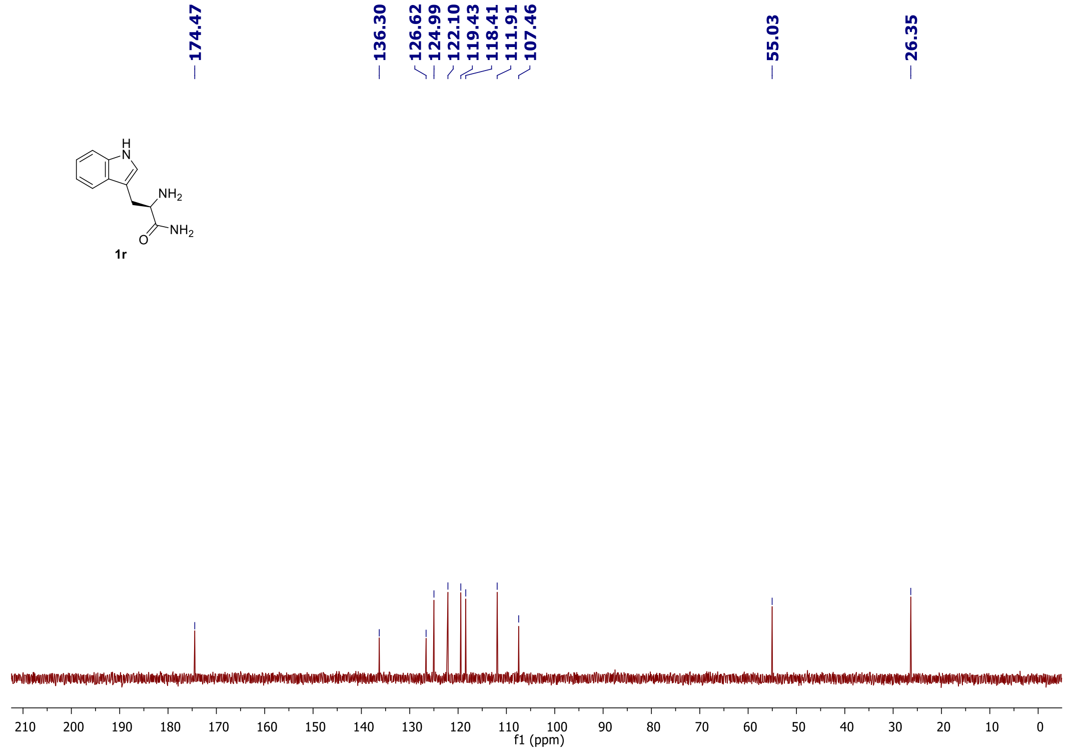

Supplementary Figure 145. ^{13}C NMR spectrum in D_2O of compound **1r**

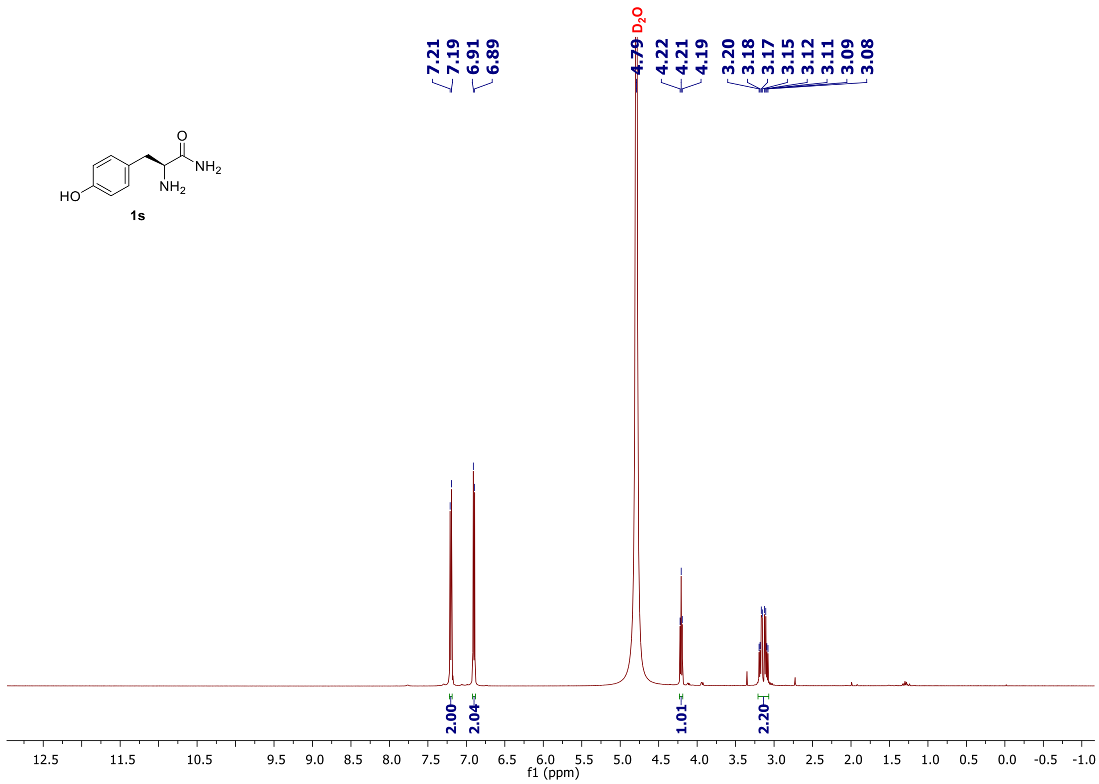

Supplementary Figure 146. ^{1}H NMR spectrum in D_2O of compound **1s**

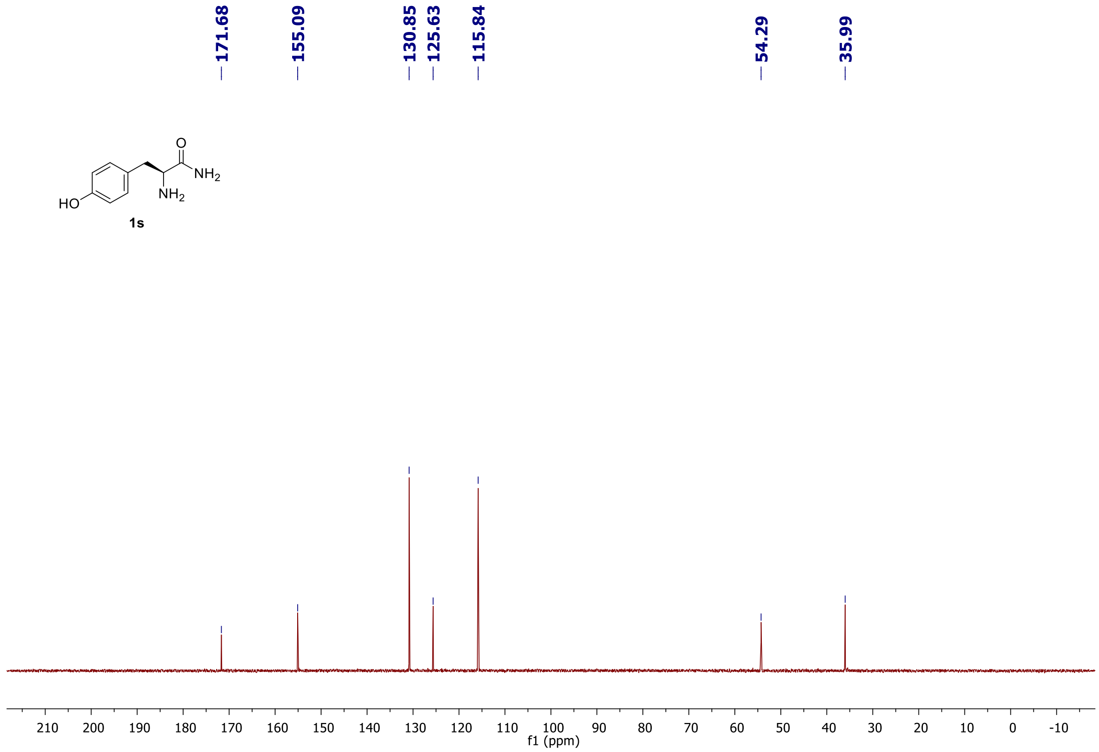

Supplementary Figure 147. ^{13}C NMR spectrum in D_2O of compound **1s**

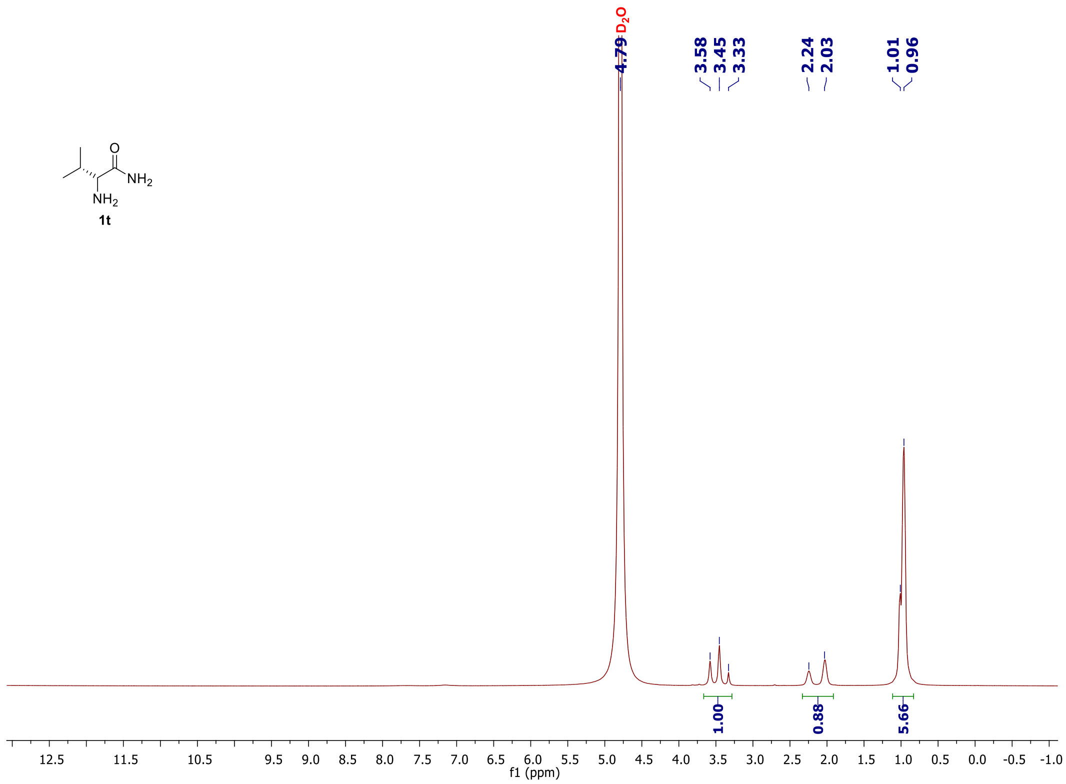

Supplementary Figure 148. ^{1}H NMR spectrum in D_2O of compound **1t**

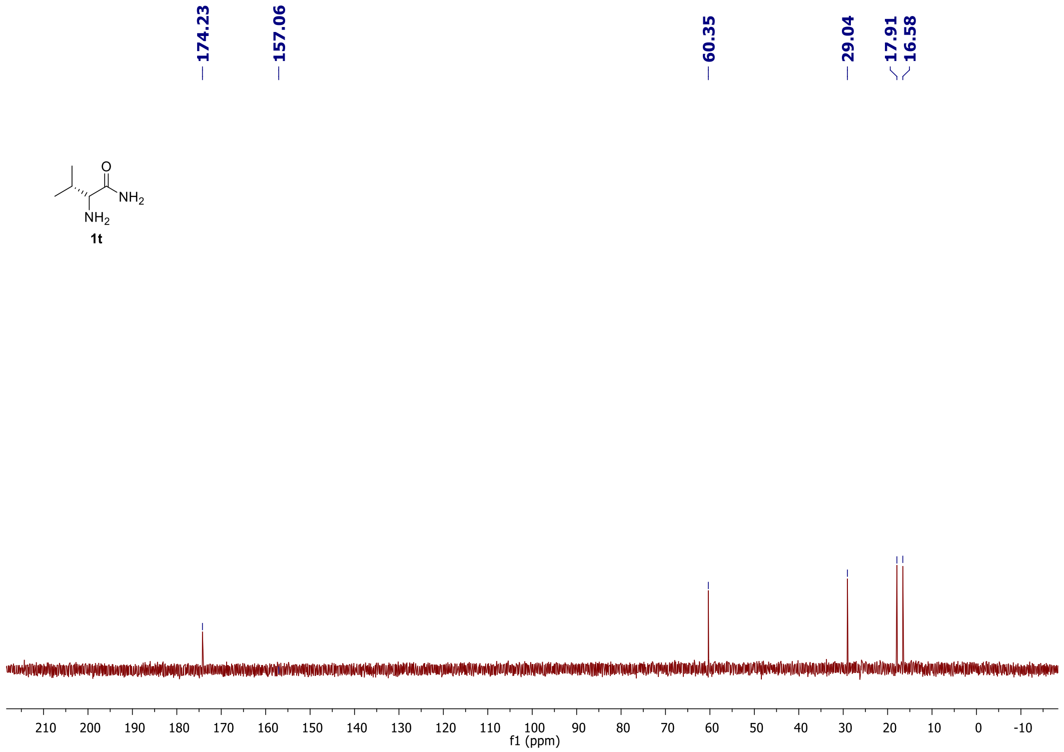

Supplementary Figure 149. ^{13}C NMR spectrum in D_2O of compound **1t**

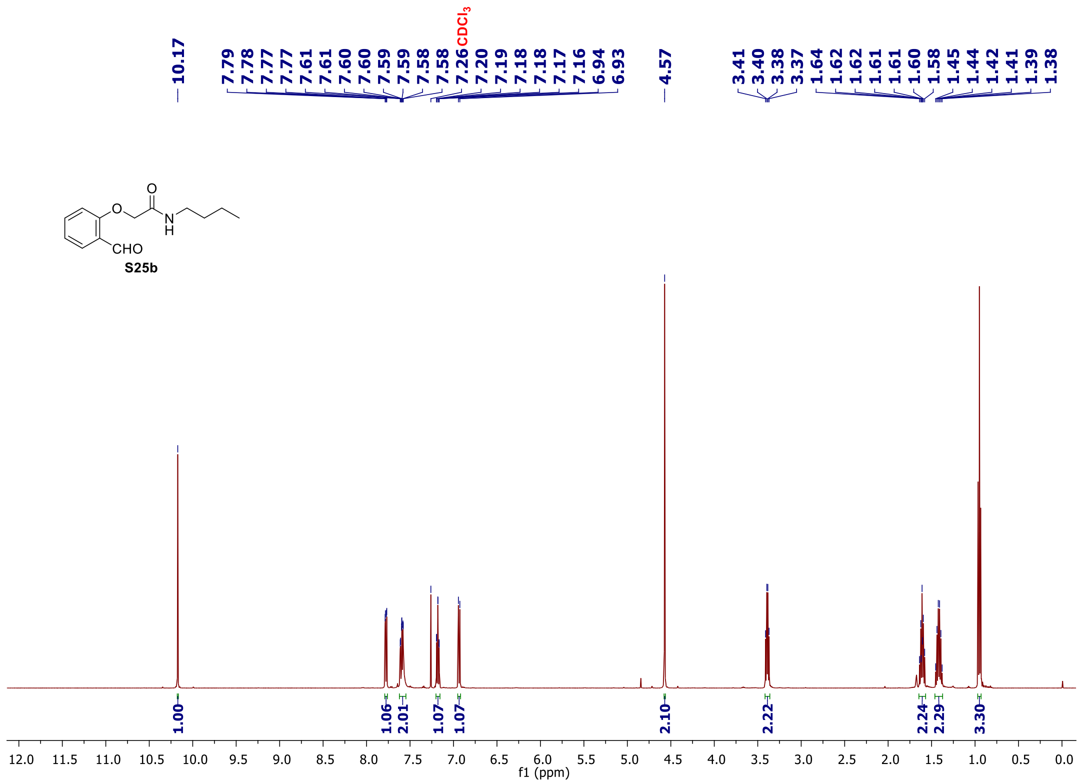

Supplementary Figure 150. ^{1}H NMR spectrum in $CDCl_3$ of compound **S25b**

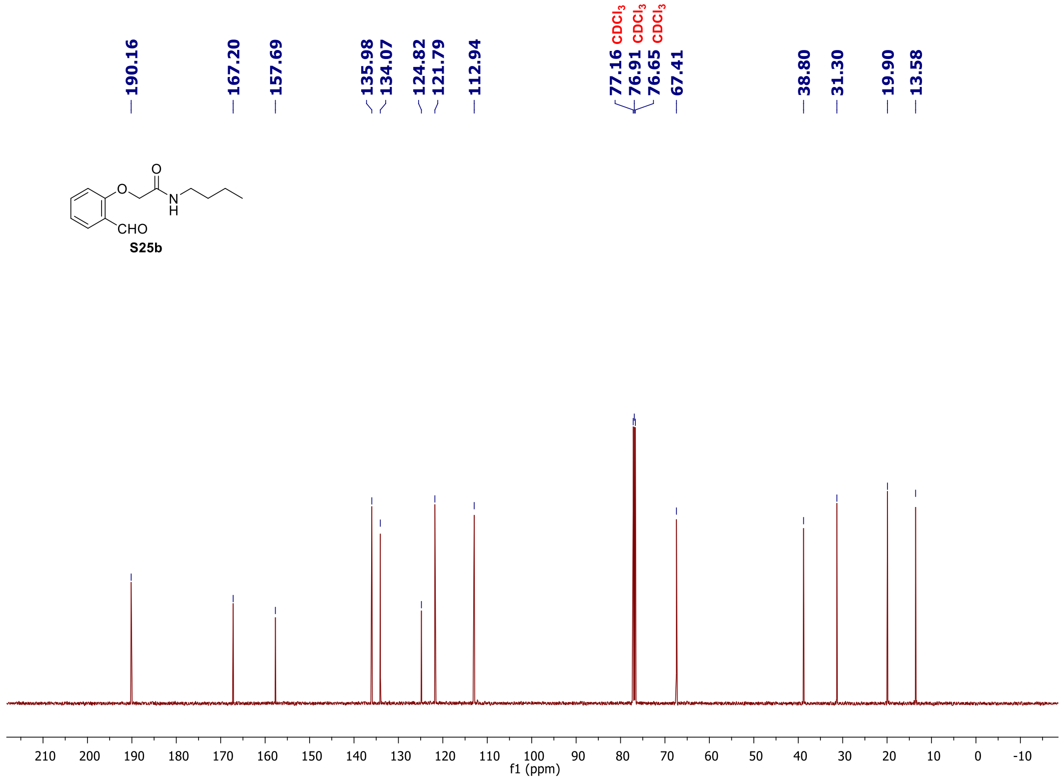

Supplementary Figure 151. ^{13}C NMR spectrum in $CDCl_3$ of compound **S25b**

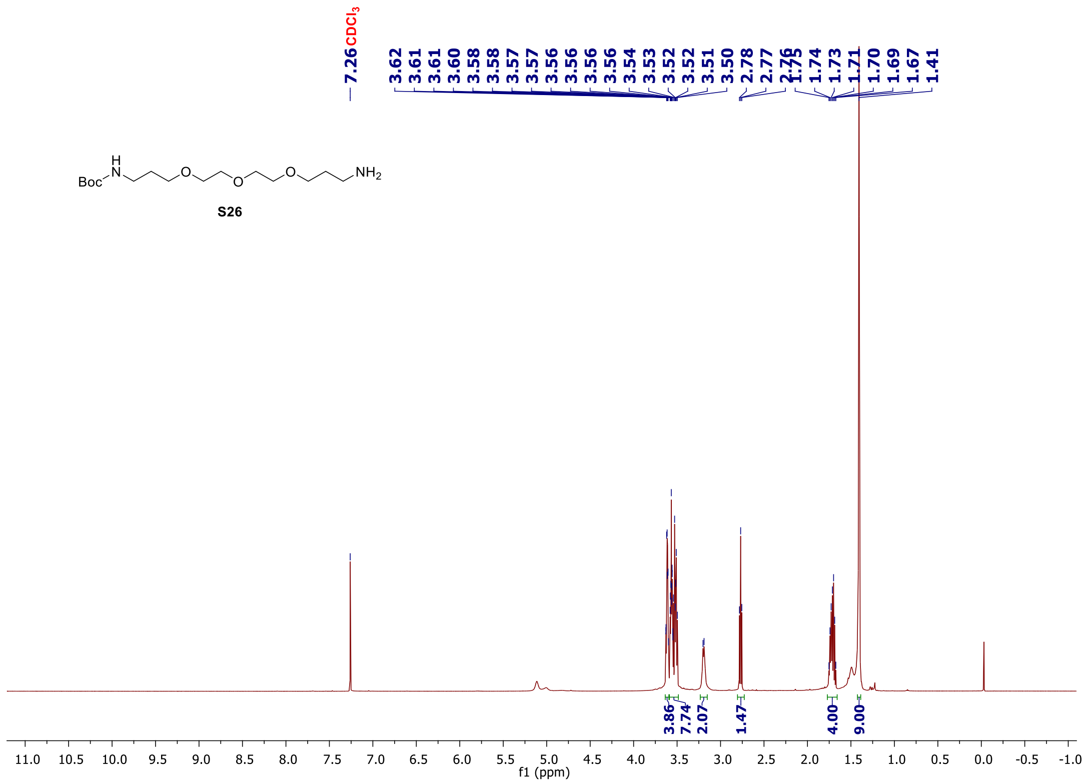

Supplementary Figure 152. ^{1}H NMR spectrum in $CDCl_3$ of compound **S26**

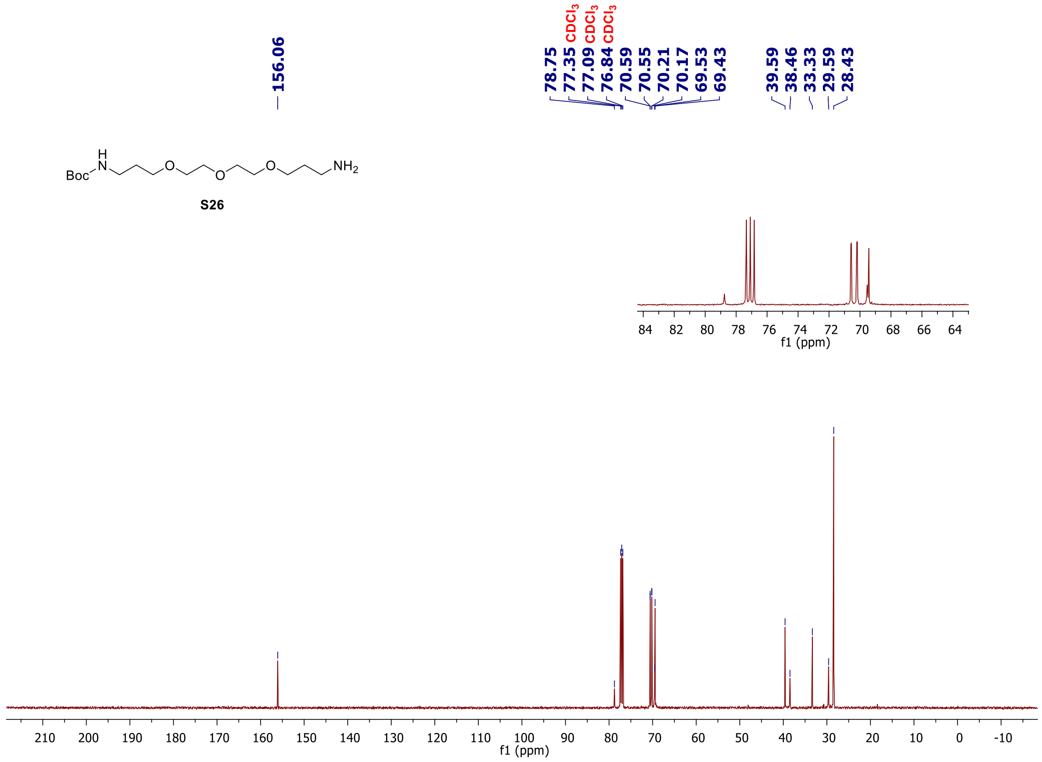

Supplementary Figure 153. ^{13}C NMR spectrum in $CDCl_3$ of compound **S26**

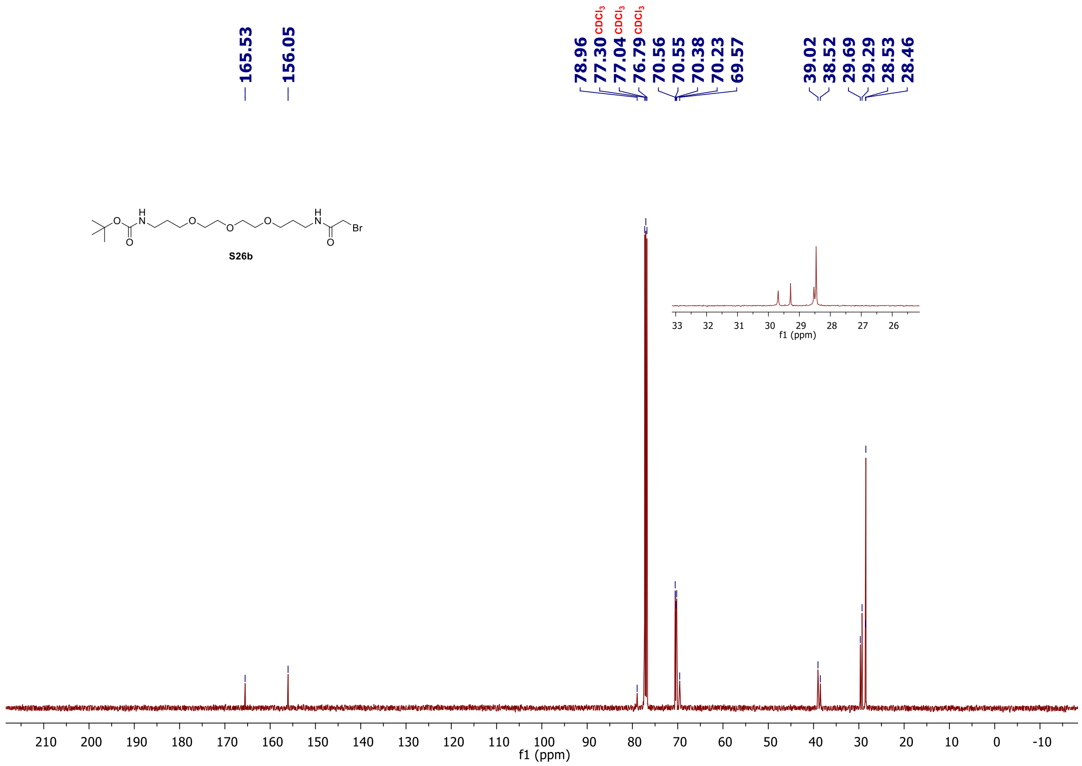

Supplementary Figure 154. ^{1}H NMR spectrum in $CDCl_3$ of compound **S26b**

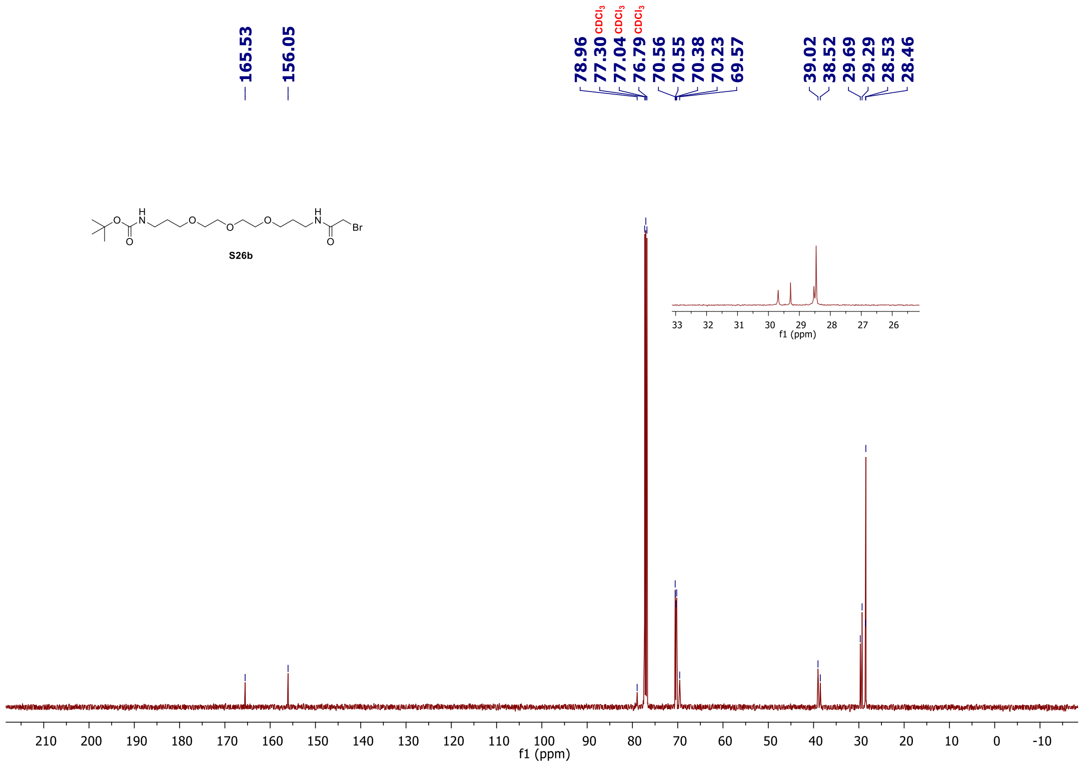

Supplementary Figure 155. ^{13}C NMR spectrum in $CDCl_3$ of compound **S26b**

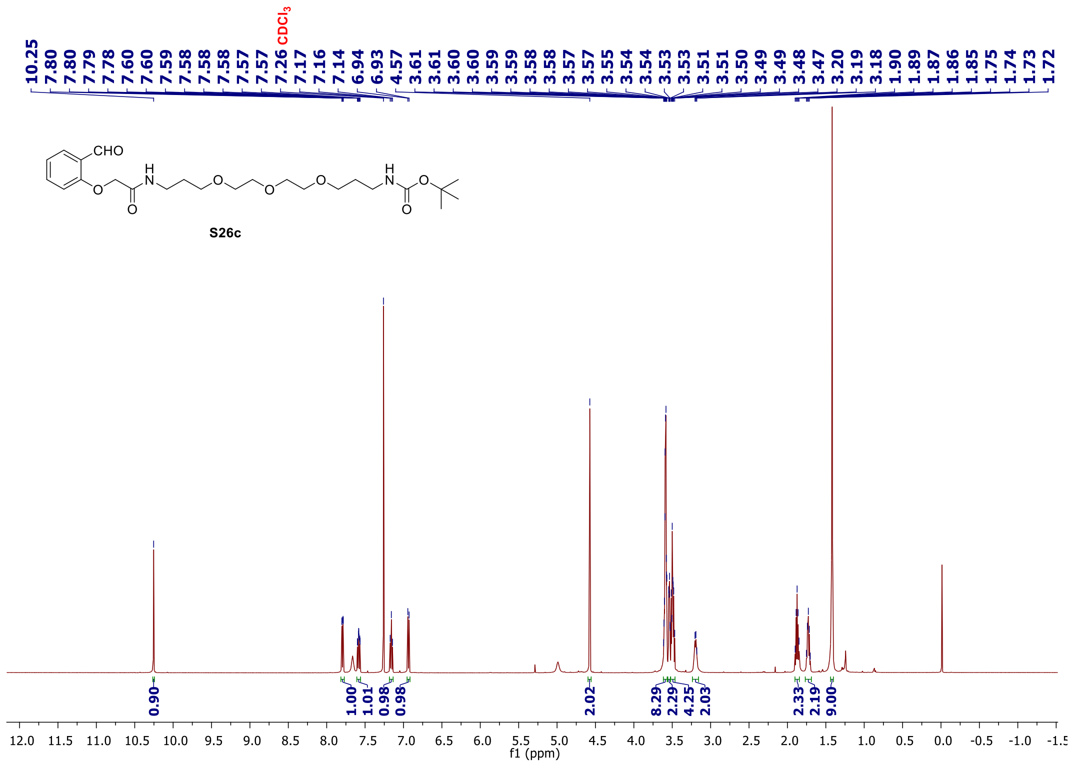

Supplementary Figure 156. ^{1}H NMR spectrum in $CDCl_3$ of compound **S26c**

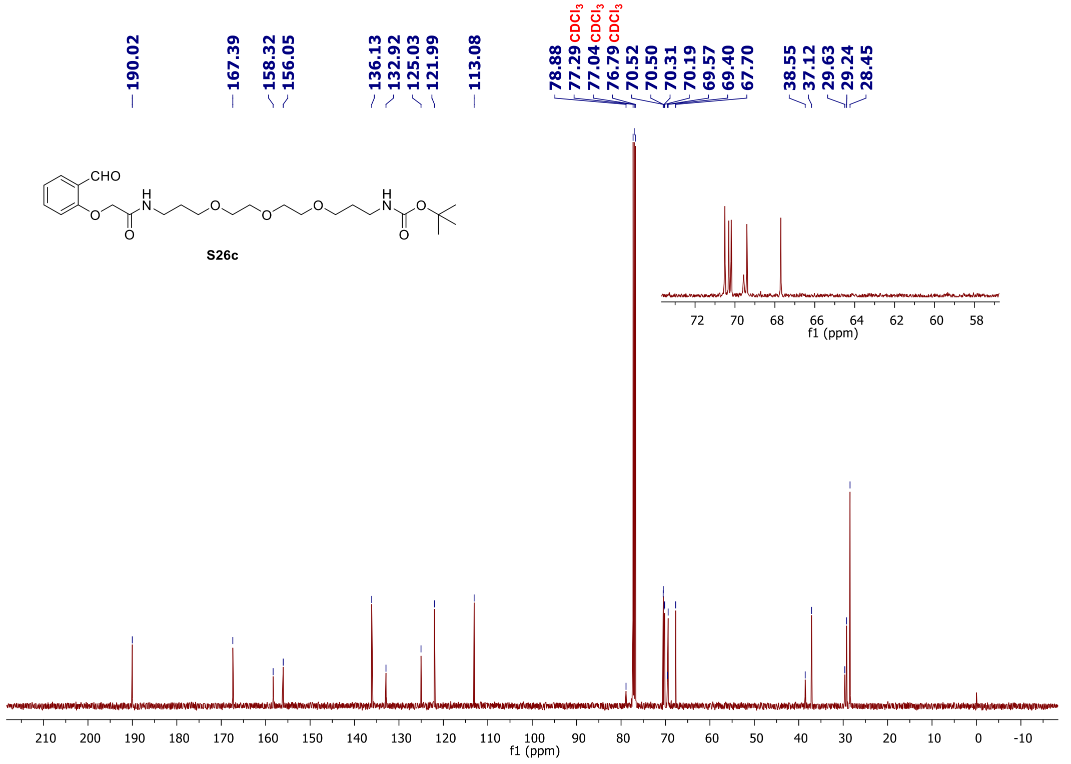

Supplementary Figure 157. ^{13}C NMR spectrum in $CDCl_3$ of compound **S26c**

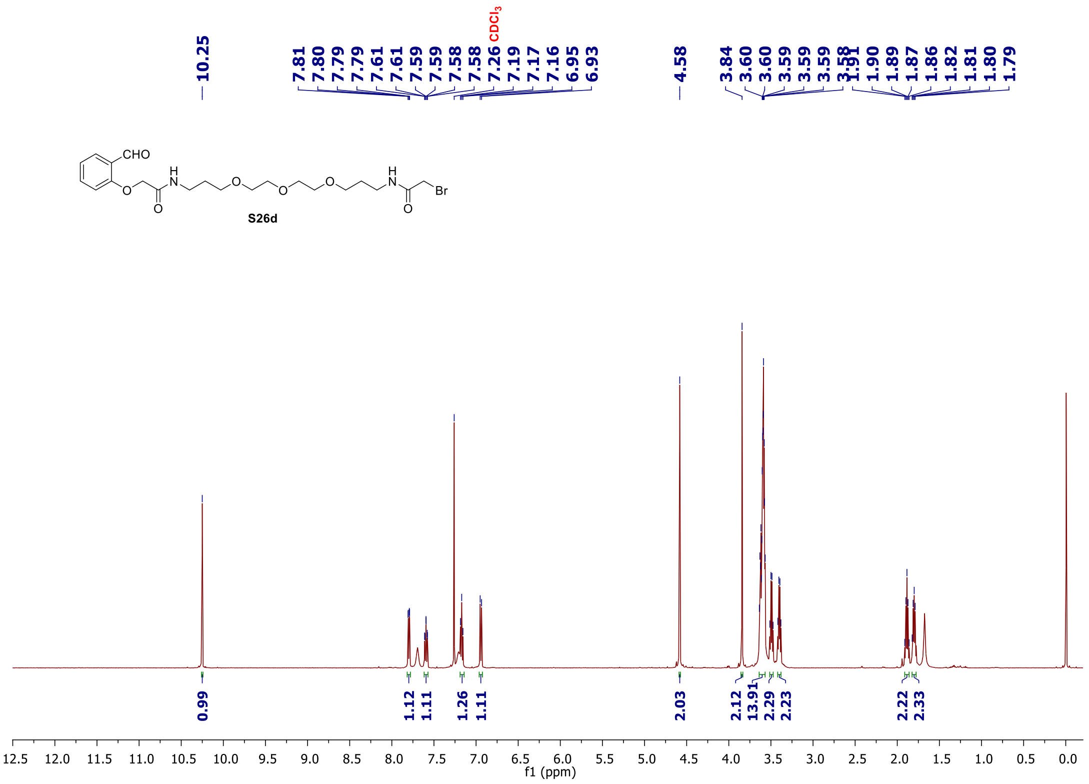

Supplementary Figure 158. ^{1}H NMR spectrum in $CDCl_3$ of compound **S26d**

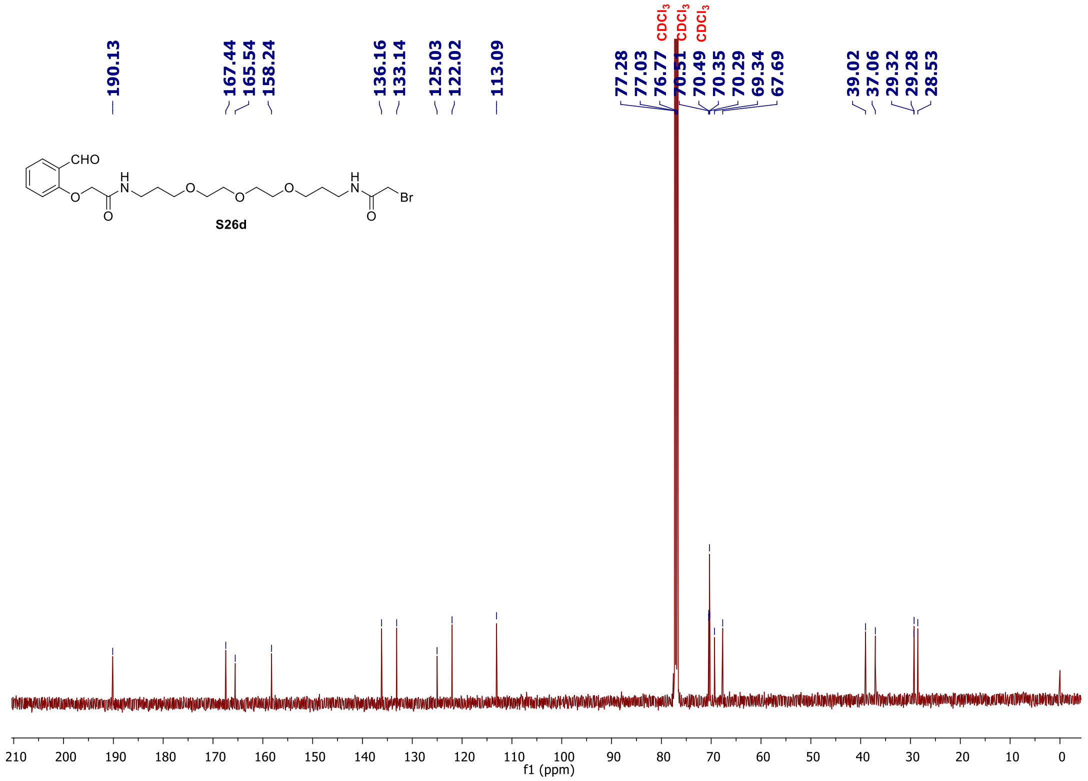

Supplementary Figure 159. ^{13}C NMR spectrum in $CDCl_3$ of compound **S26d**

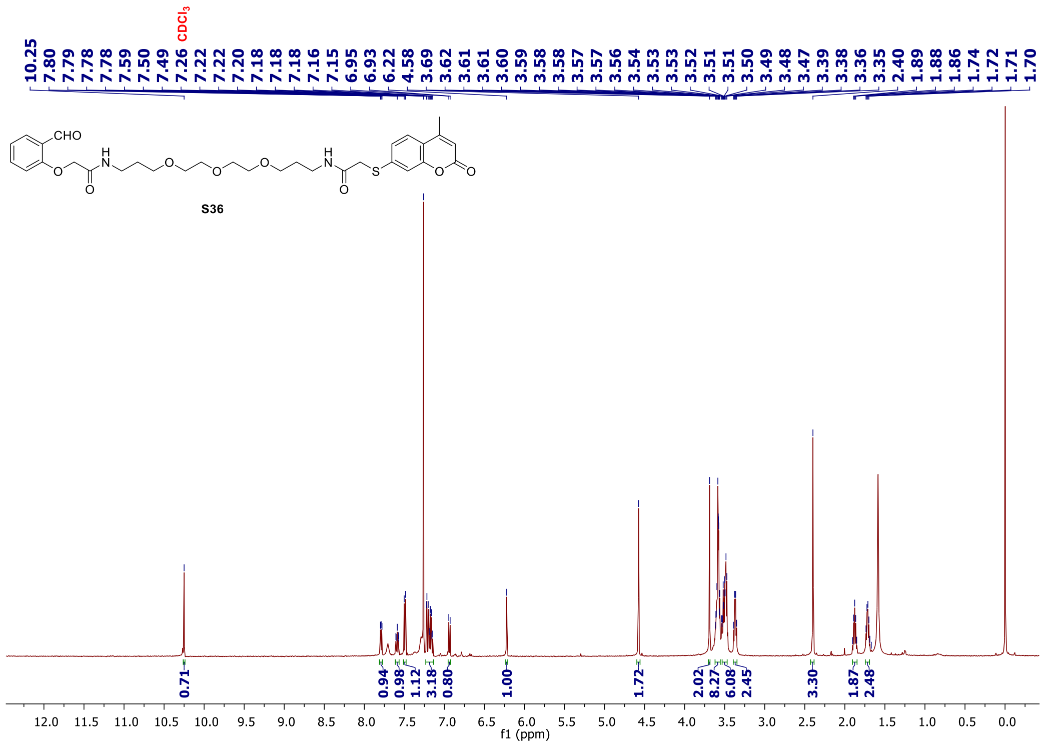

Supplementary Figure 160. ^{1}H NMR spectrum in $CDCl_3$ of compound **S36**

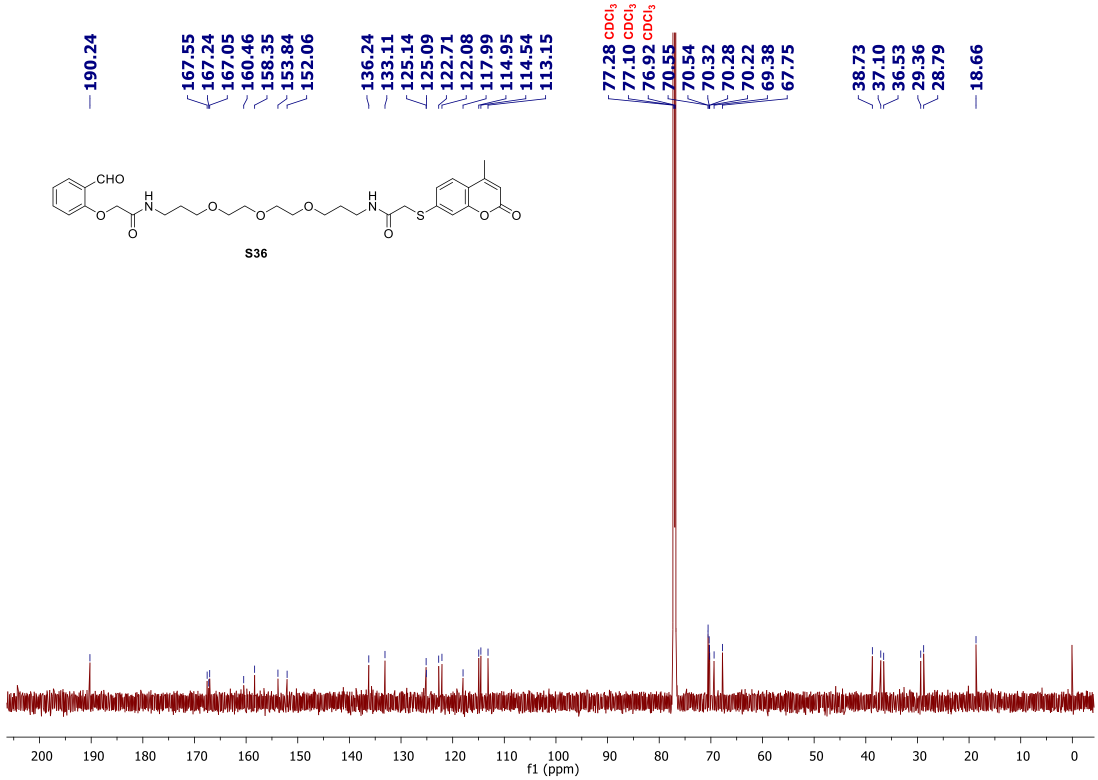

Supplementary Figure 161. ^{13}C NMR spectrum in $CDCl_3$ of compound **S36**

Supplementary Tables

Supplementary Table 1. Design of aldehyde with hydrogen bond acceptor

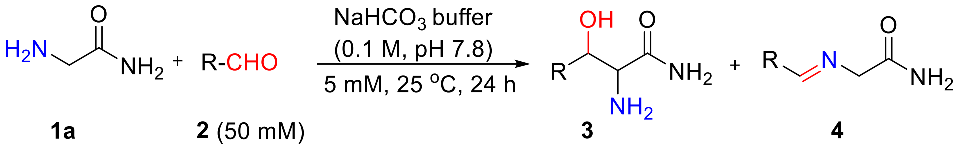

1a **2** (50 mM) **3** **4**

Entry	R	% Conversion (3)[a]	% Conversion (4)[a]
1	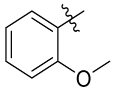	0	69
2	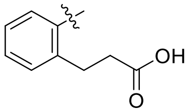	27	19
3	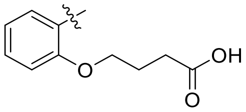	33	50
4	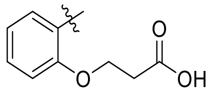	63	20
5	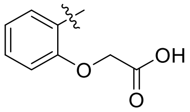	95	<5

[a] % Conversions were monitored by LC-MS. [b] The control experiment with unsubstituted benzaldehyde does not result in any isolable aminoalcohol.

Supplementary Table 2. Aminoalcohol formation with amino acid amide

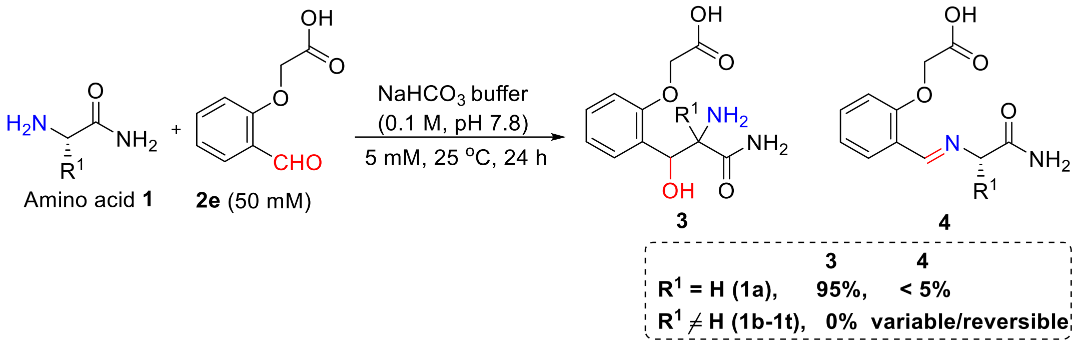

Entry	Amino acid amide 1	% Conversion 3	% Conversion 4
1	Gly **1a**	>95	<5
2	Ala **1b**	0	99
3	Arg **1c**	0	99
4	Asn **1d**	0	>99
5	Asp **1e**	0	>99
6	Cys **1f**	0	>99
7	Glu **1g**	0	95
8	Gln **1h**	0	>99
9	His **1i**	0	98
10	Ile **1j**	0	98
11	Leu **1k**	0	98
12	Lys **1l**	0	96
13	Met **1m**	0	94
14	Phe **1n**	0	78
15	Pro **1o**	0	>99
16	Ser **1p**	0	94
17	Thr **1q**	0	95
18	Trp **1r**	0	98
19	Tyr **1s**	0	90
20	Val **1t**	0	98

% Conversions were monitored by LC-MS.

Supplementary Table 3. Optimization of the stoichiometry of reagent **2g**

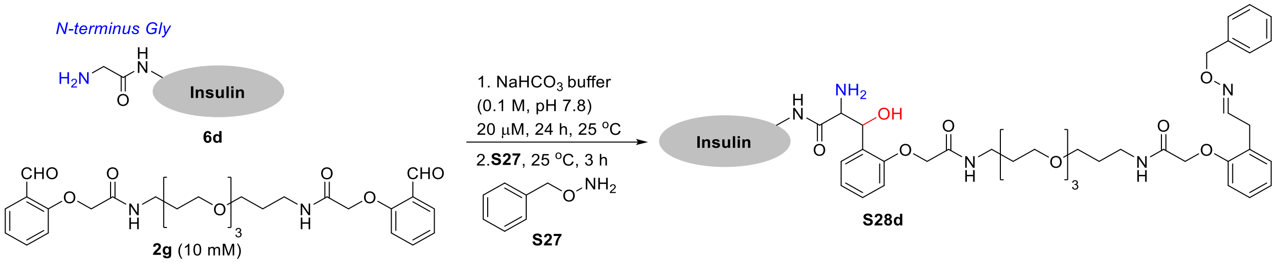

Entry	Reagent **2g** (equiv.)	% Conversion **S28d**
1	10	23
2	50	41
3	100	53
4	300	75
5	500	88

% Conversion was monitored by ESI-MS.

Supplementary Table 4. pH screening for aminoalcohol formation

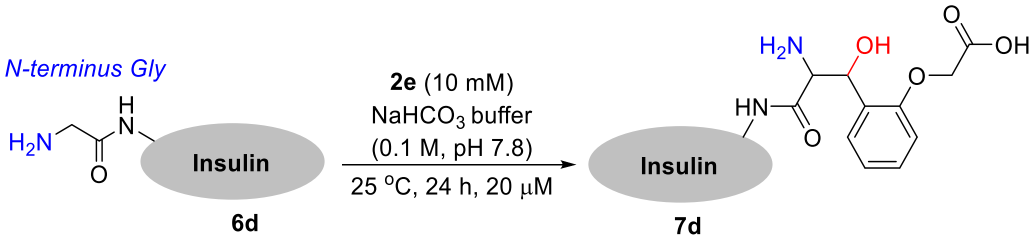

Entry	$NaHCO_3$ buffer (0.1 M)	% Conversion
1	pH 6.0	50
2	pH 7.0	63
3	pH 7.8	71
4	pH 9.0	71

% Conversions were monitored by ESI-MS.

Supplementary Methods

Synthesis of 2-amino-3-(2-(2-(butylamino)-2-oxoethoxy)phenyl)-3-hydroxypropanamide S30. In a 25 ml round bottom flask, glycine amide **1a** (100 mg, 1.35 mmol) was dissolved in sodium bicarbonate buffer (7 ml, 0.1 M, pH 7.8). To this solution, n-butyl-2-(2-formylphenoxy)acetamide **S25b** (3.1 g, 13.5 mmol) in DMF (3 ml) from a freshly prepared stock solution was added, and the reaction mixture was allowed to stir at room temperature. After 24 h, the reaction mixture was concentrated by lyophilization. The product was purified using flash column chromatography (MeOH:$CHCl_3$ 5:95) to afford 2-amino-3-(2-(2-(butylamino)-2-oxoethoxy)phenyl)-3-hydroxypropanamide **S30** (18% yield, 75 mg). ^{1}H NMR (500 MHz, CD_3OD) δ 7.45 (d, *J* = 7.5 Hz, 1H), 7.22 (t, *J* = 7.4 Hz, 1H), 6.97 (t, *J* = 7.5 Hz, 1H), 6.78 (d, *J* = 8.3 Hz, 1H), 5.28 (d, *J* = 5.3 Hz, 1H), 4.61 (dd, *J* = 39.0, 15.3 Hz, 2H), 4.30 (d, *J* = 5.3 Hz, 1H), 3.14 (t, *J* = 7.0 Hz, 2H), 1.42 -1.37 (m, 2H), 1.24 (m, 2H), 0.82 (t, *J* = 7.4 Hz, 3H) ppm. ^{13}C NMR (125 MHz, CD_3OD) δ 169.5, 168.9, 154.7, 129.5, 128.1, 127.1, 121.5, 111.1, 67.6, 66.0, 56.3, 38.6, 31.1, 19.7, 12.7 ppm. ^{1}H NMR (500 MHz, DMSO-d_6) δ 8.22 (t, *J* = 5.6 Hz, 1H), 7.44 (dd, *J* = 7.6, 1.3 Hz, 1H), 7.29-7.24 (t, *J* = 7.3 Hz, 1H), 7.00 (t, *J* = 7.3 Hz, 1H), 6.86 (d, *J* = 8.0 Hz, 1H), 6.02 (d, *J* = 4.7 Hz, 1H), 5.27 (t, *J* = 4.7 Hz, 1H), 4.66 (dd, *J* = 46.3, 15.2 Hz, 2H), 4.13 (d, *J* = 4.8 Hz, 1H), 3.11 (m, 2H), 1.45-1.35 (m, 2H), 1.33-1.17 (m, 2H), 0.86 (t, *J* = 7.3 Hz, 3H) ppm. ^{13}C NMR (125 MHz, DMSO) δ 168.0, 154.2, 128.9, 127.9, 127.8, 120.9, 111.1, 66.7, 66.1, 55.5, 38.1, 31.0, 19.4, 13.6 ppm. HRMS (ESI) $[M+Na]^+$ calcd. $C_{15}H_{23}N_3NaO_4$ 332.1586, found 332.1605.

Synthesis of ethyl 2-(2-formylphenoxy) acetate S3. In a 25 ml round bottom flask, 2-hydroxy benzaldehyde **S1** (366 mg, 3 mmol), ethyl 2-bromoacetate **S2** (1 g, 6 mmol) and K_2CO_3 (828 mg, 6 mmol) was dissolved in acetone (6 ml). The reaction mixture was allowed to reflux for 6 h. The reaction was monitored using thin layer chromatography and upon completion, the reaction mixture was filtered to remove potassium carbonate. The solution was concentrated under vacuum and the product was purified using flash column chromatography (ethyl acetate:n-hexane 2:98) to afford ethyl 4-(4-formylphenoxy) acetate **S3** (77% yield, 480 mg). TLC (ethyl acetate:n-hexane 10:90), ^{1}H NMR (500 MHz, $CDCl_3$) δ 10.56 (s, 1H), 7.85 (dd, *J* = 7.7, 1.8 Hz, 1H), 7.56-7.46 (m, 1H), 7.14-7.01 (m, 1H), 6.87 (t, *J* = 11.7 Hz, 1H), 4.74 (s, 2H), 4.26 (q, *J* = 7.1 Hz, 2H), 1.29 (t, *J* = 10.9, 3H) ppm. ^{13}C NMR (125 MHz, $CDCl_3$) δ 189.5, 168.1, 160.1, 135.7, 128.6, 125.4, 121.8, 112.6, 65.6, 61.6, 14.1 ppm. HRMS (ESI) $[MH]^+$ calcd. $C_{11}H_{12}NaO_4$ 231.0633, found 231.0656.

Synthesis of 2-(2-formylphenoxy) acetic acid 2e. In a 25ml round bottom flask, ethyl 4-(4-formylphenoxy) acetate **S3** (440 mg, 2.1 mmol), was mixed with water (1 ml). To this solution trifluoro acetic acid (964 mg, 8.4 mmol) was added drop wise at 0-5 °C. The reaction mixture was allowed to reflux for 12 h. The reaction was monitored using thin layer chromatography and upon completion of the reaction,

the solution was concentrated under vacuum and the product was purified using flash column chromatography (ethyl acetate: n-hexane 50:50) to afford ethyl 4-(4-formylphenoxy) acetate **2e** (63% yield, 240 mg). TLC (ethyl acetate:n-hexane 40:60), ^{1}H NMR [500 MHz, $(CD_3)_2CO$] δ 10.55 (s, 1H), 7.78 (dd, *J* = 7.7, 1.8 Hz, 1H), 7.63 (m, 1H), 7.19 (d, *J* = 8.5 Hz, 1H), 7.15-7.09 (m, 1H), 4.94 (s, 2H) ppm. ^{13}C NMR [125 MHz, $(CD_3)_2CO$] δ 189.6, 169.7, 161.4, 136.7, 128.5, 126.3, 122.3, 114.5, 65.9 ppm. HRMS (ESI) $[M+Na]^+$ calcd. C_9H_8 NaO_4 203.0320, found 203.0330.

Synthesis of 3-(2-formylphenyl)propanoic acid 2b. In a 25 ml Ace pressure tube, β-tetralone **S4** (146 mg, 1 mmol), $FeCl_3$ (16.2 mg, 1 mmol), H_2O (1 mmol), and DMSO (2 ml) was added. Then the tube was pressurized with air, after that it was stirred at 110°C for 20 h. The reaction was monitored using thin layer chromatography and upon completion of the reaction, the reaction mixture was purified by column chromatography (MeOH:DCM 0.5:99.5) to afford **2b** (36% yield, 64 mg). TLC (MeOH:DCM 10:90), ^{1}H NMR (500 MHz, $CDCl_3$) δ 10.19 (s, 1H), 7.82 (dd, *J* = 7.6, 1.3 Hz, 1H), 7.53 (t, *J* = 7.5, 1H), 7.44 (t, *J* = 7.5, 1H), 7.34 (d, *J* = 7.5 Hz, 1H), 3.36 (t, *J* = 7.6 Hz, 2H), 2.70 (t, *J* = 7.6 Hz, 2H) ppm. ^{13}C NMR (125 MHz, $CDCl_3$) δ 192.9, 178.0, 142.4, 134.1, 133.9, 133.8, 131.2, 127.2, 35.1, 27.9 ppm. HRMS (ESI) $[M+Na]^+$ calcd. $C_{10}H_{10}NaO_3$ 201.0528, found 201.0548.

Synthesis of 3-(2-formylphenoxy)propanoic acid 2d. In a 25ml round bottom flask, 3-bromopropanoic acid **S5** (619 mg, 4 mmol) and salicylaldehyde **S1** (500 mg, 4 mmol), was mixed with water (8 ml). To this sodium hydroxide solution (964 mg, 8 mmol) was added drop wise at 0-5 °C with vigorous stirring. The reaction mixture was allowed to reflux for 6 h. The reaction was monitored using thin layer chromatography and upon completion of the reaction, neutralize the aqueous layer with the slow addition of conc.HCl with constant stirring. The solution was concentrated under vacuum and the product was purified by using column chromatography ($CHCl_3$) to afford 3-(2-formylphenoxy)propanoic acid **2d** (39% yield, 300 mg). TLC (MeOH:$CHCl_3$ 5:95), ^{1}H NMR (500 MHz, $CDCl_3$) δ 10.44 (s, 1H), 7.83 (dd, *J* = 7.7, 1.7 Hz, 1H), 7.58-7.53 (m, 1H), 7.05 (t, *J* = 7.5 Hz, 1H), 7.01 (d, *J* = 8.4 Hz, 1H), 4.37 (t, *J* = 6.1 Hz, 2H), 2.93 (t, *J* = 6.1 Hz, 2H) ppm. ^{13}C NMR (125 MHz, $CDCl_3$) δ 189.9, 175.8, 160.7, 136.0, 128.5, 124.9, 121.2, 112.6, 63.8, 34.1 ppm. HRMS (ESI) $[M+Na]^+$ calcd. $C_{10}H_{10}NaO_4$ 217.0477, found 217.0486.

Synthesis of 4-(2-formylphenoxy)butanoic acid 2c. This compound is synthesized according to the synthesis of compound **2e**. 60% yield; MeOH:$CHCl_3$ 0.5:99.5, ^{1}H NMR (500 MHz, $CDCl_3$) δ 10.47 (s, 1H), 7.83 (dd, *J* = 7.7, 1.7 Hz, 1H), 7.56-7.51 (m, 1H), 7.02 (t, *J* = 7.5 Hz, 1H), 6.97 (d, *J* = 8.4 Hz, 1H), 4.16 (t, *J* = 6.0 Hz, 2H), 2.62 (t, *J* = 7.0 Hz, 2H), 2.25-2.18 (m, 2H) ppm. ^{13}C NMR (125 MHz, $CDCl_3$) δ 189.8, 178.3, 161.1, 136.0, 128.5, 124.8, 120.8, 112.3, 67.2, 30.6, 24.2 ppm. HRMS (ESI) $[M+Na]^+$ calcd. $C_{11}H_{12}NaO_4$ 231.0633, found 231.0637.

Synthesis of N-butyl-2-(2-formylphenoxy)acetamide S25b. The n-butylamine **S24** (10 mmol, 730 mg) was dissolved in DCM (10 ml), in a 100 ml round bottom flask and K_2CO_3 (4.1 g, 30 mmol) in 20 ml of H_2O was added to it. Bromoacetyl bromide **S9** (30 mmol, 6 g), dissolved in DCM (20 ml) was added drop wise to the mixture at 0-5 ^oC. The reaction mixture was stirred for 12 h and the reaction progress was analyzed using thin layer chromatography. On completion of the reaction, reaction mixture was extracted with DCM. The collected organic fractions were dried over anhydrous sodium sulfate and filtered; the filtrate was concentrated under reduced pressure to afford 2-bromo-N-butylacetamide **S25a** as a red-yellow liquid. Further the crude material was utilized for the synthesis of **S25b**. In a 100 ml round bottom flask, 2-hydroxy benzaldehyde **S1** (1.97 g, 16.2 mmol), 2-bromo-N-butylacetamide **S25a** (1.0 g, 5.4 mmol) and K_2CO_3 (2.23 g, 16.2 mmol) dissolved in acetonitrile (54 ml). The reaction mixture was allowed to reflux for 12 h. The reaction was monitored using thin layer chromatography and upon completion, the reaction mixture was filtered to remove potassium carbonate. The solution was concentrated under vacuum and the product was purified using flash column chromatography (MeOH:DCM 3:97) to afford N-butyl-2-(2-formylphenoxy)acetamide **S25b** (70% yield, 0.88 g). TLC (MeOH:DCM 10:90), ^{1}H NMR (500 MHz, $CDCl_3$) δ 10.17 (s, 1H), 7.78 (dd, *J* = 7.6, 1.8 Hz, 1H), 7.60 (m, 2H), 7.18 (t, *J* = 7.5 Hz, 1H), 6.94 (d, *J* = 8.3 Hz, 1H), 4.57 (s, 2H), 3.39 (m, 2H), 1.65-1.57 (m, 2H), 1.42 (m, 2H), 0.97-0.92 (m, 3H) ppm. ^{13}C NMR (125 MHz, $CDCl_3$) δ 190.1, 167.2, 157.6, 135.9, 134.0, 124.8, 121.7, 112.9, 67.4, 38.8, 31.3, 19.9, 13.5 ppm. HRMS (ESI) $[M+H]^+$ calcd. $C_{13}H_{18}NO_3$ 236.1287, found 236.1281.

Synthesis of N,N'-(ethane-1,2-diyl)bis(2-bromoacetamide) S10. The ethane-1,2-diamine **S8** (10 mmol, 670 mg) was dissolved in DCM (10 ml), in a 100 ml round bottom flask and K_2CO_3 (4.1 g, 30 mmol) in 20 ml of H_2O was added to it. Bromoacetyl bromide **S9** (30 mmol, 6 g), dissolved in DCM (20 ml) was added drop wise to the mixture at 0-5 ^oC. The reaction mixture was stirred for 12 h and the reaction progress was analyzed using thin layer chromatography. On completion of the reaction, reaction mixture was extracted with DCM. The collected organic fractions were dried over anhydrous sodium sulfate and filtered, the filtrate was concentrated under reduced pressure to afford N,N'-(ethane-1,2-diyl)bis(2-bromoacetamide) **S10** as a white solid (95% yield, 2.85 g). ^{1}H NMR (500 MHz, $CDCl_3$) ^{1}H NMR (500 MHz, $CDCl_3$) δ 3.88 (s, 2H), 3.50-3.47 (m, 2H) ppm. ^{13}C NMR (125 MHz, $CDCl_3$) δ 166.7, 40.2, 28.8 ppm. HRMS (ESI) $[M+H]^+$ calcd. $C_6H_{11}Br_2N_2O_2$ 300.9187, found 300.9182.

Synthesis N,N'-(ethane-1,2-diyl)bis(2-(2-formylphenoxy)acetamide) 2f. In a 100 ml round bottom flask, 2-hydroxy benzaldehyde **S1** (1.97 g, 16.2 mmol), N,N'-(ethane-1,2-diyl)bis(2-bromoacetamide) **S10** (1.6 g, 5.4 mmol) and K_2CO_3 (2.23 g, 16.2 mmol) dissolved in acetonitrile (54 ml). The reaction mixture was allowed to reflux for 12 h. The reaction was monitored using thin layer chromatography and upon

completion, the reaction mixture was filtered to remove potassium carbonate. The solution was concentrated under vacuum and the product was purified using flash column chromatography (MeOH:DCM 3:97) to afford N,N'-(ethane-1,2-diyl)bis(2-(2-formylphenoxy)acetamide) **2f** (78% yield, 1.56 g). TLC (MeOH:DCM 10:90), ^{1}H NMR (500 MHz, $CDCl_3$) δ 10.11 (s, 1H), 7.72 (dd, *J* = 7.6, 1.7 Hz, 1H), 7.58 (t, *J* = 8.4, 1H), 7.14 (t, *J* = 7.2 Hz, 1H), 6.92 (d, *J* = 8.3 Hz, 1H), 4.58 (s, 2H), 3.66-3.62 (m, 2H) ppm. ^{13}C NMR (125 MHz, $CDCl_3$) δ 190.5, 168.3, 158.1, 136.1, 133.3, 124.9, 121.9, 113.0, 67.5, 39.3 ppm. HRMS (ESI) $[M+Na]^+$ calcd. $C_{20}H_{20}N_2NaO_6$ 407.1219, found 407.1214.

Synthesis of compound S12. This compound is synthesized according to the synthesis of compound **S10.** Yield 72%; TLC (MeOH: DCM 10:90), ^{1}H NMR (500 MHz, $CDCl_3$) δ 3.85 (s, 4H), 3.68-3.64 (m, 4H), 3.64-3.56 (m, 8H), 3.41 (dd, *J* = 12.2, 5.8 Hz, 4H), 1.81 (dd, *J* = 11.5, 5.8 Hz, 4H) ppm. ^{13}C NMR (125 MHz, $CDCl_3$) δ 165.4, 70.5, 70.3, 70.3, 38.9, 29.3, 28.5 ppm. HRMS (ESI) $[M+H]^+$ calcd. $C_{14}H_{27}Br_2N_2O_5$ 463.0287, found 463.0267.

Synthesis of compound 2g. This compound is synthesized according to the synthesis of compound **2f.** Yield 73%; MeOH : DCM 10:90, ^{1}H NMR (500 MHz, $CDCl_3$) δ 10.25 (s, 2H), 7.77 (d, *J* = 7.6 Hz, 2H), 7.59-7.53 (m, 2H), 7.13 (t, *J* = 7.5 Hz, 2H), 6.92 (d, *J* = 8.4 Hz, 2H), 4.55 (s, 4H), 3.56-3.51 (m, 10H), 3.48-3.42 (m, 4H), 1.87-1.80 (m, 4H) ppm. ^{13}C NMR (125 MHz, $CDCl_3$) δ 190.0, 167.3, 158.4, 136.1, 132.6, 125.0, 121.9, 113.0, 70.4, 70.2, 69.3, 67.6, 37.0, 29.2 ppm. HRMS (ESI) $[M+H]^+$ calcd. $C_{28}H_{36}N_2O_9$ 545.2499, found 545.2508.

Synthesis of 2-(3-bromopropoxy)isoindoline-1,3-dione S15[6]. In a 250 ml round bottom flask, N-hydroxyphthalimide **S13** (4894 mg, 30 mmol) and triethyl amine (6.09 ml, 60 mmol) were dissolved in ACN (60 ml). To this solution, 1,3-dibromo propane **S14** (8.34 ml, 60 mmol) was added and stirred at 25 ^{o}C for 16 h. The reaction mixture was concentrated in vacuo and was added 1 N NaOH solution and ethyl acetate. The organic layer was separated, dried over anhydrous sodium sulfate, filtered and concentrated in vacuo. Purification of the crude mixture by flash column chromatography using ethyl acetate:n-hexane (3:97) gave **S15** in 50% yield. ^{1}H NMR (400 MHz, $CDCl_3$) δ 7.89-7.81 (m, 2H), 7.80-7.73 (m, 2H), 4.37 (t, *J* = 5.8 Hz, 2H), 3.71 (t, *J* = 6.5 Hz, 2H), 2.31 (m, 2H) ppm. ^{13}C NMR (100 MHz, CDCl3) δ 163.7, 134.7, 129.0, 123.7, 76.2, 31.6, 29.4 ppm. HRMS (ESI) $[MH]^+$ calcd. $C_{11}H_{11}{}^{79}BrNO_3$ 283.9922, found 283.9917 and calcd. $C_{11}H_{11}{}^{81}BrNO_3$ 285.9902, found 285.9893.

Synthesis of compound S17. In a 25 ml round bottom flask, 7-Mercapto-4-methylcoumarin **S16** (192 mg, 1 mmol), K_2CO_3 (276 mg, 2 mmol) and 2-(3-bromopropoxy)isoindoline-1,3-dione **S15** (568 mg, 2 mmol) were dissolved in degassed acetonitrile (5 ml) and refluxed for 16 h. The reaction mixture was concentrated in vacuo and purified by silica gel flash column chromatography using ethyl acetate:n-hexane (7:3) to give

S17 in 95% yield. ^{1}H NMR (400 MHz, $CDCl_3$) δ 7.90-7.82 (m, 2H), 7.81-7.73 (m, 2H), 7.50 (d, *J* = 8.2 Hz, 1H), 7.26-7.20 (m, 2H), 6.22 (d, *J* = 0.8 Hz, 1H), 4.36 (t, *J* = 5.8 Hz, 2H), 3.35 (t, *J* = 7.1 Hz, 2H), 2.41 (d, *J* = 0.9 Hz, 3H), 2.23-2.08 (m, 2H). ^{13}C NMR (100 MHz, $CDCl_3$) δ 163.8, 160.7, 154.0, 152.3, 142.6, 134.7, 129.0, 124.9, 123.8, 123.4, 117.5, 114.8, 114.1, 76.6, 28.7, 27.8, 18.7 ppm. HRMS (ESI) $[MH]^+$ calcd. $C_{21}H_{18}NO_5S$ 396.0906, found 396.0925.

Synthesis of 7-((3-(aminooxy)propyl)thio)-4-methyl-2H-chromen-2-one 10c. 2-(3-((4-methyl-2-oxo-2H-chromen-7-yl)thio)propoxy)isoindoline-1,3-dione **S17** (237 mg, 0.6 mmol) in 5 ml round bottom flask was dissolved in CH_2Cl_2 (12 ml). To this solution, hydrazine monohydrate (80%, 29 µl, 0.6 mmol) was added and stirred at 25 ^{o}C for 3 h. The reaction mixture was filtered and the filtrate was concentrated. Purification of crude mixture by reverse phase preparative HPLC gave **10c** (76 mg, 45% yield). ^{1}H NMR (400 MHz, $CDCl_3$) δ 7.44 (d, *J* = 8.3 Hz, 1H), 7.20-7.11 (m, 2H), 6.18 (d, *J* = 0.9 Hz, 1H), 3.77 (t, *J* = 5.9 Hz, 2H), 3.05 (t, *J* = 7.3 Hz, 2H), 2.38 (d, *J* = 0.8 Hz, 3H), 2.02-1.90 (m, 2H) ppm. ^{13}C NMR (100 MHz, $CDCl_3$) δ 160.7, 154.0, 152.3, 143.3, 124.7, 123.1, 117.2, 114.1, 113.9, 73.9, 29.0, 27.8, 18.7 ppm. HRMS (ESI) $[MH]^+$ calcd. $C_{13}H_{16}NO_3S$ 266.0851, found 266.0841.

Synthesis of 3-((1,3-dioxoisoindolin-2-yl)oxy)propyl 3,5-bis(trifluoromethyl)benzoate S19. In a 25 ml round bottom flask, 3,5-bis(trifluoromethyl)benzoic acid **S18** (258 mg, 1 mmol), 2-(3-bromopropoxy)isoindoline-1,3-dione **S15** (312 mg, 1.1 mmol) and TEA (418 µl, 3 mmol) were dissolved in acetonitrile (5 ml) to reflux. Progress of the reaction was followed by TLC. After 8 h, reaction mixture was concentrated and purification of crude by flash column chromatography (ethyl acetate:n-hexane, 2:98) gave 3-((1,3-dioxoisoindolin-2-yl)oxy)propyl 3,5-bis(trifluoromethyl)benzoate **S19** (335 mg, 73% yield). ^{1}H NMR (400 MHz, $CDCl_3$) δ 8.51 (s, 2H), 8.05 (s, 1H), 7.90-7.70 (m, 4H), 4.70 (t, *J* = 6.3 Hz, 2H), 4.39 (t, *J* = 6.0 Hz, 2H), 2.29 (m, 2H) ppm. ^{13}C NMR (101 MHz, $CDCl_3$) δ 164.0, 163.7, 134.7, 132.5, 132.3 (q, *J* = 34.1 Hz, 2C), 130.1-129.8 (m, 2C), 129.0, 126.6-126.6 (m, 1C), 123.7, 123.0 (q, *J* = 272.8 Hz, 2C), 74.9, 62.7, 27.8 ppm. ^{19}F NMR (376 MHz, $CDCl_3$) δ -62.94 [Trifluoro acetic acid (TFA) was used as an internal standard, -75.70 ppm] ppm. HRMS (ESI) $[MH]^+$ calcd. $C_{20}H_{14}F_6NO_5$ 462.0776, found 462.0775.

Synthesis of 3-(aminooxy)propyl 3,5-bis(trifluoromethyl)benzoate 10a. In a 5 ml round bottom flask, 3-((1,3-dioxoisoindolin-2-yl)oxy)propyl 3,5-bis(trifluoromethyl)benzoate **S19** (138 mg, 0.3 mmol) in DCM (3 ml) was added hydrazine monohydrate (80%, 37 µl, 0.75 mmol) and stirred at room temperature. The progress of the reaction was followed by TLC. After 3 h, reaction mixture was filtered and concentration of filtrate in vacuo gave 3-(aminooxy)propyl 3,5-bis(trifluoromethyl)benzoate **10a** (80 mg, 81% yield). ^{1}H NMR (400 MHz, $CDCl_3$) δ 8.48 (s, 2H), 8.07 (s, 1H), 5.42 (bs, 2H), 4.50 (t, *J* = 6.5 Hz, 2H),

3.83 (t, J = 6.1 Hz, 2H), 2.11 (m, 2H) ppm. ^{13}C NMR (101 MHz, $CDCl_3$) δ 164.1, 132.6, 132.4 (q, J = 33.9 Hz, 2C), 130.0-129.7 (m, 2C), 126.6-126.3 (m, 1C), 123.02 (q, J = 273.0 Hz, 2C), 72.2, 63.6, 27.9 ppm. ^{19}F NMR (376 MHz, $CDCl_3$) δ -62.54 (TFA was used as an internal standard, -75.70 ppm) ppm. HRMS (ESI) $[MH]^+$ calcd. $C_{12}H_{12}F_6NO_3$ 332.0721, found 332.0699.

Synthesis of compound S21. In 5 ml round bottom flask, biotin (244 mg, 1mmol), 2-(3-bromopropoxy)isoindoline-1,3-dione **S15** (568 mg, 2 mmol) and DBU (304 µl, 2 mmol) were dissolved in acetonitrile (20 ml) to reflux. The progress of the reaction was analyzed by TLC. After 16 h, reaction mixture was concentrated on vacuum and carried out for ethyl acetate and water work up. The collected organic fractions were dried on anhydrous sodium sulfate, filtered and concentrated on rotary evaporator. Purification of crude reaction mixture by flash chromatography (MeOH/DCM, 0.5-5%) gave 3-((1,3-dioxoisoindolin-2-yl)oxy)propyl 5-(2-oxohexahydro-1H-thieno[3,4-d]imidazol-4-yl)pentanoate **S21** (224 mg, 50% yield).^{1}H NMR (500 MHz, $CDCl_3$) δ 7.87-7.80 (m, 2H), 7.78-7.71 (m, 2H), 5.95 (s, 1H), 5.46 (s, 1H), 4.48 (dd, J = 15.0, 9.8 Hz, 1H), 4.38-4.23 (m, 5H), 3.20-3.11 (m, 1H), 2.89 (dd, J = 12.8, 5.0 Hz, 1H), 2.72 (d, J = 12.8 Hz, 1H), 2.34 (t, J = 7.5 Hz, 2H), 2.16-2.05 (m, 2H), 1.80-1.60 (m, 4H), 1.53-1.37 (m, 2H) ppm. ^{13}C NMR (126 MHz, $CDCl_3$) δ 173.7, 163.7, 163.7, 134.7, 128.9, 123.7, 75.1, 62.0, 60.7, 60.2, 55.5, 40.7, 33.9, 28.4, 28.3, 27.8, 24.9 ppm. HRMS (ESI) $[MH]^+$ calcd. $C_{21}H_{26}N_3O_6S$ 448.1542, found 448.1548.

Synthesis of compound 10b. This compound is synthesized according to the synthesis of compound **10a**. ^{1}H NMR (500 MHz, D_2O) δ 4.63 (dd, J = 7.9, 4.9 Hz, 1H), 4.45 (dd, J = 7.9, 4.5 Hz, 1H), 4.21 (t, J = 6.3 Hz, 2H), 3.90 (t, J = 6.2 Hz, 2H), 3.42-3.31 (m, 1H), 3.02 (dd, J = 13.1, 5.0 Hz, 1H), 2.80 (d, J = 13.0 Hz, 1H), 2.44 (t, J = 7.3 Hz, 2H), 2.08-1.94 (m, 2H), 1.84-1.55 (m, 4H), 1.53-1.37 (m, 2H) ppm. ^{13}C NMR (126 MHz, D_2O) δ 176.9, 165.3, 72.4, 62.1, 62.0, 60.3, 55.3, 39.7, 33.6, 27.9, 27.6, 26.7, 24.1 ppm. HRMS (ESI) $[MH]^+$ calcd. $C_{13}H_{24}N_3O_4S$ 318.1488, found 318.1467.

Synthesis of amino acid amides 1. The amino acid (4 mmol) was dissolved in methanol (10 ml), the solution was cooled to 0 ºC and thionyl chloride (8 mmol) was added drop wise. The reaction mixture was heated to reflux, stirred for 6 h-12 h and cooled to room temperature. Solvents were evaporated under reduced pressure, and the resulting product was used in the next step without further purification (95% yield). The amino acid ester hydrochloride (4 mmol) was dissolved in ammonia solution (2 ml) and the reaction mixture was stirred at room temperature for 2-4 h. Solvents were evaporated under reduced pressure and resulting in amino acid amide **1**.

Synthesis of 2-aminoacetamide 1a. This compound is synthesized according to the synthesis of amino acid amides **1**. ^{1}H NMR (500 MHz, D_2O) δ 3.75 (d, J = 7.5 Hz, 1H) ppm. ^{13}C NMR (125 MHz, D_2O) δ 169.3, 40.0 ppm. HRMS (ESI) $[M+H]^+$ calcd. $C_2H_7N_2O$ 75.0558, found 75.0556.

Synthesis of (S)-2-aminopropanamide 1b. This compound is synthesized according to the synthesis of amino acid amides **1**. ^{1}H NMR (500 MHz, D_2O) δ 4.13 (q, *J* = 6.0 Hz, 1H), 1.55 (d, *J* = 18.1 Hz, 3H) ppm. ^{13}C NMR (125 MHz, D_2O) δ 173.1, 48.8, 16.4 ppm. MS (ESI) $[M+H]^+$ calcd. $C_3H_9N_2O$ 89.1, found 89.1.

Synthesis of (S)-2-amino-5-guanidinopentanamide 1c. This compound is synthesized according to the synthesis of amino acid amides **1**. ^{1}H NMR (500 MHz, D_2O) δ 4.07 (t, *J* = 6.5 Hz, 1H), 3.28 (t, *J* = 6.8 Hz, 2H), 2.0-1.90 (m, 2H), 1.68 (m, 2H) ppm. ^{13}C NMR (125 MHz, D_2O) δ 172.1, 156.7, 52.6, 40.3, 28.0, 23.5 ppm. HRMS (ESI) $[M+H]^+$ calcd. $C_6H_{16}N_5O$ 174.1355, found 174.1341.

Synthesis of (S)-2-aminosuccinamide 1d. This compound is synthesized according to the synthesis of amino acid amides **1**. ^{1}H NMR (500 MHz, D_2O) δ 4.02 (dd, *J* = 7.8, 4.2 Hz, 1H), 2.97 (dd, *J* = 16.9, 4.2 Hz, 1H), 2.87 (dd, *J* = 16.9, 7.8 Hz, 1H) ppm. ^{13}C NMR (125 MHz, D_2O) δ 174.4, 173.2, 51.2, 34.4 ppm. HRMS (ESI) $[M+H]^+$ calcd. $C_4H_{10}N_3O_2$ 132.0773, found 132.0779.

Synthesis of (R)-2-amino-3-mercaptopropanamide 1f. This compound is synthesized according to the synthesis of amino acid amides **1**. ^{1}H NMR (500 MHz, D_2O) δ 3.92 (m, 1H), 3.08-2.92 (m, 2H) ppm. ^{13}C NMR (125 MHz, D_2O) δ 172.3, 55.8, 24.7 ppm. HRMS (ESI) $[M+H]^+$ calcd. $C_3H_9N_2OS$ 121.0436, found 121.0438.

Synthesis of (S)-2-aminopentanediamide 1h. This compound is synthesized according to the synthesis of amino acid amides **1**. ^{1}H NMR (500 MHz, D_2O) δ 4.34 (dd, *J* = 9.1, 5.2 Hz, 1H), 2.64-2.37 (m, 3H), 2.18-2.06 (m, 1H) ppm. ^{13}C NMR (125 MHz, D_2O) δ 182.2, 177.8, 56.6, 29.2, 25.2 ppm. HRMS (ESI) $[M+H]^+$ calcd. $C_5H_{11}N_3NaO_2$ 168.0749, found 168.0745.

Synthesis of (S)-2-amino-3-(1H-imidazol-4-yl)propanamide 1i. This compound is synthesized according to the synthesis of amino acid amides **1**. ^{1}H NMR (500 MHz, D_2O) δ 7.70 (s, 1H), 7.00 (s, 1H), 3.93 (dd, J = 8.0, 4.8 Hz, 1H), 3.17 (dd, J = 15.4, 4.6 Hz, 1H), 3.07 (dd, J = 15.5, 8.0 Hz, 1H) ppm. ^{13}C NMR (125 MHz, D_2O) δ 174.0, 136.3, 132.2, 54.7, 28.1 ppm. HRMS (ESI) $[M+H]^+$ calcd. $C_6H_{11}N_4O$ 155.0933, found 155.0950.

Synthesis of (2S,3S)-2-amino-3-methylpentanamide 1j. This compound is synthesized according to the synthesis of amino acid amides **1**. ^{1}H NMR (500 MHz, D_2O) δ 3.82 (d, *J* = 5.5 Hz, 1H), 1.96-1.86 (m, 1H), 1.51-1.35 (m, 1H), 1.24-1.12 (m, 1H), 0.94 (dd, *J* = 12.1, 7.0 Hz, 3H), 0.87 (q, *J* = 7.3 Hz, 3H) ppm. ^{13}C NMR (125 MHz, D_2O) δ 174.1, 59.5, 35.8, 24.4, 14.6, 11.0 ppm. HRMS (ESI) $[M+H]^+$ calcd. $C_6H_{15}N_2O$ 131.1184, found 131.1192.

Synthesis of (S)-2-amino-4-methylpentanamide 1k. This compound is synthesized according to the synthesis of amino acid amides **1**. ^{1}H NMR (500 MHz, D_2O) δ 3.96 (m, 1H), 1.67 (s, 4H), 0.89 (s, 9H) ppm.

^{13}C NMR (125 MHz, D_2O) δ 173.0, 51.6, 39.8, 23.8, 21.8, 20.9 ppm. HRMS (ESI) $[M+H]^+$ calcd. $C_6H_{15}N_2O$ 131.1184, found 131.1168.

Synthesis of (S)-2,6-diaminohexanamide 1l. This compound is synthesized according to the synthesis of amino acid amides **1**. ^1H NMR (500 MHz, D_2O) δ 3.76 (t, J = 6.1 Hz, 1H), 3.06 -3.01 (m, 2H), 1.96-1.85 (m, 2H), 1.78-1.68 (m, 2H), 1.56-1.38 (m, 2H) ppm. ^{13}C NMR (125 MHz, D_2O) δ 174.5, 54.4, 39.0, 29.8, 26.3, 21.4 ppm. HRMS (ESI) $[M+H]^+$ calcd. $C_6H_{16}N_3O$ 146.1293, found 146.1292.

Synthesis of (S)-2-amino-4-(methylthio)butanamide 1m. This compound is synthesized according to the synthesis of amino acid amides **1**. ^1H NMR (500 MHz, D_2O) δ 4.19 (t, *J* = 6.6 Hz, 1H), 2.67 (m, 2H), 2.26-2.19 (m, 2H), 2.15 (d, *J* = 2.4 Hz, 3H) ppm. ^{13}C NMR (125 MHz, D_2O) δ 171.6, 52.1, 29.9, 28.1, 13.9 ppm. HRMS (ESI) $[M+H]^+$ calcd. $C_5H_{13}N_2OS$ 149.0749, found 149.0765.

Synthesis of (S)-2-amino-3-phenylpropanamide 1n. This compound is synthesized according to the synthesis of amino acid amides **1**. ^1H NMR (500 MHz, D_2O) δ 7.40-7.30 (m, 3H), 7.27 (d, *J* = 7.2 Hz, 2H), 4.21 (t, *J* = 7.1 Hz, 1H), 3.24-3.02 (m, 2H) ppm. ^{13}C NMR (125 MHz, D_2O) δ 171.4, 135.0, 133.8, 129.4, 129.1, 128.0, 127.6, 54.1, 36.7 ppm. HRMS (ESI) $[M+H]^+$ calcd. $C_9H_{13}N_2O$ 165.1028, found 165.1034.

Synthesis of (S)-pyrrolidine-2-carboxamide 1o. This compound is synthesized according to the synthesis of amino acid amides **1**. ^1H NMR (500 MHz, D_2O) δ 4.11 (m, 1H), 3.41 (m, 1H), 3.32 (m, 1H), 2.33 (m, 1H), 2.11-1.92 (m, 3H) ppm. ^{13}C NMR (125 MHz, D_2O) δ 174.6, 61.1, 46.0, 28.9, 23.7 ppm. HRMS (ESI) $[M+H]^+$ calcd. $C_5H_{10}N_2NaO$ 137.0691, found 137.0697.

Synthesis of (S)-2-amino-3-hydroxypropanamide 1p. This compound is synthesized according to the synthesis of amino acid amides **1**. ^1H NMR (500 MHz, D_2O) δ 4.19 (m, 1H), 4.08-3.96 (m, 2H) ppm. ^{13}C NMR (125 MHz, D_2O) δ 169.9, 60.1, 54.4 ppm. HRMS (ESI) $[M+H]^+$ calcd. $C_3H_9N_2O_2$ 105.0664, found 105.0662.

Synthesis of (2S,3R)-2-amino-3-hydroxybutanamide 1q. This compound is synthesized according to the synthesis of amino acid amides **1**. ^1H NMR (500 MHz, D_2O) δ 4.13-4.05 (m, 1H), 3.70 (d, *J* = 3.9 Hz, 1H), 1.22 (d, *J* = 6.1 Hz, 3H) ppm. ^{13}C NMR (125 MHz, D_2O) δ 172.0, 66.5, 58.6, 18.6 ppm. HRMS (ESI) $[M+H]^+$ calcd. $C_4H_{11}N_2O_2$ 119.0821, found 119.0825.

Synthesis of (S)-2-amino-3-(1H-indol-3-yl)propanamide 1r. This compound is synthesized according to the synthesis of amino acid amides **1**. ^1H NMR (500 MHz, D_2O) δ 7.73 (d, J = 8.0 Hz, 1H), 7.54 (d, J = 8.2 Hz, 1H), 7.29 (m, 2H), 7.20 (t, J = 7.9 Hz, 1H), 4.04 (dd, J = 8.1, 4.8 Hz, 1H), 3.48 (dd, J = 15.3, 4.8 Hz, 1H), 3.30 (dd, J = 15.4, 8.1 Hz, 1H) ppm. ^{13}C NMR (125 MHz, D_2O) δ 174.4, 136.3, 126.6, 124.9, 122.1,

119.4, 118.4, 111.9, 107.4, 55.0, 26.3 ppm. HRMS (ESI) $[M+H]^+$ calcd. $C_{11}H_{14}N_3O$ 204.1137, found 204.1117.

Synthesis of (S)-2-amino-3-(4-hydroxyphenyl)propanamide 1s. This compound is synthesized according to the synthesis of amino acid amides **1**. ^{1}H NMR (500 MHz, D_2O) δ 7.10 (t, *J* = 8.9 Hz, 1H), 6.85-6.77 (m, 1H), 4.12 (t, *J* = 7.0 Hz, 1H), 3.12-2.98 (m, 1H) ppm. ^{13}C NMR (125 MHz, D_2O) δ 171.6, 155.0, 130.8, 125.6, 115.8, 54.2, 35.9 ppm. HRMS (ESI) $[M+H]^+$ calcd. $C_9H_{13}N_2O_2$ 181.0977, found 181.0960.

Synthesis of (S)-2-amino-3-methylbutanamide 1t. This compound is synthesized according to the synthesis of amino acid amides **1**. ^{1}H NMR (500 MHz, D_2O) δ 3.37 (t, *J* = 62.3 Hz, 1H), 2.23-1.85 (m, 1H), 0.90 (d, *J* = 24.5 Hz, 6H) ppm. ^{13}C NMR (125 MHz, D_2O) δ 174.2, 60.3, 29.0, 17.9, 16.5 ppm.

Synthesis of compound S26. A solution of 4,7,10-trioxa-1,13-tridecanediamine **S11** (34.1 mmol, 7.50 g) in 1,4-dioxane (100 mL) in RBF immersed in a RT water bath was treated with Boc anhydride (16.9 mmol, 3.70 g) and the reaction mixture was stirred at room temperature overnight. The solvent was removed under reduced pressure and the residue was purified by silica gel chromatography (MeOH/$CHCl_3$ 3:97) to afford *t*ert-butyl (1-bromo-2-oxo-7,10,13-trioxa-3-azahexadecan-16-yl)carbamate **S26** (34% yield, 1.86 g). TLC (MeOH/$CHCl_3$ 10: 90), ^{1}H NMR (500 MHz, $CDCl_3$) δ 3.63-3.60 (m, 4H), 3.61-3.56 (m, 4H), 3.56-3.52 (m, 4H), 3.22 (d, *J* = 6.0 Hz, 2H), 2.79 (t, *J* = 6.7 Hz, 2H), 1.79-1.69 (m, 4H), 1.45 (s, 9H) ppm. ^{13}C NMR (125 MHz, $CDCl_3$) δ 156.0, 78.7, 70.5, 70.5, 70.2, 70.1, 69.5, 69.4, 39.5, 38.4, 33.3, 29.5, 28.4 ppm. MS (ESI) $[MH]^+$ calcd. $C_{15}H_{32}N_2NaO_5$ 343.2, found 343.1.

Synthesis of compound S26a. Tert-butyl (3-(2-(2-(3-aminopropoxy)ethoxy)ethoxy)propyl)carbamate **S26** (4.7 mmol, 1.5g) was dissolved in DCM (3 ml), in a 50 ml round bottom flask and K_2CO_3 (7 mmol, 1 g) in 3 ml of H_2O was added to it. Bromoacetyl bromide **S9** (7 mmol, 1.4 g), dissolved in DCM (3 ml), was added drop wise to the mixture at 0-5 ^{o}C. The reaction mixture was stirred for 12 h and the progress of the reaction was analyzed by using thin layer chromatography. Upon completion, the reaction mixture was extracted with DCM and the solution was concentrated under vacuum. The product was purified using silica gel column chromatography (MeOH/$CHCl_3$ 3:97) to afford tert-butyl (1-bromo-2-oxo-7,10,13-trioxa-3-azahexadecan-16-yl)carbamate **S26a** (75% yield, 1.5 g). TLC (MeOH/DCM 10: 90), ^{1}H NMR (500 MHz, $CDCl_3$) δ 3.84 (s, 2H), 3.68-3.65 (m, 2H), 3.64-3.56 (m, 8H), 3.52 (t, *J* = 6.0 Hz, 2H), 3.41 (dd, *J* = 12.1, 5.8 Hz, 2H), 3.21 (d, *J* = 6.0 Hz, 2H), 1.78-1.83 (m, 2H), 1.77-1.71 (m, 2H), 1.42 (s, 9H) ppm. ^{13}C NMR (125 MHz, $CDCl_3$) δ 156.0, 78.7, 70.5, 70.5, 70.2, 70.1, 69.5, 69.4, 39.5, 38.4, 33.3, 29.5, 28.4 ppm. MS (ESI) $[M+H]^+$ calcd. $C_{17}H_{34}{}^{79}BrN_2O_6$ 441.1, found 441.1.

Synthesis of compound S26b. In a 50 ml round bottom flask, 2-hydroxybenzaldehyde **S1** (4.1 mmol, 500 mg) was dissolved in acetonitrile (8 ml). To this solution, K_2CO_3 (5.2g, 37.7 mmol) and tert-butyl (1-bromo-2-oxo-7,10,13-trioxa-3-azahexadecan-16-yl)carbamate **S26a** (6 mmol, 828 mg) were added and the reaction mixture was allowed to reflux for 12 h. The reaction was monitored using thin layer chromatography. Upon completion, the reaction mixture was filtered to remove potassium carbonate. The solution was concentrated under vacuum and the product was purified using silica gel column chromatography (MeOH/DCM 5:95) to afford tert-butyl (1-(2-formylphenoxy)-2-oxo-7,10,13-trioxa-3-azahexadecan-16-yl)carbamate **S26b** (53% yield, 1.0 g). TLC (MeOH/DCM 10: 90), ^{1}H NMR (500 MHz, $CDCl_3$) δ 10.25 (s, 1H), 7.79 (dd, *J* = 7.6, 1.8 Hz, 1H), 7.58 (m, 1H), 7.16 (t, *J* = 7.3 Hz, 1H), 6.94 (d, *J* = 8.3 Hz, 1H), 4.57 (s, 2H), 3.61-3.57 (m, 8H), 3.55-3.53 (m, 2H), 3.51-3.46 (m, 4H), 3.20 (d, *J* = 6.0 Hz, 2H), 1.90-1.85 (m, 2H), 1.78-1.68 (m, 2H), 1.42 (s, 9H) ppm. ^{13}C NMR (125MHz, $CDCl_3$) δ 190.0, 158.3, 156.05, 136.1, 132.9, 125.0, 121.9, 113.0, 78.8, 70.5, 70.5, 70.3, 70.1, 69.5, 69.4, 38.5, 37.1, 29.6, 29.2, 28.4 ppm. MS (ESI) $[MH]^+$ calcd. $C_{24}H_{38}N_2O_8$ 483.2, found 483.1.

Synthesis of compound S26d. In a 25ml round bottom flask, tert-butyl (1-(2-formylphenoxy)-2-oxo-7,10,13-trioxa-3-azahexadecan-16-yl)carbamate **S26b** (500 mg, 1.3 mmol), was mixed with dichloromethane (3 ml). To this solution trifluoro acetic acid (1 ml) was added drop wise at 0-5 ^{o}C. The reaction mixture was allowed to stir for 2 h. The reaction was monitored using thin layer chromatography and upon completion of the reaction, the solution was concentrated under vacuum to afford N-(3-(2-(2-(3-aminopropoxy)ethoxy)ethoxy)propyl)-2-(2-formylphenoxy)acetamide **S26c** (90% yield, 450 mg). HRMS (ESI) $[MH]^+$ calcd. $C_{19}H_{30}N_2O_6$ 383.2182, found 383.2192. The compound **S26d** is synthesized according to the synthesis of compound **S26a** (51% yield). TLC (MeOH/DCM 10: 90), ^{1}H NMR (500 MHz, $CDCl_3$) δ 10.25 (s, 1H), 7.80 (dd, *J* = 7.6, 1.7 Hz, 1H), 7.59 (t, *J* =1.8 Hz, 1H), 7.17 (t, *J* = 7.4 Hz, 1H), 6.94 (d, *J* = 8.3 Hz, 1H), 4.58 (s, 2H), 3.84 (s, 2H), 3.64-3.56 (m, 14H), 3.49 (m, 2H), 3.40 (m, 2H), 1.92-1.85 (m, 2H), 1.81 (m, 3H) ppm. ^{13}C NMR (125MHz, $CDCl_3$) δ 190.1, 167.4, 165.5, 158.2, 136.1, 133.1, 125.0, 122.0, 113.0, 77.2, 77.0, 76.7, 70.5, 70.4, 70.3, 70.3, 70.2, 69.3, 67.6, 39.0, 37.0, 29.3, 29.2, 28.5 ppm. HRMS (ESI) $[M+H]^+$ calcd. $C_{21}H_{32}{}^{79}BrN_2O_7$ 503.1393, found 503.1369.

Synthesis of compound S36. In a 25 ml round bottom flask, 2-bromo-N-(1-(2-formylphenoxy)-2-oxo-7,10,13-trioxa-3-azahexadecan-16-yl)acetamide **S26d** (0.5 mmol, 252 mg) was dissolved in acetonitrile (2.5 ml). To this solution, K_2CO_3 (138 mg, 1 mmol) and 7-mercapto-4-methyl-2H-chromen-2-one **S16** (0.5 mmol, 96 mg) were added and the reaction mixture was allowed to reflux for 12 h. The reaction was monitored using thin layer chromatography. Upon completion, the reaction mixture was filtered to remove potassium carbonate. The solution was concentrated under vacuum and the product was purified using silica gel column chromatography (MeOH/DCM 5:95) to afford 2-(2-formylphenoxy)-N-(1-((4-methyl-2-oxo-

2H-chromen-7-yl)thio)-2-oxo-7,10,13-trioxa-3-azahexadecan-16-yl)acetamide **S36** (30% yield, 92 mg). TLC (MeOH/DCM 10: 90), ^{1}H NMR (500 MHz, $CDCl_3$) δ 10.25 (s, 1H), 7.79 (dd, *J* = 7.6, 1.7 Hz, 1H), 7.61-7.56 (m, 1H), 7.49 (d, *J* = 8.3 Hz, 1H), 7.23-7.14 (m, 3H), 6.94 (d, *J* = 8.3 Hz, 1H), 6.22 (s, 1H), 4.58 (s, 2H), 3.69 (s, 2H), 3.66 -3.55 (m, 8H), 3.55-3.45 (m, 6H), 3.37 (dd, *J* = 12.1, 5.9 Hz, 2H), 2.40 (s, 3H), 1.93-1.85 (m, 2H), 1.79-1.66 (m, 2H) ppm. ^{13}C NMR (175MHz, $CDCl_3$) δ 190.2, 167.5, 167.2, 167.0, 160.4, 158.3, 153.8, 152.0, 136.2, 133.1, 125.1, 125.0, 122.7, 122.0, 117.9, 114.9, 114.5, 113.1, 77.2, 77.1, 76.9, 70.5, 70.5, 70.3, 70.2, 70.2, 69.3, 67.7, 38.7, 37.1, 36.5, 29.3, 28.7, 18.6 ppm. HRMS (ESI) $[M+H]^+$ calcd. $C_{31}H_{39}N_2O_9S$ 615.2376, found 615.2352.

Screening of amino acids. In a 5 ml vial, amino acid amide (0.005 mmol) was dissolved in sodium bicarbonate buffer (700 µl, 0.1 M, pH 7.8). To this solution, 2-(2-formyl phenoxy) acetic acid (0.05 mmol) in DMF (300 µl) from a freshly prepared stock solution was added, and the reaction mixture was allowed to stir at room temperature. After 24 h, the reaction mixture was concentrated by lyophilization. Subsequently, desalting was performed by addition of methanol and conversions were monitored by reversed-phase HPLC-ESI-MS.

Labeling of single protein in a mixture of proteins. Representative mixture of seven proteins - insulin, aprotinin, ubiquitin, cytochrome C, lysozyme C, β-lactoglobulin, and α-chymotrypsinogen A.
In a 1.5 ml Eppendorf tube, each protein (10 nmol) was mixed with sodium bicarbonate buffer (160 µl, 0.1 M, pH 7.8). To this solution, 2-(2-formyl phenoxy) acetic acid **2e** (5000 nmol) in DMSO (40 µl) from a freshly prepared stock solution was added and vortexed at 25 ºC. After 48 h, the reaction mixture was diluted with acetonitrile:buffer (10:90, 1500 µl). Unreacted N,N'-(((oxybis(ethane-2,1-diyl))bis(oxy))bis(propane-3,1-diyl))bis(2-(2-formylphenoxy)acetamide) and salts were removed by spin concentrator (0.5 ml 3-kDa MWCO). The modification of protein was analyzed by ESI-MS.

Single-site installation of tags. Protein (3 nmol) in sodium bicarbonate buffer (120 µl, 0.1 M, pH 7.8) was taken in a 1.5 ml Eppendorf tube. To this solution, N,N'-(((oxybis(ethane-2,1-diyl))bis(oxy))bis(propane-3,1-diyl))bis(2-(2-formylphenoxy)acetamide) **2g** (1500 nmol) in DMSO (30 µl) from a freshly prepared stock solution was added and vortexed at 25 ºC. After 24-48 h, the reaction mixture was diluted with acetonitrile:buffer (10:90, 1500 µl). Unreacted N,N'-(((oxybis(ethane-2,1-diyl))bis(oxy))bis(propane-3,1-diyl))bis(2-(2-formylphenoxy)acetamide) and salts were removed by spin concentrator (0.5 ml 3-kDa MWCO). The protein mixture was further washed with sodium bicarbonate buffer (0.1 M, pH 7.8) and concentrated to 160 µl. To the concentrated sample in sodium bicarbonate buffer, derivatives of O-hydroxylamine such as 3-(aminooxy)propyl 3,5-bis(trifluoromethyl)benzoate, 3-(aminooxy)propyl 5-((3aS,4S,6aR)-2-oxohexahydro-1H-thieno[3,4-d]imidazol-4-yl) pentanoate and 7-((3-

(aminooxy)propyl)thio)-4-methyl-2H-chromen-2-one (2 µmol) in DMSO (40 µl) from a freshly prepared stock solution was added to convert mono-labeled protein to its oxime derivative for 3-6 h. The excess of O-alkoxyamine and salts were removed by the spin concentrator. The sample was analyzed by ESI-MS. The salt-free sample was concentrated by lyophilization before subjecting it to digestion, peptide mapping, and sequencing by MS-MS.

Expression of SUMO1 and *Pf*AOS1 protein with N-Gly. Bacterial transformation: Desired *E. coli* strain was thawed [(DH5α for plasmid replication and BL21 (DE3) for protein expression]. The plasmid (1 µl) was added to the competent cells (50-100 µl) and was incubated on ice for 20 min. Subsequently, the heat shock was given at 42 ^{o}C for 40 seconds. The cells were kept on ice for 1 min, and 1 ml of LB was added to cells for recovery. The cells were incubated at 37 ^{o}C, 180 rpm for 45 min. The recovered cells were plated on LB plates containing desired antibiotics. The plates were incubated at 37 ^{o}C, overnight.

Protein purification. Primary culture was grown in LB overnight at 37 ^{o}C. The 1:100 inoculation was done from the primary culture in LB media for secondary culture. At approximately 0.6 OD (600 nm), the secondary culture was induced with IPTG (200 µM) for 4 h at 30 ^{o}C for SUMO1 and *Pf*AOS1 for overnight at 30 ^{o}C. The induced culture was spun at 9803xg for 10 min to pellet down, and the pellet was stored at -80 ^{o}C. For lysis, the cells were thawed. The pellet was resuspended in lysis buffer [20 mM Tris (pH 7.5), 150 mM NaCl, 1 mM EDTA, 50 µg/ml lysozyme, 0.2% Triton X-100, 1 mM PMSF, 1X LPA mix, 5 mM β-ME] and incubated for 10-15 min in ice with constant shaking in between. This was followed by sonication (45% Amplitude, 10 sec ON 10 sec OFF) till the solution became clear. The supernatant was collected after spinning for 30 min at 11000 rpm, 4 ^{o}C.

For binding and elution, the supernatant was transferred to column containing washed GSH beads. The protein bead binding was facilitated at 4 ^{o}C on the tumbler for 1 h. The beads were washed thrice with wash buffer [20 mM Tris (pH 7.5), 400 mM NaCl, 1 mM EDTA, 5 mM β-ME]. The protein was eluted in elution buffer [20 mM Tris (pH 8.0), 150 mM NaCl, 1 mM EDTA, 20 mM glutathione] and concentration was determined using Bradford assay.

For clipping, protein-bound beads were washed thrice with prescission protease buffer [50 mM Tris (pH 7.5), 1 mM EDTA, 1 mM DTT, 150 mM NaCl, 0.1% triton]. Prescission protease buffer and prescission protease was added to the column (1:50). The column was incubated at 4 ^{o}C overnight and the soup containing clipped protein was eluted out.

Digestion of protein. All solutions were made freshly prior to use.[1] Enzymatic digestion of modified myoglobin, SUMO1 and PfAOS1. Protein (0.1 mg) in 100 mM tris (10 µl, pH 7.8) with urea (6 M) was taken in a 1.5 ml Eppendorf tube. Tert-butanol (10 µL) was added to this solution and incubated for 3 h at

37 °C. Grade I water was used to reduce the concentration of the sample to 0.6 M. The enzyme (10 μg) dissolved in aqueous medium (10 μL) was added to this solution and the mixture was incubated at 37 °C for 18 h. The pH of digested solution was adjusted to < 6 (verified by pH paper) with trifluoroacetic acid (0.5 %). Afterwards, the sample was used for peptide mapping by MS and sequencing by MS-MS investigations. Trypsin used for digestion of myoglobin and insulin and α-Chymotrypsin used for *Pf*AOS1 and SUMO1 respectively.

Reduction of disulfide bond in modified insulin[2]

Protein (0.1 mg) in 100 μL of H_2O was taken in a 1.5 ml Eppendorf tube. To reduce the disulfide bond between chain A and chain B, dithiothreitol (10 μL, 0.2 M DTT in H_2O) was added to the modified insulin solution. The reaction mixture was incubated at 37°C for 3-6 h. The N-terminal modification of chain A was confirmed by LC-MS/MS analysis.

Procedure for *in vitro* SUMOylation. *In vitro* SUMOylation reactions contained E1 enzyme (0.25ug GST-SAE2/SAE1), E2 enzyme (1.0μg $(His)_6$-Ubc9), SUMO1 protein (4μg)/modified SUMO1 and substrate (4μg) in SUMOylation buffer (50 mM Tris, pH 7.5, 5 mM $MgCl_2$, 5 mM ATP, 5 mM DTT). SUMOylation reactions were incubated at 37°C for 4 h and terminated with 6X Laemmlli buffer. SUMOylation was analyzed by resolving reaction products on SDS-PAGE.

Analysis summary for aminoalcohol (S30): The aminoalcohol **3a** (Figure 2a) was not separable from Gly derivative **1a** in multiple attempts. To isolate the product for characterization, we transformed the carboxylic acid group of aldehyde **2e** to its *n*-butyl amide derivative (**S25b**, Figure S1). We were pleased to note that the aminoalcohol **S30** was separable from both the starting materials (**1a** and **S25b**, Figure S1). Initially, the LC-ESI-MS of crude reaction mixture confirmed the formation of an adduct (**S30**) without loss of atoms (Figure S2). The ^1H NMR and ^{13}C NMR experiments were performed with aminoalcohol **S30** in CD_3OD. Next, we performed COSY and TOCSY for the assignment of all the non-exchangeable protons. The vicinal distribution of H_a-H_b-H_c-H_d, H_k-H_l-H_m-H_n, and H_p-H_r was unambiguously determined with the help of chemical shift, coupling constants, and 2D-correlations. Subsequently, we performed DEPT-135 and HSQC experiments to assign all the carbons in the ^{13}C NMR spectrum.

Next, we performed the ^1H NMR and ^{13}C NMR experiments with aminoalcohol **S30** in DMSO-d_6. Here, we were able to visualize H_e, H_q, and H_t that were exchanged in CD_3OD. The vicinal and long-range correlations of H_e and H_q can be observed in COSY and TOCSY. Also, ^2J correlations for H_{t1}-H_{t2} and H_{h1}-H_{h2} can be noted. The HSQC experiment was performed to re-validate the previous assignments in the ^{13}C NMR spectrum. Next, the HMBC experiment was conducted to bridge the connectivity that is lost due to the heteroatoms or a carbon without an attached proton. Here, the cross-peaks of H_p-C_o, H_p-C_j, H_h-C_j, H_h-C_f, and H_d-C_f helped us complete the correlation network.

Conversion measurement techniques in protein reactions. In the past, ESI-MS [8, 9, 10] and MALDI [11, 12, 13] have been used for the estimation of conversions. We did an independent investigation of the reliability of conversions measured by the MS data. We invested efforts to separate the unreacted protein and the labeled protein on HPLC. We were able to achieve this task in the case of insulin (**6d)** and labeled insuin (**7d**) along with late-stage tagged insulin (**S28d**). The HPLC data (see Supplementary Figure 113a below) gives 90% conversion while ESI-MS data gives 88% conversion, Figure S50d. These results validate the accuracy of the data obtained by ESI-MS. We preferred ESI-MS over MALDI for all the samples that responded well to the electrospray ionization technique. It helped us avoid the errors that might emerge due to spatial heterogeneity during sample preparation for MALDI.[14]

Insulin imaging assay. The HEK293T (Cell Repository, NCCS, Pune India) cells were grown in a six-well plate with coverslips in 10% FBS containing DMEM media for 14-16 h at 37°C under 5% CO_2 conditions in a humidified chamber. For the treatment, cells were first washed with PBS and grown in serum lacking DMEM media for 12 h. Subsequently, the cells were washed twice with PBS and treated with coumarin tagged **11c** and untagged insulin **6d** (3 µg/ml, 0.5 µM) for 30 min in DMEM media lacking serum. Post-treatment, cells were again washed twice with PBS and fixed using 100% chilled methanol for 15 min. The cells were rehydrated for 10 min and nuclei were stained with Hoechst 33342 (Invitrogen) before capturing on Olympus Confocal Laser Scanning Microscope-FV3000.

Insulin bioactivity assay. The levels of activated Akt (pAkt) (#4060S) and GAPDH (Product No. 10-10011) (loading control) were determined by western blot and quantified using Gel-Quant software. The cells were treated as described in imaging assays, but here 10 µg mL^{-1} (1.7 µM) insulin was used for pathway activation. For sample preparation, cells were washed twice with PBS and lysed directly in the laemmli buffer by boiling at 100 °C for 10 min. Cell lysate thus obtained was analyzed on 8% SDS-polyacrylamide gel and transferred onto polyvinylidene difluoride (PVDF) membrane (0.45 µm pore size). The membrane was blocked with 3% BSA for 30 minutes and incubated overnight with primary antibodies pAkt antibody (1:2000) and GAPDH (1:6000, Abgenex) at 4 °C. After primary antibody incubation, the membrane was washed thrice with TBST (5 min each at room temperature) and then incubated with the HRP-conjugated (1:10,000, Genie) or Alexa flour 680 conjugated (1:40000, Invitrogen) anti-rabbit IgG secondary antibody for 1 h at room temperature. Protein bands were detected by chemiluminescence using ECL plus Western Blotting Detection System (Thermo Pierce) and fluorescence using Odyssey IR imaging system (Li-COR).

Supplementary Notes

Supplementary Note 1. General information

The reagents, proteins, and enzymes were purchased from Sigma-Aldrich, Alfa Aeser and Merck Novabiochem. Hydrazide agarose beads were purchased from Thermo Scientific. Boronic acid (polymer bound) was purchased from Sigma Aldrich. The organic solvents used were reagent grade. Aqueous buffers were prepared freshly using Millipore Grade I water (Resistivity > 5 MΩ cm, Conductivity < 0.2 μS/cm, TOC <30 ppb). Mettler Toledo (FE20) pH meter was used to adjust the final pH. The reaction mixture for the small molecules was stirred (Heidolph, 500-600 rpm). Proteins were vortexed in the incubator-shaker Thermo Scientific MaxQ 8000 (350 rpm, 25-37 °C). Amicon® Ultra-0.5 mL 3-kDa or 10-kDa MWCO Centrifugal Filters from Merck Millipore was used to remove small molecules from protein mixture, desalting and buffer exchange. Organic solvents were removed by BUCHI rotavapor R-210/215 whereas aqueous samples were lyophilized by CHRiST ALPHA 2-4 LD plus lyophilizer. Circular Dichroism (CD) measurements were recorded on JASCO J-815 CD spectropolarimeter equipped with Peltier temperature controller. All the spectra were measured with a scan speed of 50 nm/min, spectral band width 1 nm using 1 mm path length cuvette at 25 °C. UV-Vis spectra were recorded in Agilent Carry-100 UV-Vis Spectrophotometer connected with Peltier temperature controller. Steady-state fluorescence spectra was carried out in HORIBA JOBIN YVON, FLUOROLOG 3-111. The fluorescence spectra were measured with a quartz cuvette of 1 cm path length. The pAkt antibody (1:2000, CST- 4060S) and GAPDH (1:6000, Abgenex- 10-10011) were used for insulin bioactivity assay. Fluorescence images were captured on APOTOME/Zeiss LSM 780 confocal microscope. The image analysis was performed using ZEN (Zeiss) or Image J software.

Supplementary Note 2. Chromatography

Thin-layer chromatography (TLC) was performed on silica gel coated aluminium TLC plates (Merck, TLC Silica gel 60 F254). The compounds were visualized using a UV lamp (254 nm) and stains such as iodine, ninhydrin, 2,4-diphenylhydrazine. The flash column chromatography of reagents was carried out on Combiflash Rf 200 or gravity columns using 230-400 or 100-200 mesh silica gel from Merck.

Supplementary Note 3. Nuclear magnetic resonance spectra

^{1}H, ^{13}C, COSY, TOCSY, HSQC, HMBC, DEPT-135, and ^{19}F NMR spectra were recorded on Bruker Avance III 400 and 500 MHz NMR spectrometer. ^{1}H NMR spectra were referenced to TMS (0 ppm) DMSO-d_6 (2.50 ppm), D_2O (4.79 ppm) and acetone-d_6 (2.05 ppm) whereas ^{13}C NMR spectra were referenced to $CDCl_3$ (77.16 ppm), DMSO-d_6 (39.52 ppm) and acetone-d_6 (29.84 ppm). ^{19}F was referenced to trifluoro acetic acid (-75.70 ppm). Peak multiplicities are designated by the following abbreviations: s,

singlet; bs, broad singlet; d, doublet; t, triplet; q, quartet; m, multiplet; dd, doublet of doublets; ddd, doublet of doublet of doublets. Spectra were recorded at 298 K.

Supplementary Note 4. Mass spectrometry

Agilent Technologies 1200 series HPLC paired to Agilent 6130 mass spectrometer (ESI/APCI) was used for ESI-MS data. HRMS data were recorded on Bruker Daltonics MicroTOF-Q-II with electron spray ionization (ESI). Matrix assisted laser desorption/ionisation time of flight mass spectrometry was performed with Bruker Daltonics UltrafleXtreme Software-Flex control version 3.4, using sinapic acid and α-cyano-4-hydroxycinnamic acid (HCCA) matrix. Data analysis was performed using flex analysis. Peptide mass and fragment ion calculator (http://db.systemsbiology.net:8080/proteomicsToolkit/FragIonServlet.html) were used for peptide mapping and sequencing.

Acetonitrile and H_2O were buffered with 0.01% formic acid.

Method A (Column: Advance Bio Peptide 2.7 µm 4.6 × 150 mm, flow rate 0.4 mL/min)

**Time (min)**	**Acetonitrile (%)**	**H_2O (%)**
0	10	90
5	35	65
15	90	10
20	90	10

Method A was used to record the amino acid data.

Method B (Column: Agilent, Poroshell 300 SB-C18 5 µm 2.1 × 75 mm, flow rate 0.4 mL/min)

**Time (min)**	**Acetonitrile (%)**	**H_2O (%)**
0	10	90
1	10	90
8	60	40
12	90	10
15	90	10

Method B was used to record the protein data.

Method C (Column: Crownpak CR (-), 150x4 mm, 5µm, solvents H_2O/ACN, 95/5,
wavelength 215 nm, column temperature – ambient, flow 0.4 mL·min-1.

Method C was used to record the racemization profile.

Supplementary Note 5. Determination of percentage conversion

ESI-MS: Conversion for protein labeling was calculated based on the relative peak intensity of native protein and labeled protein in the deconvoluted mass spectrum.

$$\% \text{ Conversion} = I_{\text{desired product}} / I_{\text{all relevant species}} \quad (1)$$

where $I_{desired\ product}$ is the peak intensity of labeled protein, and $I_{all\ relevant\ species}$ is the sum of the peak intensities of native protein and labeled protein in the deconvoluted mass spectra.

MALDI-ToF-MS: Conversion for protein labeling was calculated based on the relative peak intensity of native protein and labeled protein in the mass spectrum.

We preferred ESI-MS data over MALDI-ToF-MS for the estimation of conversions. Also see additional results and discussion (section 5i) for detailed investigation.

Supplementary Note 6. Protein sequence

Melittin

GIGAV LKVLTTGLPALISWIKRKRQQ

Myoglobin

GLSDGEWQQVLNVWGKVEADIAGHGQEVLIRLFTGHPETLEKFDKFKHLKTEAEMKASEDLKK
HGTVVLTALGGILKKKGHHEAELKPLAQSHATKHKIPIKYLEFISDAIIHVLHSKHPGDFGADAQ
GAMTKALELFRNDIAAKYKELGFQG

SUMO1

The SUMO1 and *Pf*AOS1 protein with N-terminus glycine was expressed as per the procedure in section 2d.

Insulin

Chain A: GIVEQCCTSICSLYQLENYCN

Chain B: FVNQHLCGSHLVEALYLVCGERGFFYTPKT

Supplementary References

1. Kinter, M. & Sherman, N. E. Protein sequencing and identification using tandem mass spectrometry. *Wiley Interscience*, 2000.

2. Chan, A. O., et al. Modification of N-terminal α-amino groups of peptides and proteins using ketenes. *J. Am. Chem. Soc.* **134**, 2589-2598 (2012).

3. Velagapudi, S. P., Seedhouse, S. J., French, J. & Disney, M. D. Defining the RNA internal loops preferred by benzimidazole derivatives via 2D combinatorial screening and computational analysis. J. Am. Chem. Soc. 133, 10111-10118 (2011).

4. Xing, Q., Lv, H., Xia, C. & Li, F. Iron-catalyzed aerobic oxidative cleavage of the C-C σ-bond using air as the oxidant: chemoselective synthesis of carbon chain-shortened aldehydes, ketones and 1,2-dicarbonyl compounds. *Chem. Commun*. **52,** 489-492 (2016).

5. Hoque, J., et al. Selective and broad spectrum amphiphilic small molecules to combat bacterial resistance and eradicate biofilms. *Chem. Commun*. **51,** 13670-13673 (2015).

6. Ito, F., Ando, S., Iuchi, M., Ukari, T., Takasaki, M. & Yamaguchi, K. ^{15}N-D-labeled ionic probes for mass spectrometry. *Tetrahedron* **67,** 8009-8013 (2011).

7. Mofford, D. M., Jr. Adams, S .T., kiran, G. S., randheer, G. & Miller, S. C. Luciferin amides enable in vivo bioluminescence detection of endogenous fattyacid amide hydrolase activity. *J. Am. Chem. Soc.* **137**, 8684-8687 (2015).

8. Dai, P., et al. Salt Effect Accelerates Site-Selective Cysteine Bioconjugation. *ACS Cent. Sci.* **2,** 637-646 (2016).

9. Willwacher, J., Raj, R., Mohammed, S. & Davis, B. G. Selective Metal-Site-Guided Arylation of Proteins. *J. Am. Chem. Soc.* **138,** 8678-8681 (2016).

10. Lin, Y. A., Chalker, J. M. & Davis, B. G. Olefin Cross-Metathesis on Proteins: Investigation of Allylic Chalcogen Effects and Guiding Principles in Metathesis Partner Selection. *J. Am. Chem. Soc.* **132,** 16805-16811 (2010).

11. Takaoka, Y., Tsutsumi, H., Kasagi, N., Nakata, E. & Hamachi, I. Regulating Enzymatic Activity with a Photoswitchable Affinity Label. *J. Am. Chem. Soc.* **128,** 3273-3280 (2006).

12. Wakabayashi, H., Miyagawa, M., Koshi, Y., Takaoka, Y., Tsukiji, S. & Hamachi, I. Affinity-labeling-based introduction of a reactive handle for natural protein modification. *Chem. Asian J.* **3,** 1134-1139 (2008).

13. Takaoka, Y., Nishikawa, Y., Hashimoto, Y., Sasaki, K. & Hamachi, I. Ligand-directed dibromophenyl benzoate chemistry for rapid and selective acylation of intracellular natural proteins. *Chem. Sci.* **6,** 3217-3224 (2015).

14. Lai, Y.-H., et al. Critical factors determining the quantification capability of matrix-assisted laser desorption/ionization– time-of-flight mass spectrometry. *J. Am. Soc. Mass Spectrom.* **27,** 1314-1321 (2016).

15. Walsh, C. T., Garneau-Tsodikova, S. Jr. & Gatto. G. J. Protein posttranslational modifications: the chemistry of proteome diversifications. *Angew. Chem. Int. Ed.* **44,** 7342-7372 (2005).

16. Sletten, E. M. & Bertozzi, C. R. Bioorthogonal chemistry: fishing for selectivity in a sea of functionality. *Angew. Chem. Int. Ed.* **48,** 6974-6998 (2009).

17. Spicer, C. D. & Davis, B. G. Selective chemical protein modification. *Nat. Commun.* **5,** 4740 (2014).

18. Joshi, P. N. & Rai, V. Single-site labeling of histidine in proteins, on-demand reversibility, and traceless metal-free protein purification. *Chem. Commun*. **55,** 1100-1103 (2019).

19. Junutula, J. R., et al. Site-specific conjugation of a cytotoxic drug to an antibody improves the therapeutic index. *Nat. Biotechnol.* **26,** 925-932 (2008).

20. Tsukiji, S., Miyagawa, M., Takaoka, Y., Tamura, T. & Hamachi, I. Ligand-directed tosyl chemistry for protein labeling in vivo. *Nat. Chem. Biol.* **5,** 341-343 (2009).

21. Chen, Z., et al. Potent and selective inhibition of SH3 domains with dirhodium metalloinhibitors. *J. Am. Chem. Soc.* **134**, 10138-10145 (2012).
